# A quantum crystallographic protocol for general use

**DOI:** 10.1038/s41598-025-96400-0

**Published:** 2025-04-19

**Authors:** Yaser Balmohammadi, Lorraine A. Malaspina, Yuiga Nakamura, Georgia Cametti, Milosz Siczek, Simon Grabowsky

**Affiliations:** 1 Department of Chemistry, Biochemistry and Pharmaceutical Sciences, University of Bern, Freiestrasse 3, 3012 Bern, Switzerland; 2 Japan Synchrotron Radiation Research Institute (JASRI), Sayo-cho, Hyogo 679-5198 Japan; 3 Institute of Geological Sciences, University of Bern, Baltzerstrasse 3, 3012 Bern, Switzerland; 4 Faculty of Chemistry, University of Wrocław, F. Joliot-Curie 14, 50383 Wrocław, Poland

**Keywords:** Analytical chemistry, X-ray diffraction, Chemical physics, Structural materials

## Abstract

Quantum crystallography has become ever more important in recent years for the accurate determination of molecular and crystal structures. The results of quantum crystallographic refinements of X-ray data are as accurate and precise as from neutron diffraction and they give access to the complete electronic structure of the compound under investigation. Is the method now mature enough and easy enough to use to extend and ultimately supersede standard X-ray crystal structure determination routines? We utilize the world’s most common crystal structure—the YLID test crystal that every owner of an X-ray diffractometer receives with their machine—to show under which circumstances routine, low-resolution, and even room-temperature measurements can be subjected to quantum crystallographic refinement. Based on this, we describe a *quantum crystallographic protocol* step by step that makes application of quantum crystallographic refinement easy to use and reproducible. We encourage that valuable YLID test measurements are not discarded but made available for method development, increasing the availability of repeated measurements from a handful to tens of thousands.

## Introduction

Chemical crystallography is currently going through revolutionary developments. One step-change is the advent of three-dimensional electron diffraction for structure elucidation^1^, so that nanocrystals can now be used in chemical crystallography^2^. It provides structural details down to the possibility of localizing hydrogen atoms^3^. Another step-change is the advent of quantum crystallography for X-ray diffraction^4–6^, which allows very accurate structure determination, e.g., hydrogen atom positions and displacement parameters can be determined with the same accuracy and precision as from neutron-diffraction experiments^7–9^. Furthermore, quantum crystallography allows the derivation of experimentally reconstructed electron densities^10^ and wavefunctions^11^ that are directly related to chemical bonding and materials properties^12,13^. Importantly, quantum crystallographic techniques for X-ray diffraction are becoming widely accessible through easy-to-use software^14–16^, so that the barrier between a routine refinement—that only employs spherical atoms and hence neglects chemical bonding and physical properties—and an accurate refinement using the correct electron-density distribution in molecules and solids has become much lower. Hence, here we want to establish a *Quantum Crystallographic Protocol* that we recommend for use for every routine and unproblematic crystal structure determination such as when testing the quality and settings of local in-house diffractometers with standard samples. We want to put a hold to the waste of useful experimental data and the waste of useful chemical information if instead a simple quantum-crystallographic analysis protocol can be followed, and the results can be deposited in the Cambridge Structural Database (CSD)^17^ for the benefit of the scientific community.

**Scheme 1:**
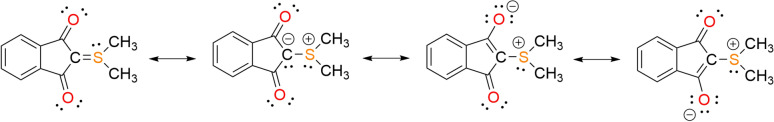
The molecular structure of 2-dimethylsulfuranylidene-l,3-indanedione (YLID) in the most relevant Lewis resonance forms. (Figure S1 represents the 3-D chemical structure with the atomic labels.)

Since 1969, 2-dimethylsulfuranylidene-l,3-indanedione (YLID) crystals (Scheme 1) have been shipped to users and employed as test crystals for adjusting and calibrating single-crystal diffractometers by the two market leaders Bruker and Rigaku as well as the earlier Syntex, Nicolet and Siemens Analytical Instruments, Oxford Diffraction and Kuma^18,19^. The chiral, orthorhombic polymorph in space group P2_1_2_1_2_1_ is used. Because of its use as standard, tens of thousands of YLID crystal structures must have been determined under various conditions and setups everywhere around the world over the past half a century. It is undoubtedly the world’s most common crystal structure. However, already Webster stated in 1998 that “few duplicated cell dimensions are in the literature even though the compound must have been examined innumerable times”^19^. In the most complete work on YLID to date, also comprising the history of the YLID compound, Guzei et al. analyse lattice constants for a total of 223 measurements, but they rely on private communications with subscribers of the Bruker users group and employees of the Bruker-AXS company^18^. In fact, there are only 11 depositions of the three-dimensional structure of YLID in the CSD and only 8 structure factor sets are publicly available for re-refinement and method validation (see Table S1 in the Supplementary Information)^18,20–26^. Presently, new quantum crystallographic techniques are mostly validated against data of oxalic acid for which the record number of 14 independent structure factor data sets are publicly available^27–29^. What if instead of these low numbers we could base method validation on tens of thousands of repeated measurements if all existing YLID structures had been deposited?

The aim of this paper is two-fold: We want to motivate that every YLID measurement will be followed by a quick but accurate quantum crystallographic refinement which is called *Quantum Crystallographic Protocol* (QCP), and we want to motivate that the results will always be deposited in the CSD together with the corresponding structure factors in the future as a community effort. These two points hold for every other structure as well, but YLID as *the* test crystal is a special case. The quality of the measured data obtained from the respective tested setup can much more reliably be ascertained by the total electron-density distribution and the refined hydrogen atom parameters than just the internal and refinement R-values as usually done^30^. We will at first establish these quality criteria to show how quickly they can be accessed nowadays and how they can be interpreted. Subsequently, it is necessary to analyse in some detail how far these criteria can be generalized with respect to the following points: i) crystal habit, ii) temperature, iii) wavelength and resolution. We note that very recently the first test of hardware using the total electron-density distribution of YLID in the sense that we wish to establish here has been published, however using a different quantum crystallographic method^26^.

1. The YLID test crystal is delivered and used as a spherical crystal ground in a ball mill. As an advantage, this facilitates absorption and other corrections during the data reduction procedure; but as a disadvantage it introduces mechanical strain which may deteriorate the crystal quality. Therefore, here we compare measurements on five different spherical crystals with crystals of natural shape synthesized and produced for this study (see Figure S2 in the Supplementary Information).
2. The spherical test crystals should not be cooled to cryogenic temperatures as the mechanical strain, or the adhesive material used, will possibly lead to cracks. There is no phase transition upon cooling that could cause this damage, also investigated in detail by Guzei et al.^18^, but we and others have empirically found this behaviour before^18^, which does not occur in the crystals of natural shape. Therefore, the structural and electron-density comparisons suggested in this paper should be carried out at room temperature as long as it involves the YLID because everybody owns these spherically ground crystals. However, the deconvolution of atomic thermal motions and valence electron density is largely facilitated at ultra-low temperatures^31^, but with good crystal quality and known reference values validation at room temperature might still be useful. Therefore, we compare results for the crystals of natural shape between 292, 150 and 100 K.
3. The most widely used in-house X-ray tubes contain Cu-, Mo- and Ag-sources (λ(Cu) = 1.54184 Å, λ(Mo) = 0.71073 Å, λ(Ag) = 0.56087 Å, K_α_ radiation each). YLID crystals are normally used to test diffractometers with all these sources, so we employ those three in this study as well. In addition, three measurements at beamline BL02B1 of the synchrotron SPring-8, Hyogo, Japan, at a wavelength of 0.2483 Å (50 keV) were carried out. Especially with Cu radiation, the attainable resolution limit (d_max_ ~ 0.78 Å) is significantly below the recommended limit of d = 0.5 Å for experimental electron-density studies^32^. However, this limit is arbitrary and traditionally justified with the high number of parameters that are refined in a multipole model (MM) of experimental charge-density determination^33^. More modern approaches within quantum crystallography, e.g., Hirshfeld Atom Refinement (HAR)^34,35^ and X-ray constrained wavefunction (XCW) fitting^11,36^, are not as clearly dependent on resolution; e.g. with HAR, hydrogen atom parameters can be determined accurately and precisely at the Cu resolution limit^7^. Therefore, here we employ all three methods MM, HAR and XCW fitting in a resolution range from d = 0.417 to 0.807 Å.

## Materials and methods

### Synthesis and crystal growth

2-Dimethylsulfuranylidene-1,3-indanedione was prepared using a one-step synthesis in accordance with a previously reported procedure by Lácová and Šišková^37^. 1 g of 1,3-indanedione and 1.7 mL of dimethyl sulfoxide were dissolved in 3.5 mL of acetic anhydride. The reaction mixture was stirred at 80 °C for 8 h. Then, the reaction mixture was cooled to room temperature and 40 mL of ethyl ether were added. The obtained crude reddish precipitate was dissolved in acetonitrile and purified by silica column chromatography. Elution with acetonitrile removed reddish coloured impurities and the desired compound was isolated with acetone. After evaporation of solvent, the compound was recrystallized from acetone to give yellow crystals of the orthorhombic polymorph of YLID (0.94 g). Yield = 66%.

### Measurements

From the batch of the orthorhombic polymorph, we selected seven different uncut crystals with natural faces, and we used the five commercially obtained spherical test crystals that are in our possession. Table 1 summarizes the conditions under which they were measured. A total of 23 different measurements were conducted. All crystallographic and measurement details are summarized in the Supplementary Information Table S2. The Ag- and Cu-measurements were carried out on a Rigaku Synergy-S instrument with a microfocus dual source and HyPix-6000 hybrid pixel detector. The Mo-measurements were carried out on an older Oxford Diffraction SuperNova instrument with a microfocus source and an EOS CCD detector or a modern Rigaku Synergy-R instrument with a rotating anode source and HyPix-Arc-100 hybrid pixel detector. The measurements at 292 K were held constant at this temperature with a nitrogen gas-stream device, so it is not fluctuating room temperature. The synchrotron experiments were conducted at beamline BL02B1 of the synchrotron SPring-8, which is equipped with a Dectris PILATUS3 X 1M CdTe pixel detector, at a wavelength of 0.2483 Å (50 keV).

**Table 1:** Summary of X-ray data collection conditions and refinement results.

	Resolution (Å)/CCDC-no	Wavelength*	Temperature	Crystal/crystallographic chirality	HAR/XCW/MM	R-value	Residual density e/Å^3^ (max/min)
1	0.491/ 2309624	Ag	100 K	Natural shape 1 LS^§^	Yes/yes/yes	0.0118 0.0104 0.0110	0.110/− 0.076 0.089/− 0.072 0.090/− 0.082
2	0.805/ 2309650	Cu	100 K	Natural shape 1 LS	Yes/yes/no	0.0100 0.0076 N/A	0.078/− 0.053 0.052/− 0.044 N/A
3	0.541/ 2309729	Mo**	100 K	Natural shape 1 LS	Yes/yes/yes	0.0157 0.0141 0.0147	0.119/− 0.136 0.103/− 0.116 0.121/− 0.118
4	0.460/ 2310481	Mo^#^	100 K	Natural shape 4 LS	Yes/yes/no	0.0078 0.0072 N/A	0.099/− 0.065 0.101/− 0.059 N/A
5	0.513/ 2310470	Mo^#^	100 K	Natural shape 2 LS	Yes/yes/no	0.0100 0.0089 N/A	0.189/− 0.072 0.165/− 0.102 N/A
6	0.417/ 2310479	Synchrotron SPring-8	100 K	Natural shape 5 LS	Yes/yes/no	0.0088 0.0092 N/A	0.111/− 0.104 0.065/− 0.077 N/A
7	0.436/ 2310478	Synchrotron SPring-8	100 K	Natural shape 6 LS	Yes/yes/no	0.0107 0.0101 N/A	0.113/− 0.167 0.088/− 0.103 N/A
8	0.491/ 2310480	Synchrotron SPring-8	100 K	Natural shape 7 LS	Yes/yes/no	0.0157 0.0119 N/A	0.282/− 0.516 0.184/− 0.223 N/A
9	0.532/ 2309730	Ag	150 K	Natural shape 1 LS	Yes/yes/no	0.0110 0.0093 N/A	0.091/− 0.059 0.075/− 0.053 N/A
10	0.802/ 2309737	Cu	150 K	Natural shape 1 LS	Yes/yes/no	0.0070 0.0059 N/A	0.070/− 0.046 0.051/− 0.038 N/A
11	0.694/ 2309738	Ag	292 K	Natural shape 2 LS	Yes/yes/no	0.0141 0.0103 N/A	0.108/− 0.052 0.064/− 0.044 N/A
12	0.748/ CCDC- 2309739	Mo**	292 K	Natural shape 2 LS	Yes/yes/no	0.0133 0.0109 N/A	0.069/− 0.060 0.048/− 0.055 N/A
13	0.623/ 2310190	Ag	292 K	Test crystal 1 RS^§^	Yes/yes/no	0.0112 0.0096 N/A	0.089/− 0.044 0.094/− 0.090 N/A
14	0.620/ 2310191	Mo**	292 K	Test crystal 1 RS	Yes/yes/no	0.0125 0.0115 N/A	0.075/− 0.070 0.095/− 0.097 N/A
15	0.806/ 2310482	Cu	292 K	Test crystal 2 LS	Yes/yes/no	0.0110 0.0062 N/A	0.070/− 0.076 0.026/− 0.029 N/A
16	0.657/ 2310193	Mo**	292 K	Test crystal 2 LS	Yes/yes/yes	0.0164 0.0135 0.0141	0.087/− 0.073 0.058/− 0.058 0.066/− 0.066
17	0.628/ 2310192	Ag	292 K	Test crystal 2 LS	Yes/yes/yes	0.0178 0.0146 0.0143	0.145/− 0.091 0.076/− 0.069 0.072/− 0.066
18	0.807/ 2310197	Cu	292 K	Natural shape 2 LS	Yes/yes/no	0.0099 0.0088 N/A	0.048/− 0.053 0.055/− 0.073 N/A
19	0.789/ 2310198	Cu	292 K	Test crystal 1 RS	Yes/yes/no	0.0120 0.0097 N/A	0.081/− 0.050 0.105/− 0.035 N/A
20	0.807/ 2310199	Cu	292 K	Natural shape 3 LS	Yes/yes/no	0.0097 0.0083 N/A	0.059/− 0.057 0.046/− 0.059 N/A
21	0.787/ 2310195	Cu	292 K	Test crystal 3 LS	Yes/yes/no	0.0125 0.0094 N/A	0.097/− 0.095 0.059/− 0.044 N/A
22	0.800/ 2310196	Cu	292 K	Test crystal 4 LS	Yes/yes/no	0.0103 0.0099 N/A	0.059/− 0.072 0.061/− 0.067 N/A
23	0.784/ 2310194	Cu	292 K	Test crystal 5 RS	Yes/yes/no	0.0108 0.0093 N/A	0.077/− 0.053 0.057/− 0.047 N/A

*Wavelength: Ag = 0.56087 Å; Mo = 0.71073 Å; Cu = 1.54184 Å; Synchrotron SPring8 = 0.2483 Å.

^#^Synergy-R diffractometer.

**SuperNova diffractometer.

^§^LS = left-handed screw axis; RS = right-handed screw axis.

All structures were solved with ShelxT^38^ and initially refined with ShelxL^39^ (https://shelx.uni-goettingen.de) to obtain a set of starting coordinates and atomic displacement parameters for the quantum crystallographic refinements (see Table S3). To perform HAR and XCW fitting using Tonto, a merged hkl file of structure factor magnitudes |F| is needed (details on HAR and XCW in the next subsection). To this end, the Shelx LIST 6 command was applied during IAM refinement to obtain a merged hkl file which already is corrected for anomalous dispersion and extinction effects. To compare the Tonto results with HAR using NoSpherA2 and with multipole refinement using XD, the same merged hkl file was applied for those. See Table S2 for the numbers of merged and unmerged reflections as well as the redundancies of the measurements, which range from 10 to 40.

### Quantum crystallographic refinement techniques and software

In the broader definition of quantum crystallography^5,6^, all methods of applying non-spherical atomic electron densities to account for chemical bonding and non-bonding deformations in the refinement are included. Of these methods, here we use three different ones: HAR, XWR and MM, whereby details and results for MM are only discussed in the Supplementary Information—Part I. Comparison of models.

(i) *Hirshfeld Atom Refinement* (HAR)^7,34,35^, in which the non-spherical atomic electron densities are calculated from the quantum–mechanical electron densities of the molecules on-the-fly and repeatedly during the refinement cycles. Only positional parameters and atomic displacement parameters [anisotropic displacement parameters, ADPs] for all atoms including hydrogen atoms are refined; the electron density is not refined. For the recommended protocol that we discuss in section 1) of the Results and Discussion chapter, we used the software NoSpherA2^14^ (https://www.olexsys.org/olex2/docs/nosphera2), which combines the refinement engine olex2.refine^40^ (version 1.5, https://www.olexsys.org/olex2) and the quantum-chemical software Orca5.0^41^ (version 5.0, https://www.faccts.de/orca) within the graphical user interface Olex2^42^ (version 1.5, https://www.olexsys.org/olex2)^43^. The B3LYP functional^44,45^ and def2-TZVP basis set^46^ were chosen, and the procedure was applied to measurements nos. 4–8 and 18–23. All hydrogen atoms were refined freely and anisotropically. Gram–Charlier parameters describing anharmonic atomic motions were also refined for the sulfur atom in some data sets, depending on the observation of a shashlik-like residual density pattern (more information in the Supplementary Information, Figure S3 and corresponding text)^47^.

HARs for measurements 1–3 and 9–17 were additionally carried out using the Tonto^48^ (revision affdad5b, https://github.com/dylan-jayatilaka/tonto) and lamaGOET^15^ (https://github.com/lomalaspina/lamaGOET) software packages with the same level of theory as in NoSpherA2. In contrast to NoSpherA2 refinements, here self-consistent atomic Hirshfeld charges and point dipoles on all atoms of completed molecules that have at least one atom within an 8 Å radius of any atom in the asymmetric unit were used in the wavefunction calculations to simulate the electrostatic crystal field effect. Moreover, anharmonic parameters were also refined for sulfur atoms where applicable. The refinement was based on structure factor magnitudes |F| based on the merged datasets whose generation was described above.

The details of all HARs are collected in Table S4. The crystallographic information files (CIFs) along with the structure factor lists for all refinements based on HAR were submitted to the Cambridge Structural Database under deposition numbers CCDC-2309624, 2309650, 2309729-2309730, 2309737-2309739, 2310190-2310199, 2310470, 2310478-2310482. They can be obtained free of charge from https://www.ccdc.cam.ac.uk/structures.

(ii) *X-ray wavefunction refinement* (XWR)^49^, which is a combination of HAR and subsequent X-ray constrained wavefunction (XCW) fitting^11,36^ upon fixed coordinates and ADPs (including higher order anharmonic terms) from HAR. In XCW fitting, molecular orbital coefficients of a wavefunction are varied relative to the measured structure factors so that the quantum mechanical state for the energy of the system is minimized by considering a constraint/restraint concerning the desired compromise between the model and the experimental charge density. Both steps of the XWR procedure—HAR and XCW fitting—were done with the Tonto program; it is not available in NoSpherA2 or any other software than Tonto. XWR was performed for all measurements 1–23. It was done with the HF method and the def2-TZVP basis set without a simulated crystal field because the effects of both electron correlation and polarization in the crystal field are included into the wavefunction via the experimental structure factors^50^. The λ steps—λ being the manually adjustable parameter that disturbs the theoretical wavefunction^29,51^—applied during the fit were 0.01, 0.05, 0.1, or 0.5, depending on the dataset. The final XWR result is based on the fitted wavefunction from the last λ step for which the refinement was convergent. More details can be found in Table S5 in the Supplementary Information.

### Theoretical calculations

The experimental geometry of dataset 1 was chosen as a starting point to perform a DFT geometry optimization for a single YLID molecule in the isolated state at the B3LYP/def2-TZVP level of theory. The software Gaussian 09^52^ (revision D, https://gaussian.com) was used and the resulting wavefunction was analysed using the AIMALL software (14_11_23, https://aim.tkgristmill.com)^53^.

This paper contains an extensive Supplementary Information document (including parts I. to III. with Figures S1 to S25 and Tables S1 to S25) to support the findings and conclusions that are discussed and summarized in the next sections.

## Results and discussion

### A quantum crystallographic protocol (QCP) and related quality criteria

For the reliable and meaningful evaluation of the data quality that an X-ray diffractometer produces in a given setup, only a comparison of intensities (R_int_/R_merge_) and a check of the R-value after a routine ShelxL-type refinement (IAM = Independent Atom Model) is not enough^26^. Therefore, here we suggest a *quantum crystallographic protocol* (QCP) based on room-temperature and low-resolution data sets of the spherical YLID test crystal that is fast and easy to do. It is based on the free and GUI-assisted NoSpherA2 software in Olex2^14,42^. Although it is here suggested in the context of YLID test measurements, it is valid in exactly the same way for any other measurement for which the data quality should be assessed for further scientific applications or determination of derived properties.

**Step 1**: Restrict data to a maximum resolution so that the included high-resolution data still contain valuable information and do not dilute the data set with noise. We suggest the following criteria for exclusion of high-order resolution shells: R_int_/R_merge_ > 15%, completeness < 100%, I/σ (intensity over standard uncertainty) < 3.0. This information can be accessed, e.g., via the Olex2 GUI under the tabs “Info” → “Reflection Statistics”.

**Step 2**: Standard IAM refinement in ShelxL or olex2.refine. Check whether all hydrogen atoms can be found from the Fourier difference map and whether their coordinates and isotropic displacement parameters can be refined freely. There should be no need for a riding model or any other restraints or constraints at this stage.

**Step 3**: Visually evaluate the residual electron-density map (accessible via the Olex2 GUI under the tabs “Tools” → “Maps”) and Q-peak positions. Figure 2a, c represent the residual-density distribution as it should look like for a good-quality measurement: the positive residual electron density (green, or brown balls) is systematically distributed in chemically meaningful positions, namely chemical bonds, and lone pair regions.

**Fig. 1:**
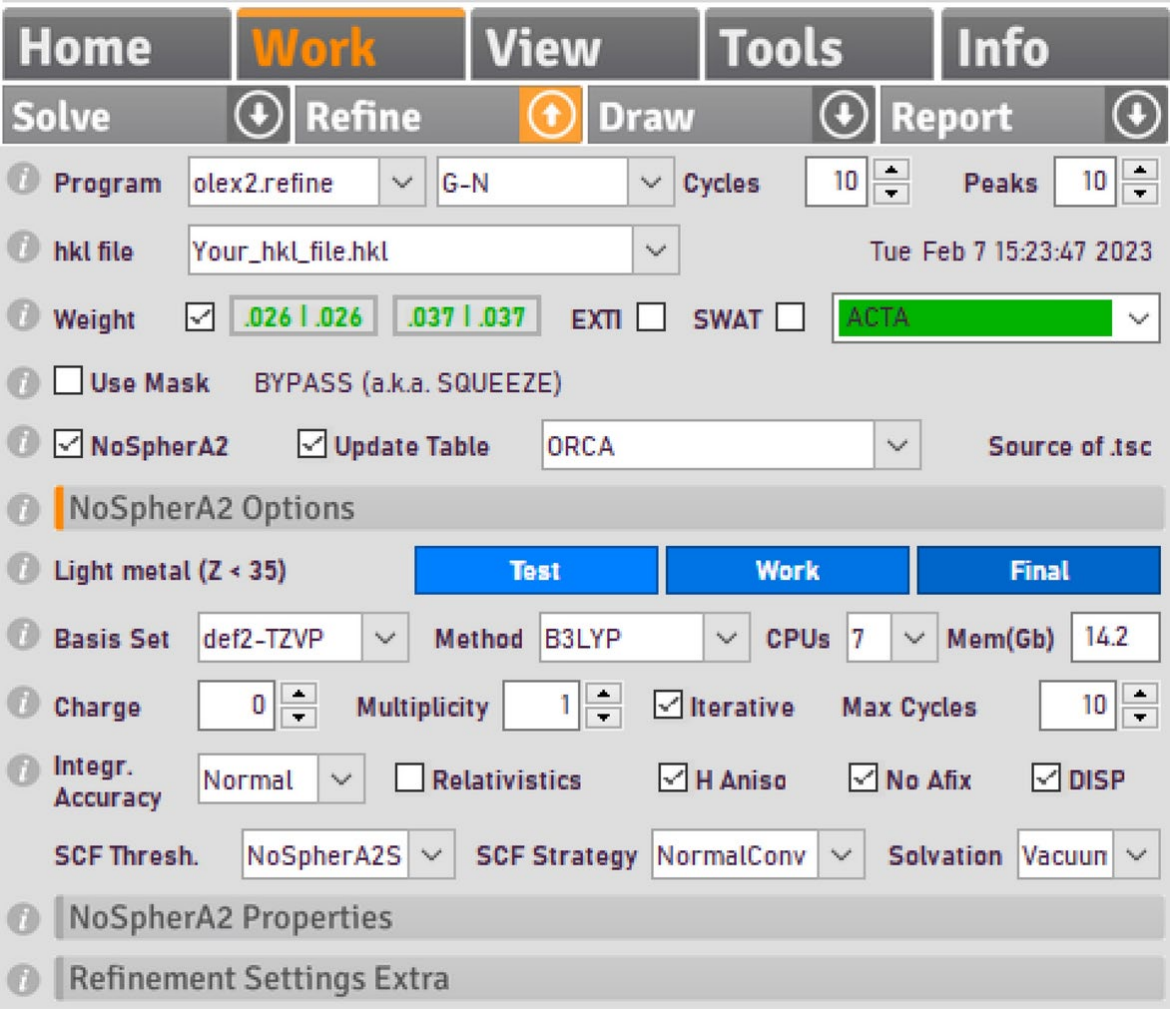
An example of NoSpherA2 settings to perform a HAR on a desktop computer.

**Step 4**: Perform a Hirshfeld Atom Refinement (HAR) using NoSpherA2^14,54^. To this end, one must use olex2.refine as a refinement engine. If olex2.refine is selected, then the checkbox for NoSpherA2 appears, and upon its selection various options are unlocked. See Fig. 1 for typical settings used for small molecules such as the YLID structure. Hydrogen atoms should be refined freely with anisotropic displacement parameters. The main steps in a HAR using NoSpherA2 are:

1. A molecular or cluster wavefunction of the system is computed to obtain the quantum–mechanical electron density of the input model. Typically, the software ORCA5.0^41^ is used for that step. It needs to be installed on the used computer by the user but is afterwards embedded in the automatic workflow of NoSpherA2.
2. NoSpherA2 uses Hirshfeld partitioning to cut the quantum–mechanical electron density into non-spherical atomic density functions, which are Fourier transformed to generate non-spherical atomic form factors. These are handed over to olex2.refine for further refinement steps in form of a tsc file (tabulated scattering factors)^14^.
3. The procedure needs to be repeated iteratively until the shift of any parameter over its standard uncertainty relative to the previous HAR cycle is lower than 0.01. The NoSpherA2 GUI therefore provides a checkbox to make the wavefunction calculation—partitioning—refinement process iterative.

**Fig. 2:**
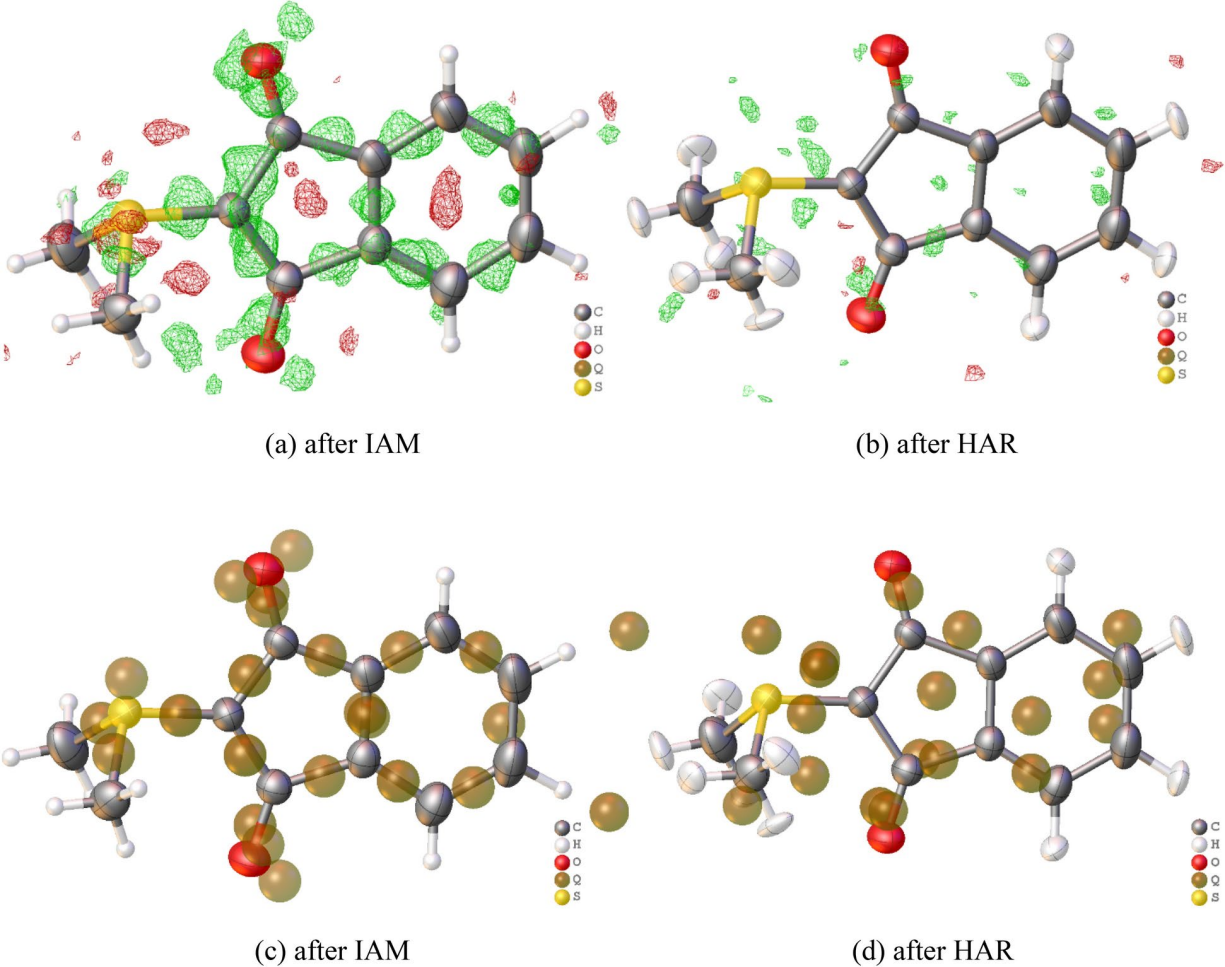
(**a**) Three-dimensional residual electron-density map (isovalue = 0.07 e/Å^3^, green = positive, red = negative) after IAM, (**b**) three-dimensional residual electron-density map (isovalue = 0.07 e/Å^3^, green = positive, red = negative) after HAR, (**c**) alternative representation of residual electron density as Q-peaks (local residual density maxima) after IAM (five highest peaks: Q1 = 0.270, Q2 = 0.260, Q3 = 0.220, Q4 = 0.220, Q5 = 0.220 e/Å^3^), (**d**) Q-peaks after HAR (five highest peaks: Q1 = 0.100, Q2 = 0.090, Q3 = 0.080, Q4 = 0.080,Q5 = 0.080 e/Å^3^). For dataset 16 (λ = Mo, r.t., d_max_ = 0.629 Å; corresponding representations for dataset 15 at λ = Cu, r.t., d_max_ = 0.806 Å are given in the Supplementary Information, Figure S4). Pictures generated with Olex2.

**Step 5**: Check the residual density map after HAR. Minimum and maximum residual density values should now be of the same low magnitude, and the Q-peaks should be dispersed randomly (see Fig. 2b, d, and the Q-peak values in the caption). This should be accompanied by a significant drop in the R-value. For datasets 16 and 15 that were used in Fig. 2, the values are: 16—IAM 2.80%, HAR 1.64%; 15—IAM 2.10%, HAR 1.10%.

**Step 6**: Check how the hydrogen atoms have been refined. Do the X–H bond lengths correspond to average values from neutron-diffraction measurements within the uncertainties? For the example in Fig. 2, the average C_phenyl_-H/C_methyl_-H distances are 1.090(12)/ 1.059(13) Å, whereas the average values from neutron diffraction of reference compounds are 1.083(17)/1.077(26) Å^55^. Furthermore, are the hydrogen atom ADPs physically meaningful by visual inspection, as shown in Fig. 2b, d? If they are skewed, oblique or even non-positive definite (NPD), isotropic displacement parameters or restraints can be used, which was recently made available in olex2.refine for hydrogen atoms^56^. However, skewed, oblique or NPD ADPs do not influence the X–H bond distances^7,56,57^.

**Step 7**: Deposition of CIFs with structure factors after HAR with the CSD.

With this paper, we want to motivate that YLID test measurements are not discarded but deposited with their structure-factor files after they have been subjected to the above procedure. It would be a step-change for crystallographic method development if real statistics were accessible based on hundreds of repeated experiments. This is only realistic with the YLID structure, the world’s most common crystal structure, and only requires a minor effort by everybody in the science community who is in possession of or uses an X-ray diffractometer.

We suggest this QCP to be used for general practice whenever the data quality is “good enough”. We recognize that this statement is vague and “good quality” very difficult to define. Therefore, below we summarize some of the most important criteria in the form of a checklist, even if some points have been discussed above already. This list is not exhaustive, and for the purpose of an experimental electron-density determination to analyse chemical bonding it is not sufficient (more statistical analysis of the experimental structure factor list would have to be undertaken, e.g. according to Refs.^58–60^, see also Fig. S6 in the Supplementary Information). However, the below list does include checks for overfitting. This is important because we promote the refinement of hydrogen atom positions and, ideally, ADPs within the QCP which increases the number of refinable parameters significantly.

After the measurement:

- No sign of twinning or disorder.
- Low internal R value (R_int_) in all resolution shells (roughly below 5% overall).
- High average ratio of intensity over standard uncertainty (above 10), and above 3 for the highest resolution shell.
- Ideally 100% completeness or close to it; no high-intensity low-order reflections lost during the measurement.
- Suitable redundancy, minimum 5, ideally around 20 or higher.

After IAM refinement:

- Low R-value statistics (R1,wR2); R1 roughly below 5% and approximately the same value as R_int_.
- Highest residual electron density positions must be on the bonds and lone pairs. The corresponding values do not need to be low; quite the contrary, they should be significant.
- Low min/max values of residual electron density in random places; for organic compounds roughly below 0.2/0.3 eÅ^−3^.
- All hydrogen atom positions found from the Fourier difference map and freely refined, not using a riding model.

After HAR:

- Significant drop of R value, which is not due to overfitting, see the next four points.
- Significant drop of residual density min/max values, especially maximum values, leading to an even distribution of positive and negative residual electron density; min/max values should now have approximately the same magnitude.
- Positions of the highest residual electron-density peaks are now randomly distributed, signaling noise and no systematic effects [hidden disorder or anharmonic motions might now become visible].
- X–H distances are similar to distances obtained with neutron diffraction within 3 combined standard uncertainties; for organic compounds reference values are in Ref.^55^.
- Hydrogen atom ADPs are not NPD, however, use of restraints to optimize their shape and direction is sometimes still necessary and not a concern^56^.

Although not being quality criteria of a data set, higher resolution and lower temperature are always preferable to obtain more accurate results.

A quantum-crystallographic protocol using HAR and NoSpherA2, too, but from a slightly different viewpoint, was published by Hill & Boeré while this paper was under revision^61^. We recommend that the reader compares those two independently derived QCPs to find the workflow that best suits their needs and habits.

### Reproducibility and dependence of the results on crystal habit, radiation source, and temperature

In the Supplementary Information, tables and figures summarizing refinement statistics, refinement models and their geometries, residual and deformation density plots, and bond topological and atomic properties according to Bader’s Quantum Theory of Atoms in Molecules (QTAIM)^62^ are listed. QTAIM has been applied to the experimental electron-density distributions to obtain sets of descriptors related to chemical bonding and atomic properties that can be compared in a statistical manner. It was used for experimental studies of sulfur ylides before, but rarely^26,63,64^. A detailed comparison between the performance of IAM, HAR, XWR, and MM on the basis of QTAIM and the electron-density distribution is given in the Supplementary Information, part I, including Figures A and B. From that, we conclude that the HAR model (for the geometry) and the XWR model (for the electron density) are significantly more accurate than the IAM and the MM models. So in the following, we only report HAR and XWR results.

**Fig. 3:**
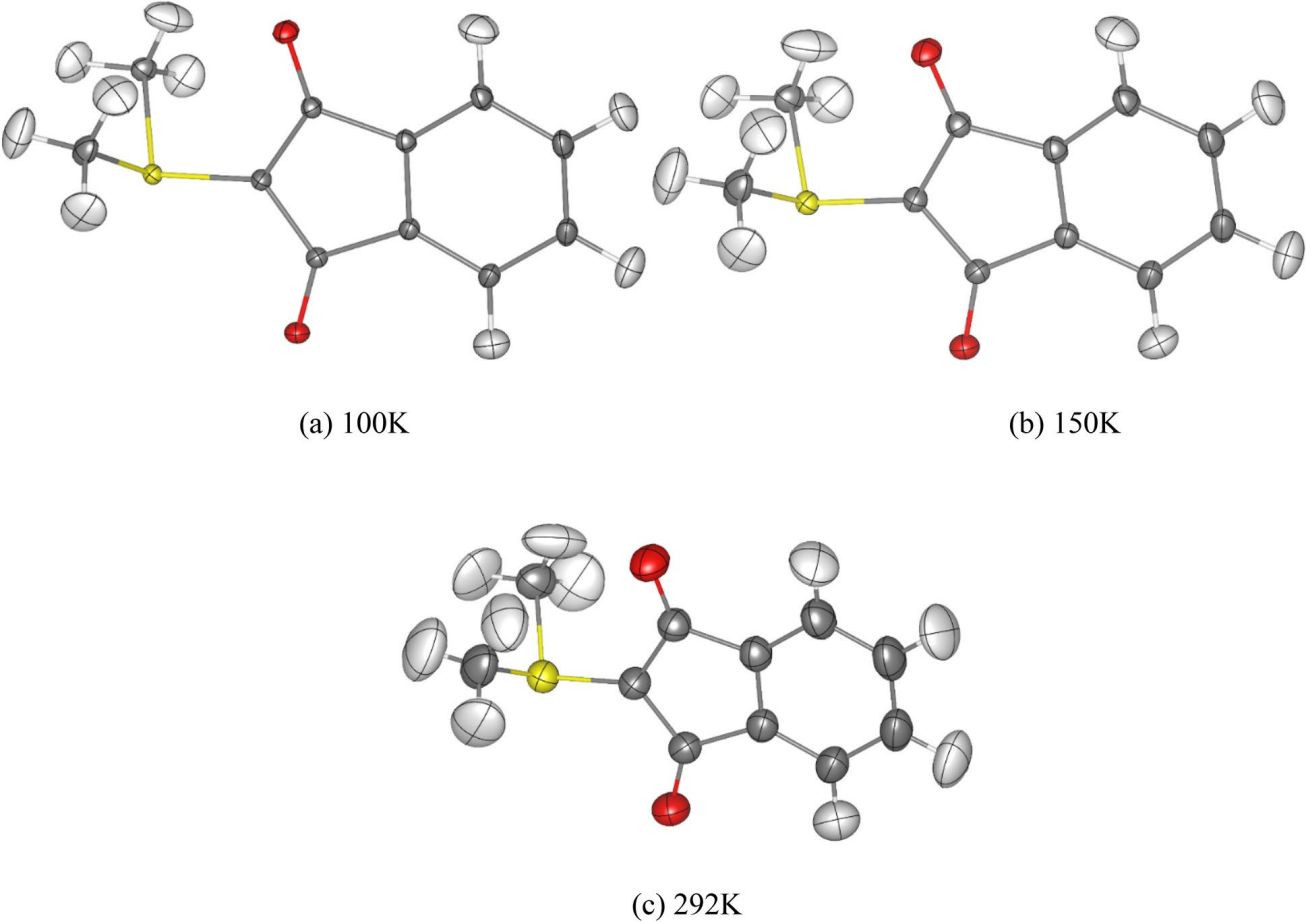
Molecular structures of YLID from HARs of the crystal structures measured with Ag wavelength at (**a**) 100 K (dataset 1) (**b**) 150 K (dataset 9) (**c**) 292 K (dataset 11). All anisotropic displacement parameters (ADPs) represented at a 50% probability level.

**Fig. 4:**
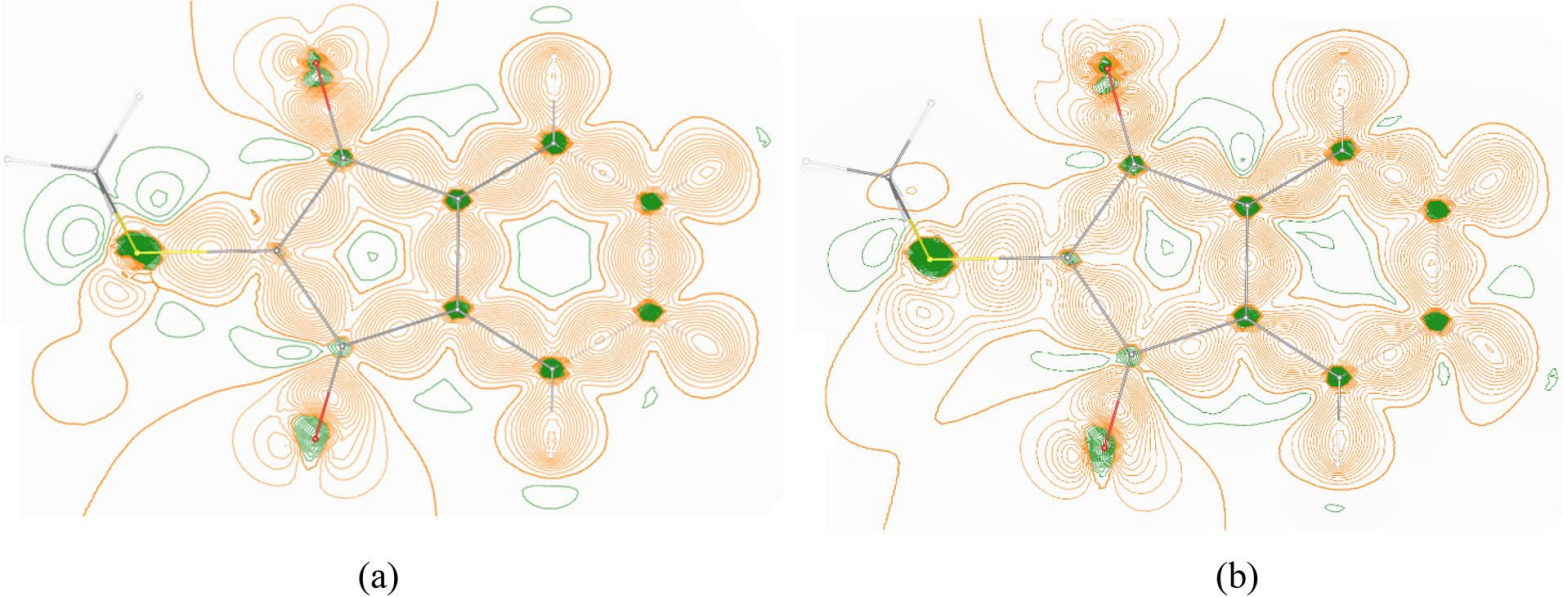
Deformation density maps for (**a**) dataset 1 (100 K) as well as (**b**) dataset 16 (292 K) in the XWR model. Isocontour value = 0.01 eÅ^−3^, green = negative, orange = positive. Pictures generated with VESTA^66^.

Before comparing numerical values, we inspect the temperature effect visually in terms of ADPs of the HAR-refined structures (Fig. 3) and deformation density maps after XWR (Fig. 4). The ADPs become larger with increasing temperature as expected, and for all hydrogen atoms all ADPs could be refined freely at all temperatures. The sizes and directions of vibrations of the hydrogen atom ADPs look physically reasonable for all temperatures; in fact the same is true for all 23 measurements, see Figure S7. This is a noteworthy result since until recently it has been doubted that unrestricted refinement of hydrogen atom ADPs from X-ray data would even be possible^65^. Here, we show that it is only a matter of crystal quality; it should generally be possible for every YLID test measurement (compare the QCP discussed in subsection 1 of the Results and Discussions). The high degree of similarity of the deformation density maps at 100 K and 292 K (Fig. 4) further confirms that evaluations of measurements with the spherical YLID test crystal are possible and meaningful that are normally done at room temperature.

For a statistical comparison, we first establish overall reproducibility limits (${\-{\sigma }}_{rep},$^67^) of derived properties by averaging over all 23 datasets in the HAR model (for geometry, Table S7) and the XWR model (for electron-density results, Tables S13 and S15). The average sample standard deviations (SDs) across all HAR-refined C-H bonds are ${\-{\sigma }}_{rep}$= 0.008 Å in aromatic rings and ${\-{\sigma }}_{rep}$= 0.014 Å in methyl groups. This result reflects that methyl groups are less rigid than aromatic rings, which emerges in the results relative to the temperature effect below clearly again. These reproducibility limits are related to the precision of the measurements, whereas the comparison to reference values is related to relative accuracy. Experimental reference values from neutron diffraction are taken from Allen and Bruno^55^: C-H(aromatic) = 1.083(17) Å and C-H(methyl) = 1.077(26) Å. Theoretical reference values are derived from our own geometry optimization: C-H(aromatic) = 1.082 Å and C-H(methyl) = 1.088 Å. The average HAR-refined values are C-H(aromatic) = 1.087(2) Å and C-H(methyl) = 1.075(6) Å. This leads to a very small root mean square deviation (RMSD) relative to neutron diffraction (0.006 Å) and one twice as large relative to theory (0.012 Å).

Reproducibility limits ${\-{\sigma }}_{rep}$ have been established before for atomic and bond-topological QTAIM parameters in the framework of the multipole model, across 13 repeated measurements of oxalic acid^27^, and across twice to four times repeated measurements of six different small peptides^68^. The established values are: 0.07 eÅ^−3^ for the electron density at the bond critical point (BCP), 3.3 eÅ^−5^ for the Laplacian of the electron density at the BCP, 0.12 e for the atomic charge Q_001_, and 0.4 Å^3^ for the atomic volume V_001_. For 23 repeated measurements of YLID in the XWR model (this study), the corresponding values are: 0.08 eÅ^−3^, 2.0 eÅ^−5^, 0.07 e, and 0.4 Å^3^. It is noteworthy that these sets of reproducibility indices are very similar despite the different approaches and models, so they reflect the limits of measurement setups and integration/ data reduction software for experimental electron-density determination. In terms of accuracy, here the reference values only come from theoretical calculations. Remarkably, the RMSDs relative to theory are very similar to the reproducibility limits: 0.09 eÅ^−3^, 1.2 eÅ^−5^, 0.05 e, and 0.5 Å^3^. This means that these orders of magnitudes for the electron-density descriptors can be used to judge whether the investigated crystal habit, wavelength, and temperature effects are significant or not.

For this purpose, we have divided the total of 23 datasets into different subsets that represent one of the parameters each, and compare their performance relative to each other by using sample standard deviations (SDs) upon averaging across the subsets and root mean square deviations (RSMDs) with respect to reference values from neutron diffraction or theoretical geometry optimization. All averaged numbers are discussed in detail in the Supplementary Information document, part II. For the temperature effect, differences between the methyl and the aromatic H atoms were found in both geometric and QTAIM properties that are caused by the higher dynamic flexibility of the methyl groups. Otherwise, it can be concluded from the statistical analysis that experimental electron-density evaluation at room temperature is possible nearly with the same precision and accuracy as at low temperature by using the XWR model. Compare also Fig. 4a with b for a visual confirmation of this result. Corresponding SD and RMSD values for the test crystals only very slightly exceed those for the entire set of 23 datasets established above, which renders experimental electron-density investigations with the test crystals possible in the XWR model. For the wavelength effect—which is simultaneously a resolution effect—no significant difference was found in geometry or electron-density properties when the data were grouped in four subsets containing the synchrotron, Cu, Mo, and Ag measurements. This, in turn, means that XWR modelling of low-resolution Cu data is possible and meaningful.

## Conclusions

Our primary objective is to introduce and establish a user-friendly *Quantum Crystallographic Protocol* (QCP) that we recommend for extending routine crystal structure refinement which is based on the approximation of spherical non-interacting atoms. The method Hirshfeld Atom Refinement (HAR) within the NoSpherA2-Olex2 software proves to be ideal for the QCP. This protocol also includes criteria based on residual electron density and hydrogen atom treatment to assess whether the quality of the measurement is sufficient. For a deeper analysis beyond the generally applicable QCP, we recommend X-ray wavefunction refinement (XWR) to analyse chemical bonding and electronic properties. This way, we demonstrate that data of sufficient quality are not restricted by temperature, wavelength, resolution or crystal shape.

The YLID test crystal must have been measured and its crystal structure refined innumerable times around the world since 1969. Despite this fact, only 8 structure factor sets are publicly available. We encourage deposition of every test measurement to increase this number to a few thousand within a short period of time. For the purpose of the analyses in this paper, we have re-measured YLID 23 times under different conditions, from room-temperature Cu-K_α_ to low-temperature synchrotron measurements. Based on such measurements, and based on quantum crystallographic HAR and XWR treatments, we could statistically analyse refined geometrical and electron-density parameters as reference values for future method development. The overall reproducibility limits for HAR-refined C-H bonds are ${\-{\sigma }}_{rep}$= 0.008 Å in aromatic rings and ${\-{\sigma }}_{rep}$= 0.014 Å in methyl groups. Averaged over all bond and atom types, for the electron density at the bond critical point, the Laplacian at the bond critical point, the atomic charge and the atomic volume, the corresponding values are ${\-{\sigma }}_{rep}$= 0.08 eÅ^−3^, 2.0 eÅ^−5^, 0.07 e, and 0.4 Å^3^.

## Supplementary Information

Supplementary Information 1.

Supplementary Information 2.

## Data Availability

The authors declare that the data supporting the findings of this study are available within the paper and its supplementary information file. Moreover, the crystallographic information files including lists of measured structure factors are available from the Cambridge Structural Database under deposition numbers CCDC-2309624, 2309650, 2309729-2309730, 2309737-2309739, 2310190-2310199, 2310470, 2310478-2310482. They can be obtained free of charge from https://www.ccdc.cam.ac.uk/structures.
