# Supplementary material for: A quantum crystallographic protocol for general use
Source: Sci Rep. 2025 Apr 19;15:13584. doi: 10.1038/s41598-025-96400-0 (PMC12009286; doi:10.1038/s41598-025-96400-0)

# checkCIF/PLATON report

Structure factors have been supplied for datablock(s) YLID_melt_Sy-R

THIS REPORT IS FOR GUIDANCE ONLY. IF USED AS PART OF A REVIEW PROCEDURE FOR PUBLICATION, IT SHOULD NOT REPLACE THE EXPERTISE OF AN EXPERIENCED CRYSTALLOGRAPHIC REFEREE.

No syntax errors found. CIF dictionary Interpreting this report

## Datablock: YLID_melt_Sy-R

---

```
Bond precision:   C-C = 0.0003 A           Wavelength=0.71073

Cell:             a=5.85874(3)     b=8.94002(5)     c=18.34043(10)
                  alpha=90         beta=90          gamma=90
Temperature:      100 K

                Calculated                    Reported
Volume          960.621(9)                    960.621(9)
Space group     P 21 21 21                    P 21 21 21
Hall group      P 2ac 2ab                     P 2ac 2ab
Moiety formula  C11 H10 O2 S                  C11 H10 O2 S
Sum formula     C11 H10 O2 S                  C11 H10 O2 S
Mr              206.25                        206.27
Dx,g cm-3       1.426                         1.426
Z               4                             4
Mu (mm-1)       0.304                         0.304
F000            432.0                         432.7
F000'           432.69
h,k,lmax        11,17,35                      11,17,35
Nref            7461[ 4198]                   7456
Tmin,Tmax       0.935,0.969                   0.826,1.000
Tmin'           0.913

Correction method= # Reported T Limits: Tmin=0.826 Tmax=1.000
AbsCorr = MULTI-SCAN

Data completeness= 1.78/1.00       Theta(max)= 43.840

R(reflections)= 0.0100( 7337)                      wR2(reflections)=
                                                   0.0230( 7456)
S = 1.436                  Npar= 242
```

The following ALERTS were generated. Each ALERT has the format
**test-name_ALERT_alert-type_alert-level.**
Click on the hyperlinks for more details of the test.

## Alert level C

```
PLAT911_ALERT_3_C Missing FCF Refl Between Thmin & STh/L=    0.600          5 Report
```

## Alert level G

```
PLAT019_ALERT_1_G _diffrn_measured_fraction_theta_full/*_max < 1.0      0.995 Report
PLAT142_ALERT_4_G s.u. on b - Axis Small or Missing .............    0.00005 Ang.
PLAT143_ALERT_4_G s.u. on c - Axis Small or Missing .............    0.00010 Ang.
PLAT802_ALERT_4_G CIF Input Record(s) with more than 80 Characters          1 Info
PLAT872_ALERT_4_G ALERTS Related to Anharmonic Refine   Suppressed          ! Info
PLAT883_ALERT_1_G No Info/Value for _atom_sites_solution_primary .     Please Do !
PLAT910_ALERT_3_G Missing # of FCF Reflection(s) Below Theta(Min).          2 Note
PLAT933_ALERT_2_G Number of HKL-OMIT Records in Embedded .res File          5 Note
PLAT978_ALERT_2_G Number C-C Bonds with Positive Residual Density.         10 Info
PLAT979_ALERT_1_G NoSpherA2 Scattering Factors Used .............     Please Note
```

0 **ALERT level A** = Most likely a serious problem - resolve or explain
0 **ALERT level B** = A potentially serious problem, consider carefully
1 **ALERT level C** = Check. Ensure it is not caused by an omission or oversight
10 **ALERT level G** = General information/check it is not something unexpected

3 ALERT type 1 CIF construction/syntax error, inconsistent or missing data
2 ALERT type 2 Indicator that the structure model may be wrong or deficient
2 ALERT type 3 Indicator that the structure quality may be low
4 ALERT type 4 Improvement, methodology, query or suggestion
0 ALERT type 5 Informative message, check

It is advisable to attempt to resolve as many as possible of the alerts in all categories. Often the minor alerts point to easily fixed oversights, errors and omissions in your CIF or refinement strategy, so attention to these fine details can be worthwhile. In order to resolve some of the more serious problems it may be necessary to carry out additional measurements or structure refinements. However, the purpose of your study may justify the reported deviations and the more serious of these should normally be commented upon in the discussion or experimental section of a paper or in the "special_details" fields of the CIF. checkCIF was carefully designed to identify outliers and unusual parameters, but every test has its limitations and alerts that are not important in a particular case may appear. Conversely, the absence of alerts does not guarantee there are no aspects of the results needing attention. It is up to the individual to critically assess their own results and, if necessary, seek expert advice.

**Publication of your CIF in IUCr journals**

A basic structural check has been run on your CIF. These basic checks will be run on all CIFs submitted for publication in IUCr journals (*Acta Crystallographica*, *Journal of Applied Crystallography*, *Journal of Synchrotron Radiation*); however, if you intend to submit to *Acta Crystallographica Section C* or *E* or *IUCrData*, you should make sure that full publication checks are run on the final version of your CIF prior to submission.

**Publication of your CIF in other journals**

Please refer to the *Notes for Authors* of the relevant journal for any special instructions relating to CIF submission.

---

**PLATON version of 06/07/2023; check.def file version of 30/06/2023**

**Datablock YLID_melt_Sy-R** - ellipsoid plot

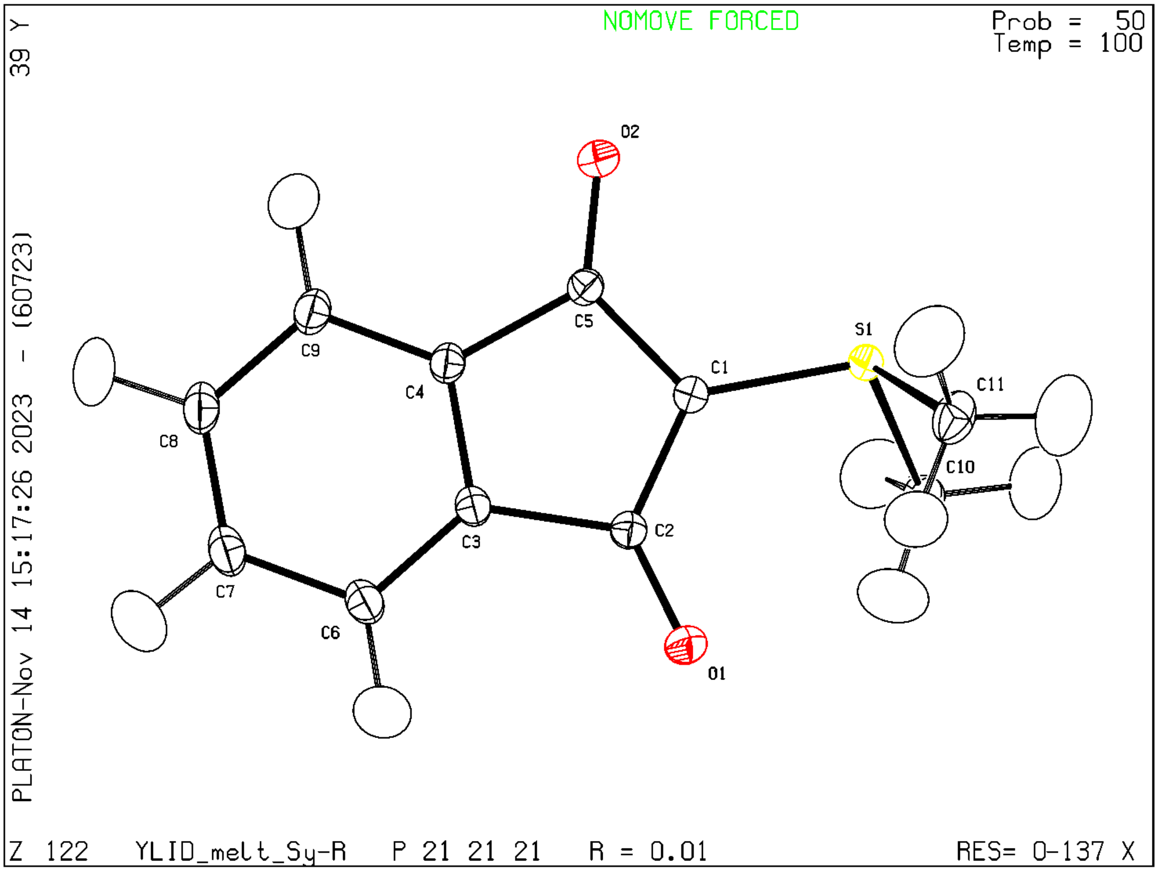

Supplement: Supplementary file 1 — Supplementary Information 1. [file 41598_2025_96400_MOESM1_ESM.zip › Dataset_5_HAR.pdf]
