# Supplementary material for: A quantum crystallographic protocol for general use
Source: Sci Rep. 2025 Apr 19;15:13584. doi: 10.1038/s41598-025-96400-0 (PMC12009286; doi:10.1038/s41598-025-96400-0)
Supplement: Supplementary file 2 — Supplementary Information 2. [file 41598_2025_96400_MOESM2_ESM.pdf]

Supplementary Information –

A quantum crystallographic protocol for general use

Yaser Balmohammadi,[a] Lorraine A. Malaspina,[a] Yuiga Nakamura,[b] Georgia Cametti,[c] Milosz Siczek,[d] Simon Grabowsky[a,*]

[a] *University of Bern, Department of Chemistry, Biochemistry and Pharmaceutical Sciences, Freiestrasse 3, 3012 Bern, Switzerland.*

[b] *Japan Synchrotron Radiation Research Institute (JASRI), Sayo-cho, Hyogo 679-5198, Japan.*

[c] *University of Bern, Institute of Geological Sciences, Baltzerstrasse 3, 3012 Bern, Switzerland.*

[d] *University of Wrocław, Faculty of Chemistry, F. Joliot-Curie 14, 50383 Wrocław, Poland.*

[*] *Correspondence e-mail: simon.grabowsky@unibe.ch*

I. Comparison of models

Multipole model. The multipolar model (MM) based on the Hansen–Coppens formalism[1] was used as implemented in the XD2016 software[2]. In the multipole model formalism, the electron density of a pseudoatom is described by spherical core and valence functions adjustable via the expansion-contraction coefficient κ as well as spherical harmonics describing the deformation valence density adjustable by multipole populations and higher-order expansion-contraction coefficients. In our study, multipole refinement was performed on datasets nos. 1, 3, 16, and 17, and was carried out for the same starting geometries as Hirshfeld Atom Refinement (HAR), and using the same set of structure factor magnitudes F. The initial multipole parameters were transferred from the databank UBDB2011 with the assistance of the LSDB program.[3]

For each MM refinement, first the scale factor was refined, then all atomic positions and anisotropic displacement parameters (ADPs) (isotropic for hydrogen atoms). Subsequently, hydrogen atom positions were fixed so that they obeyed average X-H distances from neutron diffraction experiments[4] and only their isotropic displacement parameters were still refined in the following. In the next phase, multipole parameters, subject to local symmetry constraints in agreement with the UBDB databank, were refined against all reflections by gradually releasing higher terms of the multipole expansion (up to the hexadecapolar level for non-hydrogen atoms and bond-directed multipoles up to the quadrupolar level for hydrogen atoms). Chemical constraints of the multipole parameters were applied on chemically equivalent carbon, oxygen, and hydrogen atoms. In the last phase, the simultaneous refinement of atomic positions, ADPs, and multiple parameters was done. The expansion-contraction parameter κ was refined freely for all

non-constrained atoms, but for H atoms optimized values $\kappa = 1.13$ and $\kappa' = 1.29$ were used.[5] The SHADE3 server[6] was used to estimate the values of hydrogen-atom ADPs that were fixed during the final cycles of the refinements. Details are given in Table S6.

Comparison between multipole model and XWR.

Four measurements (1, 3, 16, and 17) were chosen to be modelled in both the MM and the X-ray wavefunction refinement (XWR) model (see Materials and Methods for more details). This is one Ag and one Mo measurement each for 100K (natural-shape crystal #1) and 292K (spherical test crystal #2).

In HAR, as discussed in the *quantum crystallographic protocol* in the main document, the non-spherical electron-density distribution is calculated and used for the refinement of coordinates and ADPs, but not refined. Hence, HAR does not offer an experimental electron-density distribution. In contrast, during MM and XWR additional parameters related to the electron density are refined (multipole populations and expansion-contraction coefficients in MM; molecular orbital parameters in XWR). Figure A(a) shows that there is consequently a significant drop of the R-value from the IAM to the MM; and a smaller additional drop to the XWR model in three of four cases. This trend is also reflected in the min/max values of the residual electron density distribution (Figure A(b)). There is a significant drop from Independent Atom Model (IAM) to MM, and a smaller drop from MM to XWR. Values around 0.1 $eÅ^{-3}$ in the presence of a sulfur atom represent good data quality and a good model. Table 1 in the main document shows the residual density values for all datasets in all models. In some datasets, min/max values for the quantum crystallographic models are as low as $\pm$0.03-0.04 $eÅ^{-3}$. Importantly, in both MM and XWR the magnitudes of minimum and maximum are approximately the same, whereas in IAM the maximum value is significantly larger than the minimum values. This represents an unmodelled systematic effect, here the neglection of chemical bonding and lone-pair density. Representations of the residual electron-density distributions for all datasets and models are given in Figures S9 and S10. In terms of geometry, the C-H bond distances and H atom ADPs are most interesting to compare. However, since they are not refined but fixed in MM coming from average neutron diffraction results or from the SHADE server, respectively, in this subchapter only the electron-density models based on the final geometries are discussed further.

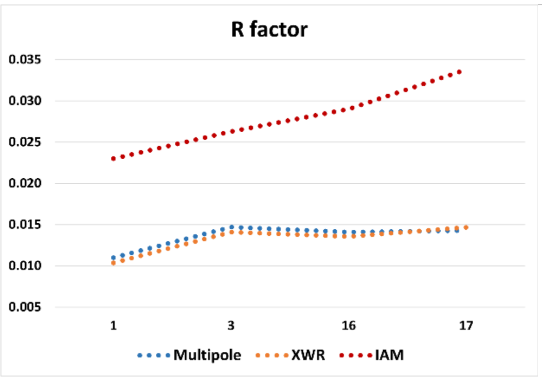

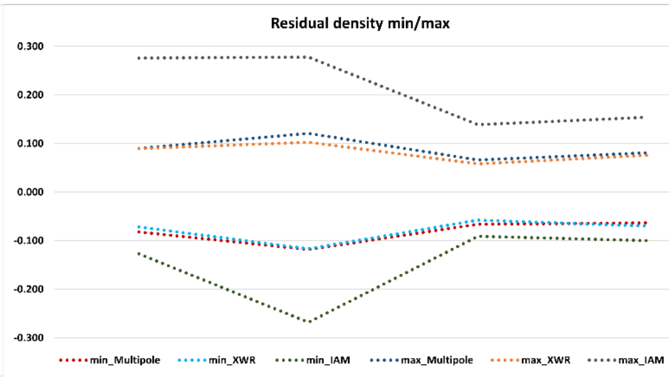

(a) (b)

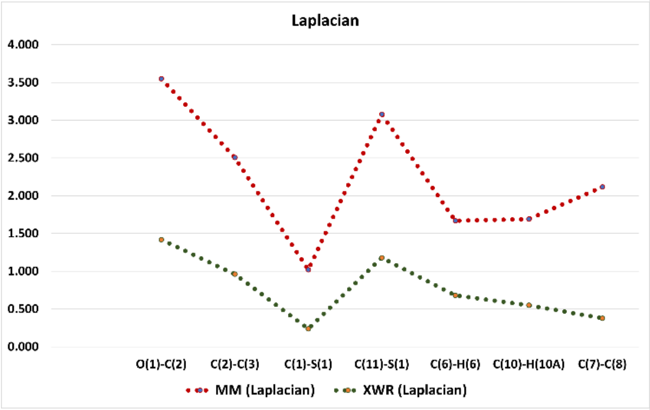

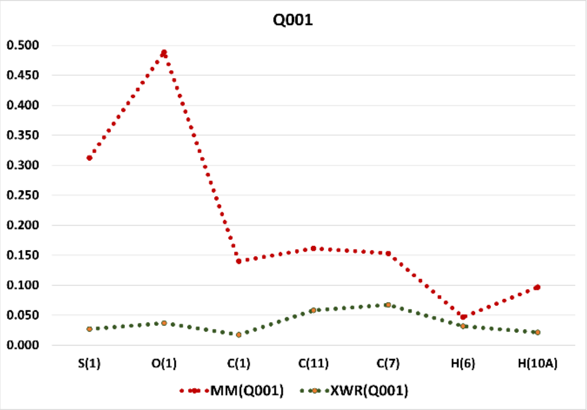

(c) (d)

Supplementary Figure A. Comparison between IAM, XWR and multipole refinement for datasets 1, 3, 16, 17 in terms of (a) R factor, (b) min/max residual density values (in eÅ^{-3}). (c) Sample standard deviation SD for the Laplacian of the electron density (in eÅ^{-5}) of selected bonds that are in different chemical environments. (Atomic labelling scheme in Figure S1.) The averaging was done across datasets 1, 3, 16, 17 per bond. Pertinent details in Table S12; averages across all 23 datasets for XWR in Table S13. (d) Sample standard deviation SD for the QTAIM atomic charge Q001 (in e; 001 means that the atomic basin was cropped at an isovalue of the electron density of 0.001 a.u.) of selected atoms that are in different chemical environments. The averaging was done across datasets 1, 3, 16, 17 per atom. Pertinent details in Table S14; averages across all 23 datasets for XWR in Table S15.

On the first glance, the deformation density maps shown in Figures Ba and Bb for 100K look quite similar between MM and XWR with bonds and lone pairs pronounced and shaped in a similar fashion. However, also here the XWR model shows a physically slightly more meaningful model in that electron density has been shifted from every core region (negative deformation density) into the valence region (positive deformation density). This distinction between core and valence is not as clear in MM. Similar comparisons and observations when comparing MM and XWR models have been made before.[7,8,9] These findings are especially important for the MM and XWR models at room temperature: whereas in MM, both lone-pair

and bonding densities are unsymmetrical and skewed (Figure Bc), they are nearly as symmetrical and meaningful in Figure Bd (XWR at 292K) as in Figure Bb (XWR at 100K). In addition, XWR models for all nine Cu datasets at limited resolution are shown in part III of this Supplementary Information file, however, they failed to converge for MM because of the low data/parameters ratios. This means that an electron-density analysis based on XWR, and therefore method development in modern quantum crystallography in general, can be carried out based on medium-resolution, room-temperature data if the quality is sufficient. In turn, this means that evaluations of measurements with the spherical YLID test crystal are possible and meaningful that are normally done at room temperature. This is the background for our request that YLID test measurements should not be discarded but deposited in the future for public use.

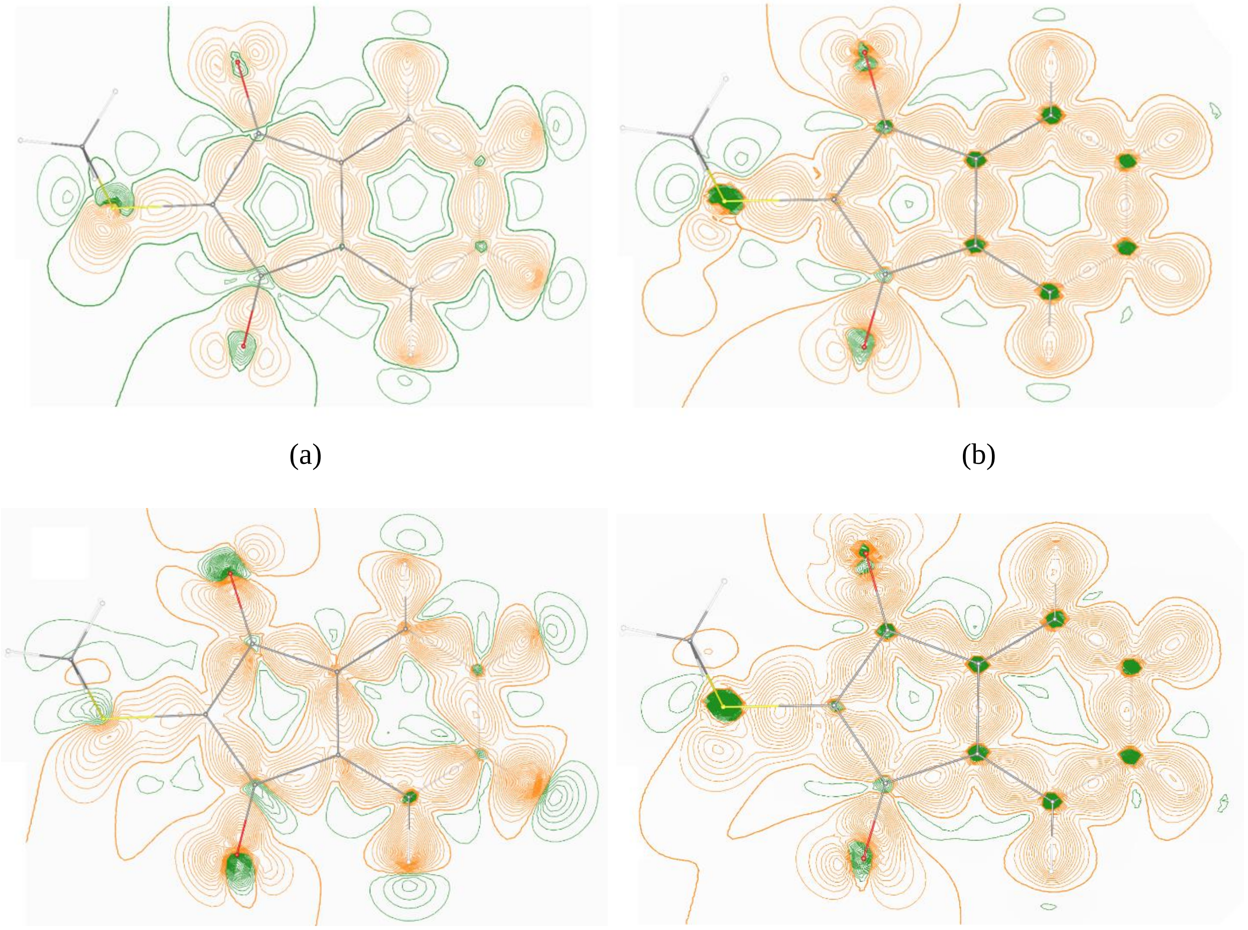

(a) (b) (c) (d)

Supplementary Figure B. Deformation density maps for dataset 1 (100K, natural-shape crystal, Ag-radiation, d_{max}=0.49Å) in (a) MM, and (b) XWR models as well as dataset 16 (292K, test crystal, Mo-radiation, d_{max}=0.63 Å) in (c) MM, and (d) XWR models. Isocontour value = 0.01 eÅ^{-3}, green = negative, orange = positive. Pictures generated with the software VESTA[10].

We further use the values of the electron density, its Laplacian, and the ellipticity for all bonds at their bond critical points (BCPs) for datasets 1, 3, 16, and 17 (MM, Table S8) and datasets 1-23 (XWR, Table S9), together with the atomic charges and volumes of all atoms (Tables S10 and S11). To compare XWR with MM for datasets 1, 3, 16, and 17, some chemical bonds and atoms that have different chemical environments in the YLID molecule have been selected to examine the QTAIM properties (Tables S12 and S14).

Figures Ac and Ad contain a representative comparison of the sample standard deviations (SDs) of bond topological and atomic properties when averaged across the various datasets in MM and XWR models (Figures S14 and S16 for all properties). These SDs are not only much lower after XWR treatment, but also more even. This means that the large SDs and outliers in MM are not related to the quality of the measurement but only to model insufficiencies that are not present in XWR. Or, in other words, XWR can handle datasets measured under different conditions more reliably to reproduce quantitative results. This can be understood as a measure of precision. Accuracy relative to an independent observation of the same properties is here calculated as the root mean square deviation (RMSD) between the MM/XWR results and those from theoretical calculations of the isolated molecule across all bonds and atomic properties per dataset. Tables and Figures S12 and S14 show the results. Here, the XWR model is significantly more accurate than the MM. RMSDs for electron density/ Laplacian at BCPs range from 0.43 to 0.13 e$Å^{-3}$/ 12.6 to 10.7 e$Å^{-5}$ for MM and 0.12 to 0.10 e$Å^{-3}$/ 2.3 to 1.1 e$Å^{-5}$ for XWR. For atomic properties charge Q_{001}/ volume V_{001}, RMSDs are 0.23 to 0.11 e/ 1.40 to 0.97 $Å^3$ for MM and 0.08 to 0.03 e/ 0.56 to 0.35 $Å^3$ for XWR.

II. Statistical analysis of the dependence of the refinement results on crystal habit, radiation source, and temperature

In this section, we will evaluate the effect of crystal habit, wavelength, and temperature on the geometrical and electron-density results. This is important since the YLID test crystal that everybody owns is spherically ground, will therefore normally only be measured at room temperature, and the resulting data will mostly be of low resolution. We have therefore divided the total of 23 datasets into different subsets that represent one of the parameters each, and compare their performance relative to each other by using sample standard deviations (SDs) upon averaging across the subsets and root mean square deviations (RSMDs) with respect to reference values from neutron diffraction or theoretical geometry optimization.

Effect of crystal habit.

To examine the effect of the crystal habit, two sub-sets were chosen: natural shape crystals (datasets 1-12,18, 20) and test crystals (datasets 13-17,19,21-23). Tables S16 to S18 and Figures S18 to S19 display the results for C-H bond lengths, atomic and bond-topological properties. It is noteworthy that the discussed

results are not controlled for the temperature and wavelength effects. Especially the temperature has a significant influence on the results because all measurements of the test crystals were done at 292K as cooling the test crystal will risk to crack it. The natural-shape crystals were measured at all temperatures 100K, 150K and 292K. We decided to include this effect already here because this will give the most realistic estimate of how reliably quantum-crystallographic refinements can be performed for the spherically-ground test crystals that are widespread around the world.

The average bond lengths with their sample standard deviations (in brackets) of C-H(aromatic) and C-H(methyl) are 1.087(2)/1.090(4) Å and 1.080(5)/1.079(11) Å for natural shape/ test crystals, respectively. Hence, in terms of precision, the absolute values are the same and thy are within the reproducibility limits ($\bar{\sigma}_{rep}$= 0.008 Å (aromatic) and 0.014 Å (methyl)) that were established in the main manuscript. However, in terms of accuracy, the RMSD values referenced against both neutron-diffraction data and the optimized geometry are smaller for the natural-shape than the test crystals: 0.005 Å (neutron) and 0.008 Å (opt) for the natural-shape crystals vs. 0.012 Å (neutron) and 0.019 Å (opt) for the test crystals. Hence, the accuracy is higher by more than a factor of two for the natural-shape crystals. This effect is clearly a temperature effect on the more dynamic methyl groups, though, as we will discuss below. Therefore, overall we conclude that the ground spherical crystal shape does not have a significantly negative influence on the refined geometries.

The average electron density at the BCP for the C2-O1 bond (2.71(5) and 2.69(4) eÅ^{-3}) and for the C1-S1 ylide bond (1.54(5) and 1.47(10) eÅ^{-3}) for the natural shape and the test crystals are the same within the sample standard deviations. A similar trend is observed for the rest of the bonds (Table S17), and both of the sub-sets have an appropriate precision mostly smaller than the reproducibility limit $\bar{\sigma}_{rep} =$ 0.08 eÅ^{-3}. The same holds for the Laplacian of the electron density, although here the SDs are significantly larger (Table S17). The trend also continues for the atomic charges and volumes that are the same within the SDs: Q_{001}(O1) = -1.28(5)/ -1.28(4) e and Q_{001} (S1) = 0.43(18)/ 0.49(16) e as well as V_{001}(O1) = 20.5(8)/ 20.5(5) Å^3 and V_{001}(S1) = 18.7(7)/ 18.4(6) Å^3 for natural shape/ test crystal sub-sets. Table S18 shows that the conclusions are the same for all other selected atoms, with reproducibility limits of $\bar{\sigma}_{rep}$ = 0.07 e, and 0.4 Å^3. In terms of accuracy for all bonds and atoms, the results of the natural shape crystals are closer to theoretical calculations (RMSD = 0.09 eÅ^{-3}, 1.2 eÅ^{-5}, 0.05 e, and 0.4 Å^3) than those for the test crystals (RMSD = 0.10 eÅ^{-3}, 1.6 eÅ^{-5}, 0.07 e, and 0.5 Å^3). This cannot as clearly be attributed to a temperature effect and may as well have to do with the increased strain in the ground crystals. However, the values for the test crystals only very slightly exceed those for the entire set of 23 datasets established above (0.09 eÅ^{-3}, 1.2 eÅ^{-5}, 0.05 e, and 0.5 Å^3), which still renders experimental electron-density determinations with the test crystals possible in the XWR model.

Wavelength effect.

Our 23 datasets are collected using synchrotron (λ = 0.2483 Å), Ag (λ = 0.56087 Å), Mo (λ = 0.71073 Å), and Cu (λ = 1.54184 Å) K_α radiations, and were accordingly sub-divided: subset 1 (synchrotron - datasets 6,7,8); subset 2 (Ag - datasets 1,9,11,13,17); subset 3 (Mo - datasets 3-5,12,14,16); subset 4 (Cu - datasets 2,10,15,18-23). Tables S19-S21 and Figures S20-S22 represent the average and statistical parameters of C-H bond lengths as well as QTAIM atomic and bond-topological properties. The wavelength dependency is also a resolution dependency as with Ag and synchrotron radiation much higher resolution can be reached than with Cu or Mo radiation. However, we employed the criterion for pruning higher-resolution reflections that we defined in the quantum-crystallographic protocol, step 1. Nevertheless, the maximum resolution range discussed here spans from d = 0.42 to 0.81Å.

The average bond lengths with their sample standard deviations (in brackets) of C-H(aromatic) and C-H(methyl) are 1.086(6), 1.087(2), 1.087(4), and 1.091(5) Å as well as 1.082(9), 1.076(7), 1.073(9), and 1.073(15) from synchrotron to copper radiation, respectively. The precision of determining the C-H(methyl) bond lengths is lower across all wavelengths compared to the C-H(aromatic) bond lengths, but even lower for the Cu radiation results, still very close to the reproducibility limit, though ($\bar{\sigma}_{rep}$= 0.008 Å (aromatic) and 0.014 Å (methyl)). The accuracy for Cu-radiation is also lower (RMSD = 0.012 Å (neutron) and 0.017 Å (opt)), and the accuracy for the synchrotron datasets highest (RMSD = 0.008 Å (neutron) and 0.009 Å (opt)), see Figure S20 for a graphical representation of the trend. The corresponding values for all 23 datasets were established above (RMSD = 0.006 Å (neutron) and 0.012 Å (opt)), showing that the HAR-refined C-H bond lengths from Cu radiation datasets at low resolution around d = 0.8 Å are still reasonable, and still significantly more meaningful than those from IAM refinement or the riding model (compare ref. 13, where a resolution limit for HAR of d = 0.8 Å was recommended).

The average electron density at the BCP for the C2-O1 bond (2.76(3), 2.68(2), 2.73(2), 2.68(6) eÅ^{-3}) and for the C1-S1 ylide bond (1.55(2), 1.55(2), 1.56(4), 1.45(9) eÅ^{-3}) from synchrotron to copper radiation, respectively, are similar (the same within three sample standard deviations), but with no clear trend, as the higher SDs for Cu radiation do not prevail for all bonds (Table S20, Figure S21). For the Laplacian of the same bonds, the trend is not clear either (Table S20, Figure S21). Concerning the accuracy relative to theoretical calculations, the RMSD for Cu radiation is as high as for Ag radiation for the electron density at the BCP (0.11 eÅ^{-3}), and the lowest in the series for the Laplacian (see Figure S21). For the atomic properties charge and volume (Table S21, Figure S22), the situation is similar, so that overall no wavelength, and, consequently, resolution effect on derived QTAIM results from XWR modelling can be detected. This, in turn, means that XWR modelling of low-resolution Cu data is possible and meaningful.

Temperature effect.

The similarity index (S) measures the agreement between the refined or estimated hydrogen atom ADPs with those from tabulated neutron data.[11] S=100(1-R), where R measures the overlap between the probability density functions (PDFs) related to the two ADPs (refined/neutron). If the two ADPs are identical, R equals 1, therefore, S expresses the percentage difference between the two ADPs. The smaller the value of S the higher is the agreement between the two ADPs. It should be noted that the similarity index does not provide information in which way the two ADPs differ. Table S25 collects the mean values of similarity indices for all 23 datasets calculated using the SHADE3 server. All similarity indices are in a reasonable range. There is a small correlation with temperature, exemplified by the values for the three refinements in Figure 3 (1.19 at 100K, 1.24 at 150K, and 2.04 at 292K), but other room-temperature test-crystal measurements with Cu radiation also have reasonable values (1.45 or 1.52 for datasets 21 and 17).

To evaluate the C-H bond lengths, atomic and bond-topological QTAIM properties, three subsets were defined for different temperatures: 100K (datasets 1-8); 150K (datasets 9-10); and 292K (datasets 11-23). Tables S22-S24 and Figures S23-S25 represent the data and statistical analyses related to these three subsets. The average values of bond lengths for C-H(aromatic) are the same 1.086(3), 1.089(6), 1.090(2) Å (100K to 292K), but for the C-H(methyl) bond lengths the room-temperature data sets lose precision and accuracy because of the increasing dynamic movement of the methyl groups: 1.083(3), 1.085(7), and 1.068(11) Å (100K to 292K) (Figure S23). The RMSD for the whole set of C-H bonds relative to neutron diffraction (0.006, 0.009, 0.011 Å) and relative to geometry optimization (0.005, 0.008, 0.018 Å) shows a trend with temperature, with the least accurate results for 292K. However, a similar conclusion as for the wavelength (resolution) dependency holds here: HAR-refined C-H bond lengths at room temperature are still reasonable (RMSDs = 0.006 Å (neutron) and 0.012 Å (opt) for all 23 datasets), especially as they are significantly more meaningful than those from IAM refinement or the riding model.

The average electron density at the BCP for the C2-O1 bond (2.74(3), 2.71(3), 2.68(5) eÅ^{-3}) and for the C1-S1 ylide bond (1.56(3), 1.55(1), 1.48(9) eÅ^{-3}) from 100K to 292K are the same within the standard deviation, but the SDs increase for the 292K data (for all bonds, see Figure S24). The SDs are nevertheless smaller or close to the reproducibility limit $\bar{\sigma}_{rep} = 0.08$ eÅ^{-3} (see also Table S23). The absolute values decrease for 292 K, but the RMSDs across all selected bonds relative to theory (0.08, 0.09, 0.10 eÅ^{-3}, 100K to 292K) do not increase significantly towards 292K. For the Laplacian of the electron density and the atomic charges, no trend relative to temperature can be observed (Figures S24 and S25). For the atomic volume, the trend is similar to the electron density at the BCP. Here, the absolute values and SDs increase with temperature, however, only for some atoms (Figure S25): 20.3(4), 20.5(1), 20.6(7) Å^3 for O1 as well as 18.5(6), 19.2(4), 18.6(7) Å^3 for S1 as examples between 100K and 292K. The RMSD values compared

to theoretical values for atomic charges Q_{001} (0.05, 0.07, 0.07 e from 100K to 292K) and volumes V_{001} (0.47, 0.52, 0.52 Å^3 from 100K to 292K) only show a minute loss of accuracy for the 292 K datasets relative to the 100K datasets. Overall, experimental electron-density evaluation at room temperature is possible nearly with the same precision and accuracy as at low temperature by using the XWR model.

III. Supplementary tables and figures

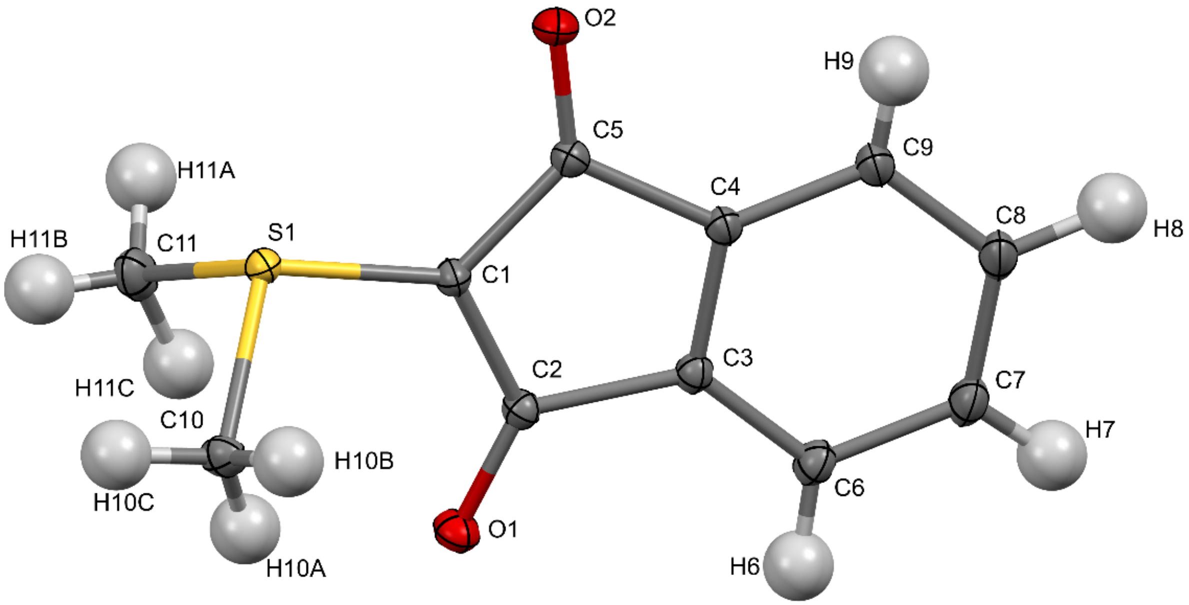

Figure S1. 3-D chemical structure of the YLID molecule with labeling scheme

Table S1. YLID crystal structures deposited in the Cambridge Structural Database; RS = right-handed screw axis. The reference no. refers to the main document.

Entry	CSD REFCODE$^{ref.}$	Year	Temperature (K)	Polymorph	Resolution (Å)	Wavelength (Å)	Crystallographic Chirality	hkl availability
1	MSULIN[20]	1971	295	Orthorhombic	0.77	1.54178 (Cu)	RS	No
2	MSULIN01[18]	2008	298	Orthorhombic	0.81	1.54178 (Cu)	RS	No
3	MSULIN02[18]	2008	100	Orthorhombic	0.81	1.54178 (Cu)	RS	No
4	MSULIN03[18]	2008	298	Monoclinic	0.81	1.54178 (Cu)	None	No
5	MSULIN04[18]	2008	100	Monoclinic	0.71	0.71073 (Mo)	None	No
6	MSULIN05[21]	2012	297	Orthorhombic	0.74	0.71073 (Mo)	RS	Yes

7	MSULIN06[22]	2015	295	Orthorhombic	0.71	0.71073 (Mo)	RS	Yes
8	MSULIN07[23]	2015	293	Orthorhombic	0.75	0.71073 (Mo)	RS	Yes
9	MSULIN08[23]	2015	293	Orthorhombic	0.75	0.71073 (Mo)	RS	Yes
10	MSULIN09[23]	2015	293	Orthorhombic	0.75	0.71073 (Mo)	RS	Yes
11	MSULIN10[24]	2016	100	Monoclinic	0.73	0.71073 (Mo)	None	No
12	MSULIN11[25]	2019	100	Orthorhombic	0.77	0.71073 (Mo)	RS	Yes
13	MSULIN12[25]	2019	100	Orthorhombic	0.77	0.71073 (Mo)	RS	Yes
14	MSULIN13[26]	2023	110	Orthorhombic	0.45	0.5134 (In)	RS	Yes

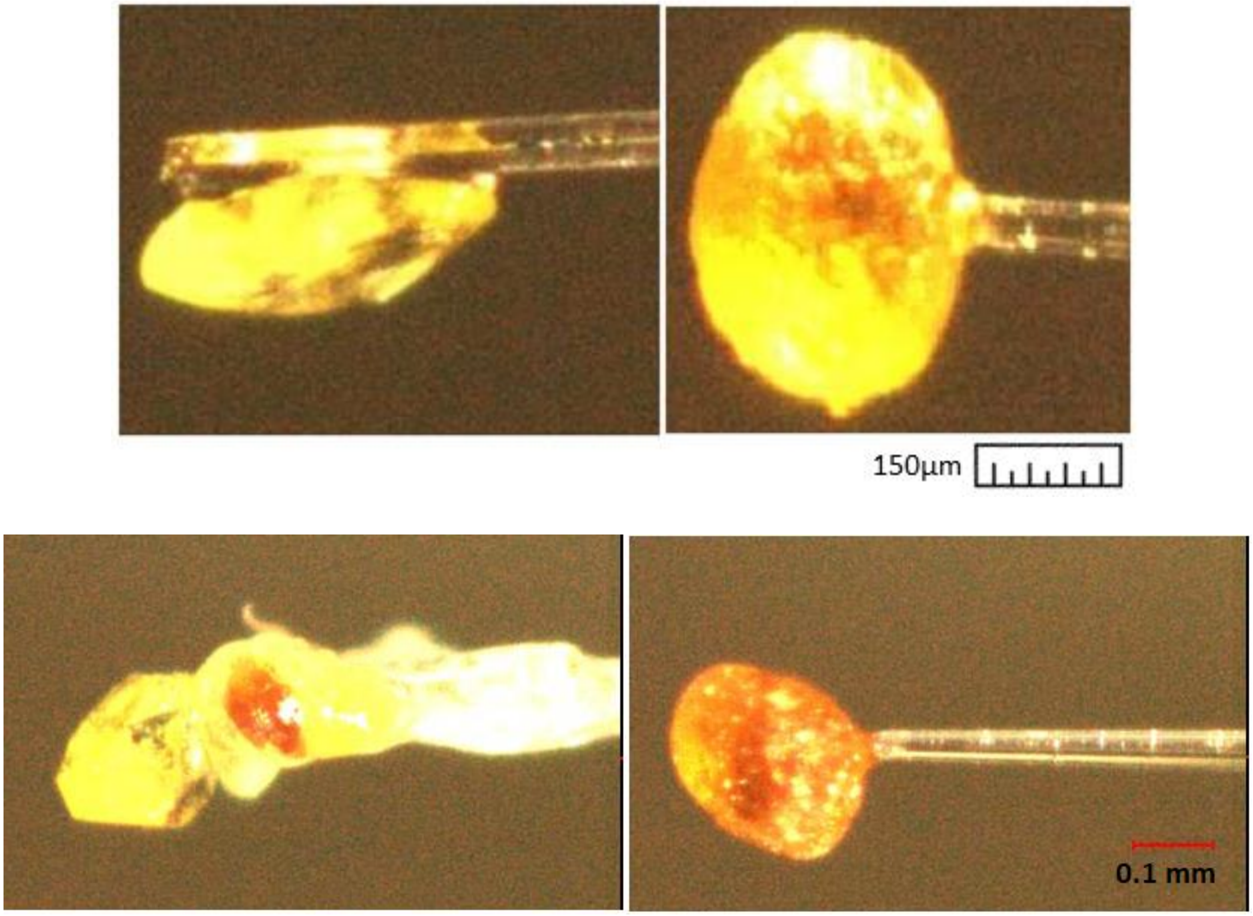

Natural shape **Spherical shape (test crystal)**

Figure S2. Images of different YLID crystals of natural shape and spherical shape (test crystal)

Table S2. Crystallographic information and measurement details for all 23 YLID datasets

Experiment	Dataset 1	Dataset 2	Dataset 3
**Crystal**	Natural shape 1	Natural shape 1	Natural shape 1
**Chemical formula**	$C_{11}H_{10}O_2S$	$C_{11}H_{10}O_2S$	$C_{11}H_{10}O_2S$
**Form. weight (g/mol)**	206.267	206.267	206.267
**Crystal size (mm^3)**	0.18 x 0.22x 0.24	0.18 x 0.22x 0.24	0.18 x 0.22x 0.24
**Crystal habit**	block	block	block
**Crystal color**	yellow	yellow	yellow
**Temperature (K)**	100	100	100
**Wavelength (Å)**	0.56087	1.54184	0.71073
**a (Å)**	5.85548(4)	5.85175(5)	5.84810(5)
**b (Å)**	8.92958(6)	8.93176(7)	8.92817(7)
**c (Å)**	18.32550(12)	18.32726(13)	18.33261(16)
**α (°)**	90	90	90
**β (°)**	90	90	90
**γ (°)**	90	90	90
**Volume (Å^3)**	958.184(11)	957.900(12)	957.197(13)
**Z, Z'**	4,1	4,1	4,1
**Space group**	P 2_1 2_1 2_1	P 2_1 2_1 2_1	P 2_1 2_1 2_1
**Number of refl.**	168178	38310	139070
**Rint/Compl./Red.**	3.68%/99.8%/19.88	4.83%/100%/19.82	8.47%/100%/21.93
**Unique reflections**	8455	1923	6328
**Unique observed [F>4sigma(F)]**	8038	1919	6131
**Reflns theta min (°)**	2.002	4.826	2.222
**Reflns theta max (°)**	34.824	73.267	41.057
**Resolution (Å)**	0.491	0.805	0.541
**Experiment**	**Dataset 4**	**Dataset 5**	**Dataset 6**
**Crystal**	Natural shape 4	Natural shape 2	Natural shape 5
**Chemical formula**	$C_{11}H_{10}O_2S$	$C_{11}H_{10}O_2S$	$C_{11}H_{10}O_2S$
**Form. weight (g/mol)**	206.267	206.267	206.267
**Crystal size (mm^3)**	0.23 x 0.20 x 0.11	0.30 x 0.18 x 0.19	0.15 x 0.15 x 0.08
**Crystal habit**	block	block	block
**Crystal color**	yellow	yellow	yellow
**Temperature (K)**	100	100	100
**Wavelength (Å)**	0.71073	0.71073	0.2483
**a (Å)**	5.85527(3)	5.85874(3)	5.85210(10)
**b (Å)**	8.93442(4)	8.94002(5)	8.93100(10)
**c (Å)**	18.33706(9)	18.34043(10)	18.3322(2)
**α (°)**	90	90	90
**β (°)**	90	90	90
**γ (°)**	90	90	90
**Volume (Å^3)**	959.274(8)	960.621(9)	958.13(2)
**Z, Z'**	4,1	4,1	4,1
**Space group**	P 2_1 2_1 2_1	P 2_1 2_1 2_1	P 2_1 2_1 2_1
**Number of refl.**	332277	271556	233408
**Rint/Compl./Red.**	3.26%/98.7%/32.25	4.33%/99.9%/36.42	5.60%/99.8%/16.89

**Unique reflections**	10302	7457	13818
**Unique observed [F>4sigma(F)]**	10114	7338	13615
**Reflns theta min (°)**	3.653	3.182	1.453
**Reflns theta max (°)**	50.579	43.844	17.319
**Resolution (Å)**	0.460	0.513	0.417
**Experiment**	**Dataset 7**	**Dataset 8**	**Dataset 9**
**Crystal**	Natural shape 6	Natural shape 7	Natural shape 1
**Chemical formula**	$C_{11}H_{10}O_2S$	$C_{11}H_{10}O_2S$	$C_{11}H_{10}O_2S$
**Form. weight (g/mol)**	206.267	206.267	206.267
**Crystal size (mm^3)**	0.20 x 0.15 x 0.05	0.15 x 0.15 x 0.15	0.18 x 0.22x 0.24
**Crystal habit**	block	block	block
**Crystal color**	yellow	yellow	yellow
**Temperature (K)**	100	100	150
**Wavelength (Å)**	0.2483	0.2483	0.56087
**a (Å)**	5.85190(10)	5.85250(10)	5.87365(4)
**b (Å)**	8.92990(10)	8.93240(10)	8.95341(7)
**c (Å)**	18.3315(2)	18.3323(2)	18.34006(15)
**α (º)**	90	90	90
**β (º)**	90	90	90
**γ (º)**	90	90	90
**Volume (Å^3)**	957.95(2)	958.36(2)	964.490(13)
**Z, Z’**	4,1	4,1	4,1
**Space group**	$P\ 2_1\ 2_1\ 2_1$	$P\ 2_1\ 2_1\ 2_1$	$P\ 2_1\ 2_1\ 2_1$
**Number of refl.**	164512	110142	153495
**Rint/Compl./Red.**	5.79%/99.8%/13.58	5.67%/99.9%/13.03	3.12%/99.9%/22.90
**Unique reflections**	12109	8456	6691
**Unique observed [F>4sigma(F)]**	11891	8278	6416
**Reflns theta min (°)**	1.442	1.442	1.997
**Reflns theta max (°)**	16.544	14.645	31.810
**Resolution (Å)**	0.436	0.491	0.532
**Experiment**	**Dataset 10**	**Dataset 11**	**Dataset 12**
**Crystal**	Natural shape 1	Natural shape 2	Natural shape 2
**Chemical formula**	$C_{11}H_{10}O_2S$	$C_{11}H_{10}O_2S$	$C_{11}H_{10}O_2S$
**Form. weight (g/mol)**	206.267	206.267	206.267
**Crystal size (mm^3)**	0.18 x 0.22x 0.24	0.30 x 0.18 x 0.19	0.30 x 0.18 x 0.19
**Crystal habit**	block	block	block
**Crystal color**	yellow	yellow	yellow
**Temperature (K)**	150	292	292
**Wavelength (Å)**	1.54184	0.56087	0.71073
**a (Å)**	5.87554(3)	5.95350(10)	5.95058(5)
**b (Å)**	8.95141(3)	9.03230(10)	9.03058(7)
**c (Å)**	18.33944(7)	18.3824(2)	18.38024(14)
**α (º)**	90	90	90
**β (º)**	90	90	90
**γ (º)**	90	90	90

**Volume (Å³)**	964.551(7)	988.49(2)	987.703(13)
**Z, Z'**	4,1	4,1	4,1
**Space group**	$P\ 2_1\ 2_1\ 2_1$	$P\ 2_1\ 2_1\ 2_1$	$P\ 2_1\ 2_1\ 2_1$
**Number of refl.**	39359	108903	88490
**Rint/Compl./Red.**	2.74%/100%/19.91	4.64%/99.9%/34.96	5.25%/100%/35.68
**Unique reflections**	1967	3099	2466
**Unique observed [F>4sigma(F)]**	1967	3039	2420
**Reflns theta min (°)**	4.823	1.982	2.216
**Reflns theta max (°)**	73.961	23.833	28.361
**Resolution (Å)**	0.802	0.694	0.748
**Experiment**	**Dataset 13**	**Dataset 14**	**Dataset 15**
**Crystal**	Test Crystal 1	Test Crystal 1	Test Crystal 2
**Chemical formula**	$C_{11}H_{10}O_2S$	$C_{11}H_{10}O_2S$	$C_{11}H_{10}O_2S$
**Form. weight (g/mol)**	206.267	206.267	206.267
**Crystal size (mm³)**	0.30 x 0.33 x 0.43	0.30 x 0.33 x 0.43	0.19 x 0.26 x 0.31
**Crystal habit**	spherical	spherical	spherical
**Crystal color**	yellow	yellow	yellow
**Temperature (K)**	292	292	292
**Wavelength (Å)**	0.56087	0.71073	1.54184
**a (Å)**	5.95476(7)	5.95336(3)	5.96061(3)
**b (Å)**	9.03021(10)	9.02867(4)	9.03790(4)
**c (Å)**	18.3841(2)	18.38155(10)	18.39811(8)
**α (°)**	90	90	90
**β (°)**	90	90	90
**γ (°)**	90	90	90
**Volume (Å³)**	988.563(19)	988.027(9)	991.132(8)
**Z, Z'**	4.1	4.1	4.1
**Space group**	$P\ 2_1\ 2_1\ 2_1$	$P\ 2_1\ 2_1\ 2_1$	$P\ 2_1\ 2_1\ 2_1$
**Number of refl.**	175024	82092	23937
**Rint/Compl./Red.**	3.20%/99.9%/27.51	3.64%/100%/18.87	2.67%/100%/11.83
**Unique reflections**	4285	4339	2041
**Unique observed [F>4sigma(F)]**	4189	4240	2033
**Reflns theta min (°)**	1.983	2.216	4.807
**Reflns theta max (°)**	26.752	34.965	74.754
**Resolution (Å)**	0.623	0.620	0.806
**Experiment**	**Dataset 16**	**Dataset 17**	**Dataset 18**
**Crystal**	Test Crystal 2	Test Crystal 2	Natural shape 2
**Chemical formula**	$C_{11}H_{10}O_2S$	$C_{11}H_{10}O_2S$	$C_{11}H_{10}O_2S$
**Form. weight (g/mol)**	206.267	206.267	206.267
**Crystal size (mm³)**	0.19.5 x 0.26.5 x 0.31	0.19.5 x 0.26.5 x 0.31	0.30 x 0.18 x 0.19
**Crystal habit**	spherical	spherical	block
**Crystal color**	yellow	yellow	yellow
**Temperature (K)**	292	292	295
**Wavelength (Å)**	0.71073	0.56087	1.54184
**a (Å)**	5.95844(4)	5.96057(7)	5.95897(4)

**b (Å)**	9.03357(6)	9.03800(12)	9.03629(4)
**c (Å)**	18.39122(12)	18.3993(2)	18.38963(8)
**α (º)**	90	90	90
**β (º)**	90	90	90
**γ (º)**	90	90	90
**Volume (Å^3)**	989.925(11)	991.20(2)	990.226(8)
**Z, Z'**	4,1	4,1	4,1
**Space group**	P 2_1 2_1 2_1	P 2_1 2_1 2_1	P 2_1 2_1 2_1
**Number of refl.**	46712	79140	38723
**Rint/Compl./Red.**	3.54%/100%/12.76	3.10%/100%/18.91	4.06%/100%/19.18
**Unique reflections**	3647	4172	1982
**Unique observed [F>4sigma(F)]**	3511	3864	1969
**Reflns theta min (°)**	2.215	1.9800	4.809
**Reflns theta max (°)**	32.737	28.2310	72.793
**Resolution (Å)**	0.657	0.629	0.807
**Experiment**	**Dataset 19**	**Dataset 20**	**Dataset 21**
**Crystal**	Test Crystal 1	Natural shape 3	Test Crystal 3
**Chemical formula**	$C_{11}H_{10}O_2S$	$C_{11}H_{10}O_2S$	$C_{11}H_{10}O_2S$
**Form. weight (g/mol)**	206.267	206.267	206.267
**Crystal size (mm^3)**	0.30 x 0.33 x 0.43	0.05 x 0.08 x 0.11	0.29 x 0.30 x 0.36
**Crystal habit**	spherical	block	spherical
**Crystal color**	yellow	yellow	yellow
**Temperature (K)**	292	292	292
**Wavelength (Å)**	1.54184	1.54184	1.54184
**a (Å)**	5.95979(2)	5.96425(5)	5.95594(2)
**b (Å)**	9.03581(3)	9.04332(6)	9.03225(3)
**c (Å)**	18.38895(6)	18.40163(13)	18.38486(7)
**α (º)**	90	90	90
**β (º)**	90	90	90
**γ (º)**	90	90	90
**Volume (Å^3)**	990.274(6)	992.522(13)	989.024(6)
**Z, Z'**	4,1	4,1	4,1
**Space group**	P 2_1 2_1 2_1	P 2_1 2_1 2_1	P 2_1 2_1 2_1
**Number of refl.**	39185	38486	39465
**Rint/Compl./Red.**	2.76%/100%/18.53	3.78%/100%/19.32	2.81%/100%/18.54
**Unique reflections**	2114	1992	2128
**Unique observed [F>4sigma(F)]**	2112	1956	2128
**Reflns theta min (°)**	4.810	4.806	4.811
**Reflns theta max (°)**	77.632	72.758	78.360
**Resolution (Å)**	0.789	0.807	0.787
**Experiment**	**Dataset 22**	**Dataset 23**	
**Crystal**	Test Crystal 4	Test Crystal 5	
**Chemical formula**	$C_{11}H_{10}O_2S$	$C_{11}H_{10}O_2S$	
**Form. weight (g/mol)**	206.267	206.267	
**Crystal size (mm^3)**	0.32 x 0.32 x 0.37	0.28 x 0.26 x 0.21	

**Crystal habit**	spherical	spherical	
**Crystal color**	yellow	yellow	
**Temperature (K)**	292	292	
**Wavelength (Å)**	1.54184	1.54184	
**a (Å)**	5.95654(3)	5.96351(2)	
**b (Å)**	9.03338(5)	9.04006(3)	
**c (Å)**	18.38582(8)	18.39659(5)	
**α (º)**	90	90	
**β (º)**	90	90	
**γ (º)**	90	90	
**Volume (Å^3)**	989.299(8)	991.768(5)	
**Z, Z'**	4,1	4,1	
**Space group**	$P\ 2_1\ 2_1\ 2_1$	$P\ 2_1\ 2_1\ 2_1$	
**Number of refl.**	21113	84284	
**Rint/Compl./Red.**	2.31%/100%/10.41	2.87%/100%/39.00	
**Unique reflections**	2027	2161	
**Unique observed [F>4sigma(F)]**	2026	2160	
**Reflns theta min (°)**	4.810	4.808	
**Reflns theta max (°)**	74.492	79.499	
**Resolution (Å)**	0.800	0.784	

Table S3. Refinement statistics- IAM

	**Dataset 1**	**Dataset 2**	**Dataset 3**	**Dataset 4**
**Number of parameters**	167[a]	168[a]	167	167
**R factor (obs)**	0.0211	0.0212	0.0251	0.0161
**R factor (all)**	0.0230	0.0212	0.0263	0.0165
**wR factor (obs)**	0.0600	0.0561	0.0692	0.0474
**Goodness of fit**	1.087	1.052	1.105	1.122
**Residual density max (e/Å^3)**	0.276	0.158	0.278	0.248
**Residual density min (e/Å^3)**	-0.127	-0.187	-0.268	-0.108
	**Dataset 5**	**Dataset 6**	**Dataset 7**	**Dataset 8**
**Number of parameters**	167	167	167	167
**R factor (obs)**	0.0203	0.0170	0.0184	0.0221
**R factor (all)**	0.0209	0.0172	0.0188	0.0225
**wR factor (obs)**	0.0598	0.0483	0.0528	0.0629

**Goodness of fit**	1.172	1.128	1.100	1.095
**Residual density max (e/Å^3)**	0.350	0.226	0.215	0.333
**Residual density min (e/Å^3)**	-0.117	-0.169	-0.182	-0.405
	**Dataset 9**	**Dataset 10**	**Dataset 11**	**Dataset 12**
**Number of parameters**	167	168	167	167
**R factor (obs)**	0.0219	0.0189	0.0299	0.0237
**R factor (all)**	0.0233	0.0189	0.0304	0.0243
**wR factor (obs)**	0.0624	0.0503	0.0790	0.0676
**Goodness of fit**	1.079	1.083	1.077	1.071
**Residual density max (e/Å^3)**	0.232	0.169	0.179	0.115
**Residual density min (e/Å^3)**	-0.094	-0.171	-0.089	-0.087
	**Dataset 13**	**Dataset 14**	**Dataset 15**	**Dataset 16**
**Number of parameters**	167	167	168	167
**R factor (obs)**	0.0268	0.0267	0.0210	0.0280
**R factor (all)**	0.0273	0.0274	0.0210	0.0290
**wR factor (obs)**	0.0755	0.0784	0.0580	0.0797
**Goodness of fit**	1.065	1.115	1.110	1.105
**Residual density max (e/Å^3)**	0.172	0.159	0.122	0.139
**Residual density min (e/Å^3)**	-0.075	-0.105	-0.147	-0.091
	**Dataset 17**	**Dataset 18**	**Dataset 19**	**Dataset 20**
**Number of parameters**	167	167	168	167
**R factor (obs)**	0.0303	0.0223	0.0212	0.0207
**R factor (all)**	0.0338	0.0224	0.0212	0.0212
**wR factor (obs)**	0.0811	0.0606	0.0560	0.0562
**Goodness of fit**	1.064	1.066	1.090	1.072
**Residual density max (e/Å^3)**	0.154	0.114	0.092	0.123
**Residual density min (e/Å^3)**	-0.100	-0.148	-0.167	-0.173

	**Dataset 21**	**Dataset 22**	**Dataset 23**	
**Number of parameters**	168	168	168	
**R factor (obs)**	0.0216	0.0223	0.0214	
**R factor (all)**	0.0216	0.0223	0.0214	
**wR factor (obs)**	0.0577	0.0583	0.0567	
**Goodness of fit**	1.085	1.075	1.071	
**Residual density max (e/Å^3)**	0.129	0.130	0.102	
**Residual density min (e/Å^3)**	-0.142	-0.151	-0.118	

[a] The difference in the number of refined parameters is the extinction coefficient.

Table S4. Refinement statistics for HAR

	**Dataset 1**	**Dataset 2**	**Dataset 3**	**Dataset 4**
**Number of parameters**	242	242	242	241
**R factor (obs)**	0.0118	0.0100	0.0157	0.0078
**wR factor (obs)**	0.0073	0.0102	0.0148	0.0196
**Goodness of fit**	1.4051	2.3277	1.4772	0.9192
**Residual density max (e/Å^3)**	0.110	0.078	0.119	0.099
**Residual density min (e/Å^3)**	-0.076	-0.053	-0.136	-0.065
	**Dataset 5**	**Dataset 6**	**Dataset 7**	**Dataset 8**
**Number of parameters**	241	241	241	241
**R factor (obs)**	0.0100	0.0088	0.0107	0.0157
**wR factor (obs)**	0.0228	0.0236	0.0248	0.0381
**Goodness of fit**	1.4365	1.3448	1.2982	1.3676
**Residual density max (e/Å^3)**	+0.189	0.111	0.113	0.282
**Residual density min (e/Å^3)**	-0.072	-0.104	-0.167	-0.516
	**Dataset 9**	**Dataset 10**	**Dataset 11**	**Dataset 12**
**Number of parameters**	242	242	242	242
**R factor (obs)**	0.0110	0.0070	0.0141	0.0133
**wR factor (obs)**	0.0070	0.0071	0.0140	0.0158
**Goodness of fit**	1.6457	2.6765	2.5270	3.1758

**Residual density max (e/Å^3)**	0.091	0.070	0.108	0.069
**Residual density min (e/Å^3)**	-0.059	-0.046	-0.052	-0.060
	**Dataset 13**	**Dataset 14**	**Dataset 15**	**Dataset 16**
**Number of parameters**	242	242	242	242
**R factor (obs)**	0.0112	0.0125	0.0110	0.0164
**wR factor (obs)**	0.0279	0.0296	0.0237	0.0160
**Goodness of fit**	0.9590	1.1869	1.1366	2.7276
**Residual density max (e/Å^3)**	0.089	0.075	0.070	0.087
**Residual density min (e/Å^3)**	-0.044	-0.070	-0.076	-0.073
	**Dataset 17**	**Dataset 18**	**Dataset 19**	**Dataset 20**
**Number of parameters**	242	241	242	241
**R factor (obs)**	0.0178	0.0101	0.0120	0.0097
**wR factor (obs)**	0.0303	0.0257	0.0316	0.0208
**Goodness of fit**	1.2358	1.2241	1.8109	1.1641
**Residual density max (e/Å^3)**	0.145	0.046	0.081	0.059
**Residual density min (e/Å^3)**	-0.091	-0.050	-0.072	-0.057
	**Dataset 21**	**Dataset 22**	**Dataset 23**	
**Number of parameters**	242	242	242	
**R factor (obs)**	0.0125	0.0103	0.0108	
**wR factor (obs)**	0.0277	0.0236	0.0251	
**Goodness of fit**	1.1285	1.1887	1.1647	
**Residual density max (e/Å^3)**	0.097	0.059	0.077	
**Residual density min (e/Å^3)**	-0.095	-0.072	-0.053	

Table S5. Refinement statistics for XCW fitting.

	Dataset 1	Dataset 2	Dataset 3	Dataset 4
**R factor (obs)**	0.0103	0.0076	0.0141	0.0071
**wR factor (obs)**	0.0055	0.0075	0.0130	0.0058
**Goodness of fit**	1.0798	1.7161	1.3039	1.7291
**Residual density max (e/Å^3)**	0.089	0.051	0.102	0.101
**Residual density min (e/Å^3)**	-0.071	-0.044	-0.116	-0.059
**λ step**	0.01	0.01	0.01	0.01
**Maximum λ**	0.18	0.11	076	0.06
	**Dataset 5**	**Dataset 6**	**Dataset 7**	**Dataset 8**
**R factor (obs)**	0.0089	0.0092	0.0101	0.0119
**wR factor (obs)**	0.0098	0.0122	0.0122	0.0168
**Goodness of fit**	2.2830	1.5361	1.2686	1.2691
**Residual density max (e/Å^3)**	0.165	0.064	0.088	0.183
**Residual density min (e/Å^3)**	-0.102	-0.077	-0.102	-0.223
**λ step**	0.01	0.1	0.1	0.1
**Maximum λ**	0.15	0.3	0.8	1.3
	**Dataset 9**	**Dataset 10**	**Dataset 11**	**Dataset 12**
**R factor (obs)**	0.0093	0.0058	0.0102	0.0109
**wR factor (obs)**	0.0051	0.0056	0.0100	0.0131
**Goodness of fit**	1.2134	2.1070	1.8129	2.6443
**Residual density max (e/Å^3)**	0.074	0.050	0.063	0.048
**Residual density min (e/Å^3)**	-0.052	-0.038	-0.044	-0.055
**λ step**	0.01	0.01	0.01	0.01
**Maximum λ**	0.09	0.03	0.09	0.12
	**Dataset 13**	**Dataset 14**	**Dataset 15**	**Dataset 16**
**R factor (obs)**	0.0096	0.0114	0.0062	0.0135
**wR factor (obs)**	0.0097	0.0128	0.0068	0.0131
**Goodness of fit**	1.9620	2.6471	1.5757	2.2480
**Residual density max (e/Å^3)**	0.093	0.095	0.025	0.058
**Residual density min (e/Å^3)**	-0.090	-0.096	-0.028	-0.057
**λ step**	0.01	0.01	0.01	0.01
**Maximum λ**	0.12	0.18	0.16	0.26

	Dataset 17	Dataset 18	Dataset 19	Dataset 20
**R factor (obs)**	0.0146	0.0088	0.0097	0.0083
**wR factor (obs)**	0.0074	0.0120	0.0148	0.0089
**Goodness of fit**	1.4751	2.8636	4.7687	1.9446
**Residual density max (e/Å^3)**	0.075	0.055	0.105	0.045
**Residual density min (e/Å^3)**	-0.069	-0.073	-0.035	-0.059
**λ step**	0.01	0.01	0.01	0.01
**Maximum λ**	0.10	0.08	0.04	0.12
	**Dataset 21**	**Dataset 22**	**Dataset 23**	
**R factor (obs)**	0.0094	0.0099	0.0093	
**wR factor (obs)**	0.0101	0.0103	0.0101	
**Goodness of fit**	3.6951	3.3451	4.8083	
**Residual density max (e/Å^3)**	0.059	0.061	0.057	
**Residual density min (e/Å^3)**	-0.044	-0.067	-0.047	
**λ step**	0.01	0.01	0.005	
**Maximum λ**	0.03	0.05	0.030	

Table S6. Refinement statistics for multipole refinement

	Dataset 1	Dataset 3	Dataset 16	Dataset 17
**Number of parameters**	541	541	541	541
**R factor (obs)**	0.0110	0.0147	0.0141	0.0143
**wR factor (obs)**	0.0065	0.0138	0.0134	0.0073
**Goodness of fit**	1.3047	1.4561	2.4957	1.5711
**Residual density max (e/Å^3)**	0.090	0.121	0.066	0.072
**Residual density min (e/Å^3)**	-0.082	-0.118	-0.066	-0.066

Refinement of anharmonic motion

In some datasets, a significant residual density pattern was observed for the sulfur atom. Therefore, Gram-Charlier parameters describing anharmonic atomic motions were refined for those atoms during the HAR and MM refinements. As a result, the min/max values of residual density and R factors of the refinement improved and the residual density pattern diminished (Figure S3 (a)). To evaluate whether it makes sense physically to refine anharmonic motions, we checked the total PDF (probability density function) isosurfaces along with the Cijk and Dijkl values (Gram-Charlier parameters, values in the deposited CIFs) of the refinements. For every anharmonic refinement, there are some clearly significant Cijk and Dijkl values, bigger than three times the standard uncertainty. Overall, Cijk and Dijkl values become more significant with increasing temperature. The PDF isosurfaces are shown for some of the datasets across the series of all refinements (Figure S3 (b)). There are only four cases with negative regions, all of them at the periphery and not in the center of the PDF. The percentages are 0.34, 0.63 and 1.57%, and in case of dataset 4 too small to be calculated. This gives us confidence that the refined models are physically meaningful, even if Kuhs' resolution rule is broken.[12]

(a)

**Dataset**	**HAR without anharmonic refinement**	**HAR with anharmonic refinement**
**4 (Mo)**	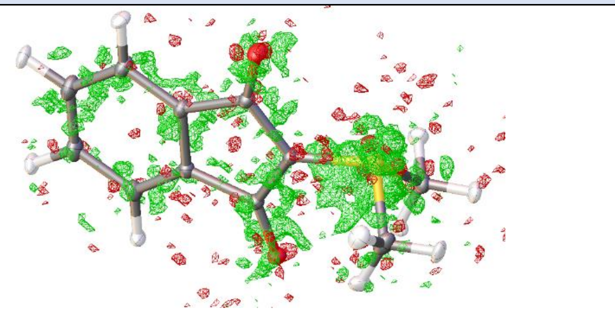	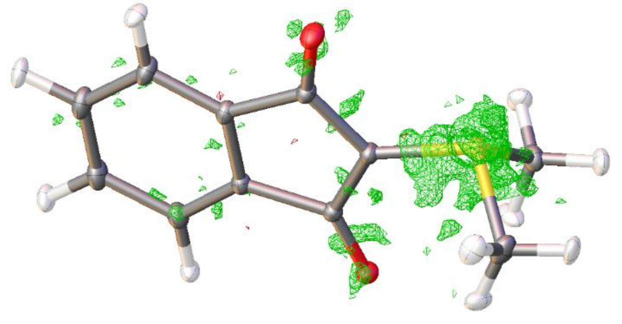
**13 (Ag)**	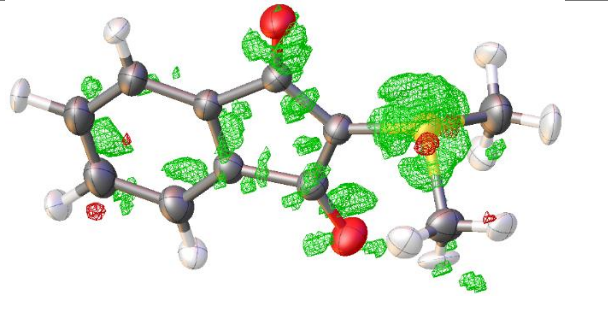	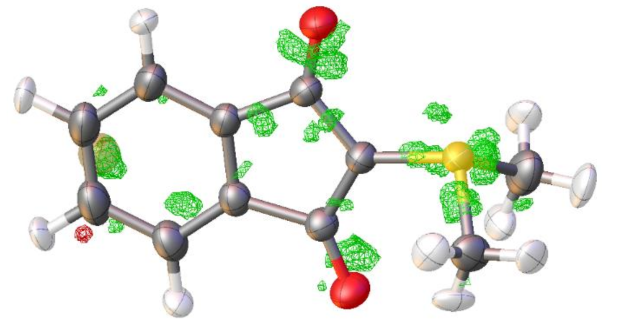

**19 (Cu)**	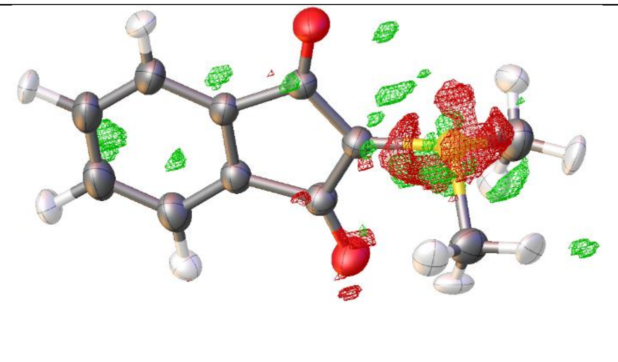	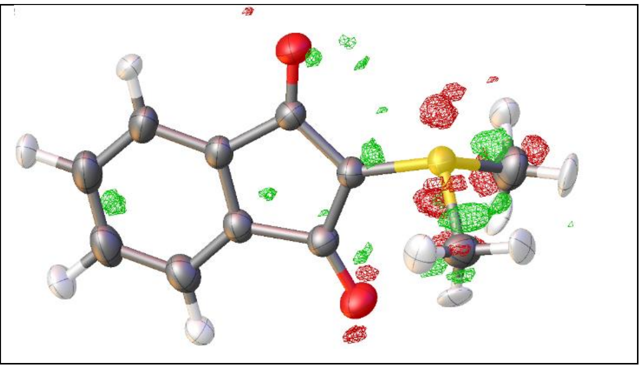

(b)

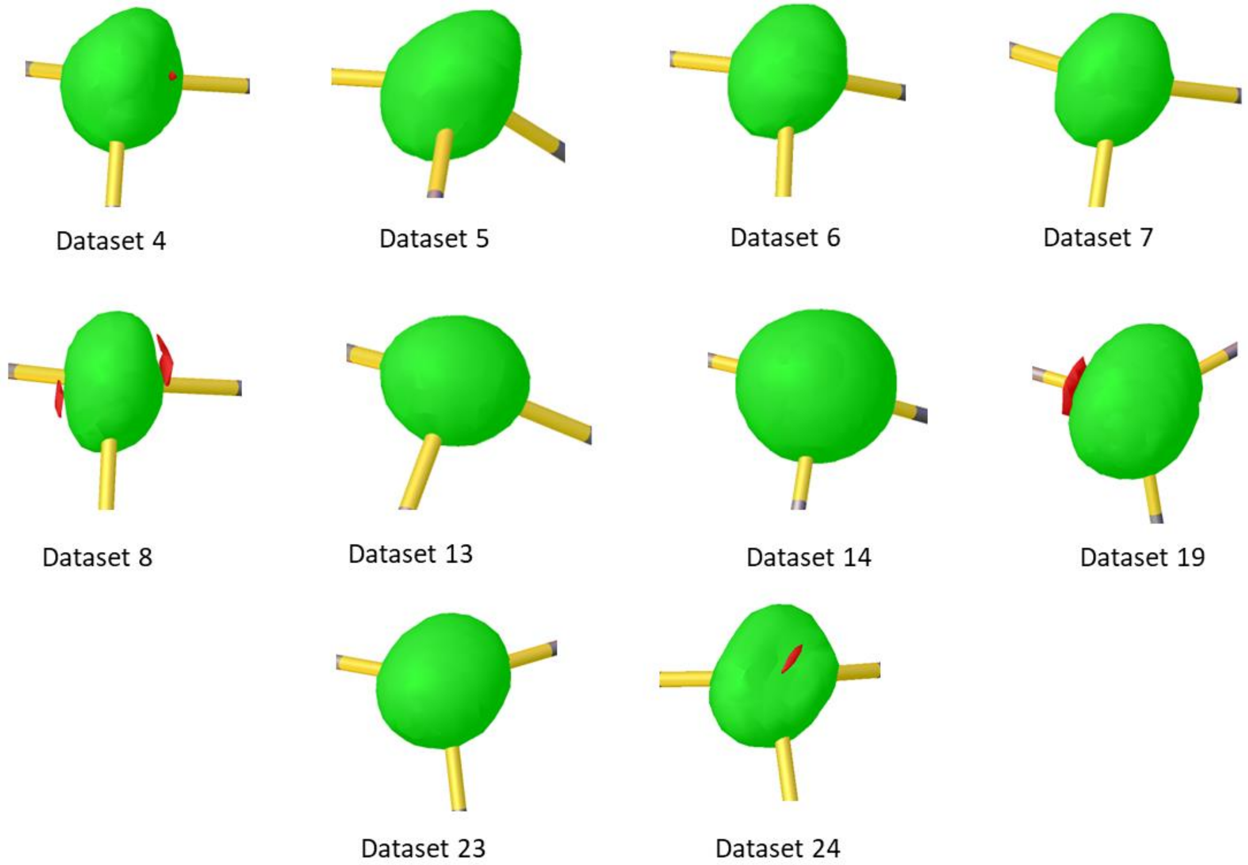

Figure S3. (a) Three-dimensional residual electron-density maps (isovalue = 0.05 e/Å^3, green = positive, red = negative) before (left) and after (right) refinement of Gram-Charlier parameters. (b) Total PDF (probability density function), 2nd to 4th order for the sulfur atom in some of the datasets using the Olex2 software. Isosurfaces at 50% probability level. Green = positive, red = negative.

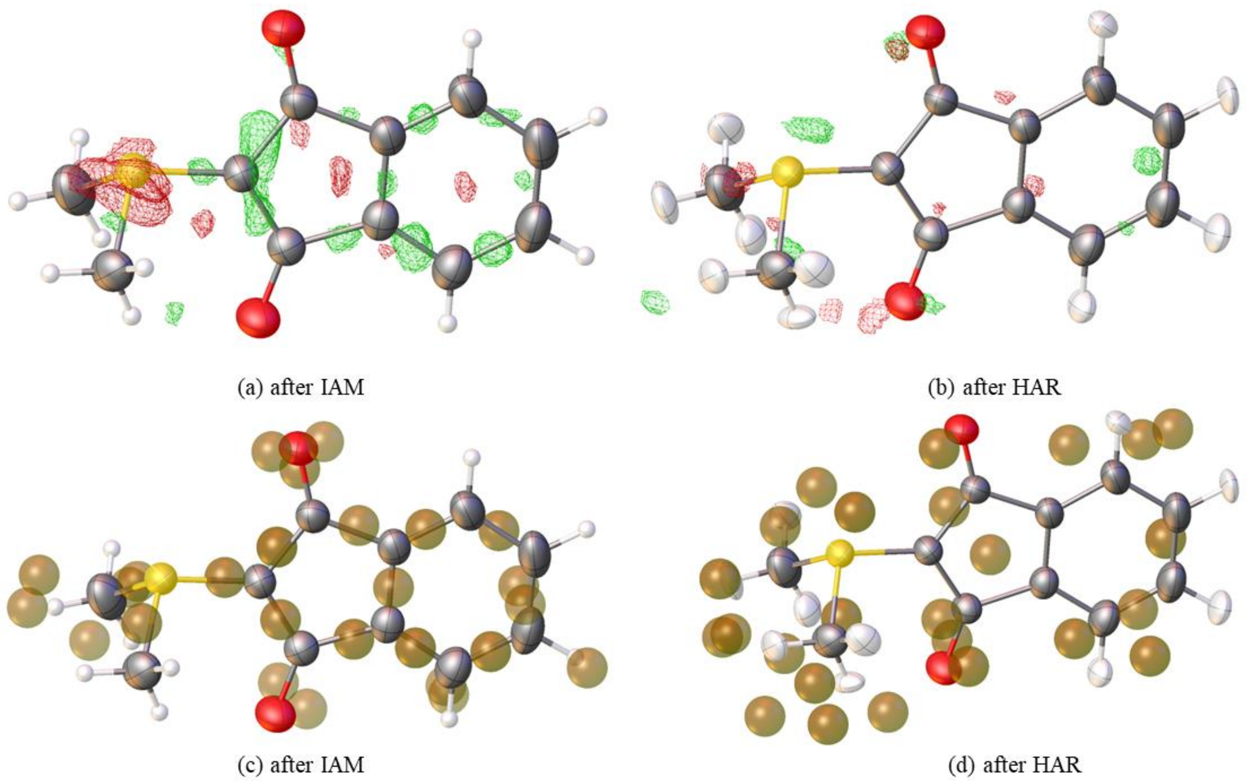

Figure S4. **(a)** Three-dimensional residual electron-density map (isovalue = 0.07 e/Å$^{3.}$ green = positive, red = negative) after IAM, **(b)** three-dimensional residual electron-density map (isovalue = 0.07 e/Å$^{3.}$ green = positive, red = negative) after HAR, **(c)** alternative representation of residual electron density as Q-peaks (local residual density maxima) after IAM (five highest peaks: Q1= 0.160. Q2= 0.140. Q3= 0.130. Q4= 0.120. Q5= 0.110 e/Å^3), **(d)** Q-peaks after HAR (five highest peaks: Q1= 0.050. Q2= 0.040. Q3= 0.040. Q4= 0.040. Q5= 0.040 e/Å^3). For dataset 15 (λ=Cu, r.t., d_{max} = 0.806 Å). Pictures generated with Olex2.

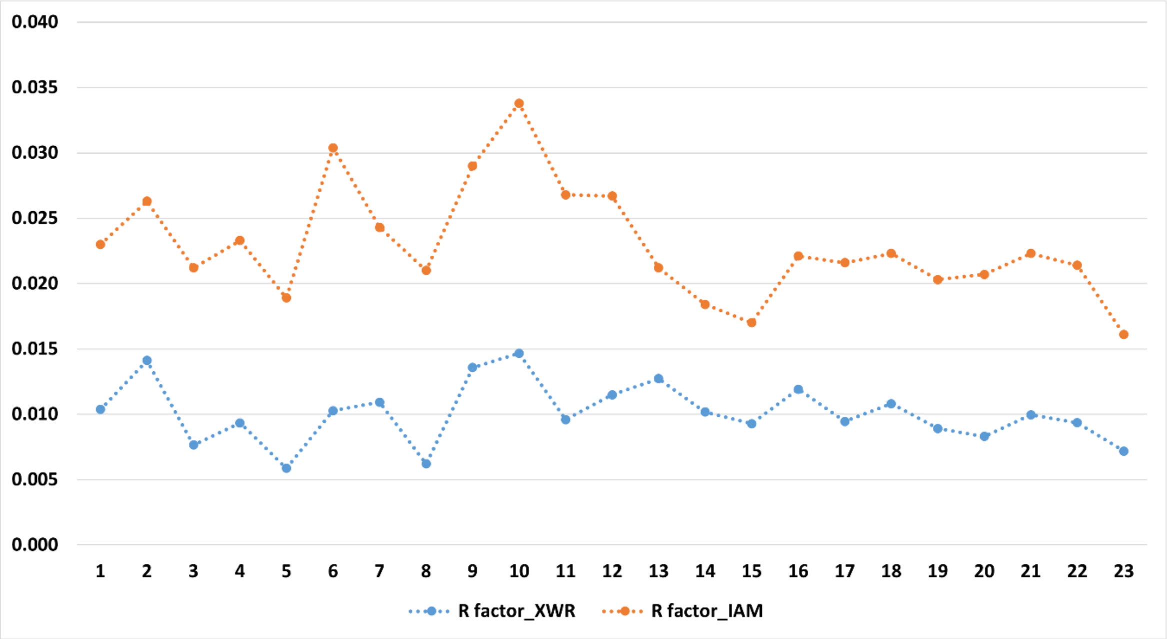

(a)

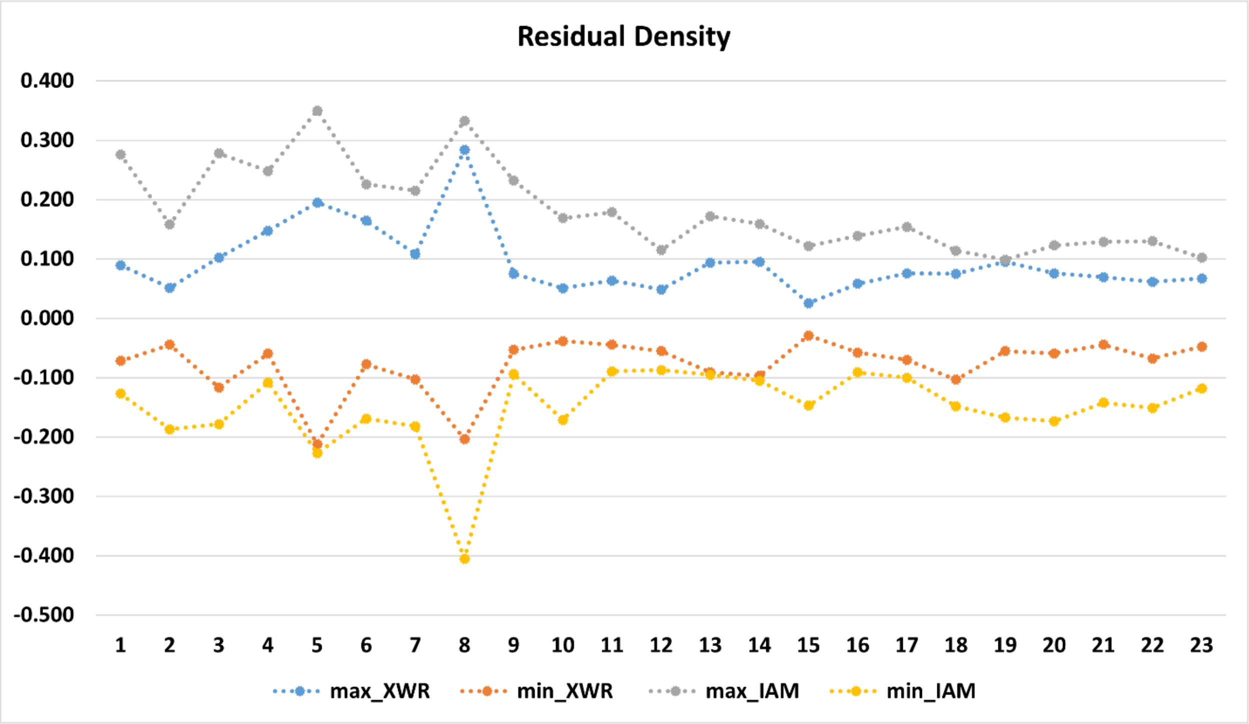

(b)

Figure S5. Comparison between IAM and XWR refinement models for datasets 1-23. **(a)** R factor; **(b)** Residual density (e/Å^3).

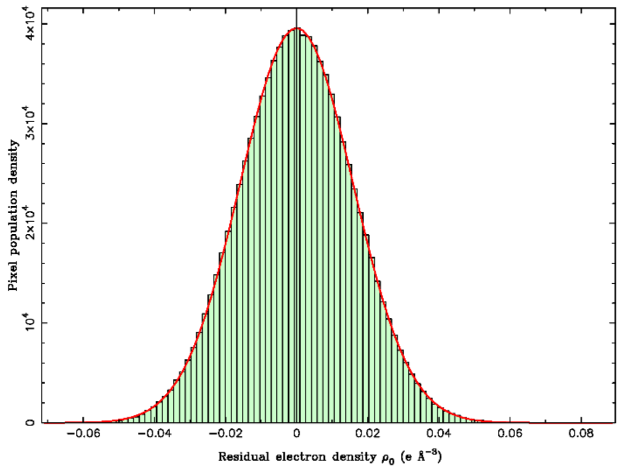

Dataset 1 – XWR

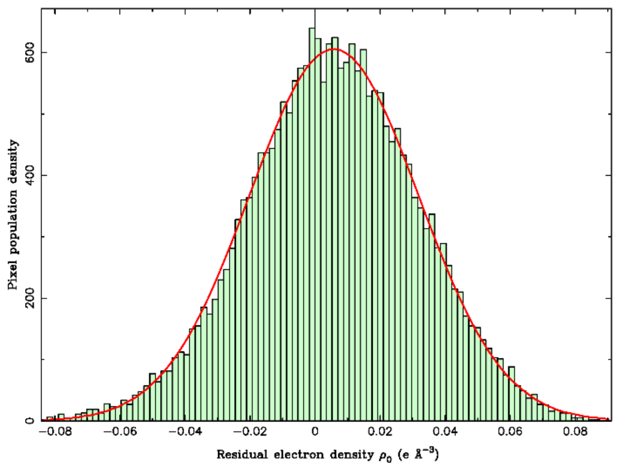

Dataset 1 – MM

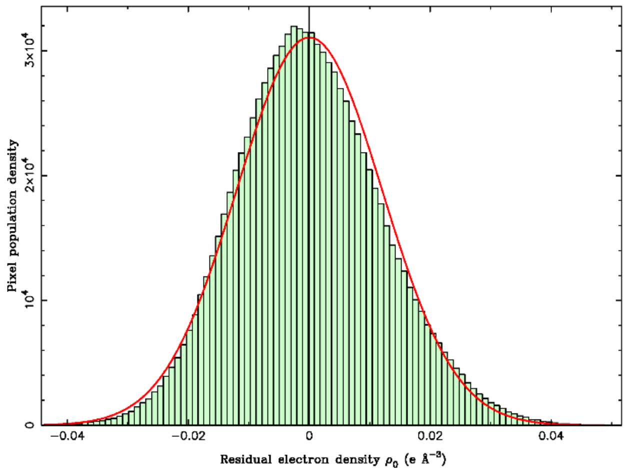

Dataset 2 – XWR

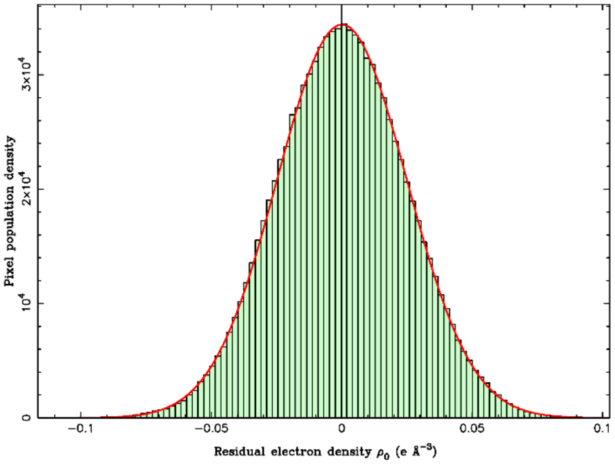

Dataset 3 – XWR

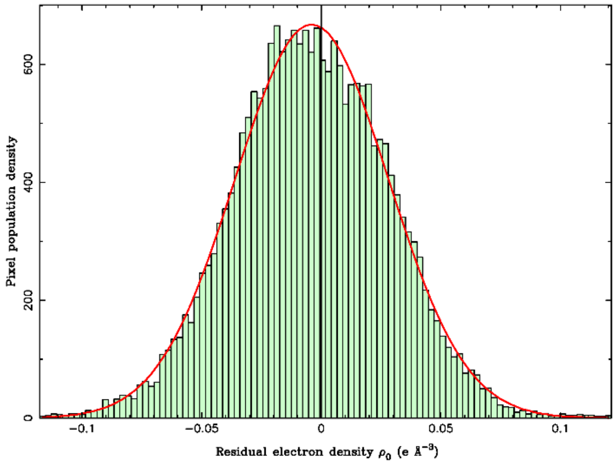

Dataset 3 – MM

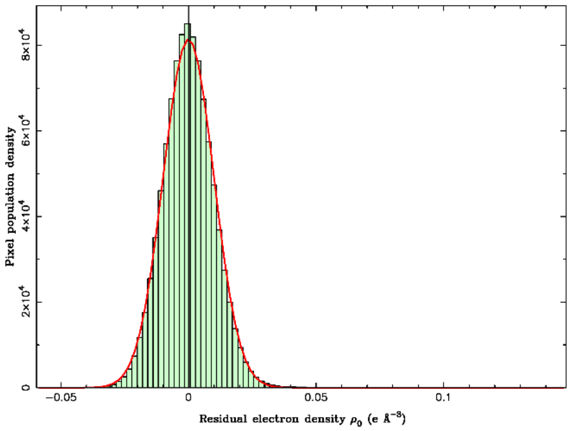

Dataset 4 – XWR

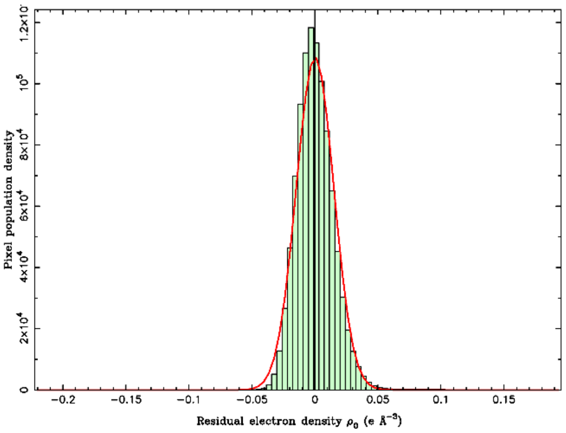

Dataset 5 - XWR

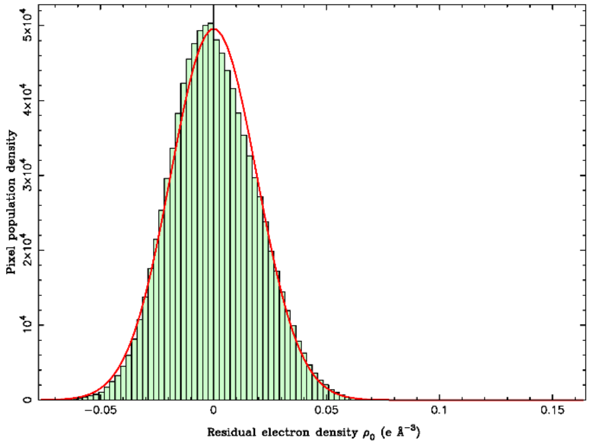

Dataset 6 – XWR

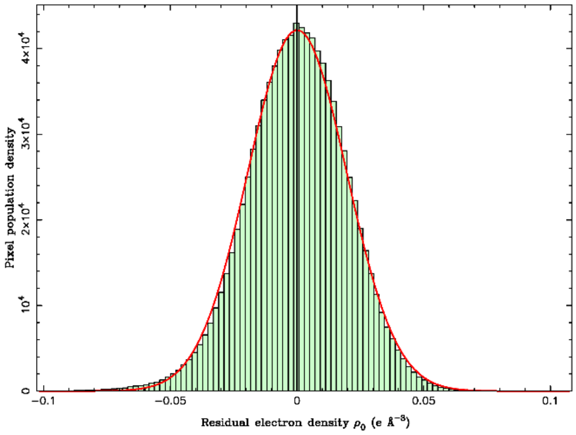

Dataset 7 – XWR

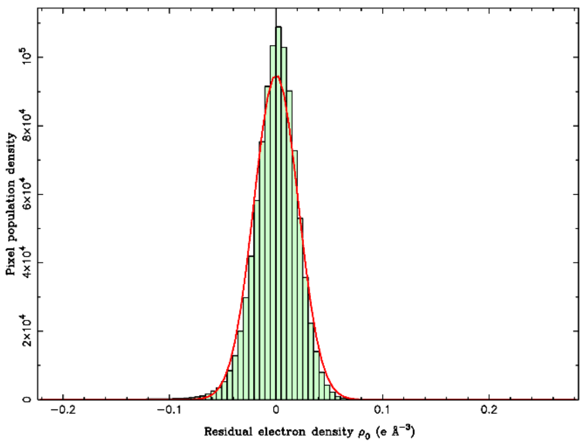

Dataset 8 – XWR

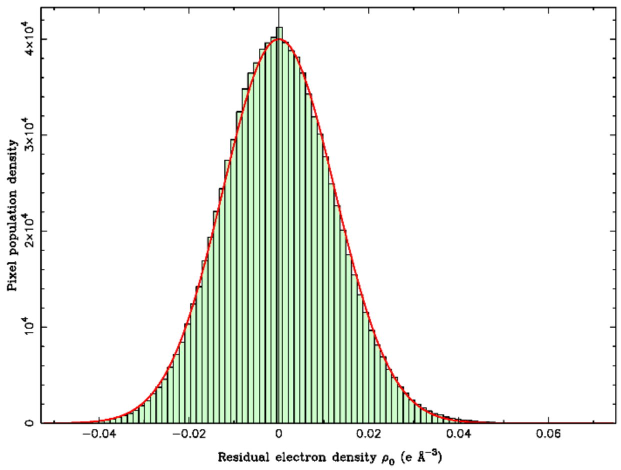

Dataset 9 – XWR

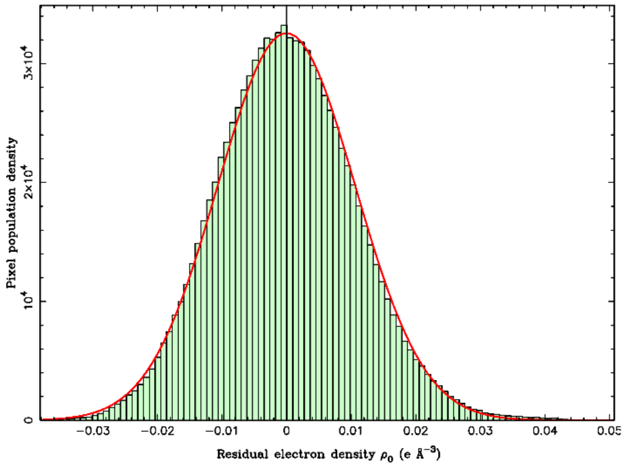

Dataset 10 – XWR

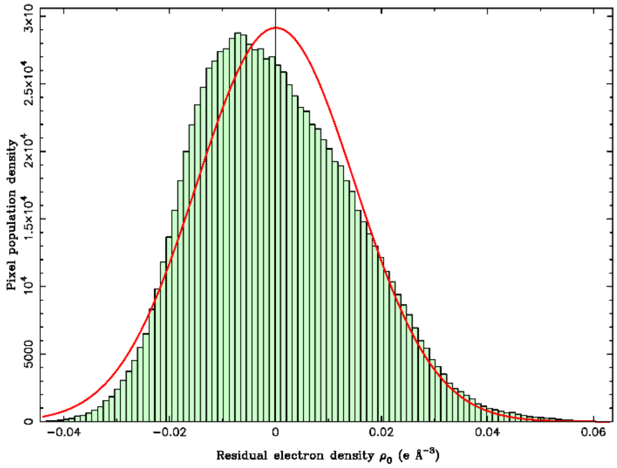

Dataset 11 – XWR

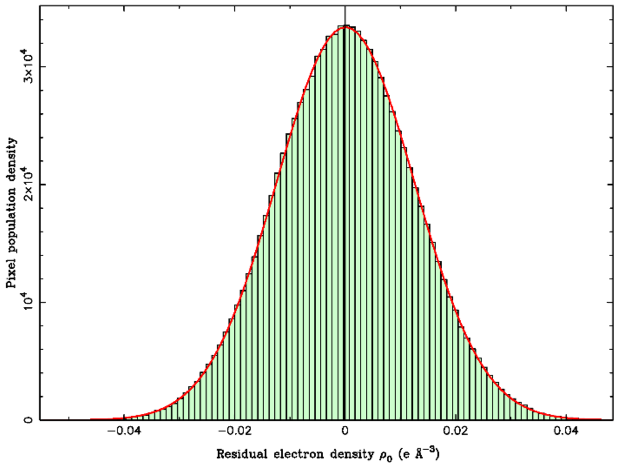

Dataset 12 - XWR

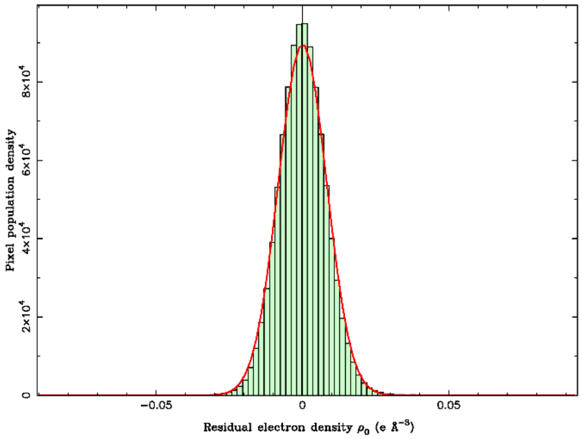

Dataset 13 - XWR

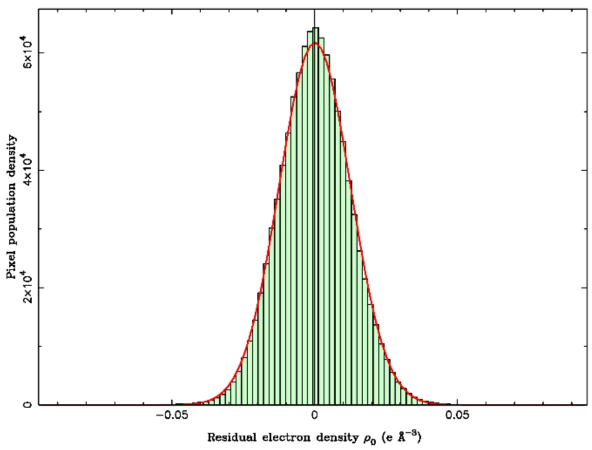

Dataset 14 – XWR

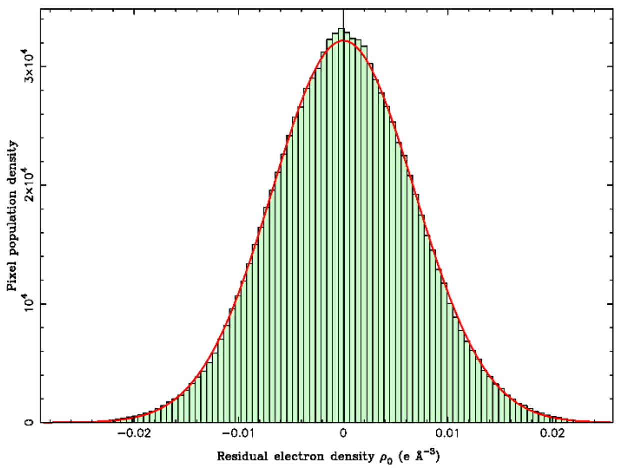

Dataset 15 – XWR

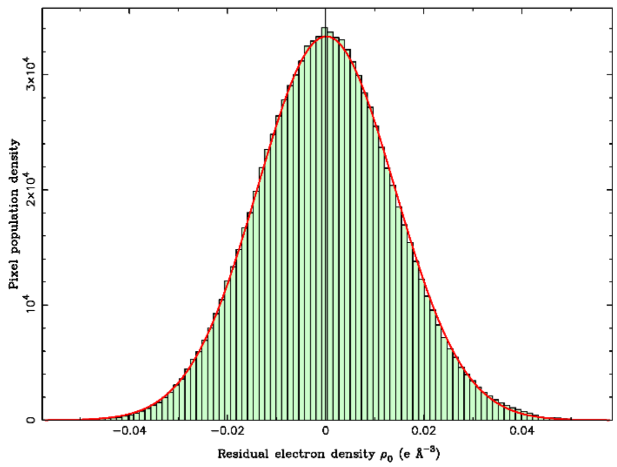

Dataset 16 – XWR

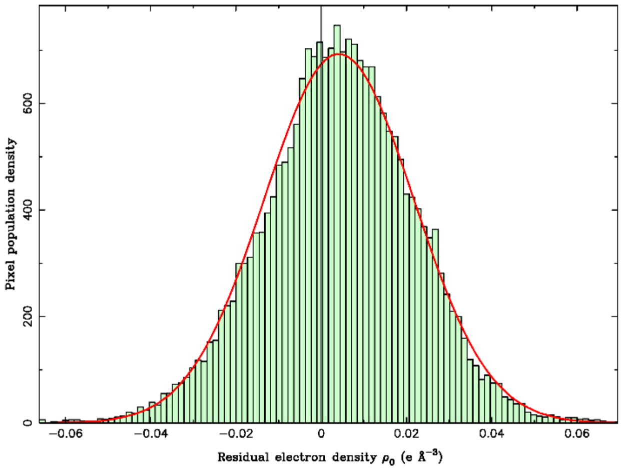

Dataset 16 – MM

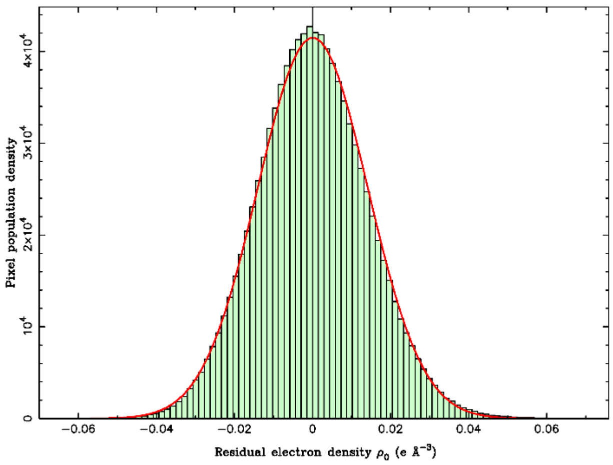

Dataset 17 – XWR

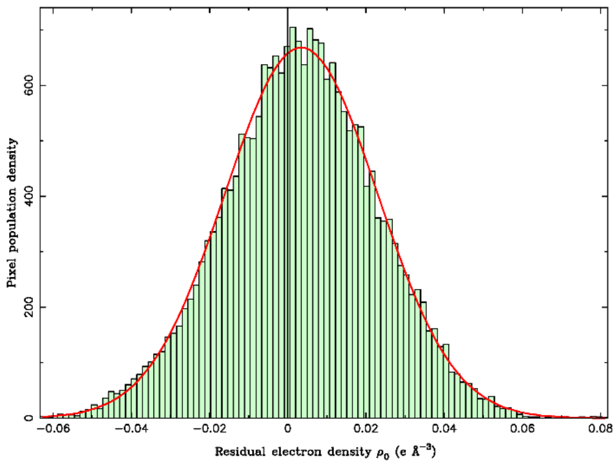

Dataset 17 – MM

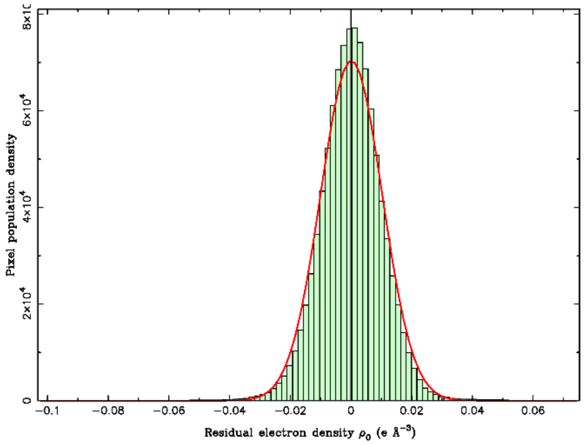

Dataset 18 – XWR

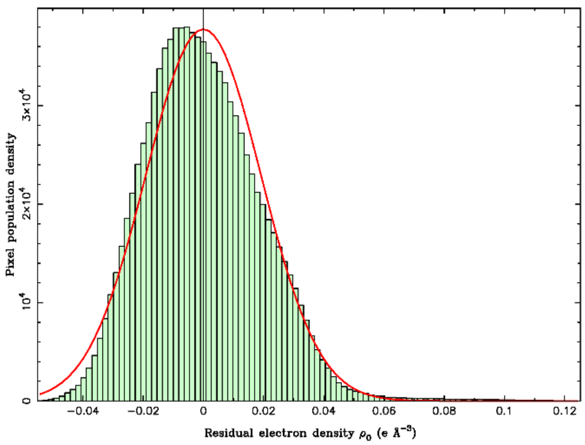

Dataset 19 – XWR

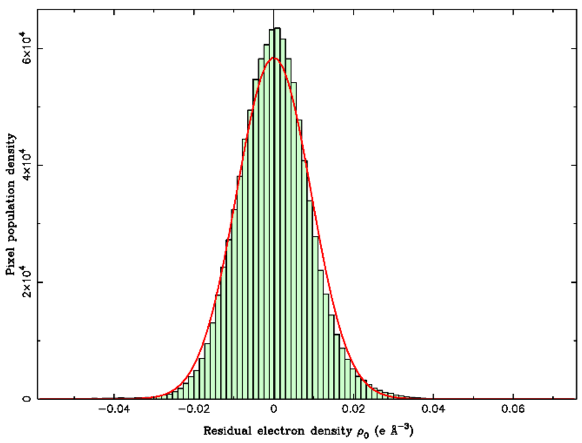

Dataset 20 – XWR

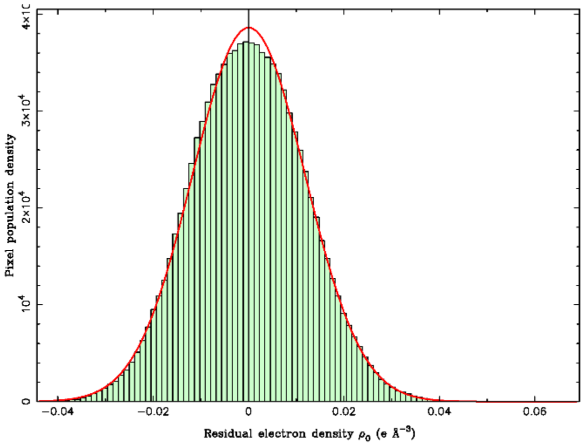

Dataset 21 – XWR

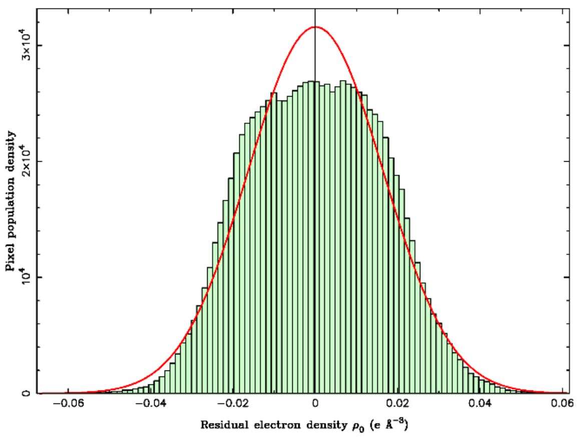

Dataset 22 – XWR

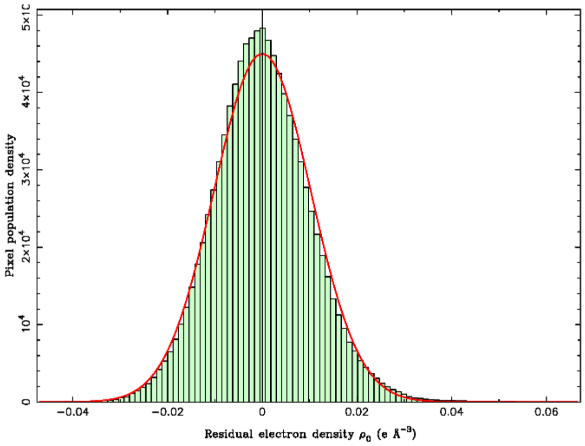

Dataset 23 – XWR

Figure S6. Probability distribution histogram plots based on the complete unit-cell electron density after XWR and multipole (MM) refinements using WinGX Software.

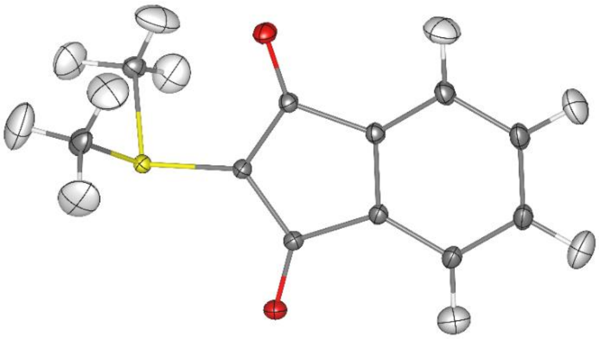

Dataset 1

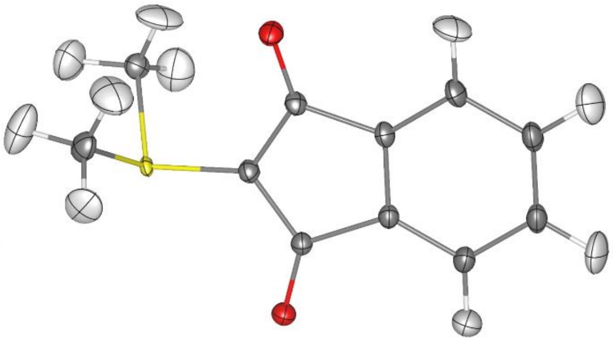

Dataset 2

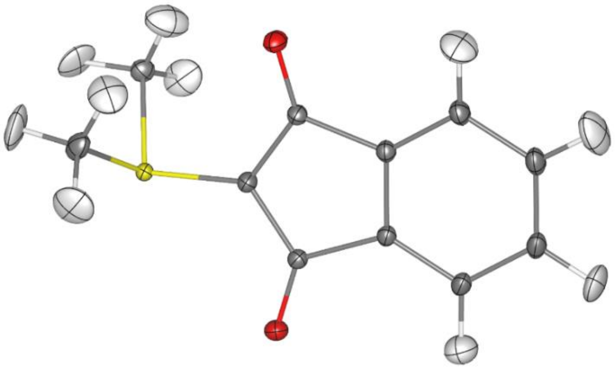

Dataset 3

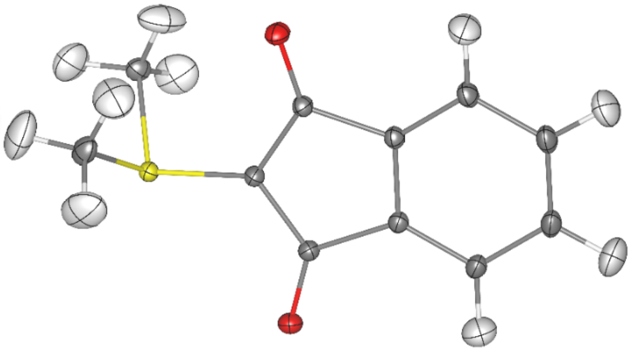

Dataset 4

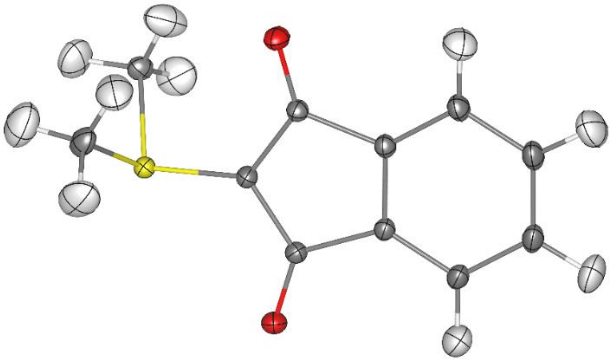

Dataset 5

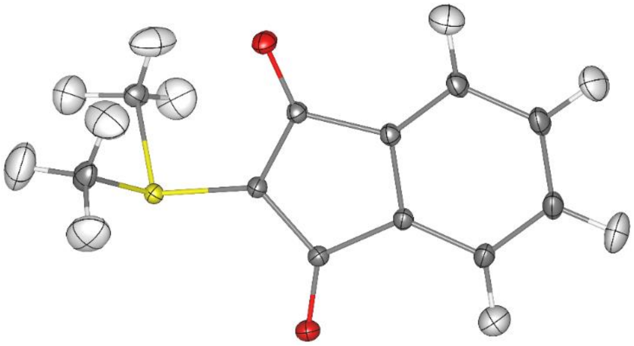

Dataset 6

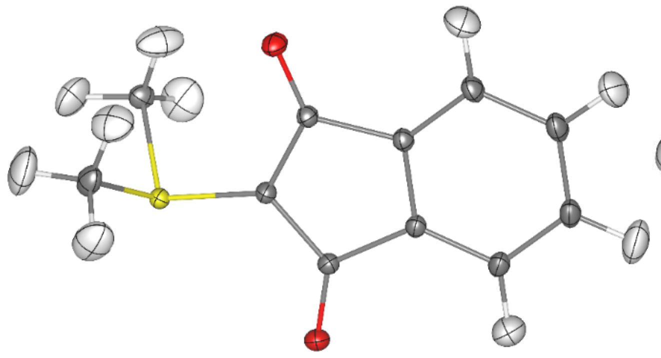

Dataset 7

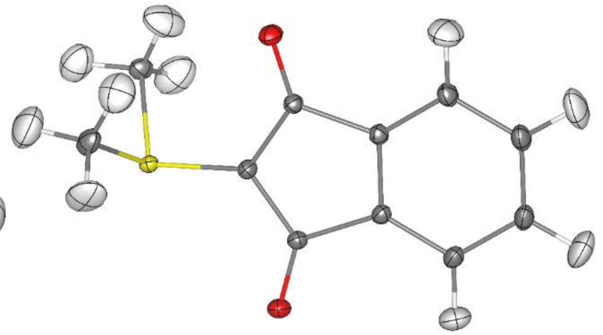

Dataset 8

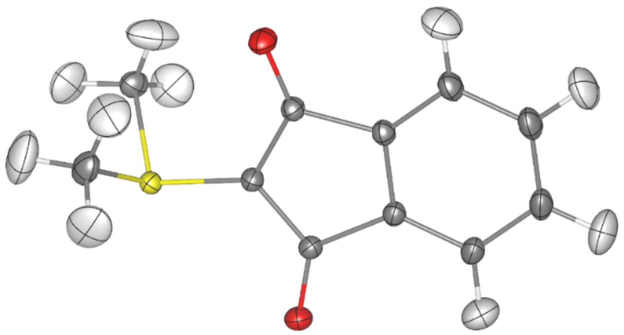

Dataset 9

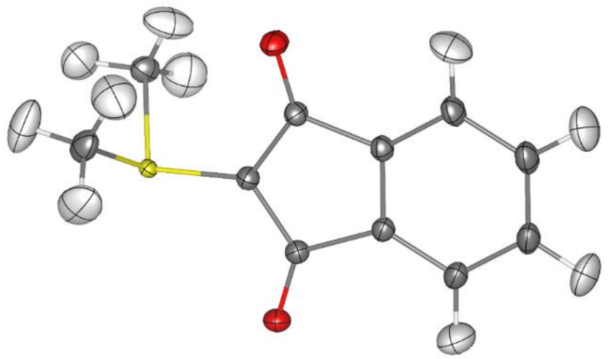

Dataset 10

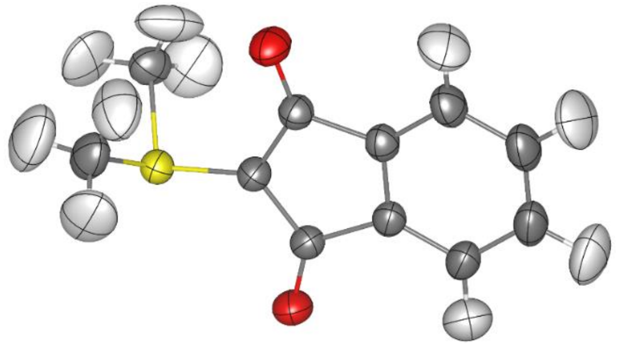

Dataset 11

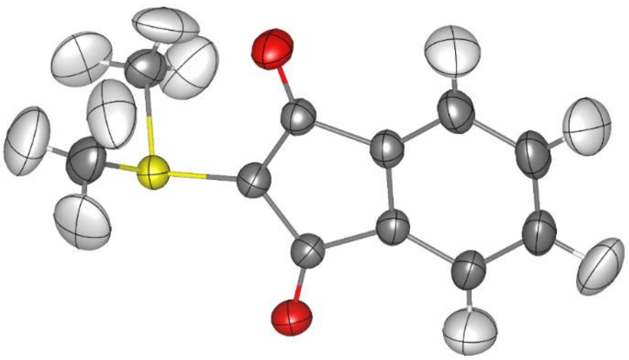

Dataset 12

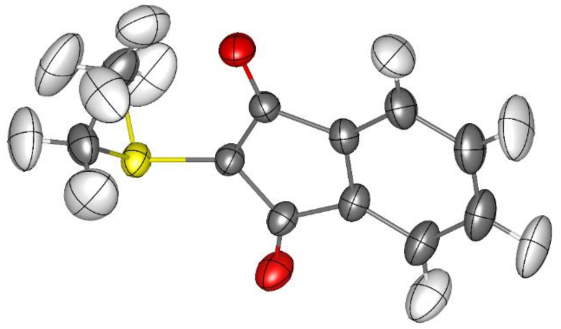

Dataset 13

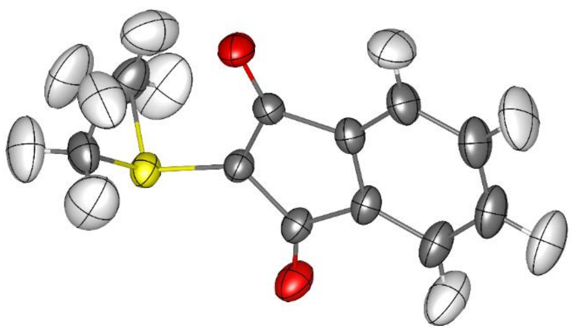

Dataset 14

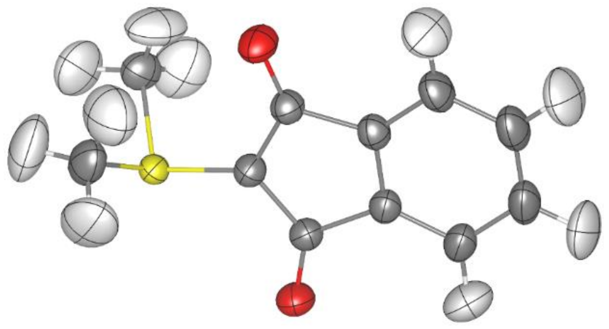

Dataset 15

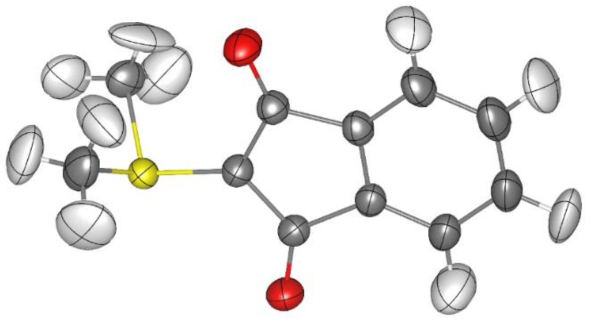

Dataset 16

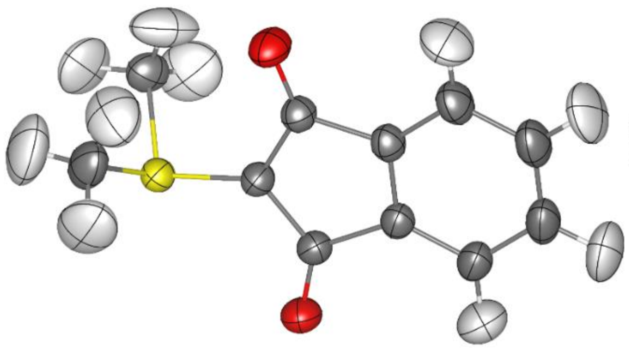

Dataset 17

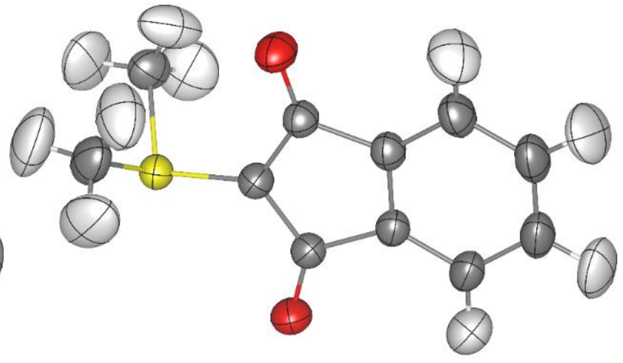

Dataset 18

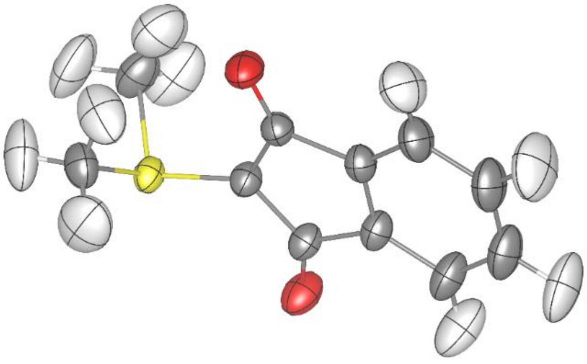

Dataset 19

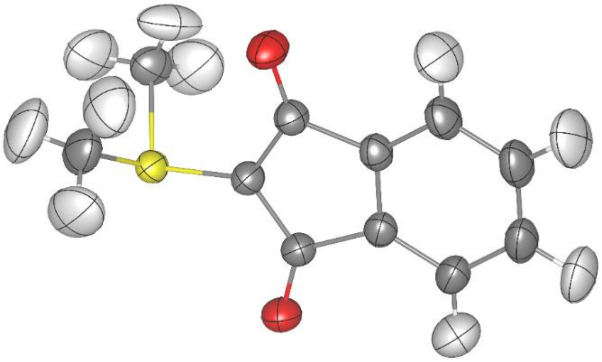

Dataset 20

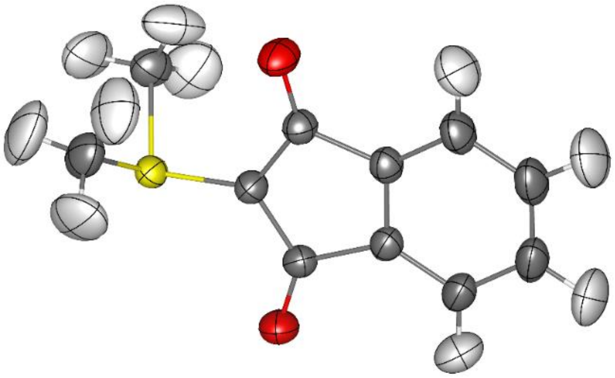

Dataset 21

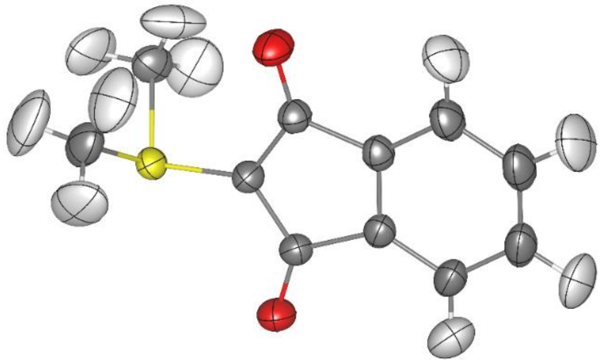

Dataset 22

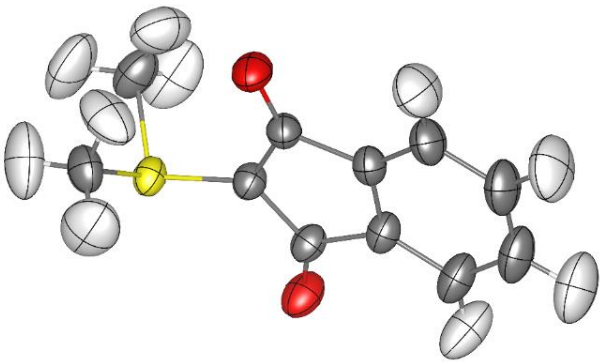

Dataset 23

Figure S7. Chemical structures of YLID with freely refined hydrogen-atom ADPs after HAR. ADPs at a 50% probability level, plotted with the software VESTA.

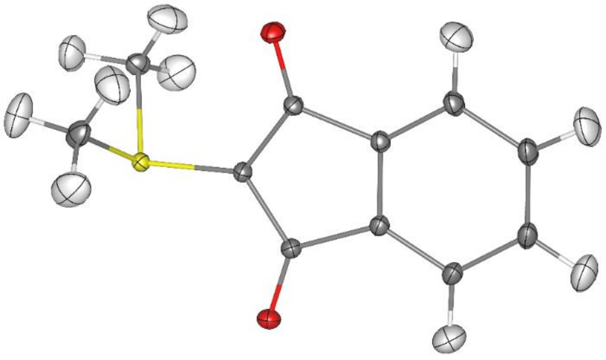

Dataset 1

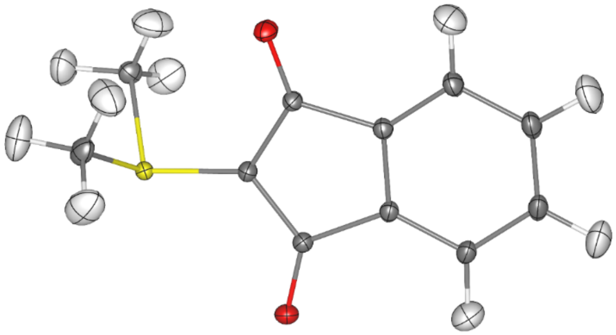

Dataset 3

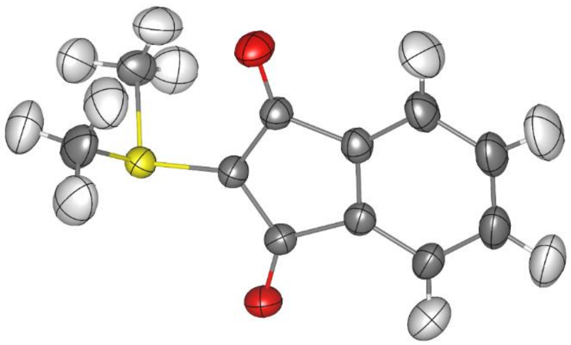

Dataset 16

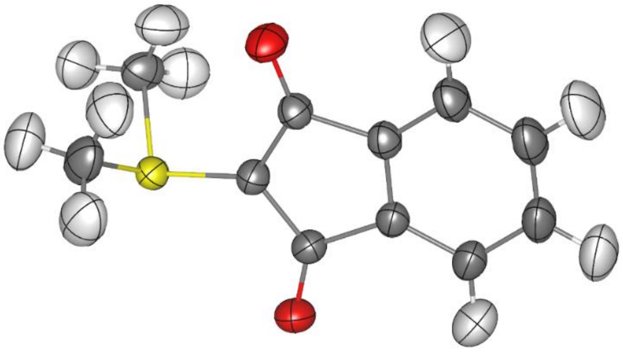

Dataset 17

Figure S8. Chemical structures of YLID after multipole refinement with hydrogen-atom ADPs fixed at values obtained from the SHADE server. ADPs at a 50% probability level, plotted with the software VESTA.

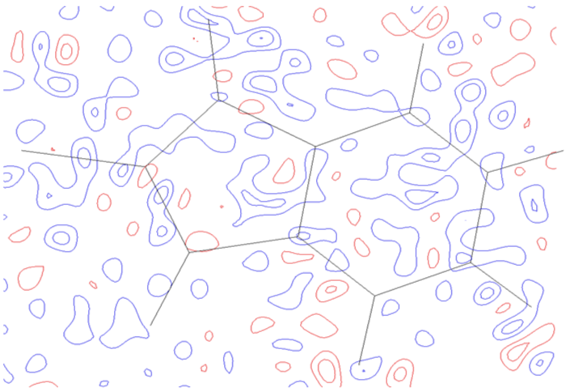

Dataset 1 – MM

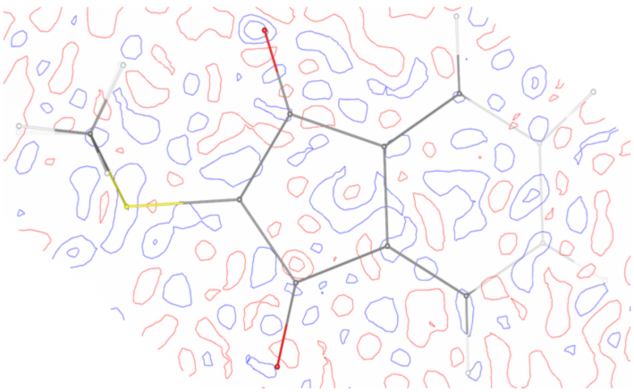

Dataset 1 – XWR

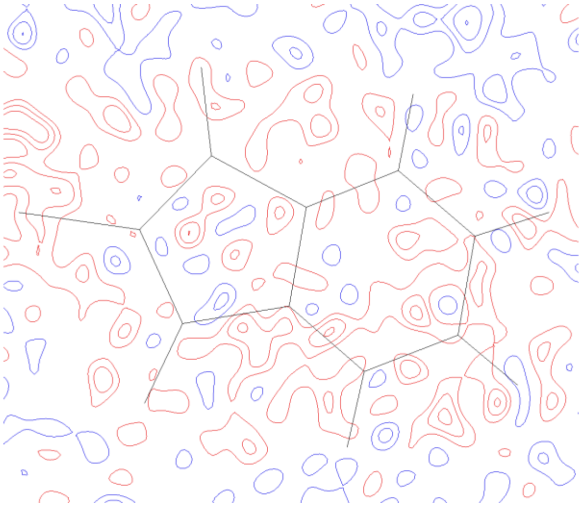

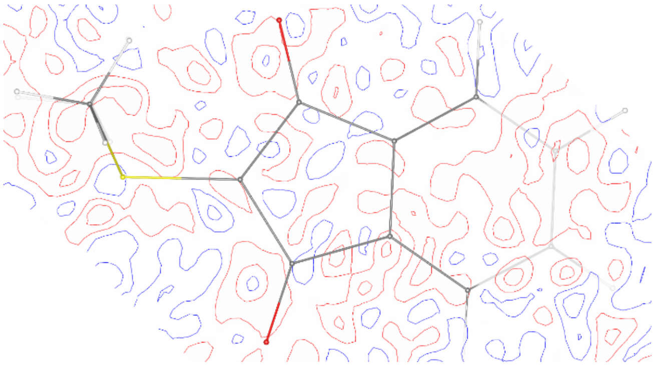

Dataset 3 – MM **Dataset 3 – XWR**

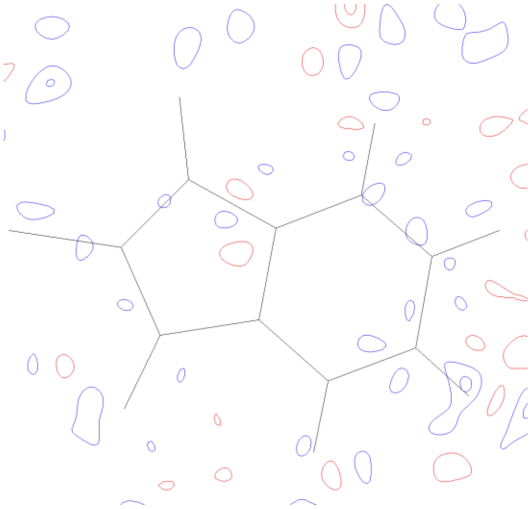

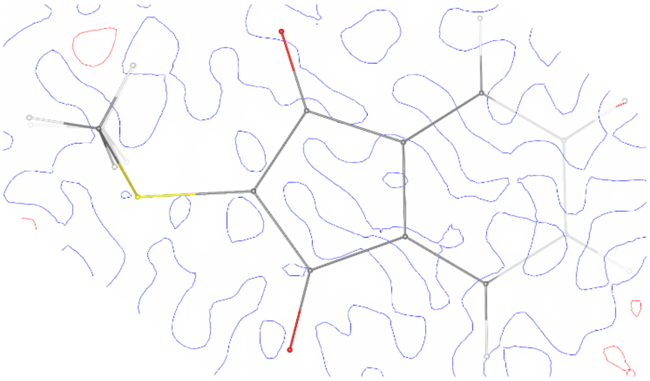

Dataset 16 – MM **Dataset 16 – XWR**

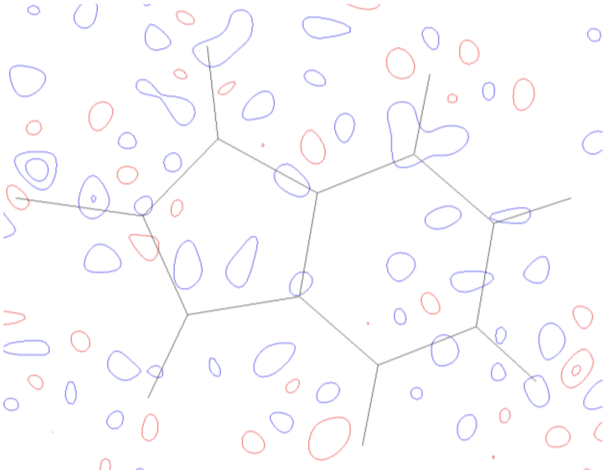

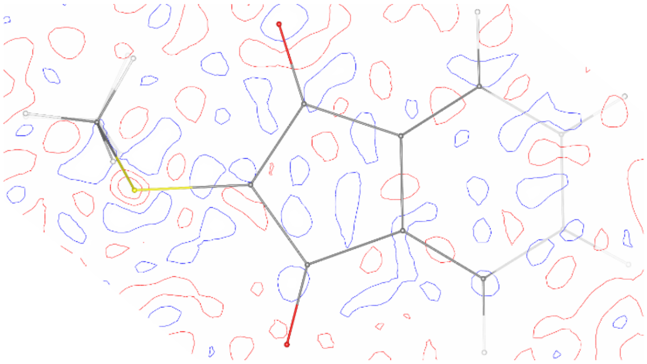

Dataset 17 – MM **Dataset 17 – XWR**

Figure S9. Comparison of 2-D residual density maps between results from MM and XWR depicted using VESTA. Isocontour value = 0.03 eÅ^{-3}. Red = negative, blue = positive.

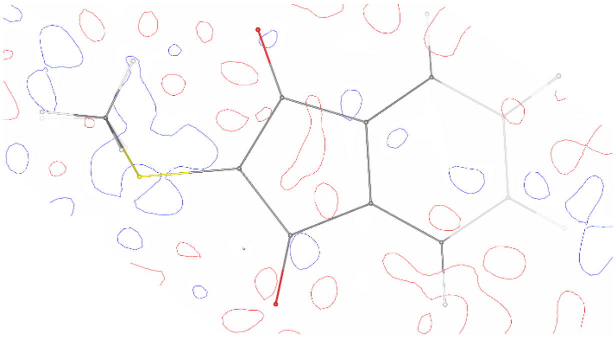

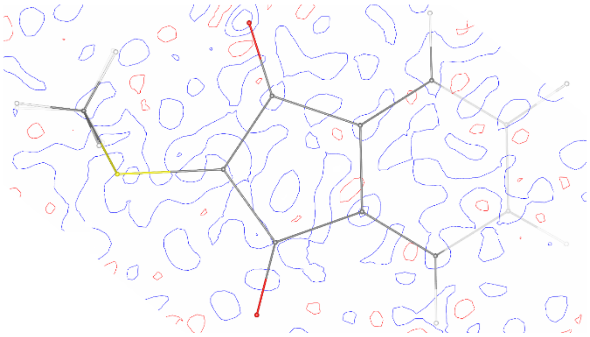

Dataset 2 **Dataset 4**

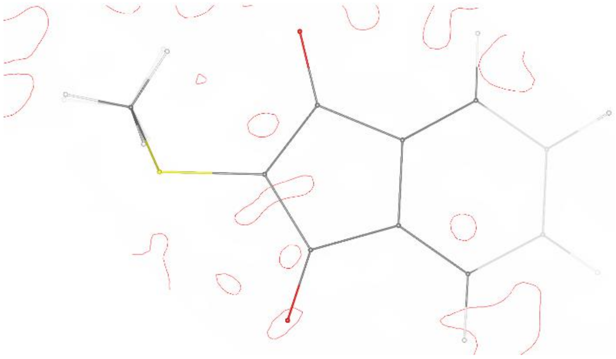

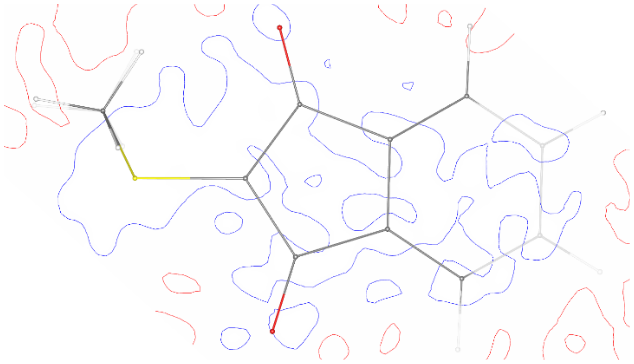

Dataset 5 **Dataset 6**

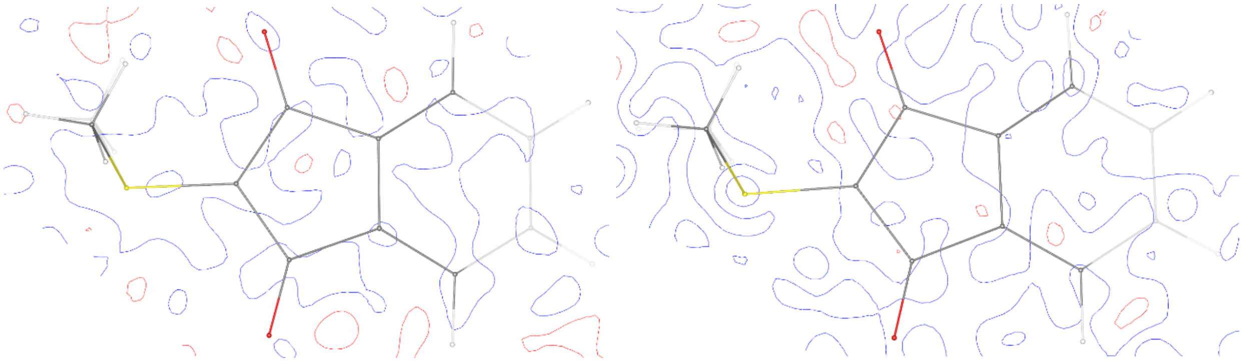

Dataset 7 **Dataset 10**

Figure S10. Selection of 2-D residual density maps for XWR depicted using VESTA. Isocontour value = 0.03 eÅ^{-3}. Red = negative, blue = positive using VESTA software.

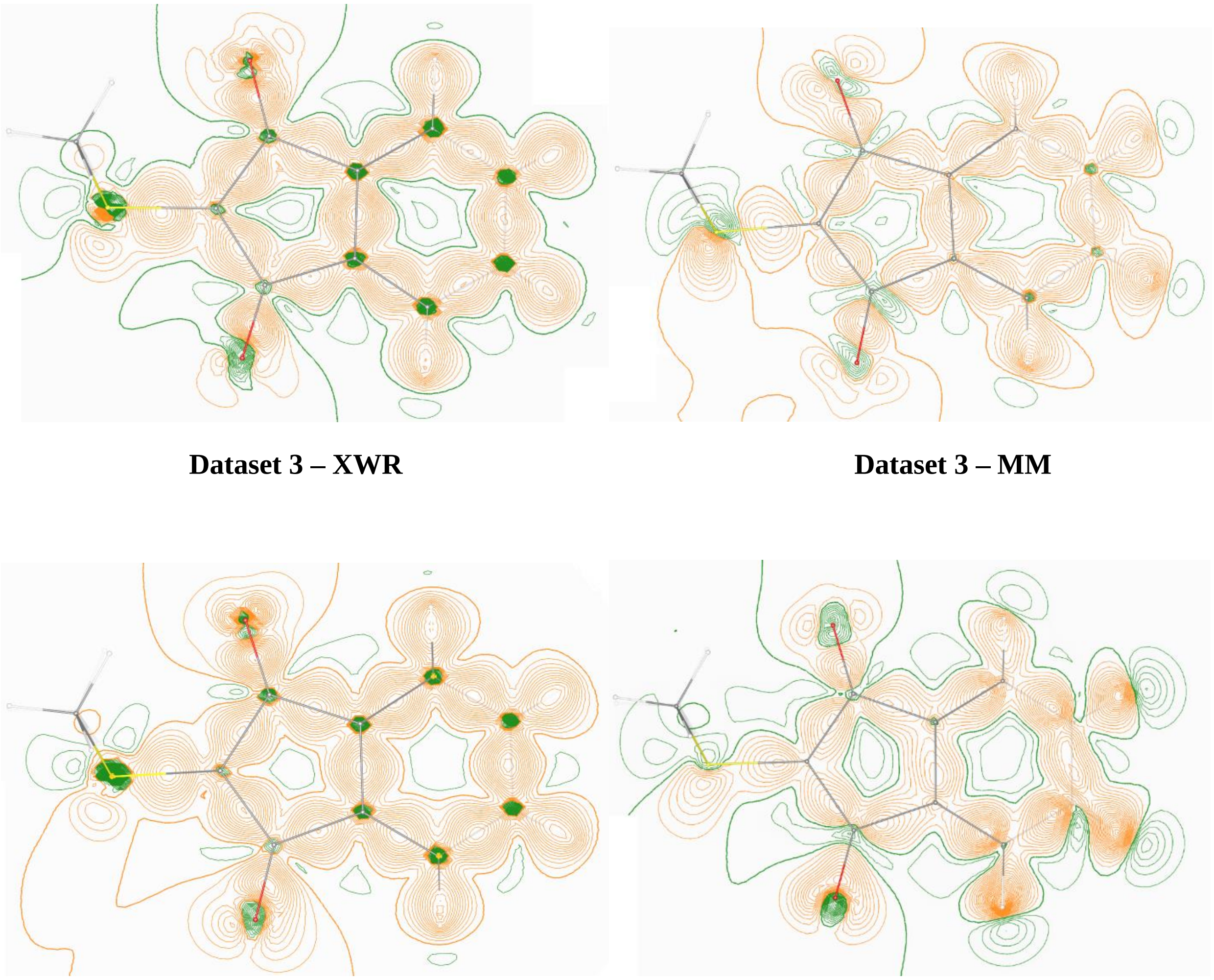

Dataset 3 – XWR **Dataset 3 – MM**

Dataset 16 – XWR **Dataset 16 – MM**

Figure S11. Comparison between 2-D static deformation density maps for XWR and MM depicted using VESTA. Isocontour value = 0.01 eÅ^{-3}. Green = negative, orange = positive. Corresponding representations for datasets 1 and 17 are shown in Figure B in part I of the Supplementary Information.

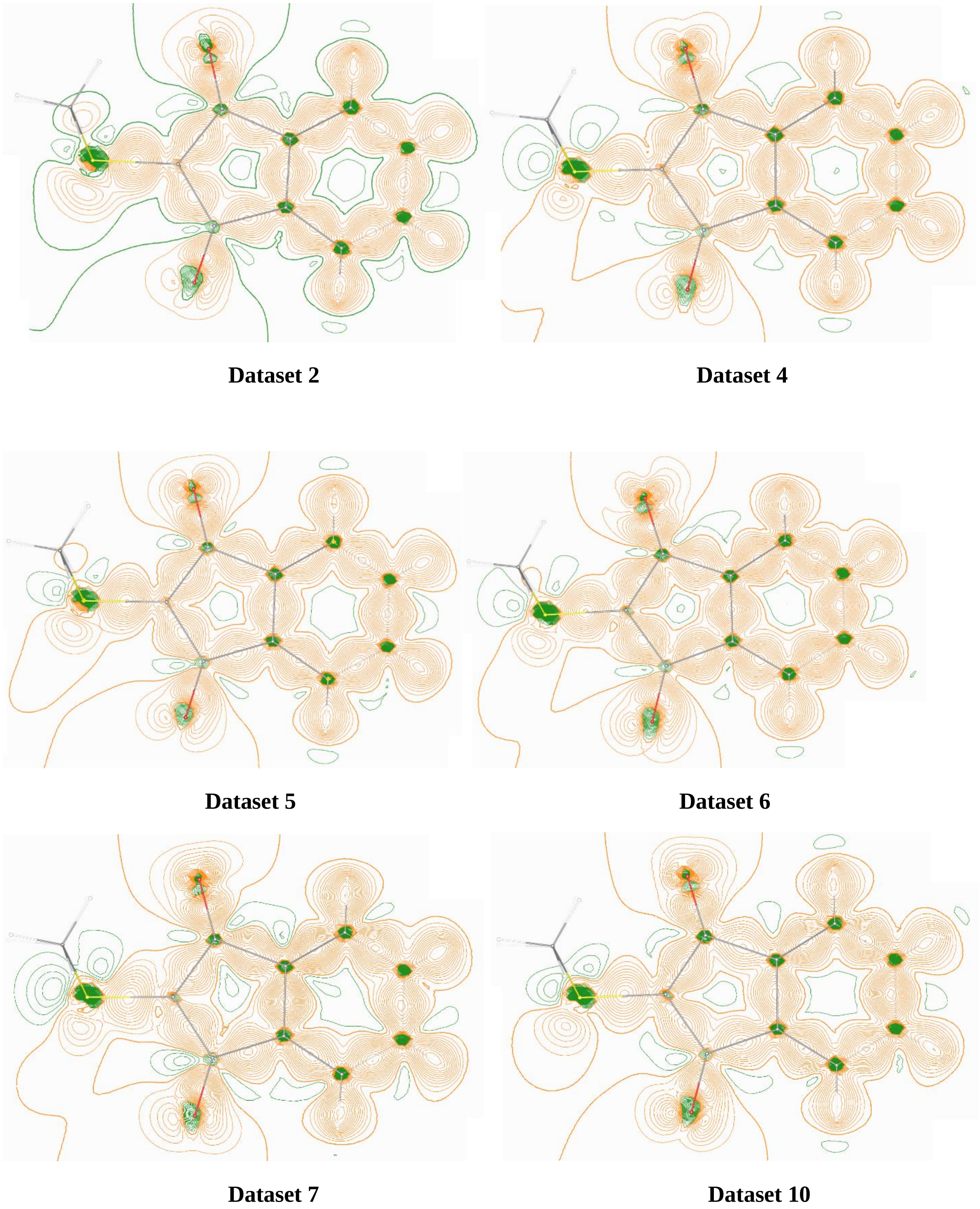

Figure S12. Selection of 2-D deformation density maps for XWR depicted using VESTA. Isocontour value = 0.01 eÅ^{-3}. Green = negative, orange = positive.

Table S7. C-H bond lengths from HAR, their average values and comparison to data from neutron diffraction as well as theoretical geometry optimization. Aromatic bonds: C6-H6, C7-H7, C8-H8, C9-H9; Non aromatic/methyl bonds: C11-H11A, C11-H11B, C11-H11C, C10-H10A, C10-H10B, C10-H10C. AVG: average value; SD: sample standard deviation. All values are in the unit Å. Neutron average bond length values for C-H (aromatic/non-aromatic) and O-H (water) are extracted from Allen & Bruno[4] and Grabowsky et al.[13]. C-H(aromatic)= 1.083(17) Å; C-H(methyl)= 1.077(26) Å; O-H(water)= 0.971(6) Å.

**Dataset**	**1**		**2**		**3**		**4**	
	**Bond length (HAR)**	**Neutron-HAR**	**Bond length (HAR)**	**Neutron-HAR**	**Bond length (HAR)**	**Neutron-HAR**	**Bond length (HAR)**	**Neutron-HAR**
**C9-H9**	1.084	-0.001	1.074	0.009	1.094	-0.011	1.081	0.002
**C8-H8**	1.081	0.002	1.095	-0.012	1.086	-0.003	1.082	0.001
**C7-H7**	1.085	-0.002	1.112	-0.029	1.081	0.002	1.084	-0.001
**C6-H6**	1.085	-0.002	1.092	-0.009	1.098	-0.015	1.084	-0.001
**C10-H10A**	1.078	-0.001	1.080	-0.003	1.098	-0.021	1.080	-0.003
**C10-H10B**	1.090	-0.013	1.099	-0.022	1.088	-0.011	1.083	-0.006
**C10-H10C**	1.082	-0.005	1.101	-0.024	1.077	0.000	1.075	0.002
**C11-H11A**	1.087	-0.010	1.092	-0.015	1.091	-0.014	1.085	-0.008
**C11-H11B**	1.089	-0.012	1.099	-0.022	1.082	-0.005	1.076	0.001
**C11-H11C**	1.082	-0.005	1.079	-0.002	1.090	-0.013	1.074	0.003
**AVG(aromatic)**	1.083	-0.001	1.093	-0.010	1.089	-0.007	1.083	0.001
**AVG (methyl)**	1.085	-0.008	1.091	-0.015	1.087	-0.011	1.079	-0.002
**SD(aromatic)**	0.002	-	0.015	-	0.008	-	0.001	-
**SD (methyl)**	0.005	-	0.009	-	0.007	-	0.004	-
**Dataset**	**5**		**6**		**7**		**8**	
**C9-H9**	1.087	-0.004	1.082	0.001	1.083	0.000	1.093	-0.010
**C8-H8**	1.083	0.00	1.080	0.003	1.081	0.002	1.071	0.012
**C7-H7**	1.078	0.005	1.089	-0.006	1.092	-0.009	1.096	-0.013
**C6-H6**	1.084	-0.001	1.079	0.004	1.085	-0.002	1.097	-0.014
**C10-H10A**	1.078	-0.001	1.086	-0.009	1.092	-0.015	1.094	-0.017
**C10-H10B**	1.087	-0.010	1.083	-0.006	1.076	0.001	1.067	0.010
**C10-H10C**	1.080	-0.003	1.068	0.009	1.078	-0.001	1.099	-0.022
**C11-H11A**	1.080	-0.006	1.084	-0.007	1.091	-0.014	1.056	0.021
**C11-H11B**	1.066	0.011	1.092	-0.015	1.089	-0.012	1.099	-0.022
**C11-H11C**	1.077	0.000	1.072	0.005	1.071	0.006	1.073	0.004
**AVG (aromatic)**	1.083	0.000	1.082	0.001	1.085	-0.002	1.089	-0.006
**AVG (methyl)**	1.083	-0.001	1.080	-0.004	1.082	-0.006	1.081	-0.004
**SD (aromatic)**	0.004	-	0.004	-	0.005	-	0.012	-
**SD (methyl)**	0.007	-	0.009	-	0.009	-	0.018	-
**Dataset**	**9**		**10**		**11**		**12**	
**C9-H9**	1.087	-0.004	1.085	-0.002	1.098	-0.015	1.095	-0.012
**C8-H8**	1.079	0.004	1.085	-0.002	1.087	-0.004	1.108	-0.025
**C7-H7**	1.087	-0.004	1.106	-0.023	1.083	0.000	1.071	0.012
**C6-H6**	1.088	-0.005	1.097	-0.014	1.085	-0.002	1.082	0.001
**C10-H10A**	1.071	0.006	1.075	0.002	1.057	0.020	1.042	0.035
**C10-H10B**	1.087	-0.010	1.092	-0.015	1.089	-0.012	1.110	-0.033
**C10-H10C**	1.076	0.001	1.090	-0.013	1.066	0.011	1.070	0.007
**C11-H11A**	1.090	-0.013	1.096	-0.019	1.080	-0.003	1.076	0.001
**C11-H11B**	1.086	-0.009	1.087	-0.010	1.056	0.021	1.047	0.030
**C11-H11C**	1.080	-0.003	1.086	-0.009	1.090	-0.013	1.078	-0.001

**AVG (aromatic)**	1.085	-0.002	1.093	-0.010	1.088	-0.005	1.089	-0.006
**AVG (methyl)**	1.081	-0.005	1.087	-0.011	1.073	0.004	1.070	0.006
**SD (aromatic)**	0.004	-	0.010	-	0.006	-	0.016	-
**SD (methyl)**	0.007	-	0.007	-	0.015	-	0.024	-
**Dataset**	**13**		**14**		**15**		**16**	
**C9-H9**	1.091	-0.008	1.080	0.003	1.099	-0.016	1.080	0.003
**C8-H8**	1.090	-0.007	1.093	-0.010	1.084	-0.001	1.084	-0.001
**C7-H7**	1.085	-0.002	1.089	-0.006	1.096	-0.013	1.091	-0.008
**C6-H6**	1.092	-0.009	1.090	-0.007	1.104	-0.021	1.108	-0.025
**C10-H10A**	1.062	0.015	1.068	0.009	1.076	0.001	1.039	0.038
**C10-H10B**	1.076	0.001	1.079	-0.002	1.083	-0.006	1.070	0.007
**C10-H10C**	1.059	0.018	1.055	0.022	1.083	-0.006	1.054	0.023
**C11-H11A**	1.073	0.004	1.075	0.002	1.087	-0.010	1.074	0.003
**C11-H11B**	1.051	0.026	1.045	0.032	1.086	-0.009	1.050	0.027
**C11-H11C**	1.069	0.008	1.077	0.000	1.059	0.018	1.066	0.011
**AVG(aromatic)**	1.089	-0.006	1.088	-0.005	1.095	-0.012	1.090	-0.007
**AVG (methyl)**	1.065	0.012	1.066	0.010	1.079	-0.002	1.058	0.018
**SD(aromatic)**	0.003	-	0.005	-	0.008	-	0.012	-
**SD (methyl)**	0.009	-	0.014	-	0.010	-	0.013	-
**Dataset**	**17**		**18**		**19**		**20**	
**C9-H9**	1.085	-0.002	1.108	-0.025	1.094	-0.011	1.091	-0.008
**C8-H8**	1.090	-0.007	1.094	-0.011	1.094	-0.011	1.082	0.001
**C7-H7**	1.098	-0.015	1.081	0.002	1.093	-0.010	1.094	-0.011
**C6-H6**	1.094	-0.011	1.070	0.013	1.083	0.000	1.104	-0.021
**C10-H10A**	1.051	0.026	1.070	0.007	1.025	0.052	1.073	0.004
**C10-H10B**	1.076	0.001	1.076	0.001	1.082	-0.005	1.074	0.003
**C10-H10C**	1.059	0.018	1.054	0.023	1.084	-0.007	1.050	0.027
**C11-H11A**	1.081	-0.004	1.083	-0.006	1.098	-0.021	1.105	-0.028
**C11-H11B**	1.059	0.018	1.064	0.013	1.093	-0.016	1.079	-0.002
**C11-H11C**	1.078	-0.001	1.067	0.010	0.991	0.086	1.019	0.058
**AVG(aromatic)**	1.091	-0.008	1.088	-0.005	1.091	-0.008	1.092	-0.009
**AVG (methyl)**	1.067	0.009	1.069	0.008	1.062	0.014	1.066	0.010
**SD(aromatic)**	0.005	-	0.016	-	0.005	-	0.009	-
**SD (methyl)**	0.012	-	0.010	-	0.043	-	0.029	-
**Dataset**	**21**		**22**		**23**		Geometry optimized	
**C9-H9**	1.080	0.003	1.060	0.023	1.090	-0.007	1.082	
**C8-H8**	1.081	0.002	1.091	-0.008	1.089	-0.006	1.082	
**C7-H7**	1.095	-0.012	1.102	-0.019	1.094	-0.011	1.082	
**C6-H6**	1.101	-0.018	1.096	-0.013	1.073	0.010	1.082	
**C10-H10A**	1.056	0.021	1.062	0.015	1.065	0.012	1.088	
**C10-H10B**	1.077	0.000	1.068	0.009	1.060	0.017	1.087	
**C10-H10C**	1.086	-0.009	1.060	0.017	1.059	0.018	1.089	
**C11-H11A**	1.086	-0.009	1.064	0.013	1.101	-0.024	1.087	
**C11-H11B**	1.084	-0.007	1.101	-0.024	1.057	0.020	1.089	
**C11-H11C**	1.040	0.037	1.031	0.046	1.060	0.017	1.088	
**AVG (aromatic)**	1.089	-0.006	1.087	-0.004	1.086	-0.003		
**AVG (methyl)**	1.071	0.005	1.064	0.013	1.067	0.010		
**SD (aromatic)**	0.010	-	0.018	-	0.009	-		
**SD (methyl)**	0.019	-	0.022	-	0.016	-		

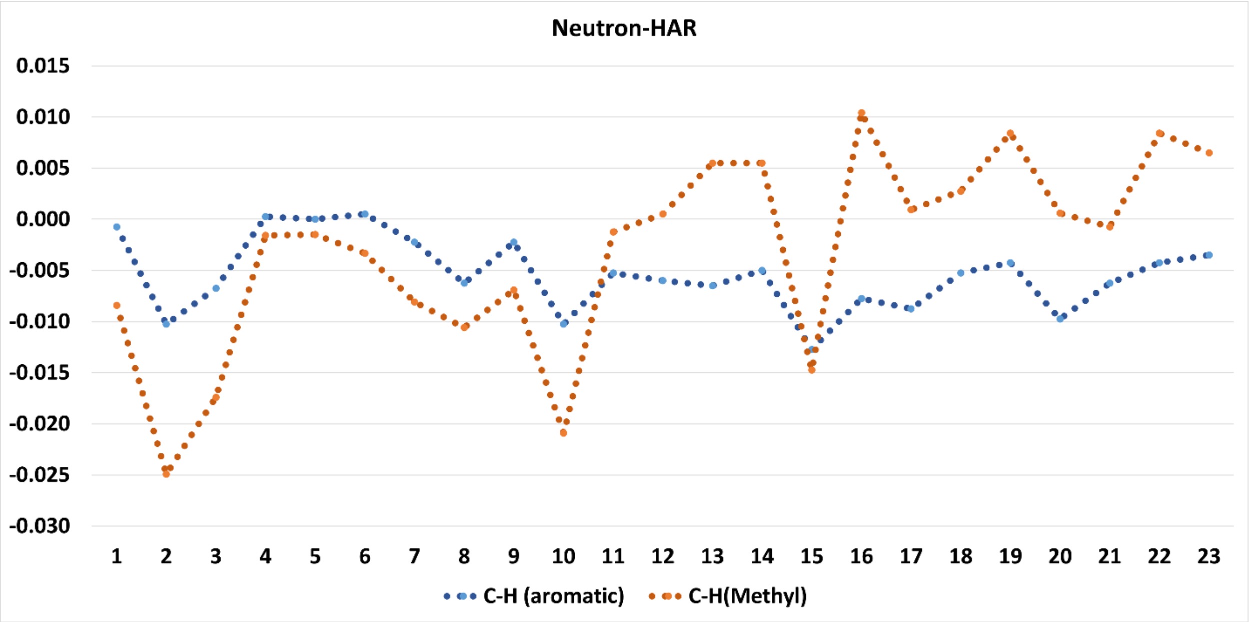

(a)

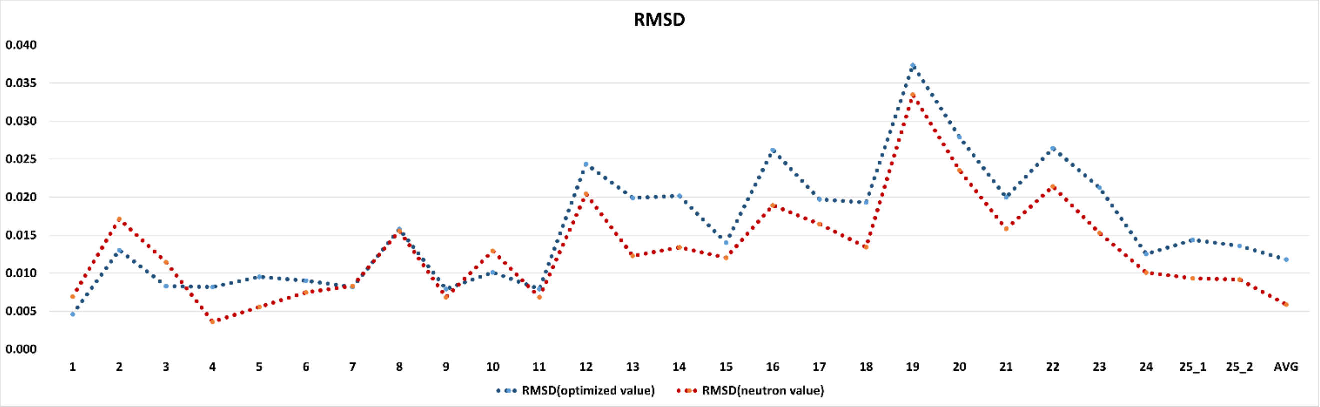

(b)

Figure S13. (a) Average difference of the HAR-refined C-H bond lengths to tabulated values from neutron diffraction for C-H (aromatic)/C-H(methyl) for each data set (according to Table S7), order: neutron minus HAR; **(b)** root-mean square deviation of the HAR-refined C-H bond lengths compared to values from neutron diffraction and isolated-molecule optimization for all 25 datasets (including other modifications)

Table S8. Topological properties of electron density at bond critical points related to multipole refinement

**Dataset number**	**Bond**	**Electron Density (ρ)** in e/Å^3	**Laplacian of Electron Density ($\nabla^2\rho$)** in e/Å^5	**Ellipticity (σ)**
**Dataset 1**	O(1)-C(2)	2.77(4)	-30.4(3)	0.05
	O(2)-C(5)	2.82(4)	-31.9(2)	0.02
	C(2)-C(3)	1.76(2)	-12.2(1)	0.09
	C(2)-C(1)	1.97(2)	-15.9(3)	0.20
	C(3)-C(4)	2.11(2)	-18.9(1)	0.07
	C(4)-C(5)	1.77(2)	-13.1(2)	0.13
	C(5)-C(1)	1.98(2)	-17.4(1)	0.26
	C(3)-C(6)	2.18(3)	-20.9(1)	0.17
	C(4)-C(9)	2.18(3)	-21.1(1)	0.11
	C(9)-C(8)	2.12(3)	-19.3(1)	0.15
	C(7)-C(8)	2.07(3)	-17.5(1)	0.21
	C(6)-C(7)	2.14(3)	-20.2(1)	0.16
	C(1)-S(1)	1.48(6)	-7.4(1)	0.01
	C(11)-S(1)	1.30(3)	-5.7(6)	0.07
	C(10)-S(1)	1.33(3)	-6.7(3)	0.05
	C(9)-H(9)	1.95(7)	-20.9(1)	0.13
	C(8)-H(8)	1.96(7)	-20.5(2)	0.05
	C(7)-H(7)	1.85(6)	-16.9(1)	0.05
	C(6)-H(6)	1.85(6)	-18.1(1)	0.07
	C(10)-H(10A)	1.74(7)	-18.2(2)	0.04
	C(10)-H(10B)	1.93(7)	-19.6(2)	0.08
	C(10)-H(10C)	2.05(7)	-24.7(1)	0.08
	C(11)-H(11A)	1.99(7)	-22.9(1)	0.10
	C(11)-H(11B)	1.88(7)	-19.6(1)	0.02
	C(11)-H(11C)	1.75(8)	-18.9(3)	0.08
**Dataset 3**	O(1)-C(2)	2.80(12)	-31.7(8)	0.12
	O(2)-C(5)	2.91(12)	-33.3(8)	0.06
	C(2)-C(3)	1.79(5)	-15.1(1)	0.21
	C(2)-C(1)	2.06(6)	-23.3(2)	0.29
	C(3)-C(4)	2.08(4)	-18.7(1)	0.05
	C(4)-C(5)	1.84(5)	-15.9(1)	0.20
	C(5)-C(1)	1.99(5)	-22.3(1)	0.10
	C(3)-C(6)	2.14(5)	-19.5(1)	0.35
	C(4)-C(9)	2.14(6)	-20.8(2)	0.12
	C(9)-C(8)	2.02(5)	-18.4(1)	0.35
	C(7)-C(8)	2.14(4)	-21.8(1)	0.24
	C(6)-C(7)	2.15(5)	-23.3(1)	0.13
	C(1)-S(1)	1.50(12)	-8.6(2)	0.31
	C(11)-S(1)	1.29(6)	-5.5(1)	0.15
	C(10)-S(1)	1.23(6)	-5.6(1)	0.16
	C(9)-H(9)	1.86(11)	-15.9(2)	0.15
	C(8)-H(8)	1.72(9)	-15.4(2)	0.39

	C(7)-H(7)	1.92(10)	-20.8(2)	0.08
	C(6)-H(6)	1.76(11)	-14.7(3)	0.08
	C(10)-H(10A)	1.67(12)	-16.5(4)	0.05
	C(10)-H(10B)	1.92(9)	-18.6(3)	0.27
	C(10)-H(10C)	2.03(11)	-22.7(3)	0.05
	C(11)-H(11A)	1.90(10)	-19.0(3)	0.24
	C(11)-H(11B)	1.83(10)	-18.5(3)	0.21
	C(11)-H(11C)	1.79(12)	-15.5(4)	0.13
**Dataset 16**	O(1)-C(2)	2.69(27)	-26.5(8)	0.09
	O(2)-C(5)	2.82(29)	-26.2(8)	0.37
	C(2)-C(3)	1.89(6)	-17.4(2)	0.24
	C(2)-C(1)	1.86(6)	-12.6(3)	0.52
	C(3)-C(4)	2.07(9)	-14.3(4)	0.13
	C(4)-C(5)	1.80(5)	-12.1(1)	0.11
	C(5)-C(1)	1.99(5)	-15.4(1)	0.29
	C(3)-C(6)	2.08(10)	-15.2(3)	0.28
	C(4)-C(9)	2.13(14)	-13.2(6)	0.38
	C(9)-C(8)	2.20(15)	-20.7(4)	0.14
	C(7)-C(8)	2.03(5)	-18.8(1)	0.02
	C(6)-C(7)	2.12(6)	-21.4(2)	0.21
	C(1)-S(1)	1.47(4)	-6.2(1)	0.14
	C(11)-S(1)	1.27(7)	-0.9(1)	0.09
	C(10)-S(1)	1.32(2)	-6.8(5)	0.14
	C(9)-H(9)	2.13(12)	-24.9(3)	0.25
	C(8)-H(8)	1.98(8)	-18.1(2)	0.35
	C(7)-H(7)	1.89(1)	-21.4(2)	0.10
	C(6)-H(6)	1.79(1)	-17.9(3)	0.13
	C(10)-H(10A)	1.74(1)	-14.6(3)	0.18
	C(10)-H(10B)	1.57(8)	-6.7(1)	0.51
	C(10)-H(10C)	1.88(10)	-18.4(1)	0.09
	C(11)-H(11A)	1.94(9)	-18.1(1)	0.22
	C(11)-H(11B)	1.83(7)	-17.5(1)	0.13
	C(11)-H(11C)	1.73(12)	-19.7(4)	0.26
**Dataset 17**	O(1)-C(2)	2.84(9)	-35.1(6)	0.10
	O(2)-C(5)	2.68(10)	-19.9(7)	0.13
	C(2)-C(3)	1.79(3)	-12.3(1)	0.12
	C(2)-C(1)	1.95(3)	-14.7(1)	0.17
	C(3)-C(4)	1.97(1)	-12.2(2)	0.15
	C(4)-C(5)	1.77(2)	-11.1(0)	0.09
	C(5)-C(1)	1.92(3)	-12.9(1)	0.27
	C(3)-C(6)	2.09(3)	-15.9(0)	0.16
	C(4)-C(9)	1.98(3)	-12.3(1)	0.11
	C(9)-C(8)	2.05(5)	-18.1(2)	0.17
	C(7)-C(8)	2.07(5)	-17.1(1)	0.15
	C(6)-C(7)	2.06(4)	-17.6(2)	0.28
	C(1)-S(1)	1.47(2)	-8.1(4)	0.02
	C(11)-S(1)	1.35(1)	-8.3(3)	0.14

	C(10)-S(1)	1.33(1)	-6.9(2)	0.06
	C(9)-H(9)	2.08(4)	-26.6(1)	0.25
	C(8)-H(8)	2.18(4)	-33.1(1)	0.11
	C(7)-H(7)	1.92(4)	-20.8(1)	0.12
	C(6)-H(6)	1.72(4)	-15.6(1)	0.06
	C(10)-H(10A)	1.81(4)	-18.1(2)	0.12
	C(10)-H(10B)	1.69(4)	-11.6(9)	0.31
	C(10)-H(10C)	1.91(4)	-19.1(0)	0.11
	C(11)-H(11A)	1.85(3)	-15.2(1)	0.43
	C(11)-H(11B)	1.80(4)	-16.5(1)	0.18
	C(11)-H(11C)	1.71(5)	-15.7(2)	0.14

Table S9. Topological properties of electron density at bond critical points related to XWR refinement and isolated-molecule DFT calculation.

**Dataset number**	**Bond**	**Electron Density (ρ)** in e/Å^3	**Laplacian of Electron Density ($\nabla^2\rho$)** in e/Å^5	**Ellipticity (σ)**
**Dataset 1**	O(1)-C(2)	2.68	-4.3	0.03
	O(2)-C(5)	2.71	-1.3	0.06
	C(2)-C(3)	1.83	-18.5	0.06
	C(2)-C(1)	2.03	-21.5	0.22
	C(3)-C(4)	2.20	-25.1	0.16
	C(4)-C(5)	1.84	-19.2	0.07
	C(5)-C(1)	2.04	-22.8	0.22
	C(3)-C(6)	2.23	-25.9	0.18
	C(9)-C(4)	2.22	-25.7	0.19
	C(6)-C(7)	2.14	-24.1	0.18
	C(7)-C(8)	2.16	-24.1	0.22
	C(8)-C(9)	2.16	-24.6	0.18
	C(1)-S(1)	1.54	-12.2	0.12
	C(11)-S(1)	1.34	-9.1	0.05
	C(10)-S(1)	1.35	-9.6	0.02
	C(9)-H(9)	1.95	-25.7	0.02
	C(8)-H(8)	1.95	-25.8	0.02
	C(7)-H(7)	1.96	-26.1	0.01
	C(6)-H(6)	1.94	-25.8	0.03
	C(10)-H(10A)	1.91	-24.9	0.01
	C(10)-H(10B)	1.96	-26.1	0.03
	C(10)-H(10C)	1.98	-26.1	0.01
	C(11)-H(11A)	1.93	-25.1	0.01
	C(11)-H(11B)	1.90	-25.4	0.02
	C(11)-H(11C)	1.94	-24.3	0.01

Dataset number	Bond	Electron Density (ρ) in e/Å^3	Laplacian of Electron Density ($\nabla^2\rho$) in e/Å^5	Ellipticity (σ)
**Dataset 2**	O(1)-C(2)	2.72	-9.5	0.09
	O(2)-C(5)	2.74	-7.6	0.02
	C(2)-C(3)	1.76	-17.1	0.12
	C(2)-C(1)	2.01	-20.4	0.23
	C(3)-C(4)	2.18	-24.2	0.13
	C(4)-C(5)	1.76	-16.7	0.13
	C(5)-C(1)	1.96	-19.1	0.23
	C(3)-C(6)	2.18	24.1	0.16
	C(9)-C(4)	2.20	-25.1	0.16
	C(6)-C(7)	2.12	-23.3	0.20
	C(7)-C(8)	2.11	-21.3	0.27
	C(8)-C(9)	2.15	-24.5	0.17
	C(1)-S(1)	1.54	-12.7	0.11
	C(11)-S(1)	1.35	-9.9	0.05
	C(10)-S(1)	1.36	-10.1	0.16
	C(9)-H(9)	1.97	-26.4	0.02
	C(8)-H(8)	1.90	-24.9	0.02
	C(7)-H(7)	1.86	-24.6	0.02
	C(6)-H(6)	1.94	-25.9	0.02
	C(10)-H(10A)	1.90	-25.2	0.01
	C(10)-H(10B)	1.91	-25.1	0.02
	C(10)-H(10C)	1.99	-25.2	0.02
	C(11)-H(11A)	1.99	-26.6	0.03
	C(11)-H(11B)	1.93	-24.8	0.01
	C(11)-H(11C)	1.89	-26.4	0.02
**Dataset number**	**Bond**	**Electron Density (ρ)** in e/Å^3	**Laplacian of Electron Density ($\nabla^2\rho$)** in e/Å^5	**Ellipticity (σ)**
**Dataset 3**	O(1)-C(2)	2.75	-10.5	0.06
	O(2)-C(5)	2.81	-3.4	0.12
	C(2)-C(3)	1.80	-17.2	0.15
	C(2)-C(1)	2.14	-26.6	0.16
	C(3)-C(4)	2.24	-27.1	0.07
	C(4)-C(5)	1.84	-18.6	0.11
	C(5)-C(1)	2.01	-21.8	0.12
	C(3)-C(6)	2.20	-23.6	0.33
	C(9)-C(4)	2.21	-24.6	0.18
	C(6)-C(7)	2.13	-23.9	0.15
	C(7)-C(8)	2.16	-23.9	0.18

	C(8)-C(9)	2.08	-20.8	0.25
	C(1)-S(1)	1.56	-12.5	0.14
	C(11)-S(1)	1.34	-9.1	0.09
	C(10)-S(1)	1.28	-8.1	0.01
	C(9)-H(9)	1.91	-24.5	0.02
	C(8)-H(8)	1.94	-25.6	0.05
	C(7)-H(7)	1.98	-27.1	0.01
	C(6)-H(6)	1.90	-25.1	0.03
	C(10)-H(10A)	1.92	-24.3	0.04
	C(10)-H(10B)	1.97	-25.3	0.01
	C(10)-H(10C)	1.90	-24.8	0.01
	C(11)-H(11A)	1.91	-23.9	0.02
	C(11)-H(11B)	1.89	-24.1	0.04
	C(11)-H(11C)	1.91	-24.7	0.01
**Dataset number**	**Bond**	**Electron Density (ρ)** in e/Å^3	**Laplacian of Electron Density ($\nabla^2\rho$)** in e/Å^5	**Ellipticity (σ)**
**Dataset 4**	O(1)-C(2)	2.73	-1.6	0.06
	O(2)-C(5)	2.72	-0.1	0.06
	C(2)-C(1)	2.03	-21.6	0.23
	C(2)-C(3)	1.82	-18.6	0.07
	C(3)-C(4)	2.18	-24.6	0.18
	C(4)-C(5)	1.82	-18.4	0.08
	C(5)-C(1)	2.01	-21.5	0.23
	C(3)-C(6)	2.22	-25.5	0.19
	C(6)-C(7)	2.13	-23.4	0.19
	C(7)-C(8)	2.16	-24.5	0.20
	C(8)-C(9)	2.15	-24.1	0.18
	C(9)-C(4)	2.22	-25.5	0.18
	C(1)-S(1)	1.53	-12.3	0.12
	C(10)-S(1)	1.36	-9.7	0.01
	C(11)-S(1)	1.33	-9.4	0.05
	C(6)-H(6)	1.95	-26.0	0.01
	C(7)-H(7)	1.92	-25.4	0.02
	C(8)-H(8)	1.96	-26.4	0.01
	C(9)-H(9)	1.95	-25.9	0.01
	C(10)-H(10A)	1.92	-25.2	0.01
	C(10)-H(10B)	1.97	-25.6	0.01
	C(10)-H(10C)	1.96	-26.1	0.01
	C(11)-H(11A)	1.96	-24.7	0.01
	C(11)-H(11B)	1.92	-24.7	0.02
	C(11)-H(11C)	1.92	-25.9	0.01

Dataset number	Bond	Electron Density (ρ) in e/Å^3	Laplacian of Electron Density ($\nabla^2\rho$) in e/Å^5	Ellipticity (σ)
**Dataset 5**	O(1)-C(2)	2.72	-7.4	0.09
	O(2)-C(5)	2.76	-13.9	0.04
	C(2)-C(3)	1.83	-18.8	0.07
	C(2)-C(1)	2.12	-24.6	0.22
	C(3)-C(4)	2.24	-27.4	0.18
	C(4)-C(5)	1.90	-21.0	0.05
	C(5)-C(1)	2.02	-21.8	0.20
	C(3)-C(6)	2.33	-30.1	0.18
	C(9)-C(4)	2.23	-25.7	0.16
	C(6)-C(7)	2.12	-22.9	0.14
	C(7)-C(8)	2.22	-26.5	0.20
	C(8)-C(9)	2.23	-27.2	0.17
	C(1)-S(1)	1.62	-14.4	0.06
	C(11)-S(1)	1.37	-10.2	0.07
	C(10)-S(1)	1.33	-8.8	0.05
	C(9)-H(9)	1.93	-25.4	0.02
	C(8)-H(8)	1.93	-25.6	0.01
	C(7)-H(7)	1.99	-27.1	0.01
	C(6)-H(6)	1.94	-25.4	0.03
	C(10)-H(10A)	1.92	-25.1	0.03
	C(10)-H(10B)	2.02	-26.4	0.01
	C(10)-H(10C)	1.98	-27.2	0.03
	C(11)-H(11A)	1.92	-24.3	0.02
	C(11)-H(11B)	1.95	-24.3	0.03
	C(11)-H(11C)	1.97	-26.3	0.01
**Dataset number**	**Bond**	**Electron Density (ρ)** in e/Å^3	**Laplacian of Electron Density ($\nabla^2\rho$)** in e/Å^5	**Ellipticity (σ)**
**Dataset 6**	O(1)-C(2)	2.75	-5.7	0.09
	O(2)-C(5)	2.71	-6.1	0.08
	C(2)-C(3)	1.84	-18.4	0.07
	C(2)-C(1)	2.05	-21.6	0.25
	C(3)-C(4)	2.20	-24.8	0.19
	C(4)-C(5)	1.79	-17.9	0.09
	C(5)-C(1)	2.01	-20.9	0.27
	C(3)-C(6)	2.22	-25.1	0.21
	C(9)-C(4)	2.17	-23.5	0.20
	C(6)-C(7)	2.16	-23.6	0.32
	C(7)-C(8)	2.20	-25.7	0.21

	C(8)-C(9)	2.11	-22.6	0.20
	C(1)-S(1)	1.55	-13.3	0.11
	C(11)-S(1)	1.33	-9.9	0.11
	C(10)-S(1)	1.36	-10.0	0.05
	C(9)-H(9)	1.97	-26.2	0.00
	C(8)-H(8)	1.96	-25.9	0.03
	C(7)-H(7)	1.92	-25.9	0.01
	C(6)-H(6)	2.01	-27.7	0.01
	C(10)-H(10A)	1.90	-24.6	0.03
	C(10)-H(10B)	1.99	-25.8	0.01
	C(10)-H(10C)	1.98	-26.3	0.01
	C(11)-H(11A)	1.89	-25.8	0.03
	C(11)-H(11B)	1.88	-23.7	0.01
	C(11)-H(11C)	1.97	-23.2	0.03
**Dataset number**	**Bond**	**Electron Density (ρ)** in e/Å^3	**Laplacian of Electron Density** **($\nabla^2\rho$)** in e/Å^5	**Ellipticity (σ)**
**Dataset 7**	O(1)-C(2)	2.73	-6.1	0.10
	O(2)-C(5)	2.78	-2.8	0.04
	C(2)-C(3)	1.80	-17.9	0.12
	C(2)-C(1)	2.03	-20.7	0.28
	C(3)-C(4)	2.19	-24.7	0.17
	C(4)-C(5)	1.78	-17.3	0.09
	C(5)-C(1)	1.97	-19.9	0.23
	C(3)-C(6)	2.19	-23.5	0.23
	C(9)-C(4)	2.16	-23.2	0.21
	C(6)-C(7)	2.11	-22.6	0.20
	C(7)-C(8)	2.15	-24.0	0.22
	C(8)-C(9)	2.14	-22.9	0.18
	C(1)-S(1)	1.52	-13.0	0.16
	C(11)-S(1)	1.34	-10.7	0.10
	C(10)-S(1)	1.35	-10.1	0.08
	C(9)-H(9)	1.94	-26.2	0.01
	C(8)-H(8)	1.93	-25.2	0.02
	C(7)-H(7)	1.89	-24.5	0.01
	C(6)-H(6)	1.97	-26.7	0.03
	C(10)-H(10A)	1.97	-26.1	0.04
	C(10)-H(10B)	1.94	-24.3	0.01
	C(10)-H(10C)	1.93	-25.6	0.02
	C(11)-H(11A)	1.86	-22.3	0.02
	C(11)-H(11B)	1.89	-23.4	0.02
	C(11)-H(11C)	1.99	-26.6	0.02

**Dataset number**	**Bond**	**Electron Density (ρ)** in e/Å^3	**Laplacian of Electron Density ($\nabla^2\rho$)** in e/Å^5	**Ellipticity (σ)**
**Dataset 8**	O(1)-C(2)	2.79	-11.4	0.04
	O(2)-C(5)	2.64	-13.8	0.06
	C(2)-C(3)	1.82	-18.6	0.11
	C(2)-C(1)	2.04	-21.2	0.26
	C(3)-C(4)	2.10	-21.1	0.13
	C(4)-C(5)	1.92	-20.2	0.15
	C(5)-C(1)	1.93	-18.8	0.21
	C(3)-C(6)	2.30	-28.3	0.25
	C(9)-C(4)	2.18	-24.3	0.24
	C(6)-C(7)	2.12	-23.4	0.16
	C(7)-C(8)	2.14	-23.8	0.14
	C(8)-C(9)	2.21	-25.6	0.27
	C(1)-S(1)	1.56	-11.5	0.26
	C(11)-S(1)	1.38	-9.4	0.12
	C(10)-S(1)	1.06	-3.7	0.53
	C(9)-H(9)	1.91	-25.3	0.03
	C(8)-H(8)	1.96	-25.9	0.02
	C(7)-H(7)	1.98	-27.5	0.06
	C(6)-H(6)	1.92	-25.8	0.01
	C(10)-H(10A)	2.02	-27.3	0.03
	C(10)-H(10B)	1.94	-25.8	0.05
	C(10)-H(10C)	2.02	-27.9	0.03
	C(11)-H(11A)	1.90	-23.1	0.02
	C(11)-H(11B)	1.87	-23.1	0.01
	C(11)-H(11C)	1.96	-26.2	0.01
**Dataset number**	**Bond**	**Electron Density (ρ)** in e/Å^3	**Laplacian of Electron Density ($\nabla^2\rho$)** in e/Å^5	**Ellipticity (σ)**
**Dataset 9**	O(1)-C(2)	2.69	-2.1	0.04
	O(2)-C(5)	2.72	-1.2	0.08
	C(2)-C(3)	1.84	-19.1	0.09
	C(2)-C(1)	2.04	-21.8	0.21
	C(3)-C(4)	2.19	-24.9	0.19
	C(4)-C(5)	1.83	-18.8	0.08
	C(5)-C(1)	2.03	-21.9	0.25
	C(3)-C(6)	2.23	-26.1	0.18
	C(9)-C(4)	2.22	-25.5	0.21
	C(6)-C(7)	2.15	-24.3	0.22
	C(7)-C(8)	2.16	-24.2	0.21

	C(8)-C(9)	2.16	-24.6	0.17
	C(1)-S(1)	1.54	-12.3	0.15
	C(11)-S(1)	1.36	-10.0	0.06
	C(10)-S(1)	1.36	-9.7	0.02
	C(9)-H(9)	1.97	-25.6	0.02
	C(8)-H(8)	1.97	-26.3	0.02
	C(7)-H(7)	1.94	-24.3	0.02
	C(6)-H(6)	1.94	-26.1	0.02
	C(10)-H(10A)	1.92	-25.2	0.02
	C(10)-H(10B)	1.98	-26.5	0.01
	C(10)-H(10C)	2.02	-27.5	0.01
	C(11)-H(11A)	1.92	-25.2	0.01
	C(11)-H(11B)	1.94	-24.8	0.01
	C(11)-H(11C)	1.92	-25.4	0.01
**Dataset number**	**Bond**	**Electron Density (ρ)** in e/Å^3	**Laplacian of Electron Density ($\nabla^2\rho$)** in e/Å^5	**Ellipticity (σ)**
**Dataset 10**	O(1)-C(2)	2.73	-8.5	0.07
	O(2)-C(5)	2.75	-6.2	0.07
	C(2)-C(3)	1.81	-18.3	0.07
	C(2)-C(1)	2.03	-21.1	0.25
	C(3)-C(4)	2.16	-23.5	0.14
	C(4)-C(5)	1.78	-17.5	0.09
	C(5)-C(1)	1.99	-20.5	0.25
	C(3)-C(6)	2.19	-24.7	0.13
	C(9)-C(4)	2.21	-25.3	0.18
	C(6)-C(7)	2.12	-23.3	0.19
	C(7)-C(8)	2.16	-24.1	0.23
	C(8)-C(9)	2.14	-24.2	0.18
	C(1)-S(1)	1.54	-12.8	0.15
	C(11)-S(1)	1.36	-9.5	0.04
	C(10)-S(1)	1.34	-10.1	0.02
	C(9)-H(9)	1.93	-25.8	0.03
	C(8)-H(8)	1.95	-25.8	0.02
	C(7)-H(7)	1.88	-24.8	0.01
	C(6)-H(6)	1.91	-25.2	0.02
	C(10)-H(10A)	1.92	-25.2	0.02
	C(10)-H(10B)	1.92	-25.4	0.01
	C(10)-H(10C)	2.01	-27.4	0.01
	C(11)-H(11A)	1.88	-25.6	0.02
	C(11)-H(11B)	1.93	-24.3	0.02
	C(11)-H(11C)	1.95	-25.3	0.01

**Dataset number**	**Bond**	**Electron Density (ρ)** in e/Å^3	**Laplacian of Electron Density ($\nabla^2\rho$)** in e/Å^5	**Ellipticity (σ)**
Dataset 11	O(1)-C(2)	2.66	-1.1	0.08
	O(2)-C(5)	2.71	-3.7	0.08
	C(2)-C(3)	1.84	-19.6	0.12
	C(2)-C(1)	2.08	-23.4	0.29
	C(3)-C(4)	2.20	-25.1	0.24
	C(4)-C(5)	1.88	-21.2	0.10
	C(5)-C(1)	2.01	-21.3	0.31
	C(3)-C(6)	2.27	-27.4	0.23
	C(9)-C(4)	2.19	-24.5	0.18
	C(6)-C(7)	2.17	-25.2	0.21
	C(7)-C(8)	2.16	-24.4	0.27
	C(8)-C(9)	2.21	-26.7	0.17
	C(1)-S(1)	1.58	-13.3	0.11
	C(11)-S(1)	1.39	-10.8	0.07
	C(10)-S(1)	1.42	-11.8	0.06
	C(9)-H(9)	1.89	-24.5	0.03
	C(8)-H(8)	1.93	-25.1	0.03
	C(7)-H(7)	1.54	-27.6	0.01
	C(6)-H(6)	1.96	-26.7	0.03
	C(10)-H(10A)	2.11	-24.7	0.02
	C(10)-H(10B)	2.01	-27.5	0.02
	C(10)-H(10C)	1.91	-30.1	0.01
	C(11)-H(11A)	1.93	-25.2	0.01
	C(11)-H(11B)	2.02	-26.8	0.02
	C(11)-H(11C)	1.91	-24.9	0.04
**Dataset number**	**Bond**	**Electron Density (ρ)** in e/Å^3	**Laplacian of Electron Density ($\nabla^2\rho$)** in e/Å^5	**Ellipticity (σ)**
**Dataset 12**	O(1)-C(2)	2.70	-2.4	0.05
	O(2)-C(5)	2.77	-2.9	0.13
	C(2)-C(3)	1.85	-19.7	0.23
	C(2)-C(1)	1.99	-20.5	0.29
	C(3)-C(4)	2.25	-27.5	0.21
	C(4)-C(5)	1.85	-19.9	0.06
	C(5)-C(1)	2.01	-21.7	0.21
	C(3)-C(6)	2.26	-26.8	0.38
	C(9)-C(4)	2.18	-23.8	0.26
	C(6)-C(7)	2.17	-24.7	0.20
	C(7)-C(8)	2.14	-23.3	0.18

	C(8)-C(9)	2.16	-24.4	0.28
	C(1)-S(1)	1.53	-11.4	0.12
	C(11)-S(1)	1.37	-10.1	0.04
	C(10)-S(1)	1.31	-8.5	0.03
	C(9)-H(9)	1.94	-26.4	0.02
	C(8)-H(8)	1.87	-24.8	0.04
	C(7)-H(7)	2.01	-27.8	0.03
	C(6)-H(6)	1.98	-27.6	0.03
	C(10)-H(10A)	2.17	-22.9	0.01
	C(10)-H(10B)	1.98	-25.2	0.06
	C(10)-H(10C)	1.83	-32.7	0.02
	C(11)-H(11A)	1.92	-24.7	0.02
	C(11)-H(11B)	2.06	-27.1	0.03
	C(11)-H(11C)	1.97	-26.2	0.02
**Dataset number**	**Bond**	**Electron Density (ρ)** in e/Å^3	**Laplacian of Electron Density ($\nabla^2\rho$) in e/Å^5**	**Ellipticity (σ)**
**Dataset 13**	O(1)-C(2)	2.65	-6.8	0.10
	O(2)-C(5)	2.73	-3.6	0.04
	C(2)-C(1)	2.04	-22.3	0.26
	C(2)-C(3)	1.84	-19.7	0.10
	C(3)-C(4)	2.24	-27.5	0.21
	C(4)-C(5)	1.83	-19.2	0.11
	C(5)-C(1)	2.03	-22.2	0.25
	C(3)-C(6)	2.22	-25.5	0.17
	C(6)-C(7)	2.18	-25.8	0.20
	C(7)-C(8)	2.20	-26.4	0.24
	C(8)-C(9)	2.20	-26.4	0.15
	C(9)-C(4)	2.22	-25.9	0.19
	C(1)-S(1)	1.54	-12.3	0.14
	C(10)-S(1)	1.33	-10.1	0.09
	C(11)-S(1)	1.44	-13.1	0.03
	C(6)-H(6)	1.91	-25.5	0.01
	C(7)-H(7)	1.93	-26.1	0.02
	C(8)-H(8)	1.90	-24.7	0.04
	C(9)-H(9)	1.89	-24.1	0.02
	C(10)-H(10A)	1.91	-24.1	0.01
	C(10)-H(10B)	1.96	-25.8	0.07
	C(10)-H(10C)	1.99	-25.9	0.03
	C(11)-H(11A)	1.93	-25.6	0.03
	C(11)-H(11B)	1.98	-26.1	0.16
	C(11)-H(11C)	2.05	-28.8	0.01

**Dataset number**	**Bond**	**Electron Density (ρ)** in e/Å^3	**Laplacian of Electron Density ($\nabla^2\rho$)** in e/Å^5	**Ellipticity (σ)**
**Dataset 14**	O(1)-C(2)	2.72	-1.4	0.08
	O(2)-C(5)	2.77	-0.5	0.03
	C(2)-C(1)	2.06	-23.6	0.25
	C(2)-C(3)	1.84	-19.6	0.16
	C(3)-C(4)	2.24	-28.6	0.16
	C(4)-C(5)	1.86	-20.1	0.07
	C(5)-C(1)	2.03	-22.4	0.21
	C(3)-C(6)	2.22	-25.1	0.37
	C(6)-C(7)	2.17	-26.1	0.17
	C(7)-C(8)	2.19	-26.5	0.21
	C(8)-C(9)	2.17	-25.5	0.16
	C(9)-C(4)	2.22	-26.3	0.20
	C(1)-S(1)	1.52	-11.9	0.11
	C(10)-S(1)	1.35	-11.8	0.02
	C(11)-S(1)	1.30	-8.2	0.09
	C(6)-H(6)	1.93	-26.7	0.01
	C(7)-H(7)	1.92	-25.6	0.02
	C(8)-H(8)	1.91	-25.4	0.03
	C(9)-H(9)	1.94	-25.8	0.01
	C(10)-H(10A)	1.89	-23.9	0.01
	C(10)-H(10B)	2.03	-27.4	0.04
	C(10)-H(10C)	1.61	-26.1	0.03
	C(11)-H(11A)	1.93	-27.5	0.04
	C(11)-H(11B)	1.96	-25.4	0.04
	C(11)-H(11C)	1.99	-24.3	0.03
**Dataset number**	**Bond**	**Electron Density (ρ)** in e/Å^3	**Laplacian of Electron Density ($\nabla^2\rho$)** in e/Å^5	**Ellipticity (σ)**
**Dataset 15**	O(1)-C(2)	2.71	-6.5	0.03
	O(2)-C(5)	2.76	-6.4	0.06
	C(2)-C(3)	1.75	-15.7	0.13
	C(2)-C(1)	2.01	-20.8	0.26
	C(3)-C(4)	2.18	-24.1	0.21
	C(4)-C(5)	1.78	-16.9	0.08
	C(5)-C(1)	1.99	-20.6	0.30
	C(3)-C(6)	2.21	-25.2	0.24
	C(9)-C(4)	2.18	-23.7	0.25
	C(6)-C(7)	2.20	-26.4	0.24
	C(7)-C(8)	2.18	-24.6	0.18

	C(8)-C(9)	2.15	-24.0	0.32
	C(1)-S(1)	1.53	-12.5	0.08
	C(11)-S(1)	1.35	-9.8	0.10
	C(10)-S(1)	1.35	-9.7	0.04
	C(9)-H(9)	1.86	-23.8	0.03
	C(8)-H(8)	1.92	-24.6	0.02
	C(7)-H(7)	1.89	-25.1	0.02
	C(6)-H(6)	1.90	-25.3	0.01
	C(10)-H(10A)	1.94	-24.4	0.07
	C(10)-H(10B)	1.91	-25.2	0.03
	C(10)-H(10C)	1.99	-27.9	0.02
	C(11)-H(11A)	2.05	-24.9	0.03
	C(11)-H(11B)	1.86	-28.6	0.04
	C(11)-H(11C)	1.92	-22.3	0.05

**Dataset number**	**Bond**	**Electron Density (ρ)** in e/Å^3	**Laplacian of Electron Density ($\nabla^2\rho$)** in e/Å^5	**Ellipticity (σ)**
**Dataset 16**	O(1)-C(2)	2.72	-2.7	0.07
	O(2)-C(5)	2.76	-4.1	0.16
	C(2)-C(3)	1.84	-19.4	0.15
	C(2)-C(1)	1.99	-20.2	0.39
	C(3)-C(4)	2.22	-25.8	0.22
	C(4)-C(5)	1.83	-19.3	0.10
	C(5)-C(1)	2.04	-22.1	0.25
	C(3)-C(6)	2.22	-25.1	0.41
	C(9)-C(4)	2.23	-25.3	0.32
	C(6)-C(7)	2.19	-25.7	0.20
	C(7)-C(8)	2.14	-23.2	0.22
	C(8)-C(9)	2.16	-23.8	0.34
	C(1)-S(1)	1.55	-12.7	0.09
	C(11)-S(1)	1.38	-11.2	0.01
	C(10)-S(1)	1.40	-11.1	0.04
	C(9)-H(9)	1.96	-26.2	0.04
	C(8)-H(8)	1.93	-25.10	0.06
	C(7)-H(7)	1.93	-25.9	0.03
	C(6)-H(6)	1.87	-24.4	0.01
	C(10)-H(10A)	1.95	-24.9	0.06
	C(10)-H(10B)	2.01	-25.8	0.05
	C(10)-H(10C)	2.15	-32.2	0.01
	C(11)-H(11A)	1.93	-26.1	0.05
	C(11)-H(11B)	2.03	-26.8	0.04
	C(11)-H(11C)	1.98	-23.8	0.07

Dataset number	Bond	Electron Density (ρ) in e/Å^3	Laplacian of Electron Density ($\nabla^2\rho$) in e/Å^5	Ellipticity (σ)
**Dataset 17**	O(1)-C(2)	2.68	-1.5	0.07
	O(2)-C(5)	2.72	-5.3	0.10
	C(2)-C(3)	1.84	-19.2	0.08
	C(2)-C(1)	2.01	-20.9	0.27
	C(3)-C(4)	2.19	-24.4	0.21
	C(4)-C(5)	1.84	-19.5	0.11
	C(5)-C(1)	2.02	-21.4	0.27
	C(3)-C(6)	2.22	-25.3	0.23
	C(9)-C(4)	2.19	-23.8	0.20
	C(6)-C(7)	2.16	-24.8	0.22
	C(7)-C(8)	2.15	-23.7	0.28
	C(8)-C(9)	2.16	-24.4	0.15
	C(1)-S(1)	1.55	-12.7	0.12
	C(11)-S(1)	1.39	-11.1	0.07
	C(10)-S(1)	1.55	-11.4	0.05
	C(9)-H(9)	1.93	-25.2	0.02
	C(8)-H(8)	1.92	-25.1	0.03
	C(7)-H(7)	1.90	-25.4	0.02
	C(6)-H(6)	1.93	-25.9	0.01
	C(10)-H(10A)	1.96	-25.6	0.01
	C(10)-H(10B)	2.03	-27.5	0.03
	C(10)-H(10C)	2.11	-30.4	0.02
	C(11)-H(11A)	1.95	-24.0	0.01
	C(11)-H(11B)	2.02	-26.6	0.01
	C(11)-H(11C)	1.91	-25.7	0.06
**Dataset number**	**Bond**	**Electron Density (ρ)** in e/Å^3	**Laplacian of Electron Density ($\nabla^2\rho$)** in e/Å^5	**Ellipticity (σ)**
**Dataset 18**	O(1)-C(2)	2.58	-1.6	0.05
	O(2)-C(5)	2.64	-5.8	0.15
	C(2)-C(3)	1.76	-17.1	0.11
	C(2)-C(1)	2.04	-22.8	0.21
	C(3)-C(4)	2.13	-22.8	0.17
	C(4)-C(5)	1.82	-19.5	0.06
	C(5)-C(1)	1.98	-20.7	0.31
	C(3)-C(6)	2.17	-24.6	0.19
	C(9)-C(4)	2.22	-27.1	0.16
	C(6)-C(7)	2.15	-25.6	0.20
	C(7)-C(8)	2.13	-24.0	0.23

	C(8)-C(9)	2.18	-26.4	0.17
	C(1)-S(1)	1.48	-11.4	0.08
	C(11)-S(1)	1.20	-9.4	0.06
	C(10)-S(1)	1.34	-5.5	0.08
	C(9)-H(9)	1.80	-22.3	0.01
	C(8)-H(8)	1.89	-24.9	0.01
	C(7)-H(7)	1.91	-25.9	0.01
	C(6)-H(6)	1.97	-26.8	0.02
	C(10)-H(10A)	1.89	-23.6	0.03
	C(10)-H(10B)	1.98	-25.3	0.01
	C(10)-H(10C)	1.96	-26.8	0.00
	C(11)-H(11A)	1.91	-21.5	0.03
	C(11)-H(11B)	1.82	-23.1	0.03
	C(11)-H(11C)	1.90	-25.8	0.02
**Dataset number**	**Bond**	**Electron Density (ρ)** in e/Å^3	**Laplacian of Electron Density ($\nabla^2\rho$)** in e/Å^5	**Ellipticity (σ)**
**Dataset 19**	O(1)-C(2)	2.73	-4.1	0.03
	O(2)-C(5)	2.75	-0.3	0.03
	C(2)-C(1)	1.99	-20.3	0.23
	C(2)-C(3)	1.78	-16.2	0.08
	C(3)-C(4)	2.21	-25.0	0.13
	C(4)-C(5)	1.79	-16.8	0.06
	C(5)-C(1)	2.00	-20.2	0.28
	C(3)-C(6)	2.20	-25.3	0.18
	C(6)-C(7)	2.21	-26.1	0.23
	C(7)-C(8)	2.21	-25.2	0.31
	C(8)-C(9)	2.21	-25.8	0.16
	C(9)-C(4)	2.28	-27.6	0.18
	C(1)-S(1)	1.28	-10.5	0.03
	C(10)-S(1)	1.14	-11.9	0.21
	C(11)-S(1)	1.32	-9.1	0.11
	C(6)-H(6)	1.97	-26.3	0.02
	C(7)-H(7)	1.89	-24.6	0.02
	C(8)-H(8)	1.89	-24.1	0.01
	C(9)-H(9)	1.94	-25.3	0.01
	C(10)-H(10A)	1.87	-21.8	0.05
	C(10)-H(10B)	1.89	-23.6	0.04
	C(10)-H(10C)	2.05	-28.0	0.02
	C(11)-H(11A)	1.92	-25.2	0.02
	C(11)-H(11B)	1.95	-25.9	0.02
	C(11)-H(11C)	2.01	-28.8	0.02

Dataset number	Bond	Electron Density (ρ) in e/Å^3	Laplacian of Electron Density ($\nabla^2\rho$) in e/Å^5	Ellipticity (σ)
**Dataset 20**	O(1)-C(2)	2.64	-0.6	0.05
	O(2)-C(5)	2.68	-0.1	0.10
	C(2)-C(1)	1.98	-20.5	0.24
	C(2)-C(3)	1.77	-16.8	0.09
	C(3)-C(4)	2.18	-25.2	0.17
	C(4)-C(5)	1.78	-17.9	0.11
	C(5)-C(1)	1.97	-20.1	0.25
	C(3)-C(6)	2.21	-26.8	0.10
	C(6)-C(7)	2.14	-24.4	0.22
	C(7)-C(8)	2.13	-23.7	0.25
	C(8)-C(9)	2.17	-25.5	0.13
	C(9)-C(4)	2.18	-24.5	0.18
	C(1)-S(1)	1.42	-8.32	0.21
	C(10)-S(1)	1.36	-11.6	0.10
	C(11)-S(1)	1.28	-8.1	0.13
	C(6)-H(6)	1.86	-23.9	0.01
	C(7)-H(7)	1.86	-24.7	0.01
	C(8)-H(8)	1.90	-24.8	0.03
	C(9)-H(9)	1.88	-24.2	0.01
	C(10)-H(10A)	1.93	-25.8	0.04
	C(10)-H(10B)	1.92	-26.6	0.01
	C(10)-H(10C)	2.03	-27.2	0.03
	C(11)-H(11A)	1.97	-21.6	0.03
	C(11)-H(11B)	1.81	-25.3	0.02
	C(11)-H(11C)	1.94	-26.6	0.02
**Dataset number**	**Bond**	**Electron Density (ρ) in e/Å^3**	**Laplacian of Electron Density ($\nabla^2\rho$) in e/Å^5**	**Ellipticity (σ)**
**Dataset 21**	O(1)-C(2)	2.60	-6.2	0.03
	O(2)-C(5)	2.66	-5.3	0.10
	C(2)-C(3)	1.77	-17.1	0.12
	C(2)-C(1)	1.96	-19.1	0.25
	C(3)-C(4)	2.16	-23.9	0.19
	C(4)-C(5)	1.77	-17.4	0.09
	C(5)-C(1)	1.99	-20.9	0.33
	C(3)-C(6)	2.17	-24.6	0.17
	C(9)-C(4)	2.19	-24.9	0.23
	C(6)-C(7)	2.18	-25.6	0.29
	C(7)-C(8)	2.15	-24.3	0.26

	C(8)-C(9)	2.15	-24.7	0.16
	C(1)-S(1)	1.45	-9.4	0.19
	C(11)-S(1)	1.32	-10.0	0.09
	C(10)-S(1)	1.36	-10.4	0.05
	C(9)-H(9)	1.93	-25.3	0.02
	C(8)-H(8)	1.90	-24.7	0.03
	C(7)-H(7)	1.89	-25.0	0.01
	C(6)-H(6)	1.87	-24.6	0.01
	C(10)-H(10A)	1.96	-26.3	0.04
	C(10)-H(10B)	1.93	-25.4	0.03
	C(10)-H(10C)	1.97	-27.4	0.02
	C(11)-H(11A)	1.83	-22.3	0.03
	C(11)-H(11B)	1.92	-24.9	0.05
	C(11)-H(11C)	2.03	-28.0	0.02
**Dataset number**	**Bond**	**Electron Density (ρ)** in e/Å^3	**Laplacian of Electron Density ($\nabla^2\rho$)** in e/Å^5	**Ellipticity (σ)**
**Dataset 22**	O(1)-C(2)	2.67	-0.1	0.03
	O(2)-C(5)	6.66	-2.2	0.07
	C(2)-C(1)	2.00	-21.6	0.32
	C(2)-C(3)	1.80	-17.7	0.10
	C(3)-C(4)	2.18	-24.9	0.25
	C(4)-C(5)	1.78	-18.0	0.12
	C(5)-C(1)	2.03	-22.4	0.34
	C(3)-C(6)	2.20	-26.7	0.16
	C(6)-C(7)	2.17	-25.6	0.22
	C(7)-C(8)	2.15	-24.5	0.28
	C(8)-C(9)	2.15	-25.2	0.18
	C(9)-C(4)	2.24	-27.0	0.20
	C(1)-S(1)	1.40	-6.6	0.03
	C(10)-S(1)	1.29	-9.7	0.31
	C(11)-S(1)	1.25	-5.7	0.20
	C(6)-H(6)	1.89	-25.2	0.01
	C(7)-H(7)	1.86	-24.9	0.01
	C(8)-H(8)	1.88	-24.1	0.02
	C(9)-H(9)	1.95	-25.5	0.01
	C(10)-H(10A)	1.93	-25.4	0.06
	C(10)-H(10B)	2.03	-25.8	0.07
	C(10)-H(10C)	1.95	-29.3	0.04
	C(11)-H(11A)	1.99	-26.8	0.02
	C(11)-H(11B)	1.81	-26.3	0.05
	C(11)-H(11C)	1.97	-21.7	0.03

**Dataset number**	**Bond**	**Electron Density (ρ)** in e/Å^3	**Laplacian of Electron Density ($\nabla^2\rho$)** in e/Å^5	**Ellipticity (σ)**
**Dataset 23**	O(1)-C(2)	2.68	-3.5	0.12
	O(2)-C(5)	2.68	-4.1	0.05
	C(2)-C(1)	2.02	-22.2	0.26
	C(2)-C(3)	1.75	-16.1	0.09
	C(3)-C(4)	2.17	-23.9	0.21
	C(4)-C(5)	1.78	-16.9	0.04
	C(5)-C(1)	1.97	-19.6	0.37
	C(3)-C(6)	2.16	-23.7	0.22
	C(6)-C(7)	2.18	-26.1	0.23
	C(7)-C(8)	2.15	-24.0	0.28
	C(8)-C(9)	2.20	-26.5	0.16
	C(9)-C(4)	2.19	-24.8	0.21
	C(1)-S(1)	1.38	-5.6	0.12
	C(10)-S(1)	1.21	-5.8	0.07
	C(11)-S(1)	1.31	-8.1	0.07
	C(6)-H(6)	1.98	-27.2	0.01
	C(7)-H(7)	1.88	-24.5	0.01
	C(8)-H(8)	1.89	-24.2	0.01
	C(9)-H(9)	1.88	-24.2	0.01
	C(10)-H(10A)	1.77	-20.6	0.04
	C(10)-H(10B)	1.95	-24.7	0.03
	C(10)-H(10C)	1.95	-26.7	0.04
	C(11)-H(11A)	1.98	-26.1	0.06
	C(11)-H(11B)	2.00	-26.5	0.04
	C(11)-H(11C)	1.97	-27.6	0.03
**Dataset number**	**Bond**	**Electron Density (ρ)** in e/Å^3	**Laplacian of Electron Density ($\nabla^2\rho$)** in e/Å^5	**Ellipticity (σ)**
**Theoretical calculations, opt**	O(1)-C(2)	2.82	-4.6	0.07
	O(2)-C(5)	2.76	-6.3	0.06
	C(2)-C(3)	1.77	-16.8	0.06
	C(2)-C(1)	1.94	-18.7	0.20
	C(3)-C(4)	2.18	-23.1	0.16
	C(4)-C(5)	1.79	-17.1	0.07
	C(5)-C(1)	1.98	-19.4	0.21
	C(3)-C(9)	2.19	-23.5	0.19
	C(6)-C(4)	2.13	-22.4	0.18
	C(6)-C(7)	2.15	-22.7	0.19
	C(7)-C(8)	2.13	-22.4	0.18

	C(8)-C(9)	2.19	-23.4	0.19
	C(1)-S(1)	1.55	-11.7	0.19
	C(11)-S(1)	1.29	-8.5	0.05
	C(10)-S(1)	1.29	-8.5	0.05
	C(9)-H(9)	1.93	-24.6	0.01
	C(8)-H(8)	1.93	-24.6	0.01
	C(7)-H(7)	1.93	-24.6	0.01
	C(6)-H(6)	2.13	-24.8	0.01
	C(10)-H(10A)	1.90	-24.1	0.01
	C(10)-H(10B)	1.29	-24.1	0.01
	C(10)-H(10C)	1.88	-23.1	0.01
	C(11)-H(11A)	1.88	-23.6	0.01
	C(11)-H(11B)	1.90	-23.6	0.01
	C(11)-H(11C)	1.914	-23.1	0.01

Table S10. Atomic properties obtained from QTAIM analysis related to multipole refinement. Q= Atomic integrated charge; V= Atomic volume; N= Population of electrons. 001 means that the basin was cropped at an electron-density isovalue of 0.001 a.u.

**Dataset number**	**Atom**	**Q001** in e	**V001** in Å^3	**N001** in e
**Dataset 1**	S(1)	0.33	18.90	15.66
	O(1)	−0.99	17.03	8.99
	O(2)	−1.00	18.55	9.00
	C(1)	−0.28	11.12	6.28
	C(2)	0.75	7.50	5.24
	C(3)	0.03	9.96	5.96
	C(4)	0.07	9.94	5.92
	C(5)	0.85	7.45	5.14
	C(6)	0.01	11.31	5.99
	C(7)	0.11	11.52	5.88
	C(8)	0.29	10.69	5.70
	C(9)	0.19	11.45	5.80
	C(10)	−0.21	10.39	6.21
	C(11)	−0.13	10.70	6.13
	H(6)	0.02	7.44	0.97
	H(7)	−0.03	6.94	1.03
	H(8)	−0.17	8.02	1.17
	H(9)	−0.11	8.43	1.11
	H(10C)	-0.01	5.59	1.01
	H(10B)	−0.01	6.84	1.01
	H(10A)	0.23	5.27	0.76
	H(11A)	−0.07	6.33	1.07
	H(11B)	−0.07	7.58	1.07
	H(11C)	0.26	5.71	0.73

**Dataset 3**	S(1)	0.22	19.95	15.77
	O(1)	−1.02	17.24	9.02
	O(2)	−1.03	18.45	9.03
	C(1)	−0.30	11.29	6.30
	C(2)	0.96	7.44	5.03
	C(3)	0.04	9.80	5.95
	C(4)	0.21	9.32	5.78
	C(5)	0.96	7.69	5.03
	C(6)	−0.28	12.67	6.28
	C(7)	0.26	11.33	5.73
	C(8)	0.27	10.80	5.72
	C(9)	0.09	12.44	5.90
	C(10)	0.15	9.73	5.84
	C(11)	0.21	9.62	5.78
	H(6)	0.04	7.06	0.95
	H(7)	−0.03	7.06	1.03
	H(8)	−0.27	7.92	1.27
	H(9)	−0.12	7.96	1.12
	H(10C)	−0.07	5.60	1.07
	H(10B)	−0.22	6.93	1.22
	H(10A)	0.22	5.22	0.77
	H(11A)	−0.27	7.00	1.27
	H(11B)	−0.10	7.00	1.10
	H(11C)	0.08	6.25	0.91
**Dataset 16**	S(1)	0.75	18.15	15.24
	O(1)	−1.18	18.18	9.18
	O(2)	−1.21	19.26	9.21
	C(1)	−0.16	11.22	6.16
	C(2)	0.68	7.26	5.31
	C(3)	−0.21	10.22	6.21
	C(4)	−0.04	10.06	6.04
	C(5)	0.91	7.32	5.08
	C(6)	−0.27	12.77	6.27
	C(7)	0.31	10.02	5.68
	C(8)	0.57	9.04	5.42
	C(9)	0.38	10.09	5.61
	C(10)	0.12	10.42	5.87
	C(11)	0.25	9.05	5.74
	H(6)	0.09	7.09	0.90
	H(7)	0.02	6.10	0.97
	H(8)	−0.55	8.09	1.55
	H(9)	−0.18	7.07	1.18
	H(10C)	−0.04	5.39	1.04
	H(10B)	−0.01	5.56	1.01
	H(10A)	0.11	5.65	0.88
	H(11A)	−0.33	6.52	1.33
	H(11B)	−0.21	6.40	1.21

	H(11C)	0.25	5.41	0.74
**Dataset 17**	S(1)	0.66	18.34	15.33
	O(1)	−1.10	17.85	9.10
	O(2)	−1.14	19.24	9.14
	C(1)	−0.34	11.68	6.34
	C(2)	0.77	6.88	5.22
	C(3)	0.24	9.08	5.75
	C(4)	0.25	8.63	5.74
	C(5)	0.75	7.60	5.24
	C(6)	0.08	11.39	5.91
	C(7)	−0.02	11.70	6.02
	C(8)	0.27	9.41	5.72
	C(9)	0.35	10.32	5.64
	C(10)	0.11	9.78	5.88
	C(11)	0.03	10.57	5.96
	H(6)	0.11	7.24	0.88
	H(7)	−0.11	6.63	1.11
	H(8)	−0.42	7.65	1.42
	H(9)	−0.36	7.84	1.36
	H(10C)	−0.09	5.73	1.09
	H(10B)	−0.06	6.26	1.06
	H(10A)	0.18	5.46	0.81
	H(11A)	−0.31	6.49	1.31
	H(11B)	−0.02	6.96	1.02
	H(11C)	0.23	5.46	0.76

Table S11. Atomic properties obtained from QTAIM analysis related to XWR and isolated-molecule DFT calculation. Q= Atomic integrated charge; V= Atomic volume; N= Population of electrons. 001 means that the basin was cropped at an electron-density isovalue of 0.001 a.u.

Dataset Number	Atom	**Q001** in e	**V001** in Å^3	**N001** in e
**Dataset 1**	S(1)	0.46	18.64	15.54
	O(1)	-1.28	20.03	9.28
	O(2)	-1.23	20.45	9.23
	C(1)	-0.29	12.16	6.29
	C(2)	1.07	6.63	4.92
	C(3)	0.01	10.26	5.98
	C(4)	-0.06	10.37	6.06
	C(5)	1.06	6.79	4.93
	C(6)	-0.01	12.35	6.01
	C(7)	-0.06	12.60	6.06
	C(8)	-0.01	12.41	6.01
	C(9)	0.00	12.24	5.99

	C(10)	-0.15	11.01	6.15
	C(11)	-0.16	11.12	6.16
	H(6)	0.10	6.83	0.89
	H(7)	0.09	6.88	0.90
	H(8)	0.08	6.90	0.91
	H(9)	0.09	6.81	0.90
	H(10C)	0.12	5.73	0.87
	H(10B)	0.11	6.46	0.88
	H(10A)	0.11	6.31	0.88
	H(11A)	0.12	6.41	0.87
	H(11B)	0.13	6.59	0.86
	H(11C)	0.11	6.30	0.88
**Dataset Number**	**Atom**	**Q001** in e	**V001** in Å^3	**N001** in e
Dataset 2	S(1)	0.24	19.28	15.75
	O(1)	-1.17	20.39	9.17
	O(2)	-1.17	21.32	9.17
	C(1)	-0.18	12.01	6.18
	C(2)	0.97	7.07	5.02
	C(3)	0.07	10.40	5.92
	C(4)	-0.01	10.72	6.01
	C(5)	1.01	7.12	4.99
	C(6)	-0.03	12.93	6.03
	C(7)	-0.11	12.94	6.11
	C(8)	0.00	12.53	5.99
	C(9)	-0.07	12.89	6.07
	C(10)	-0.13	10.98	6.13
	C(11)	-0.18	11.32	6.18
	H(6)	0.13	6.63	0.86
	H(7)	0.09	7.27	0.90
	H(8)	0.09	7.01	0.90
	H(9)	0.12	6.62	0.87
	H(10C)	0.13	6.63	0.86
	H(10B)	0.15	5.71	0.84
	H(10A)	0.11	6.77	0.88
	H(11A)	0.13	6.21	0.86
	H(11B)	0.15	6.45	0.84
	H(11C)	0.13	6.62	0.86
**Dataset Number**	**Atom**	**Q001** in e	**V001** in Å^3	**N001** in e
Dataset 3	S(1)	0.42	19.43	15.57
	O(1)	-1.25	20.07	9.25
	O(2)	-1.23	20.87	9.23

	C(1)	-0.30	12.76	6.30
	C(2)	1.09	6.76	4.90
	C(3)	-0.05	10.53	6.05
	C(4)	0.05	10.23	5.94
	C(5)	1.09	6.96	4.90
	C(6)	-0.05	13.12	6.05
	C(7)	0.02	12.83	5.97
	C(8)	0.06	12.34	5.93
	C(9)	-0.03	12.79	6.03
	C(10)	-0.16	11.23	6.16
	C(11)	-0.04	10.87	6.04
	H(6)	0.08	7.31	0.91
	H(7)	0.05	7.38	0.94
	H(8)	0.04	7.52	0.95
	H(9)	0.07	7.09	0.92
	H(10C)	0.12	6.58	0.87
	H(10B)	0.11	6.61	0.88
	H(10A)	0.09	5.98	0.90
	H(11A)	0.09	6.95	0.90
	H(11B)	0.09	6.96	0.90
	H(11C)	0.08	6.59	0.91
**Dataset Number**	**Atom**	**Q001** in e	**V001** in Å^3	**N001** in e
Dataset 4	S(1)	0.42	18.55	15.54
	O(1)	-1.31	20.11	9.27
	O(2)	-1.28	20.69	9.23
	C(1)	-0.27	11.99	6.26
	C(2)	1.08	6.63	4.90
	C(3)	-0.01	6.77	6.00
	C(4)	-0.07	10.34	6.06
	C(5)	1.06	10.37	4.93
	C(6)	-0.01	12.18	5.98
	C(7)	-0.10	12.68	6.08
	C(8)	-0.03	12.44	6.01
	C(9)	-0.00	12.20	5.98
	C(10)	-0.11	10.83	6.10
	C(11)	-0.11	10.84	6.09
	H(6)	0.07	6.90	0.90
	H(7)	0.06	7.05	0.91
	H(8)	0.06	7.00	0.91
	H(9)	0.06	6.92	0.91
	H(10C)	0.08	6.58	0.89

	H(10B)	0.07	6.59	0.90
	H(10A)	0.11	5.73	0.86
	H(11A)	0.07	6.31	0.88
	H(11B)	0.04	6.58	0.90
	H(11C)	0.09	6.81	0.93
**Dataset Number**	**Atom**	**Q001** in e	**V001** in Å^3	**N001** in e
Dataset 5	S(1)	0.26	17.80	15.71
	O(1)	-1.34	19.48	9.33
	O(2)	-1.37	19.71	9.30
	C(1)	-0.17	11.66	6.16
	C(2)	1.09	6.49	4.89
	C(3)	-0.09	10.22	6.08
	C(4)	-0.10	10.13	6.09
	C(5)	1.13	6.45	4.86
	C(6)	0.06	11.84	5.92
	C(7)	-0.09	12.39	6.07
	C(8)	-0.12	12.49	6.10
	C(9)	-0.02	11.93	6.00
	C(10)	-0.17	11.08	6.16
	C(11)	-0.11	10.86	6.10
	H(6)	0.11	6.50	0.86
	H(7)	0.10	6.44	0.87
	H(8)	0.11	6.54	0.86
	H(9)	0.08	6.65	0.89
	H(10C)	0.11	6.25	0.86
	H(10B)	0.12	6.26	0.87
	H(10A)	0.10	5.47	0.86
	H(11A)	0.13	6.38	0.85
	H(11B)	0.06	5.94	0.88
	H(11C)	0.09	6.51	0.91
**Dataset Number**	**Atom**	**Q001** in e	**V001** in Å^3	**N001** in e
Dataset 6	S(1)	0.50	17.90	15.47
	O(1)	-1.30	20.56	9.26
	O(2)	-1.30	19.77	9.26
	C(1)	-0.41	12.14	6.40
	C(2)	1.10	6.42	4.88
	C(3)	-0.11	10.45	6.10
	C(4)	-0.07	10.63	6.06
	C(5)	1.15	6.51	4.84
	C(6)	0.11	11.50	5.87
	C(7)	-0.11	12.33	6.09

	C(8)	-0.02	12.49	6.00
	C(9)	0.10	11.70	5.87
	C(10)	-0.02	10.22	6.01
	C(11)	-0.04	10.43	6.03
	H(6)	0.02	7.04	0.95
	H(7)	0.07	7.06	0.90
	H(8)	-0.01	7.40	0.98
	H(9)	0.01	7.27	0.96
	H(10C)	0.06	6.91	0.90
	H(10B)	0.02	6.68	0.96
	H(10A)	0.10	5.64	0.87
	H(11A)	0.03	6.34	0.94
	H(11B)	0.12	7.00	0.98
	H(11C)	-0.01	6.62	0.85
**Dataset Number**	**Atom**	**Q001** in e	**V001** in Å^3	**N001** in e
Dataset 7	S(1)	0.54	18.12	15.43
	O(1)	-1.28	20.88	9.23
	O(2)	-1.32	20.02	9.28
	C(1)	-0.37	11.85	6.35
	C(2)	1.03	6.77	4.95
	C(3)	-0.05	10.47	6.04
	C(4)	0.05	10.14	5.93
	C(5)	1.13	6.64	4.85
	C(6)	0.06	11.77	5.91
	C(7)	-0.11	12.67	6.09
	C(8)	-0.05	12.27	6.03
	C(9)	-0.02	12.27	6.00
	C(10)	-0.13	10.54	6.12
	C(11)	-0.10	10.51	6.09
	H(6)	0.03	7.19	0.94
	H(7)	0.08	6.96	0.89
	H(8)	0.01	7.49	0.95
	H(9)	0.03	7.47	0.94
	H(10C)	0.05	6.72	0.92
	H(10B)	0.06	5.46	0.91
	H(10A)	0.16	6.58	0.82
	H(11A)	0.10	6.33	0.87
	H(11B)	-0.02	7.24	1.00
	H(11C)	0.08	6.33	0.89
**Dataset Number**	**Atom**	**Q001** in e	**V001** in Å^3	**N001** in e
Dataset 8	S(1)	0.84	18.01	15.12

	O(1)	-1.26	20.67	9.21
	O(2)	-1.21	19.92	9.18
	C(1)	-0.21	11.64	6.19
	C(2)	0.95	7.09	5.03
	C(3)	-0.01	10.54	6.00
	C(4)	-0.04	10.49	6.03
	C(5)	1.05	6.76	4.94
	C(6)	0.02	12.05	5.95
	C(7)	-0.03	12.45	6.01
	C(8)	-0.18	12.80	6.16
	C(9)	0.03	12.03	5.94
	C(10)	-0.57	11.90	6.56
	C(11)	-0.02	10.54	6.01
	H(6)	0.07	6.94	0.90
	H(7)	0.07	6.89	0.90
	H(8)	0.04	6.97	0.92
	H(9)	0.05	7.31	0.92
	H(10C)	0.09	6.40	0.88
	H(10B)	0.11	5.76	0.92
	H(10A)	0.05	6.86	0.87
	H(11A)	0.01	6.22	0.96
	H(11B)	0.04	6.78	0.92
	H(11C)	0.09	6.95	0.88
**Dataset Number**	**Atom**	**Q001** in e	**V001** in Å^3	**N001** in e
Dataset 9	S(1)	0.41	18.84	15.58
	O(1)	-1.28	20.59	9.28
	O(2)	-1.24	20.10	9.24
	C(1)	-0.25	12.07	6.25
	C(2)	1.10	6.58	4.89
	C(3)	-0.03	10.46	6.03
	C(4)	-0.05	10.38	6.05
	C(5)	1.07	6.75	4.92
	C(6)	0.01	12.31	5.98
	C(7)	-0.08	12.76	6.08
	C(8)	-0.02	12.48	6.02
	C(9)	0.01	12.22	5.98
	C(10)	-0.14	10.93	6.14
	C(11)	-0.14	11.09	6.14
	H(6)	0.10	6.92	0.89
	H(7)	0.10	6.95	0.89
	H(8)	0.08	6.86	0.91

	H(9)	0.09	6.87	0.90
	H(10C)	0.12	6.53	0.87
	H(10B)	0.11	6.44	0.88
	H(10A)	0.11	5.77	0.88
	H(11A)	0.12	6.61	0.87
	H(11B)	0.10	6.51	0.89
	H(11C)	0.12	6.33	0.87
**Dataset Number**	**Atom**	**Q001** in e	**V001** in Å^3	**N001** in e
Dataset 10	S(1)	0.26	19.46	15.73
	O(1)	-1.21	20.41	9.21
	O(2)	-1.20	21.29	9.20
	C(1)	-0.21	11.88	6.21
	C(2)	0.98	7.01	5.01
	C(3)	0.03	10.35	5.96
	C(4)	-0.04	10.53	6.04
	C(5)	1.05	6.89	4.94
	C(6)	0.00	12.46	5.99
	C(7)	-0.10	12.86	6.10
	C(8)	-0.01	12.45	6.01
	C(9)	-0.01	12.58	6.01
	C(10)	-0.11	10.91	6.11
	C(11)	-0.13	11.12	6.13
	H(6)	0.10	6.79	0.89
	H(7)	0.10	7.22	0.89
	H(8)	0.07	7.09	0.92
	H(9)	0.12	6.77	0.87
	H(10C)	0.13	6.48	0.86
	H(10B)	0.13	6.77	0.86
	H(10A)	0.10	5.75	0.89
	H(11A)	0.12	6.47	0.87
	H(11B)	0.14	6.58	0.85
	H(11C)	0.10	6.23	0.89
**Dataset Number**	**Atom**	**Q001** in e	**V001** in Å^3	**N001** in e
Dataset 11	S(1)	0.32	18.99	15.67
	O(1)	-1.33	20.21	9.33
	O(2)	-1.23	20.13	9.23
	C(1)	-0.20	11.98	6.20
	C(2)	1.09	6.50	4.90
	C(3)	-0.09	10.61	6.09
	C(4)	-0.01	10.33	6.00
	C(5)	1.11	6.50	4.88

	C(6)	-0.06	12.73	6.06
	C(7)	-0.12	12.83	6.12
	C(8)	-0.01	12.26	6.01
	C(9)	0.03	12.01	5.96
	C(10)	-0.12	10.87	6.12
	C(11)	-0.09	10.91	6.09
	H(6)	0.10	6.83	0.89
	H(7)	0.13	6.66	0.86
	H(8)	0.11	6.66	0.88
	H(9)	0.11	6.58	0.88
	H(10C)	0.09	6.61	0.90
	H(10B)	0.13	5.64	0.86
	H(10A)	0.10	6.30	0.89
	H(11A)	0.15	6.13	0.84
	H(11B)	0.11	6.60	0.88
	H(11C)	0.10	6.32	0.89
**Dataset Number**	**Atom**	**Q001** in e	**V001** in Å^3	**N001** in e
Dataset 12	S(1)	0.31	19.04	15.68
	O(1)	-1.26	20.85	9.26
	O(2)	-1.27	20.32	9.27
	C(1)	-0.15	11.73	6.15
	C(2)	1.02	6.72	4.97
	C(3)	-0.08	10.58	6.08
	C(4)	0.02	10.38	5.97
	C(5)	1.13	6.71	4.86
	C(6)	-0.09	12.45	6.09
	C(7)	-0.02	12.33	6.02
	C(8)	-0.01	12.51	6.01
	C(9)	-0.04	12.50	6.04
	C(10)	-0.16	11.34	6.16
	C(11)	-0.09	11.05	6.09
	H(6)	0.09	6.73	0.90
	H(7)	0.10	6.62	0.89
	H(8)	0.13	6.69	0.86
	H(9)	0.10	6.79	0.89
	H(10C)	0.13	6.49	0.86
	H(10B)	0.12	5.55	0.87
	H(10A)	0.13	6.16	0.86
	H(11A)	0.09	6.48	0.90
	H(11B)	0.16	6.43	0.83
	H(11C)	0.06	6.08	0.93

**Dataset Number**	**Atom**	**Q001** in e	**V001** in Å^3	**N001** in e
Dataset 13	S(1)	0.46	18.66	15.51
	O(1)	-1.35	20.39	9.32
	O(2)	-1.32	20.94	9.27
	C(1)	-0.06	11.72	6.05
	C(2)	1.05	6.66	4.93
	C(3)	-0.02	10.32	6.01
	C(4)	-0.03	10.28	6.02
	C(5)	1.05	6.77	4.93
	C(6)	-0.03	12.53	6.01
	C(7)	-0.22	13.14	6.20
	C(8)	-0.03	12.57	6.01
	C(9)	0.02	12.34	5.95
	C(10)	-0.19	11.08	6.18
	C(11)	-0.16	11.08	6.15
	H(6)	0.09	6.98	0.88
	H(7)	0.09	6.96	0.88
	H(8)	0.07	6.99	0.89
	H(9)	0.07	6.88	0.90
	H(10C)	0.07	6.64	0.90
	H(10B)	0.04	6.72	0.93
	H(10A)	0.11	6.20	0.86
	H(11A)	0.09	5.69	0.87
	H(11B)	0.08	6.46	0.88
	H(11C)	0.11	6.41	0.89
**Dataset Number**	**Atom**	**Q001** in e	**V001** in Å^3	**N001** in e
Dataset 14	S(1)	0.56	19.08	15.40
	O(1)	-1.3	20.92	9.32
	O(2)	-1.31	21.46	9.26
	C(1)	-0.22	12.37	6.20
	C(2)	1.10	6.70	4.88
	C(3)	-0.09	10.79	6.08
	C(4)	-0.01	10.46	5.99
	C(5)	1.07	6.86	4.91
	C(6)	-0.02	12.69	6.00
	C(7)	-0.17	13.29	6.15
	C(8)	-0.01	12.70	5.99
	C(9)	0.11	12.35	5.86
	C(10)	-0.23	11.15	6.22
	C(11)	-0.10	11.21	6.08
	H(6)	0.08	7.16	0.89

	H(7)	0.09	7.04	0.88
	H(8)	0.05	7.30	0.92
	H(9)	0.04	7.18	0.93
	H(10C)	0.08	6.70	0.89
	H(10B)	0.02	6.48	0.95
	H(10A)	0.08	6.97	0.90
	H(11A)	0.11	6.63	0.87
	H(11B)	0.08	6.57	0.89
	H(11C)	0.04	5.90	0.93
**Dataset Number**	**Atom**	**Q001** in e	**V001** in Å^3	**N001** in e
Dataset 15	S(1)	0.28	18.87	15.71
	O(1)	-1.19	20.21	9.19
	O(2)	-1.18	21.16	9.18
	C(1)	-0.27	11.91	6.27
	C(2)	1.01	6.76	4.98
	C(3)	-0.04	10.16	6.04
	C(4)	-0.05	10.25	6.05
	C(5)	1.06	6.59	4.93
	C(6)	-0.03	12.40	6.03
	C(7)	-0.13	12.71	6.13
	C(8)	0.02	12.38	5.97
	C(9)	-0.02	12.51	6.02
	C(10)	-0.17	11.20	6.17
	C(11)	-0.16	11.08	6.16
	H(6)	0.14	6.56	0.86
	H(7)	0.16	6.54	0.83
	H(8)	0.08	6.82	0.91
	H(9)	0.11	6.73	0.88
	H(10C)	0.12	6.34	0.87
	H(10B)	0.16	5.66	0.83
	H(10A)	0.12	6.38	0.87
	H(11A)	0.15	5.67	0.84
	H(11B)	0.12	6.19	0.87
	H(11C)	0.13	6.47	0.86
**Dataset Number**	**Atom**	**Q001** in e	**V001** in Å^3	**N001** in e
Dataset 16	**S(1)**	0.40	18.69	15.59
	**O(1)**	-1.29	19.65	9.29
	**O(2)**	-1.24	20.36	9.24
	**C(1)**	-0.26	12.01	6.26
	**C(2)**	1.06	6.50	4.94
	**C(3)**	-0.22	10.76	6.22

	**C(4)**	-0.03	10.15	6.03
	**C(5)**	1.14	6.54	4.85
	**C(6)**	-0.04	12.48	6.04
	**C(7)**	-0.13	12.68	6.13
	**C(8)**	0.06	12.13	5.93
	**C(9)**	0.04	12.01	5.95
	**C(10)**	-0.15	11.22	6.15
	**C(11)**	-0.04	10.82	6.04
	**H(6)**	0.15	6.36	0.84
	**H(7)**	0.15	6.56	0.84
	**H(8)**	0.11	6.61	0.88
	**H(9)**	0.10	6.70	0.89
	**H(10C)**	0.10	6.29	0.89
	**H(10B)**	0.11	5.93	0.88
	**H(10A)**	0.14	5.71	0.85
	**H(11A)**	0.10	6.42	0.89
	**H(11B)**	0.10	6.38	0.89
	**H(11C)**	0.07	6.41	0.92
**Dataset Number**	**Atom**	**Q001** in e	**V001** in Å^3	**N001** in e
Dataset 17	S(1)	0.46	18.90	15.53
	O(1)	-1.21	20.20	9.21
	O(2)	-1.29	20.54	9.29
	C(1)	-0.27	11.96	6.27
	C(2)	1.08	6.57	4.91
	C(3)	-0.10	10.45	6.10
	C(4)	-0.03	10.30	6.03
	C(5)	1.07	6.68	4.92
	C(6)	-0.01	12.28	6.00
	C(7)	-0.07	12.70	6.07
	C(8)	-0.03	12.38	6.03
	C(9)	0.01	12.10	5.98
	C(10)	-0.16	10.87	6.16
	C(11)	-0.12	10.81	6.12
	H(6)	0.10	6.76	0.89
	H(7)	0.10	6.68	0.89
	H(8)	0.14	6.76	0.85
	H(9)	0.10	6.76	0.89
	H(10C)	0.11	6.42	0.88
	H(10B)	0.11	5.70	0.88
	H(10A)	0.12	6.29	0.87
	H(11A)	0.12	6.38	0.87

	H(11B)	0.07	6.49	0.92
	H(11C)	0.12	6.22	0.87
**Dataset Number**	**Atom**	**Q001** in e	**V001** in Å^3	**N001** in e
Dataset 18	S(1)	0.41	19.13	15.56
	O(1)	-1.30	21.23	9.27
	O(2)	-1.29	22.18	9.24
	C(1)	-0.26	12.65	6.25
	C(2)	1.11	6.73	4.88
	C(3)	0.01	10.65	5.97
	C(4)	0.03	10.51	5.95
	C(5)	1.03	6.85	4.95
	C(6)	-0.01	12.74	5.99
	C(7)	-0.09	13.09	6.07
	C(8)	-0.02	12.91	6.00
	C(9)	-0.05	12.99	6.03
	C(10)	-0.04	11.01	6.03
	C(11)	-0.16	11.58	6.14
	H(6)	0.03	7.22	0.94
	H(7)	0.03	7.55	0.93
	H(8)	0.05	7.30	0.92
	H(9)	0.04	7.33	0.93
	H(10C)	0.07	6.08	0.89
	H(10B)	0.04	6.61	0.90
	H(10A)	0.09	6.76	0.93
	H(11A)	0.12	6.47	0.87
	H(11B)	0.10	6.64	0.93
	H(11C)	0.04	6.80	0.86
**Dataset Number**	**Atom**	**Q001** in e	**V001** in Å^3	**N001** in e
Dataset 19	S(1)	0.50	17.46	15.47
	O(1)	-1.23	19.57	9.19
	O(2)	-1.25	20.27	9.20
	C(1)	-0.17	11.75	6.16
	C(2)	0.92	7.02	5.06
	C(3)	-0.05	10.30	6.04
	C(4)	0.11	9.88	5.87
	C(5)	0.99	6.78	5.00
	C(6)	-0.07	12.25	6.05
	C(7)	-0.06	12.40	6.04
	C(8)	-0.02	12.30	6.01
	C(9)	-0.21	12.74	6.19
	C(10)	-0.20	11.46	6.19

	C(11)	-0.35	11.78	6.34
	H(6)	0.08	6.68	0.89
	H(7)	0.11	6.72	0.86
	H(8)	0.09	6.54	0.88
	H(9)	0.11	6.56	0.86
	H(10C)	0.10	6.33	0.87
	H(10B)	0.06	6.87	0.91
	H(10A)	0.14	5.78	0.84
	H(11A)	0.13	5.71	0.82
	H(11B)	0.11	6.31	0.84
	H(11C)	0.16	6.40	0.86
**Dataset Number**	**Atom**	**Q001** in e	**V001** in Å^3	**N001** in e
Dataset 20	S(1)	0.49	18.73	15.47
	O(1)	-1.30	20.86	9.26
	O(2)	-1.25	21.65	9.20
	C(1)	-0.23	12.36	6.21
	C(2)	1.06	6.82	4.92
	C(3)	-0.09	11.05	6.08
	C(4)	-0.03	10.53	6.02
	C(5)	1.04	6.98	4.94
	C(6)	0.02	12.58	5.95
	C(7)	-0.05	13.07	6.03
	C(8)	-0.01	12.86	5.99
	C(9)	-0.02	12.69	6.00
	C(10)	-0.30	11.70	6.29
	C(11)	-0.17	11.29	6.16
	H(6)	0.05	7.23	0.91
	H(7)	0.08	7.29	0.89
	H(8)	0.05	7.21	0.91
	H(9)	0.07	7.17	0.90
	H(10C)	0.09	6.68	0.88
	H(10B)	0.07	6.59	0.90
	H(10A)	0.17	5.79	0.81
	H(11A)	0.10	6.29	0.87
	H(11B)	0.05	6.90	0.92
	H(11C)	0.08	6.86	0.88
**Dataset Number**	**Atom**	**Q001** in e	**V001** in Å^3	**N001** in e
Dataset 21	S(1)	0.43	18.48	15.53
	O(1)	-1.29	21.66	9.24
	O(2)	-1.29	20.52	9.26
	C(1)	-0.20	12.09	6.19

	C(2)	1.05	6.72	4.93
	C(3)	-0.01	10.38	6.01
	C(4)	0.02	10.22	5.96
	C(5)	1.10	6.71	4.89
	C(6)	-0.07	12.69	6.05
	C(7)	-0.08	12.86	6.06
	C(8)	-0.02	12.64	6.01
	C(9)	-0.09	12.98	6.07
	C(10)	-0.21	11.48	6.20
	C(11)	-0.21	11.33	6.20
	H(6)	0.08	7.09	0.89
	H(7)	0.08	7.13	0.88
	H(8)	0.08	6.97	0.89
	H(9)	0.09	6.86	0.88
	H(10C)	0.09	6.46	0.88
	H(10B)	0.07	5.80	0.89
	H(10A)	0.14	6.65	0.84
	H(11A)	0.10	6.47	0.88
	H(11B)	0.03	7.03	0.86
	H(11C)	0.11	6.15	0.94
**Dataset Number**	**Atom**	**Q001** in e	**V001** in Å^3	**N001** in e
Dataset 22	S(1)	0.90	17.05	15.07
	O(1)	-1.28	21.58	9.23
	O(2)	-1.30	20.57	9.26
	C(1)	-0.13	11.90	6.12
	C(2)	0.97	6.86	5.02
	C(3)	-0.01	10.47	5.99
	C(4)	-0.08	10.54	6.07
	C(5)	1.10	6.63	4.88
	C(6)	0.01	12.33	5.96
	C(7)	-0.09	12.99	6.07
	C(8)	-0.01	12.57	5.99
	C(9)	-0.03	12.86	6.01
	C(10)	-0.76	12.65	6.75
	C(11)	-0.08	11.01	6.06
	H(6)	0.09	7.02	0.88
	H(7)	0.08	7.29	0.88
	H(8)	0.05	7.06	0.91
	H(9)	0.05	6.83	0.92
	H(10C)	0.12	6.28	0.86
	H(10B)	0.06	6.71	0.85

	H(10A)	0.12	5.84	0.91
	H(11A)	0.09	6.38	0.86
	H(11B)	0.11	6.52	0.98
	H(11C)	-0.01	7.23	0.89
**Dataset Number**	**Atom**	**Q001** in e	**V001** in Å^3	**N001** in e
Dataset 23	S(1)	0.50	18.09	15.46
	O(1)	-1.28	20.26	9.25
	O(2)	-1.27	21.24	9.23
	C(1)	-0.18	11.78	6.16
	C(2)	1.01	6.77	4.97
	C(3)	-0.07	10.54	6.06
	C(4)	0.03	10.10	5.95
	C(5)	1.01	6.73	4.97
	C(6)	0.03	12.14	5.94
	C(7)	-0.08	12.79	6.06
	C(8)	0.01	12.18	5.97
	C(9)	-0.13	12.88	6.11
	C(10)	-0.27	11.77	6.26
	C(11)	-0.30	11.81	6.28
	H(6)	0.06	6.96	0.91
	H(7)	0.09	6.95	0.88
	H(8)	0.06	6.93	0.91
	H(9)	0.09	6.74	0.88
	H(10C)	0.11	6.55	0.86
	H(10B)	0.04	6.08	0.83
	H(10A)	0.15	6.71	0.93
	H(11A)	0.12	5.55	0.81
	H(11B)	0.06	6.09	0.85
	H(11C)	0.17	6.44	0.91
**Dataset Number**	**Atom**	**Q001** in e	**V001** in Å^3	**N001** in e
Theoretical calculation, opt	**S(1)**	0.45	19.36	15.54
	**O(1)**	-1.18	19.74	9.18
	**O(2)**	-1.15	20.48	9.15
	**C(1)**	-0.32	12.03	6.32
	**C(2)**	1.07	6.77	4.92
	**C(3)**	-0.04	10.35	6.04
	**C(4)**	-0.04	10.37	6.04
	**C(5)**	1.03	6.85	4.96
	**C(6)**	0.01	12.08	5.99
	**C(7)**	-0.01	12.17	6.01
	**C(8)**	-0.01	12.18	6.01

	**C(9)**	0.00	12.09	5.99
	**C(10)**	-0.13	10.89	6.13
	**C(11)**	-0.13	10.90	6.13
	**H(6)**	0.08	6.91	0.91
	**H(7)**	0.06	7.02	0.93
	**H(8)**	0.06	7.03	0.93
	**H(9)**	0.08	6.94	0.91
	**H(10C)**	0.09	6.01	0.90
	**H(11C)**	0.12	6.66	0.87
	**H(11B)**	0.07	6.84	0.92
	**H(10B)**	0.09	6.02	0.90
	**H(11A)**	0.07	6.83	0.92
	**H(10A)**	0.12	6.67	0.87

Table S12. Electron density (e/Å^3) and Laplacian of the electron density (e/Å^5) at the bond critical points of selected bonds from multipole refinement vs. XWR with the average across data sets including sample standard deviation. The last row shows the RMSD (root mean square deviation) for all selected bonds per dataset relative to the corresponding values from theory (opt, data can be found in Table S9, last part).

**Bond (electron density)**	**Dataset 1**	**Dataset 3**	**Dataset 16**	**Dataset 17**	**Average**	**Standard deviation**
**Multipole Model**						
O(1)-C(2)	2.77	2.80	2.69	2.84	2.77	0.06
C(2)-C(3)	1.76	1.79	1.89	1.79	1.81	0.05
C(1)-S(1)	1.48	1.50	1.47	1.47	1.48	0.01
C(11)-S(1)	1.30	1.29	1.27	1.35	1.30	0.03
C(6)-H(6)	1.85	1.76	1.79	1.72	1.78	0.05
C(10)-H(10A)	1.74	1.67	1.74	1.81	1.74	0.05
C(7)-C(8)	2.07	2.14	2.03	2.07	2.08	0.04
**RMSD (opt)**	0.13	0.16	0.42	0.16	0.15	-
**XWR**						
O(1)-C(2)	2.68	2.68	2.72	2.75	2.71	0.03
C(2)-C(3)	1.84	1.83	1.84	1.80	1.83	0.02
C(1)-S(1)	1.55	1.54	1.55	1.56	1.55	0.01
C(11)-S(1)	1.39	1.34	1.38	1.34	1.368	0.02
C(6)-H(6)	1.93	1.94	1.87	1.90	1.91	0.03
C(10)-H(10A)	1.96	1.91	1.95	1.92	1.93	0.02
C(7)-C(8)	2.15	2.16	2.14	2.16	2.15	0.01
**RMSD (opt)**	0.10	0.09	0.11	0.09	0.10	-

Bond (Laplacian)	Dataset 1	Dataset 3	Dataset 16	Dataset 17	Average	Standard deviation
**Multipole Model**						
O(1)-C(2)	-30.4	-31.7	-26.5	-35.1	-30.9	3.5
C(2)-C(3)	-12.2	-15.1	-17.4	-12.3	-14.2	2.5
C(1)-S(1)	-7.4	-8.6	-6.2	-8.1	-7.6	1.0
C(11)-S(1)	-5.7	-5.5	-0.9	-8.3	-5.1	3.1
C(6)-H(6)	-18.1	-14.7	-17.9	-15.6	-16.6	1.6
C(10)-H(10A)	-18.2	-16.5	-14.6	-18.1	-16.8	1.6
C(7)-C(8)	-17.5	-21.8	-18.8	-17.1	-18.8	2.1
**RMSD (opt)**	10.7	11.4	10.7	12.5	11.1	-
**XWR**						
O(1)-C(2)	-1.5	-4.3	-2.7	-10.5	-4.8	4.0
C(2)-C(3)	-19.2	-18.5	-19.4	-17.2	-18.6	0.9
C(1)-S(1)	-12.7	-12.2	-12.7	-12.5	-12.5	0.2
C(11)-S(1)	-11.1	-9.1	-11.2	-9.1	-10.1	1.1
C(6)-H(6)	-25.9	-25.8	-24.4	-25.1	-25.3	0.6
C(10)-H(10A)	-25.6	-24.9	-24.9	-24.3	-24.9	0.5
C(7)-C(8)	-23.7	-24.1	-23.2	-23.9	-23.7	0.3
**RMSD (opt)**	2.0	1.1	1.7	2.3	1.3	-

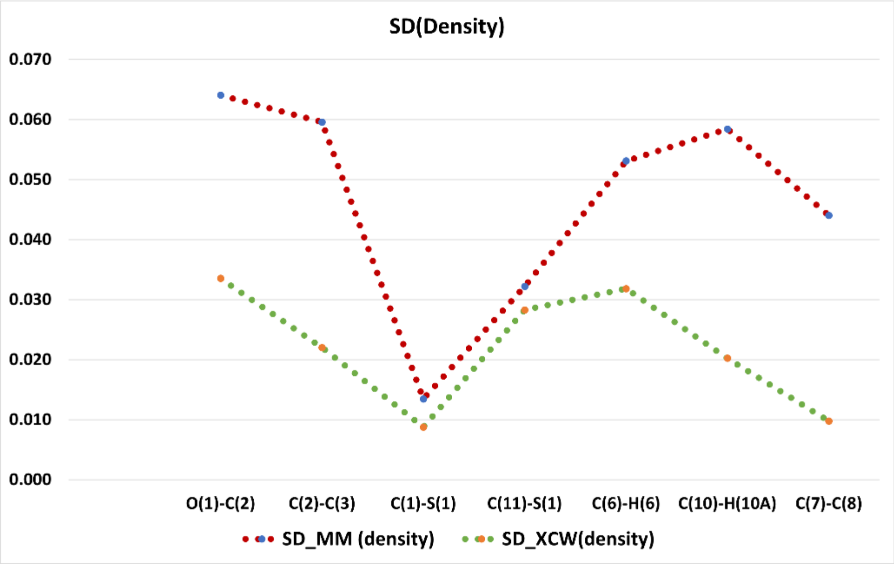

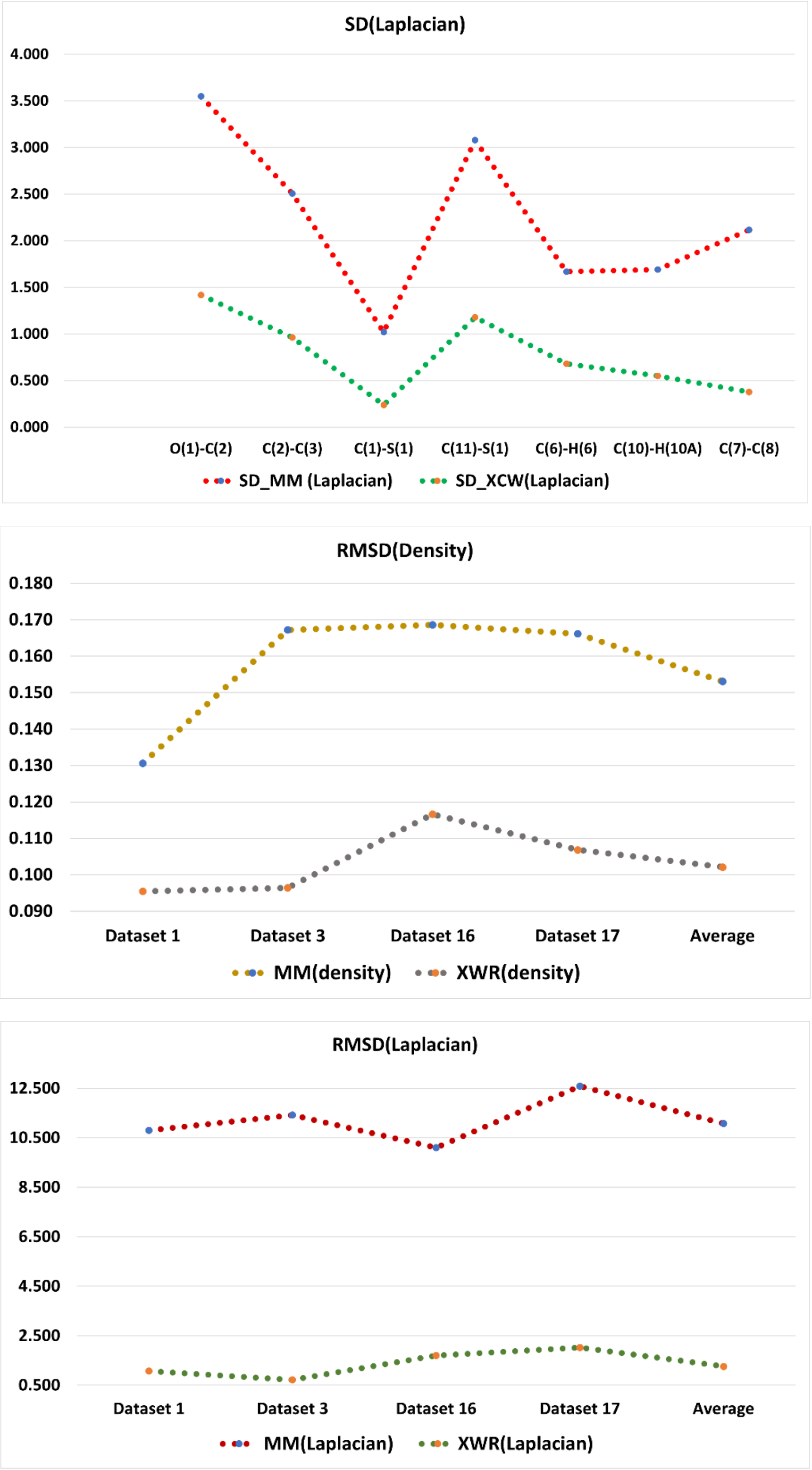

Figure S14. Graphical representation of the standard deviation (SD) per selected bond (last column in Table S12) and root mean square deviation relative to theory per selected dataset (last row in Table S12) for the electron density (e/Å^3) and the Laplacian of the electron density (e/Å^5) at the bond critical points as a comparison of multipole model and XWR.

Table S13. Electron density (e/Å^3) and the Laplacian of the electron density (e/Å^5) at the bond critical points of selected bonds from XWR with the average across all data sets including sample standard deviation. The last row shows the RMSD (root mean square deviation) for all selected bonds per dataset relative to the corresponding values from theory (opt, which can be found in Table S9, last part).

**Bond (electron density)**	**Dataset 1**	**Dataset 2**	**Dataset 3**	**Dataset 4**	**Dataset 5**
O(1)-C(2)	2.68	2.72	2.75	2.73	2.72
C(2)-C(3)	1.83	1.76	1.80	1.82	1.83
C(1)-S(1)	1.54	1.54	1.56	1.53	1.62
C(11)-S(1)	1.34	1.35	1.34	1.33	1.37
C(6)-H(6)	1.94	1.94	1.90	1.95	1.94
C(10)-H(10A)	1.91	1.90	1.92	1.92	1.92
C(7)-C(8)	2.16	2.11	2.16	2.16	2.22
**RMSD (opt)**	0.09	0.08	0.09	0.08	0.10
**Bond (electron density)**	**Dataset 6**	**Dataset 7**	**Dataset 8**	**Dataset 9**	**Dataset 10**
O(1)-C(2)	2.75	2.73	2.79	2.69	2.73
C(2)-C(3)	1.84	1.80	1.82	2.72	1.81
C(1)-S(1)	1.55	1.52	1.56	1.54	1.54
C(11)-S(1)	1.33	1.34	1.38	1.36	1.36
C(6)-H(6)	2.01	1.97	1.92	1.94	1.91
C(10)-H(10A)	1.90	1.97	2.02	1.92	1.92
C(7)-C(8)	2.20	2.15	2.14	2.16	2.16
**RMSD (opt)**	0.06	0.08	0.10	0.37	0.09
**Bond (electron density)**	**Dataset 11**	**Dataset 12**	**Dataset 13**	**Dataset 13**	**Dataset 15**
O(1)-C(2)	2.66	2.70	2.65	2.72	2.71
C(2)-C(3)	1.84	1.85	1.84	1.84	1.75
C(1)-S(1)	2.20	1.53	1.54	1.52	1.53
C(11)-S(1)	1.39	1.37	1.44	1.30	1.35
C(6)-H(6)	1.96	1.98	1.91	1.93	1.90
C(10)-H(10A)	2.11	2.17	1.91	1.89	1.94
C(7)-C(8)	2.16	2.14	2.20	2.19	2.18
**RMSD (opt)**	0.27	0.13	0.12	0.09	0.10
**Bond (electron density)**	**Dataset 16**	**Dataset 17**	**Dataset 18**	**Dataset 19**	**Dataset 20**
O(1)-C(2)	2.72	2.68	2.58	2.73	2.64
C(2)-C(3)	1.84	1.84	1.76	1.78	1.77
C(1)-S(1)	1.55	1.55	1.48	1.28	1.42
C(11)-S(1)	1.38	1.39	1.20	1.32	1.28
C(6)-H(6)	1.87	1.93	1.97	1.97	1.86
C(10)-H(10A)	1.95	1.96	1.89	1.87	1.93
C(7)-C(8)	2.14	2.15	2.13	2.21	2.13

RMSD (opt)	0.11	0.10	0.11	0.12	0.13
**Bond (electron density)**	**Dataset 21**	**Dataset 22**	**Dataset 23**	**Average**	**Standard Deviation**
O(1)-C(2)	2.60	2.67	2.68	2.70	0.04
C(2)-C(3)	1.77	1.80	1.75	1.85	0.19
C(1)-S(1)	1.45	1.40	1.38	1.54	0.16
C(11)-S(1)	1.32	1.25	1.31	1.34	0.05
C(6)-H(6)	1.87	1.89	1.98	1.93	0.03
C(10)-H(10A)	1.96	1.93	1.77	1.94	0.07
C(7)-C(8)	2.15	2.15	2.15	2.16	0.02
**RMSD (opt)**	0.13	0.12	0.11	0.09	-
**Bond(Laplacian)**	**Dataset 1**	**Dataset 2**	**Dataset 3**	**Dataset 4**	**Dataset 5**
O(1)-C(2)	-4.3	-9.5	-10.5	-1.6	-7.4
C(2)-C(3)	-18.5	-17.1	-17.2	-18.6	-18.8
C(1)-S(1)	-12.2	-12.7	-12.5	-12.3	-14.4
C(11)-S(1)	-9.1	-9.9	-9.1	-9.4	-10.2
C(6)-H(6)	-25.8	-25.9	-25.1	-26.0	-25.4
C(10)-H(10A)	-24.9	-25.2	-24.3	-25.2	-25.1
C(7)-C(8)	-24.1	-21.3	-23.9	-24.5	-26.5
**RMSD (opt)**	1.1	2.1	2.3	1.7	2.3
**Bond(Laplacian)**	**Dataset 6**	**Dataset 7**	**Dataset 8**	**Dataset 9**	**Dataset 10**
O(1)-C(2)	-5.7	-6.1	-11.4	2.1	-8.5
C(2)-C(3)	-18.4	-17.9	-18.6	-1.2	-18.3
C(1)-S(1)	-13.3	-13.0	-11.5	-12.3	-12.8
C(11)-S(1)	-9.9	-10.7	-9.4	-10.0	-9.5
C(6)-H(6)	-27.7	-26.7	-25.8	-26.1	-25.2
C(10)-H(10A)	-24.6	-26.1	-27.3	-25.2	-25.2
C(7)-C(8)	-25.7	-24.0	-23.8	-24.2	-24.1
**RMSD (opt)**	1.9	1.6	3.0	6.5	1.8
**Bond(Laplacian)**	**Dataset 11**	**Dataset 12**	**Dataset 13**	**Dataset 14**	**Dataset 15**
O(1)-C(2)	1.1	-2.4	6.8	1.4	-6.5
C(2)-C(3)	-19.6	-19.7	-19.7	-19.6	-15.7
C(1)-S(1)	-25.1	-11.4	-12.3	-11.9	-12.5
C(11)-S(1)	-10.8	-10.1	-13.1	-8.2	-9.8
C(6)-H(6)	-26.7	-27.6	-25.5	-26.7	-25.3
C(10)-H(10A)	-24.7	-22.9	-24.1	-23.9	-24.4
C(7)-C(8)	-24.4	-23.3	-26.4	-26.5	-24.6
**RMSD (opt)**	5.7	1.9	5.0	3.1	1.3
**Bond(Laplacian)**	**Dataset 16**	**Dataset 17**	**Dataset 18**	**Dataset 19**	**Dataset 20**
O(1)-C(2)	-2.7	-1.5	-1.6	-4.0	0.6
C(2)-C(3)	-19.4	-19.2	-17.1	-16.2	-16.8

C(1)-S(1)	-12.7	-12.7	-11.4	-10.5	-8.3
C(11)-S(1)	-11.2	-11.1	-9.4	-9.1	-8.1
C(6)-H(6)	-24.4	-25.9	-26.8	-26.3	-23.9
C(10)-H(10A)	-24.9	-25.6	-23.6	-21.8	-25.8
C(7)-C(8)	-23.2	-23.7	-24.0	-25.2	-23.7
**RMSD (opt)**	1.7	2.0	1.5	1.5	2.5
**Bond(Laplacian)**	**Dataset 21**	**Dataset 22**	**Dataset 23**	**Average**	**Standard Deviation**
O(1)-C(2)	-6.2	0.1	-3.5	-3.5	4.5
C(2)-C(3)	-17.1	-17.7	-16.1	-17.3	3.7
C(1)-S(1)	-9.4	-6.6	-5.6	-12.1	3.5
C(11)-S(1)	-10.0	-5.7	-8.1	-9.6	1.4
C(6)-H(6)	-24.6	-25.2	-27.2	-25.9	0.9
C(10)-H(10A)	-26.3	-25.4	-20.6	-24.6	1.4
C(7)-C(8)	-24.3	-24.5	-24.0	-24.3	1.1
**RMSD (opt)**	1.6	3.0	2.9	1.2	-

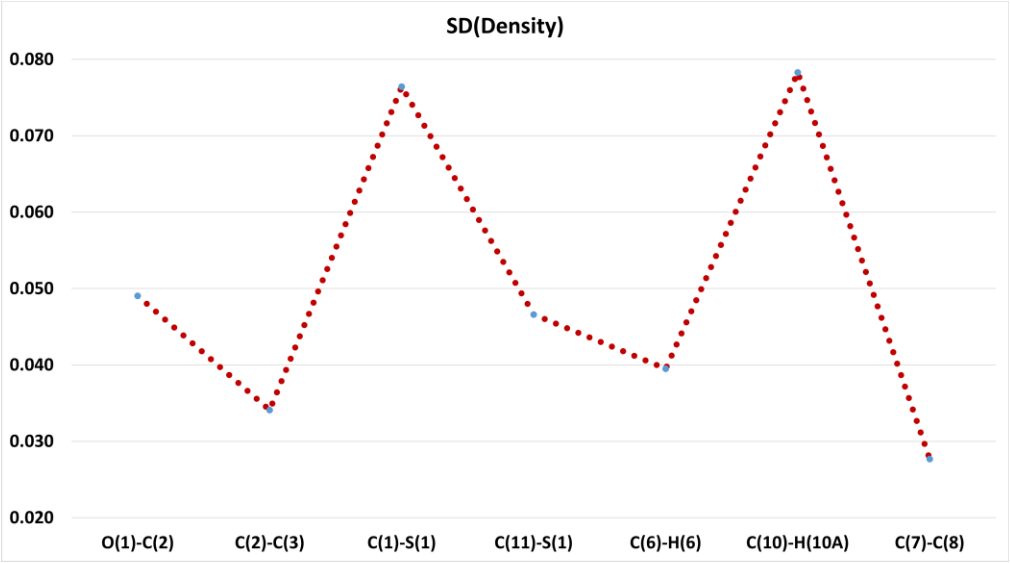

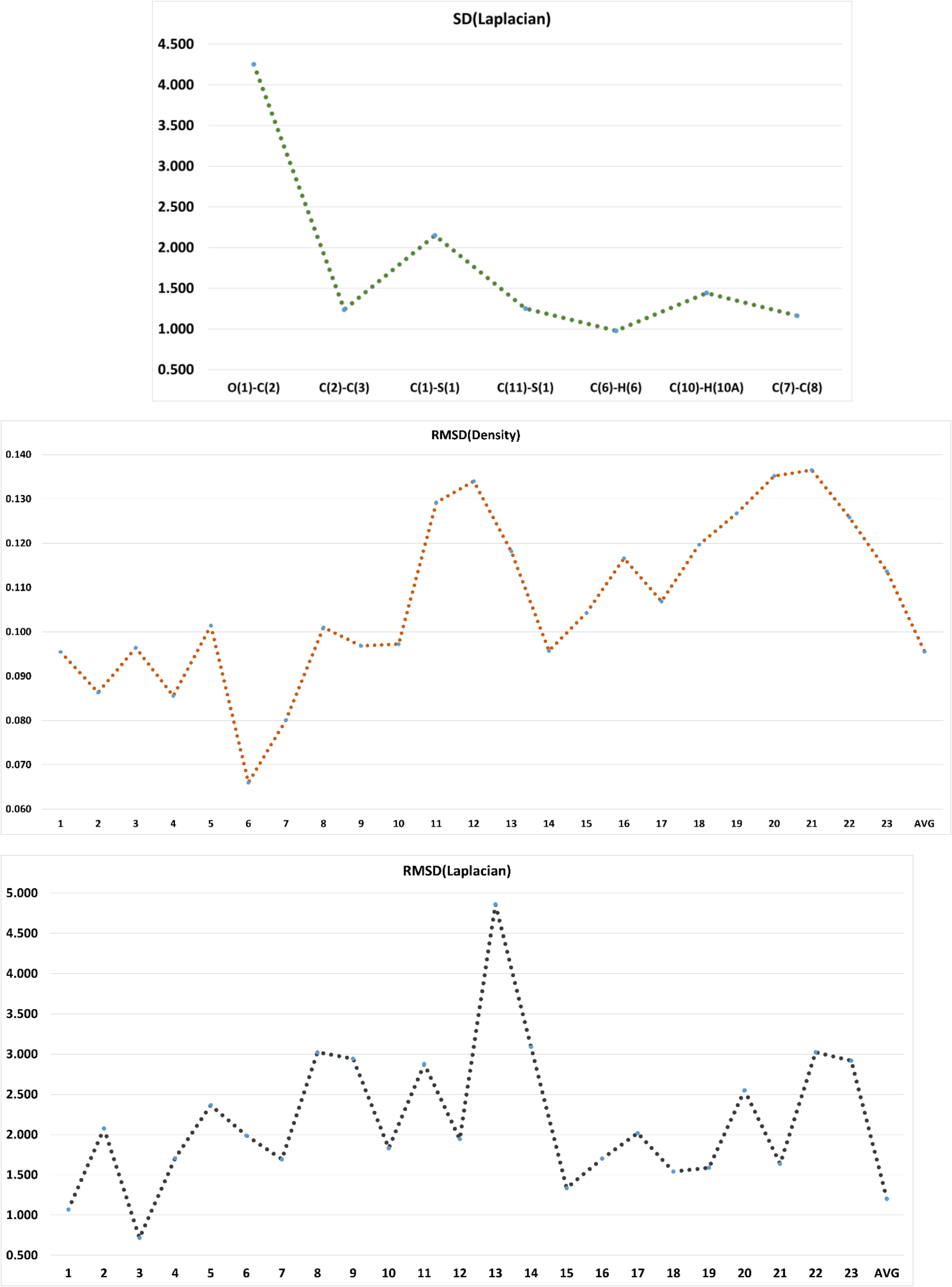

Figure S15. Graphical representation of the standard deviation (SD) per selected bond (from Table S13) and root mean square deviation relative to theory for all dataset (from Table S13) for the electron density (e/Å^3) and the Laplacian of the electron density (e/Å^5) at the bond critical points from XWR.

Table S14. QTAIM atomic charge Q001 in e and atomic volume V001 in Å^3 (001 means that the atomic basin was cropped at an isovalue of the electron density of 0.001 a.u.) for selected atoms from multipole refinement vs. XWR with the average across data sets including sample standard deviation. The last row shows the RMSD (root mean square deviation) for all selected atoms per dataset relative to the corresponding values from theory (opt, which can be found in Table S11, last part).

Atom (Q001)	Dataset 1	Dataset 3	Dataset 16	Dataset 17	Average	Standard Deviation
**Multipole Model**						
S(1)	0.33	0.22	0.75	0.66	0.49	0.31
O(1)	-0.99	-1.02	-1.18	-1.1	-1.07	0.48
C(1)	-0.28	-0.30	-0.16	-0.34	-0.27	0.14
C(11)	-0.13	0.21	0.25	0.03	0.09	0.16
C(7)	0.11	0.26	0.31	-0.02	0.16	0.15
H(6)	0.02	0.04	0.09	0.11	0.06	0.04
H(10A)	0.23	0.22	0.11	0.18	0.18	0.09
**RMSD (opt)**	0.10	0.20	0.22	0.10	0.12	-
**Atom (Q001)**	**Dataset 1**	**Dataset 3**	**Dataset 16**	**Dataset 17**	**Average**	**Standard Deviation**
**XWR**						
S(1)	0.46	0.42	0.40	0.46	0.43	0.02
O(1)	-1.28	-1.25	-1.29	-1.21	-1.26	0.03
C(1)	-0.29	-0.30	-0.26	-0.27	-0.28	0.01
C(11)	-0.16	-0.04	-0.04	-0.12	-0.09	0.05
C(7)	-0.06	0.02	-0.13	-0.07	-0.05	0.06
H(6)	0.10	0.08	0.15	0.10	0.11	0.03
H(10A)	0.11	0.09	0.14	0.12	0.11	0.02
**RMSD (opt)**	0.04	0.04	0.08	0.03	0.04	-
**Atom (V001)**	**Dataset 1**	**Dataset 3**	**Dataset 16**	**Dataset 17**	**Average**	**Standard Deviation**
**Multipole Model**						
S(1)	18.90	19.95	18.15	18.34	18.83	8.45
O(1)	17.03	17.24	18.18	17.85	17.57	7.87
C(1)	11.12	11.29	11.22	11.68	11.33	5.07
C(11)	10.70	9.62	9.05	10.57	9.99	4.51
C(7)	11.52	11.33	10.02	11.70	11.14	5.02
H(6)	7.44	7.06	7.09	7.24	7.21	3.22
H(10A)	5.27	5.22	5.65	5.46	5.40	2.42
**RMSD (opt)**	1.25	1.28	1.39	0.97	1.13	-

Atom (V001)	Dataset 1	Dataset 3	Dataset 16	Dataset 17	Average	Standard Deviation
			**XWR**			
S(1)	18.64	19.43	18.69	18.90	18.92	0.36
O(1)	20.03	20.07	19.65	20.20	19.99	0.23
C(1)	12.16	12.76	12.01	11.96	12.22	0.36
C(11)	11.12	10.87	10.82	10.81	10.91	0.14
C(7)	12.60	12.83	12.33	12.70	12.62	0.21
H(6)	6.83	7.31	5.71	6.76	6.65	0.67
H(10A)	6.31	5.98	6.16	6.29	6.18	0.14
**RMSD (opt)**	0.37	0.49	0.55	0.35	0.34	-

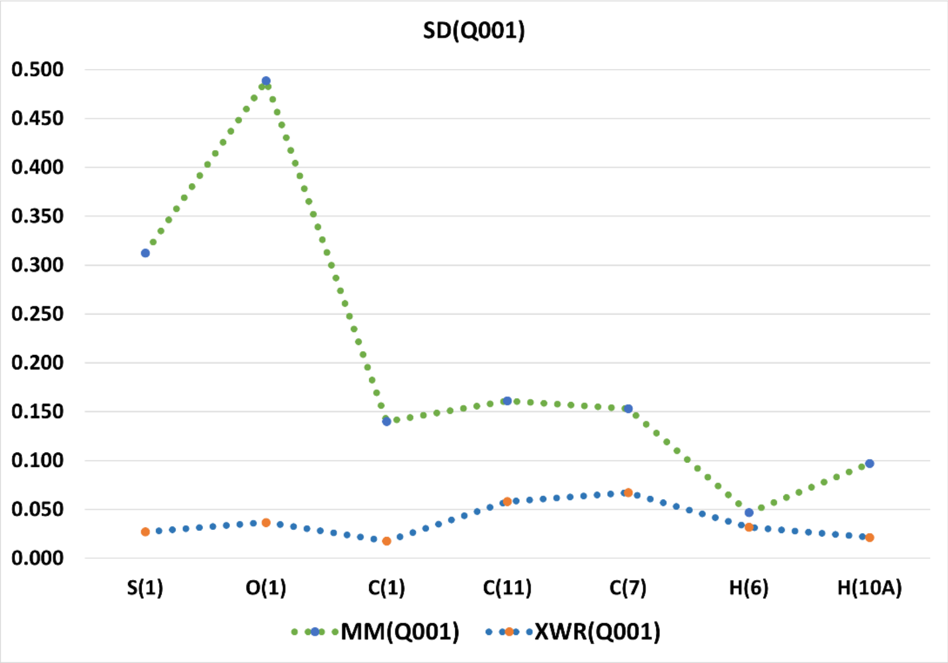

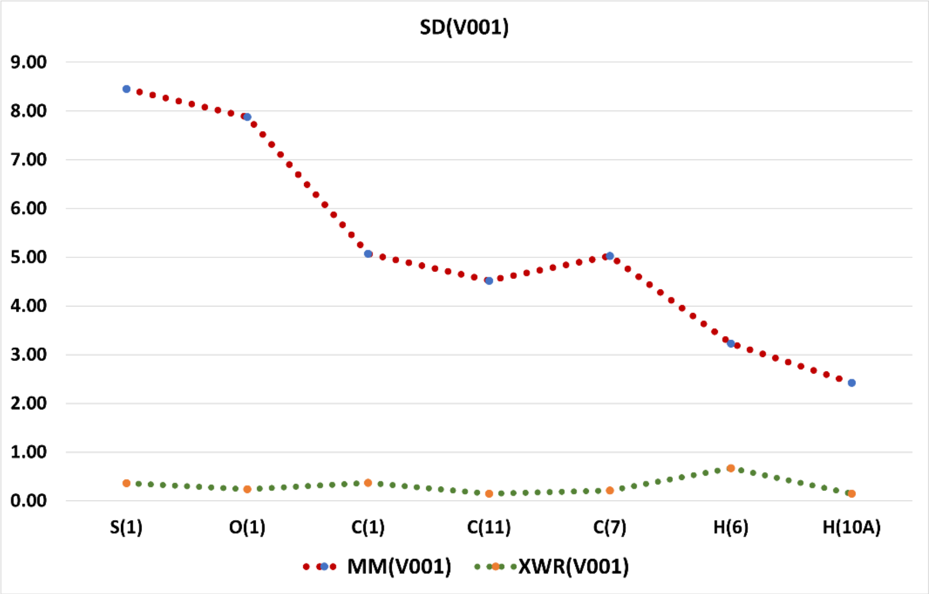
SD(V001)
9.00
8.00
7.00
6.00
5.00
4.00
3.00
2.00
1.00
0.00
S(1)
O(1)
C(1)
C(11)
C(7)
H(6)
H(10A)
MM(V001)
XWR(V001)

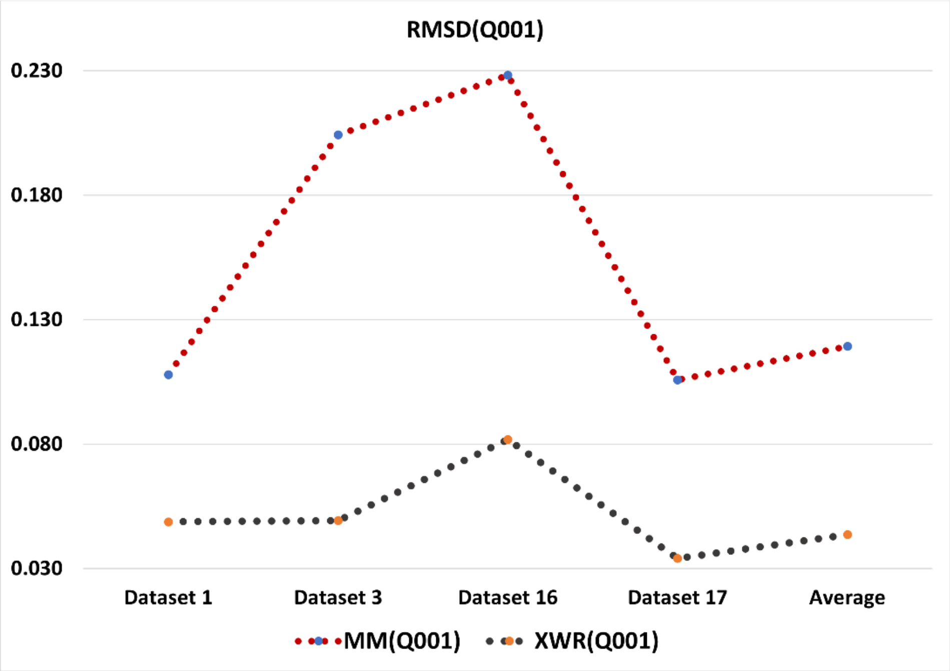
RMSD(Q001)
0.230
0.180
0.130
0.080
0.030
Dataset 1
Dataset 3
Dataset 16
Dataset 17
Average
MM(Q001)
XWR(Q001)

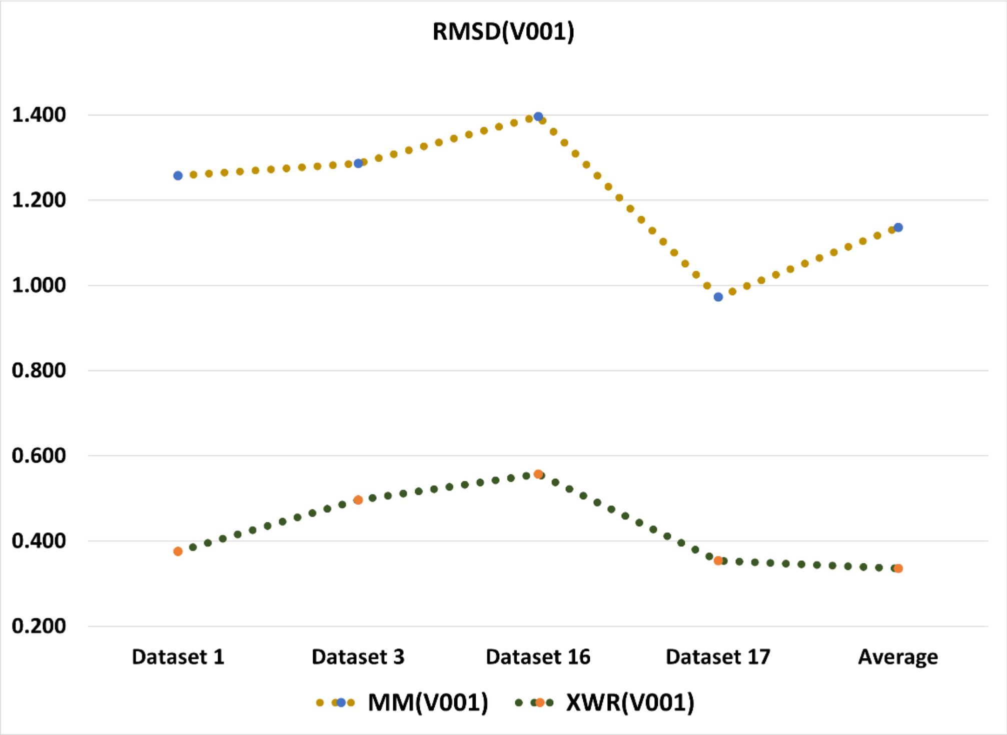

Figure S16. Graphical representation of the standard deviation (SD) per selected atom (last column in Table S14) and root mean square deviation relative to theory per selected dataset (last row in Table S14) for the atomic charge (in e) and atomic volume (in Å^3) as a comparison of multipole model and XWR.

Table S15. QTAIM atomic charge Q001 in e and atomic volume V001 in Å^3 (001 means that the atomic basin was cropped at an isovalue of the electron density of 0.001 a.u.) for selected atoms from XWR with the average across all datasets including sample standard deviation. The last row shows the RMSD (root mean square deviation) for all selected atoms per dataset relative to the corresponding values from theory (opt, which can be found in Table S11, last part).

**Atom(Q001)**	**Dataset 1**	**Dataset 2**	**Dataset 3**	**Dataset 4**	**Dataset 5**
S(1)	0.46	0.24	0.42	0.42	0.26
O(1)	-1.28	-1.17	-1.25	-1.31	-1.34
C(1)	-0.29	-0.18	-0.30	-0.27	-0.17
C(11)	-0.16	-0.18	-0.04	-0.11	-0.11
C(7)	-0.06	-0.11	0.02	-0.10	-0.09
H(6)	0.10	0.13	0.08	0.07	0.11
H(10A)	0.11	0.11	0.09	0.11	0.10
**RMSD (opt)**	0.04	0.10	0.04	0.06	0.11
**Atom(Q001)**	**Dataset 6**	**Dataset 7**	**Dataset 8**	**Dataset 9**	**Dataset 10**
S(1)	0.50	0.54	0.84	0.41	0.26
O(1)	-1.30	-1.28	-1.26	-1.28	-1.21

C(1)	-0.41	-0.37	-0.21	-0.25	-0.21
C(11)	-0.04	-0.10	-0.02	-0.14	-0.13
C(7)	-0.11	-0.11	-0.03	-0.08	-0.10
H(6)	0.02	0.03	0.07	0.10	0.10
H(10A)	0.10	0.16	0.05	0.11	0.10
**RMSD (opt)**	0.08	0.07	0.16	0.05	0.09
**Atom(Q001)**	**Dataset 11**	**Dataset 12**	**Dataset 13**	**Dataset 14**	**Dataset 15**
S(1)	0.32	0.31	0.32	0.56	0.28
O(1)	-1.33	-1.26	-1.33	-1.35	-1.19
C(1)	-0.20	-0.15	-0.20	-0.22	-0.27
C(11)	-0.09	-0.09	-0.09	-0.10	-0.16
C(7)	-0.12	-0.02	-0.12	-0.17	-0.13
H(6)	0.10	0.09	0.10	0.08	0.14
H(10A)	0.10	0.13	0.10	0.08	0.12
**RMSD (opt)**	0.09	0.08	0.09	0.10	0.08
**Atom(Q001)**	**Dataset 16**	**Dataset 17**	**Dataset 18**	**Dataset 19**	**Dataset 20**
S(1)	0.40	0.46	0.41	0.50	0.49
O(1)	-1.29	-1.21	-1.30	-1.23	-1.30
C(1)	-0.26	-0.27	-0.26	-0.17	-0.23
C(11)	-0.04	-0.12	-0.16	-0.35	-0.17
C(7)	-0.13	-0.07	-0.09	-0.06	-0.05
H(6)	0.15	0.10	0.03	0.08	0.05
H(10A)	0.14	0.12	0.09	0.14	0.17
**RMSD (opt)**	0.08	0.03	0.06	0.10	0.06
**Atom(Q001)**	**Dataset 21**	**Dataset 22**	**Dataset 23**	**Average**	**Standard Deviation**
S(1)	0.43	0.90	0.50	0.45	0.16
O(1)	-1.29	-1.28	-1.28	-1.28	0.04
C(1)	-0.20	-0.13	-0.18	-0.23	0.06
C(11)	-0.21	-0.08	-0.30	-0.13	0.07
C(7)	-0.08	-0.09	-0.08	-0.08	0.04
H(6)	0.08	0.09	0.06	0.08	0.03
H(10A)	0.14	0.12	0.15	0.11	0.02
**RMSD (opt)**	0.07	0.19	0.09	0.06	-
**Atom(V001)**	**Dataset 1**	**Dataset 2**	**Dataset 3**	**Dataset 4**	**Dataset 5**
S(1)	18.64	19.28	19.43	18.55	17.80
O(1)	20.03	20.39	20.07	20.11	19.48
C(1)	12.16	12.01	12.76	11.99	11.66
C(11)	11.12	11.32	10.87	10.84	10.86
C(7)	12.60	12.94	12.83	12.68	12.39
H(6)	6.83	6.63	7.31	6.90	6.50

H(10A)	6.31	6.77	5.98	5.73	5.47
**RMSD (opt)**	0.37	0.42	0.49	0.52	0.77
**Atom(V001)**	**Dataset 6**	**Dataset 7**	**Dataset 8**	**Dataset 9**	**Dataset 10**
S(1)	17.90	18.12	18.01	18.84	19.46
O(1)	20.56	20.88	20.67	20.59	20.41
C(1)	12.14	11.85	11.64	12.07	11.88
C(11)	10.43	10.51	10.54	11.09	11.12
C(7)	12.33	12.67	12.45	12.76	12.86
H(6)	7.04	7.19	6.94	6.92	6.79
H(10A)	5.64	6.58	6.86	5.77	5.75
**RMSD (opt)**	0.76	0.69	0.66	0.55	0.51
**Atom(V001)**	**Dataset 11**	**Dataset 12**	**Dataset 13**	**Dataset 14**	**Dataset 15**
S(1)	18.99	19.04	18.99	19.08	18.87
O(1)	20.21	20.85	20.21	20.92	20.21
C(1)	11.98	11.73	11.98	12.37	11.91
C(11)	10.91	11.05	10.91	11.21	11.08
C(7)	12.83	12.33	12.83	13.29	12.71
H(6)	6.83	6.73	6.83	7.16	6.56
H(10A)	6.30	6.16	6.30	6.97	6.38
**RMSD (opt)**	0.36	0.50	0.36	0.66	0.37
**Atom(V001)**	**Dataset 16**	**Dataset 17**	**Dataset 18**	**Dataset 19**	**Dataset 20**
S(1)	18.69	18.90	19.13	17.46	18.73
O(1)	19.65	20.20	21.23	19.57	20.86
C(1)	12.01	11.96	12.65	11.75	12.36
C(11)	10.82	10.81	11.58	11.78	11.29
C(7)	6.36	12.70	13.09	12.40	13.07
H(6)	5.71	6.76	7.22	6.68	7.23
H(10A)	12.68	6.29	6.76	5.78	5.79
**RMSD (opt)**	0.55	0.35	0.76	0.87	0.71
**Atom(V001)**	**Dataset 21**	**Dataset 22**	**Dataset 23**	**Average**	**Standard Deviation**
S(1)	18.48	17.05	18.09	18.59	0.63
O(1)	21.66	21.58	20.26	20.46	0.57
C(1)	12.09	11.90	11.78	12.03	0.28
C(11)	11.33	11.01	11.81	11.06	0.36
C(7)	12.86	12.99	12.79	12.7	0.26
H(6)	7.09	7.02	6.96	6.86	0.33
H(10A)	6.65	5.84	6.71	6.21	0.44
**RMSD (opt)**	0.85	1.19	0.67	0.49	-

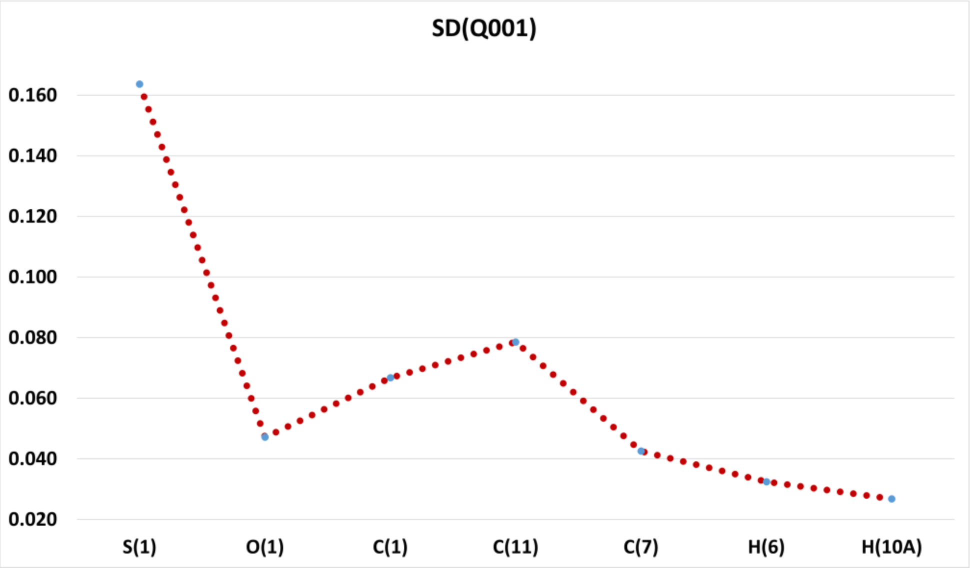
SD(Q001)
0.160
0.140
0.120
0.100
0.080
0.060
0.040
0.020
S(1)
O(1)
C(1)
C(11)
C(7)
H(6)
H(10A)

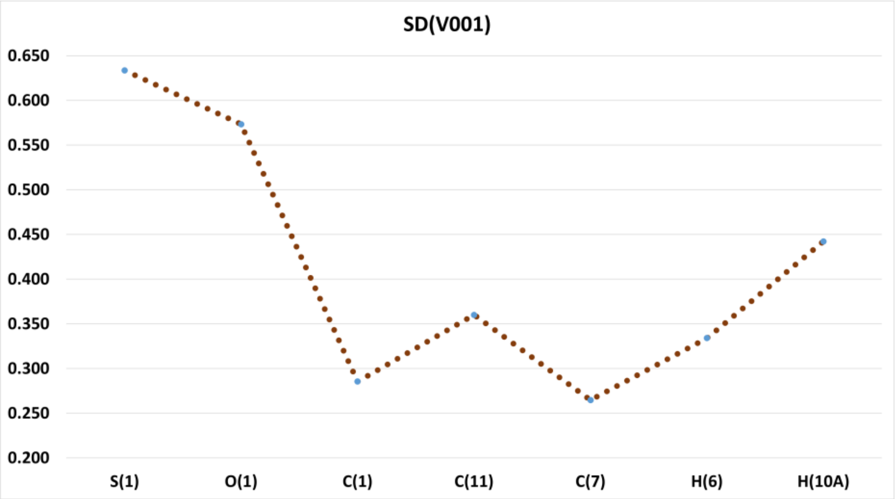
SD(V001)
0.650
0.600
0.550
0.500
0.450
0.400
0.350
0.300
0.250
0.200
S(1)
O(1)
C(1)
C(11)
C(7)
H(6)
H(10A)

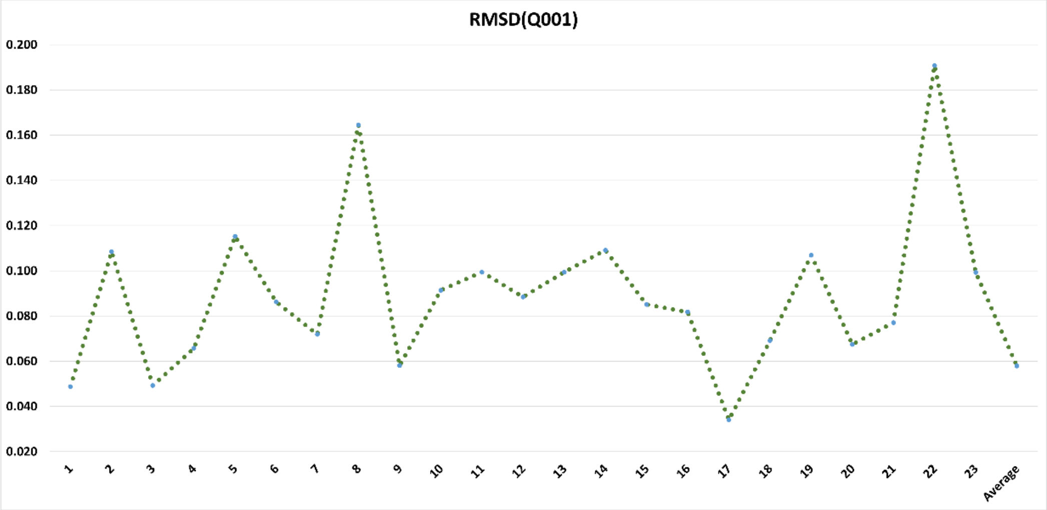
RMSD(Q001)
0.200
0.180
0.160
0.140
0.120
0.100
0.080
0.060
0.040
0.020
1
2
3
4
5
6
7
8
9
10
11
12
13
14
15
16
17
18
19
20
21
22
23
Average

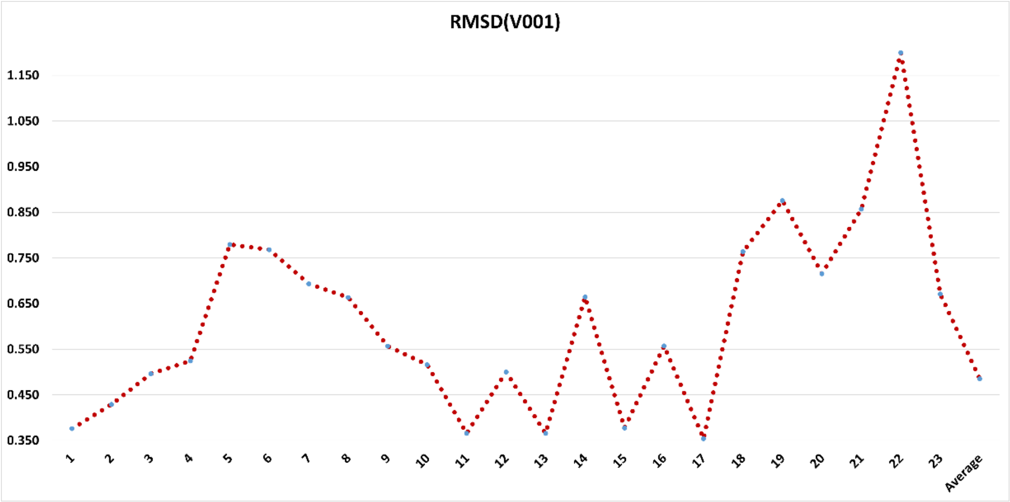

Figure S17. Graphical representation of the standard deviation (SD) per selected atom (from Table S15) and root mean square deviation relative to theory for all dataset (from Table S15) for the atomic charge (in e) and atomic volume (in Å^3) for XWR.

Crystal habit effect

Table S16. Statistical evaluation of C-H bond lengths (in Å) after HAR for the sub-sets related to the crystal habit effect. Selected datasets: natural shape crystals (datasets 1-12,18,20) and test crystals (datasets 13-17,19,21-23). The column SD refers to the sample standard deviation upon averaging the bond lengths for the same bond across all selected datasets. RMSD(Opt) refers to the root mean square deviation of the HAR results to the corresponding results from theoretical geometry optimization that are given in Table S7. RMSD(neutron) refers to the root mean square deviation of the HAR results to averaged tabulated results from neutron diffraction. The average neutron-diffraction values for C-H (methyl)= 1.083 Å and C-H (aromatic)=1.077 Å. C9-H9 to C6-H6 are the aromatic bonds; C10-H10A to C11-H11C are the methyl bonds.

Natural shape crystal	Average of selected datasets	SD of selected datasets
**C9-H9**	1.087	0.008
**C8-H8**	1.084	0.009
**C7-H7**	1.088	0.010
**C6-H6**	1.088	0.008
**C10-H10A**	1.077	0.013
**C10-H10B**	1.085	0.010
**C10-H10C**	1.076	0.014
**C11-H11A**	1.086	0.011
**C11-H11B**	1.081	0.014
**C11-H11C**	1.073	0.016
**RMSD (opt)**	0.007	**-**

**RMSD (neutron)**	0.005	-
**Average aromatic**	1.087	-
**Average methyl**	1.080	-
**SD aromatic**	0.002	-
**SD methyl**	0.005	-
**Test Crystal**	**Average of selected datasets**	**SD of selected datasets**
**C9-H9**	1.084	0.011
**C8-H8**	1.088	0.004
**C7-H7**	1.093	0.005
**C6-H6**	1.093	0.010
**C10-H10A**	1.056	0.015
**C10-H10B**	1.074	0.007
**C10-H10C**	1.066	0.013
**C11-H11A**	1.082	0.012
**C11-H11B**	1.069	0.021
**C11-H11C**	1.052	0.027
**RMSD (opt)**	0.019	-
**RMSD (neutron)**	0.012	-
**Average aromatic**	1.089	-
**Average methyl**	1.079	-
**SD aromatic**	0.004	-
**SD methyl**	0.011	-

Table S17. Statistical evaluation of the electron density at bond-critical points (in eÅ^{-3}) and its Laplacian (in eÅ^{-5}) after XWR for the sub-sets related to the crystal habit effect. Selected datasets: natural shape crystals (datasets 1-12,18,20) and test crystals (datasets 13-17,19,21-23). The column SD refers to the sample standard deviation upon averaging the values of density or Laplacian for the same bond across all selected datasets. RMSD(opt) refers to the root mean square deviation of the XWR results to the corresponding results from theoretical geometry optimization. Full list of values in Table S9.

**Natural shape crystal (Density)**	**Average of selected datasets**	**SD of selected datasets**
O(1)-C(2)	2.70	0.05
C(2)-C(3)	1.81	0.03
C(1)-S(1)	1.54	0.04
C(11)-S(1)	1.34	0.04
C(6)-H(6)	1.94	0.03
C(10)-H(10A)	1.96	0.08
C(7)-C(8)	2.16	0.02
**RMSD (opt)**	0.09	-

**Natural shape crystal (Laplacian)**	**Average of selected datasets**	**SD of selected datasets**
O(1)-C(2)	-4.2	4.1
C(2)-C(3)	-18.3	0.9
C(1)-S(1)	-12.2	1.3
C(11)-S(1)	-9.7	0.7
C(6)-H(6)	-26.1	1.0
C(10)-H(10A)	-25.0	1.1
C(7)-C(8)	-24.1	1.1
**RMSD (opt)**	1.1	-
**Test Crystal (Density)**	**Average of selected datasets**	**SD of selected datasets**
O(1)-C(2)	2.6	0.0
C(2)-C(3)	1.8	0.1
C(1)-S(1)	1.4	0.1
C(11)-S(1)	1.3	0.0
C(6)-H(6)	1.9	0.1
C(10)-H(10A)	1.9	0.1
C(7)-C(8)	2.1	0.1
**RMSD (opt)**	0.1	-
**Test Crystal (Laplacian)**	**Average of selected datasets**	**SD of selected datasets**
O(1)-C(2)	-1.8	4.1
C(2)-C(3)	-17.8	1.6
C(1)-S(1)	-10.5	2.7
C(11)-S(1)	-9.4	1.8
C(6)-H(6)	-25.7	0.9
C(10)-H(10A)	-24.1	1.8
C(7)-C(8)	-24.7	1.1
**RMSD (opt)**	1.5	-

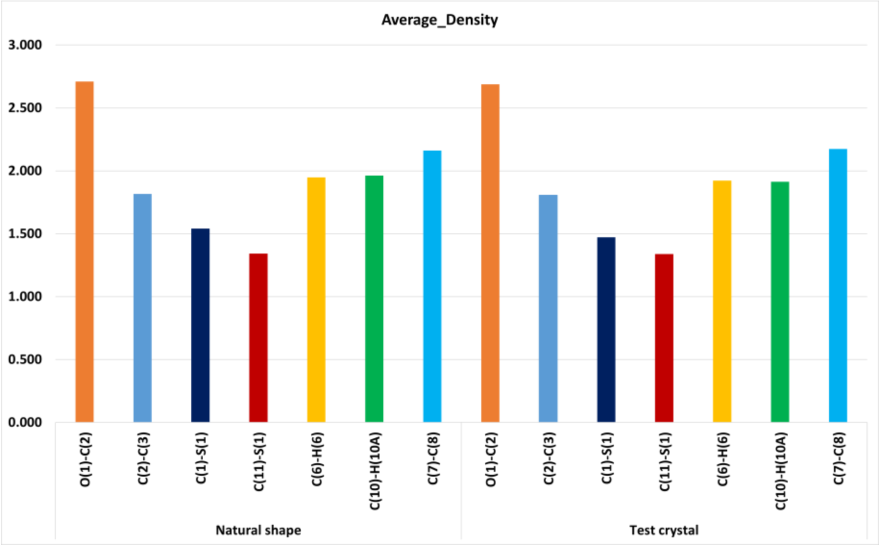
Average_Density
3.000
2.500
2.000
1.500
1.000
0.500
0.000
O(1)-C(2)
C(2)-C(3)
C(1)-S(1)
C(11)-S(1)
C(6)-H(6)
C(10)-H(10A)
C(7)-C(8)
Natural shape
Test crystal

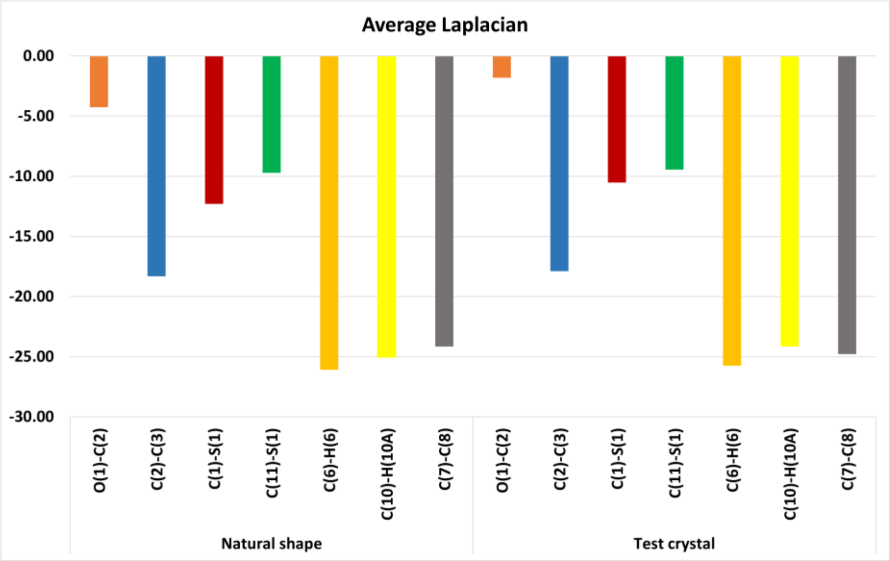
Average Laplacian
0.00
-5.00
-10.00
-15.00
-20.00
-25.00
-30.00
O(1)-C(2)
C(2)-C(3)
C(1)-S(1)
C(11)-S(1)
C(6)-H(6)
C(10)-H(10A)
C(7)-C(8)
Natural shape
Test crystal

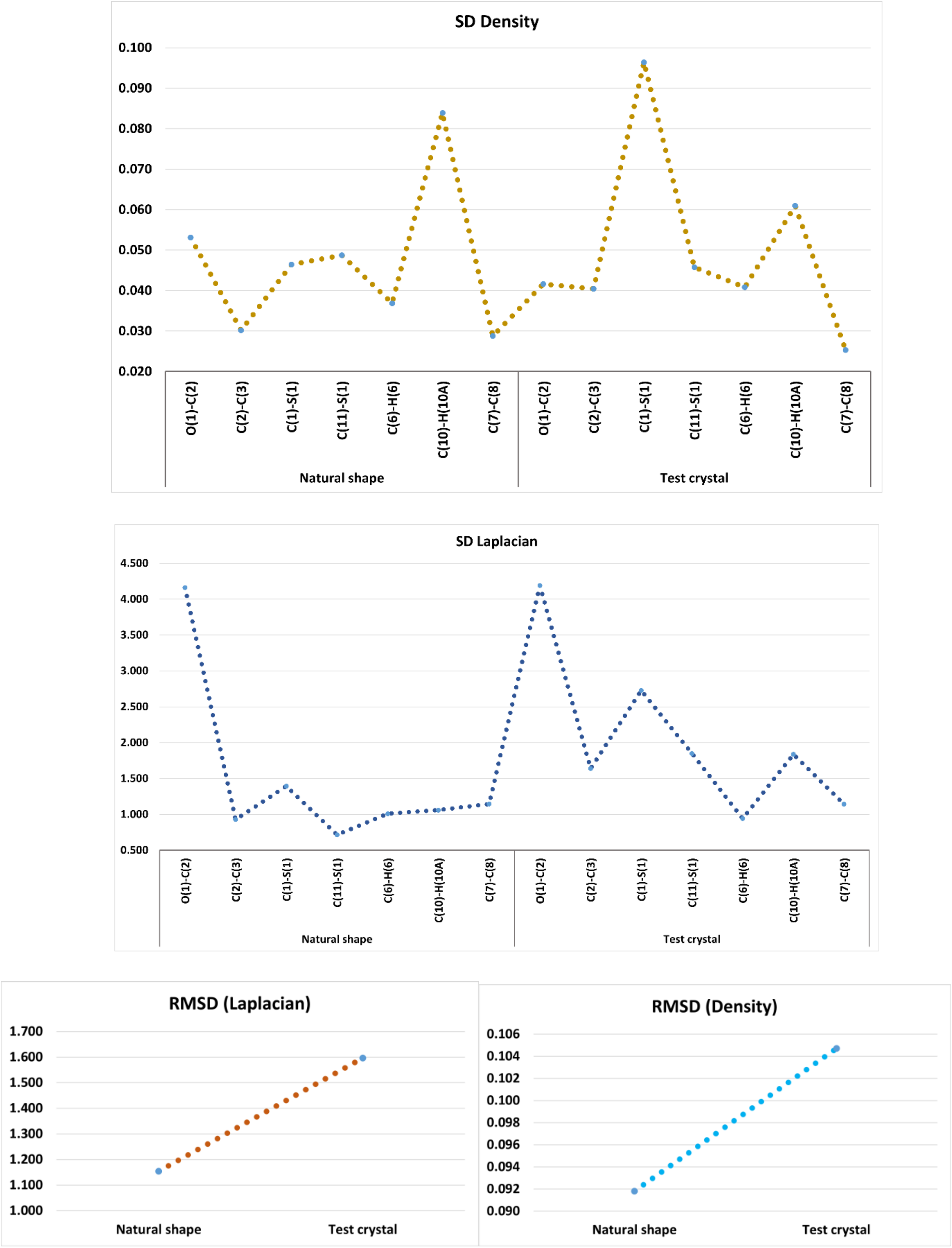

Figure S18. Plots of the statistical evaluation of topological properties of electron density for the sub-sets related to the crystal habit effect according to Table S17. Density = electron density at the bond critical point in eÅ^{-3}. Laplacian = Laplacian of the electron density at the bond critical point in eÅ^{-5}.

Table S18. Statistical evaluation of atomic properties Q001 (in e) and V001 (in $Å^3$) after XWR for the sub-sets related to the crystal habit effect. Selected datasets: natural shape crystals (datasets 1-12,18,20) and test crystals (datasets 13-17,19,21-23). The column SD refers to the sample standard deviation upon averaging the values of Q001 and V001 for the same atom across all selected datasets. RMSD(opt) refers to the root mean square deviation of the XWR results to the corresponding results from theoretical geometry optimization. Full list of values in Table S11.

**Natural shape crystal (Q001)**	**Average of selected datasets**	**SD of selected datasets**
S(1)	0.42	0.17
O(1)	-1.28	0.05
C(1)	-0.25	0.04
C(11)	-0.11	0.10
C(7)	-0.07	0.03
H(6)	0.08	0.02
H(10A)	0.11	0.02
RMSD (opt)	0.05	-
**Natural shape crystal (V001)**	**Average of selected datasets**	**SD of selected datasets**
S(1)	18.7	0.72
O(1)	20.45	0.75
C(1)	12.06	0.18
C(11)	10.97	0.38
C(7)	12.70	0.28
H(6)	6.93	0.43
H(10A)	6.13	0.39
RMSD (opt)	0.05	-
**Test Crystal (Q001)**	**Average of selected datasets**	**SD of selected datasets**
S(1)	0.49	0.15
O(1)	-1.27	0.04
C(1)	-0.21	0.07
C(11)	-0.16	0.05
C(7)	-0.10	0.04
H(6)	0.10	0.03
H(10A)	0.12	0.03
RMSD (opt)	0.07	-
**Test Crystal (V001)**	**Average of selected datasets**	**SD of selected datasets**
S(1)	18.40	0.56
O(1)	20.47	0.45
C(1)	11.97	0.33
C(11)	11.20	0.32

C(7)	12.77	0.25
H(6)	6.75	0.24
H(10A)	6.34	0.46
RMSD (opt)	0.05	-

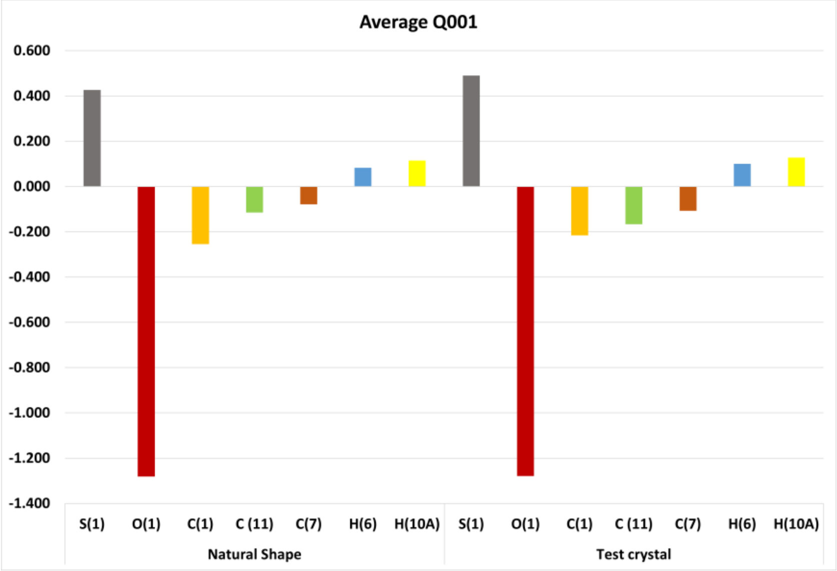

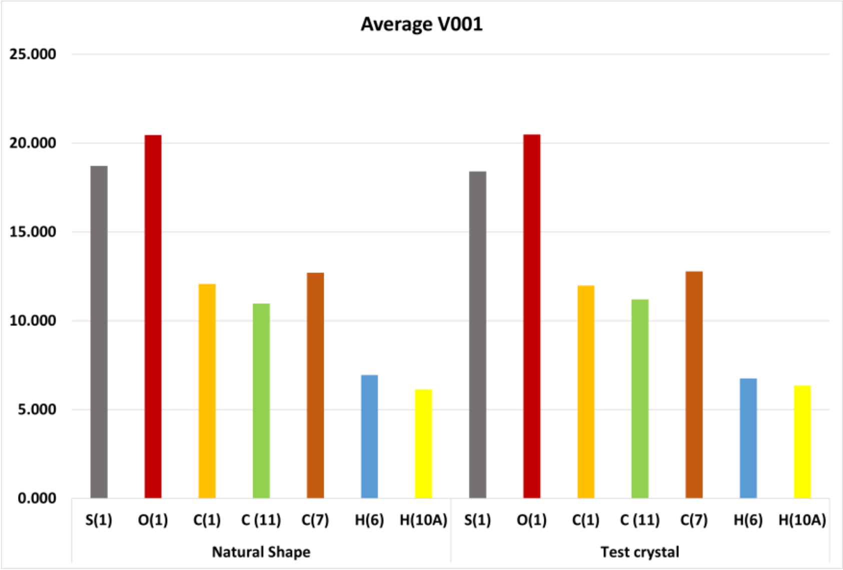

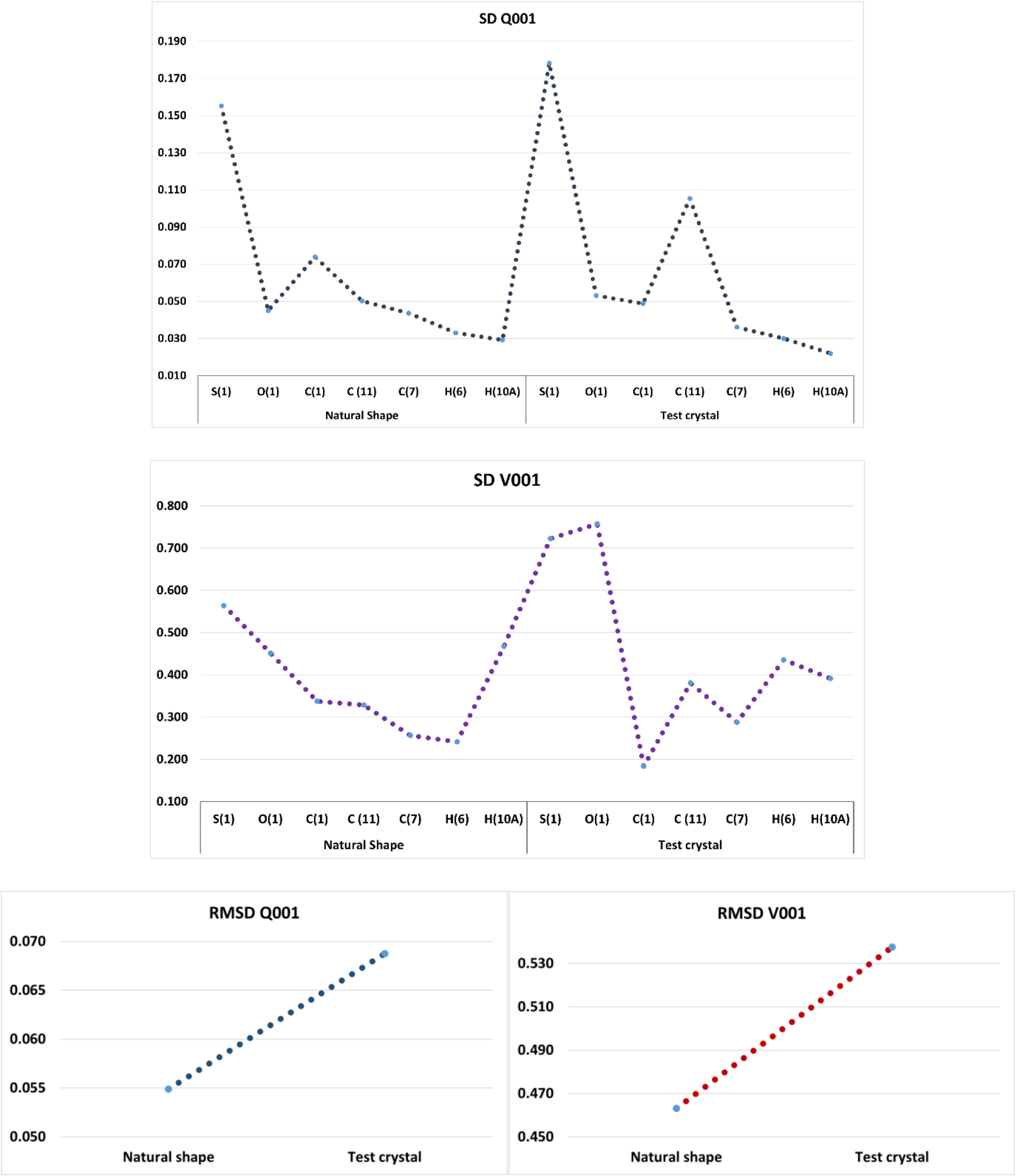

Figure S19. Plots of the statistical evaluation of atomic properties for the sub-sets related to the crystal habit effect according to Table S18. Q001 in e, V001 in Å^3.

Wavelength effect

Table S19. Statistical evaluation of C-H bond lengths (in Å) after HAR for the sub-sets related to the wavelength effect. Selected datasets: Synchrotron (datasets 6,7,8); Ag radiation (datasets 1,9,11,13,17); Mo radiation (datasets 3,4,5,12,14,16); Cu radiation (datasets 2.,10,15,18-23). The column SD refers to the sample standard deviation upon averaging the bond lengths for the same bond across all selected datasets. RMSD(opt) refers to the root mean square deviation of the HAR results to the corresponding results from theoretical geometry optimization that are given in Table S7. RMSD(neutron) refers to the root mean square deviation of the HAR results to averaged tabulated results from neutron diffraction. The average neutron-diffraction values for C-H (methyl) = 1.083 Å and C-H (aromatic) =1.077 Å.

Synchrotron	Average of selected datasets	SD of selected datasets
C9-H9	1.086	0.006
C8-H8	1.077	0.005
C7-H7	1.092	0.003
C6-H6	1.087	0.009
C10-H10A	1.090	0.004
C10-H10B	1.075	0.008
C10-H10C	1.081	0.015
C11-H11A	1.077	0.018
C11-H11B	1.093	0.005
C11-H11C	1.072	0.001
RMSD (opt)	0.008	-
RMSD (neutron)	0.008	-
Average aromatic	1.085	-
Average methyl	1.081	-
SD aromatic	0.006	-
SD methyl	0.008	-
**Ag radiation**	**Average of selected datasets**	**SD of selected datasets**
C9-H9	1.086	0.002
C8-H8	1.083	0.005
C7-H7	1.088	0.005
C6-H6	1.089	0.003
C10-H10A	1.066	0.010
C10-H10B	1.083	0.006
C10-H10C	1.070	0.010
C11-H11A	1.084	0.007
C11-H11B	1.074	0.017
C11-H11C	1.077	0.005
RMSD (opt)	0.011	-
RMSD (neutron)	0.005	-

**Average aromatic**	1.087	-
**Average methyl**	1.076	-
**SD aromatic**	0.002	-
**SD methyl**	0.007	-
**Mo radiation**	**Average of selected datasets**	**SD of selected datasets**
**C9-H9**	1.086	0.006
**C8-H8**	1.089	0.009
**C7-H7**	1.082	0.007
**C6-H6**	1.091	0.010
**C10-H10A**	1.067	0.023
**C10-H10B**	1.086	0.013
**C10-H10C**	1.068	0.011
**C11-H11A**	1.080	0.006
**C11-H11B**	1.061	0.015
**C11-H11C**	1.077	0.007
**RMSD (opt)**	0.013	-
**RMSD (neutron)**	0.007	-
**Average aromatic**	1.087	-
**Average methyl**	1.073	-
**SD aromatic**	0.003	-
**SD methyl**	0.009	-
**Cu radiation**	**Average of selected datasets**	**SD of selected datasets**
**C9-H9**	1.086	0.014
**C8-H8**	1.088	0.005
**C7-H7**	1.097	0.008
**C6-H6**	1.091	0.012
**C10-H10A**	1.064	0.016
**C10-H10B**	1.079	0.011
**C10-H10C**	1.074	0.018
**C11-H11A**	1.090	0.012
**C11-H11B**	1.083	0.014
**C11-H11C**	1.048	0.030
**RMSD (opt)**	0.016	-
**RMSD (neutron)**	0.012	-
**Average aromatic**	1.090	-
**Average methyl**	1.073	-
**SD aromatic**	0.004	-
**SD methyl**	0.015	-

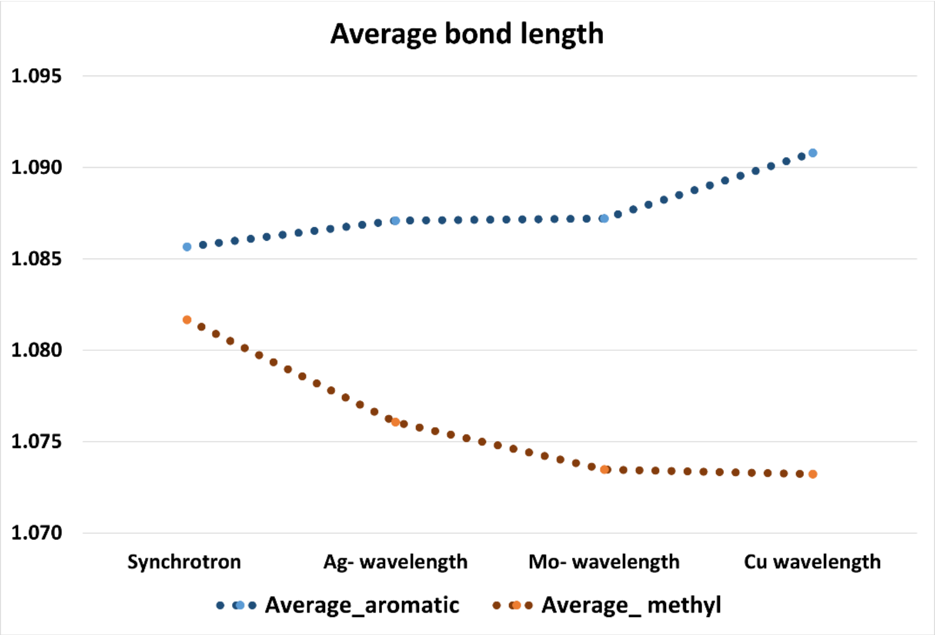
Average bond length
1.095
1.090
1.085
1.080
1.075
1.070
Synchrotron
Ag- wavelength
Mo- wavelength
Cu wavelength
Average_aromatic
Average_ methyl

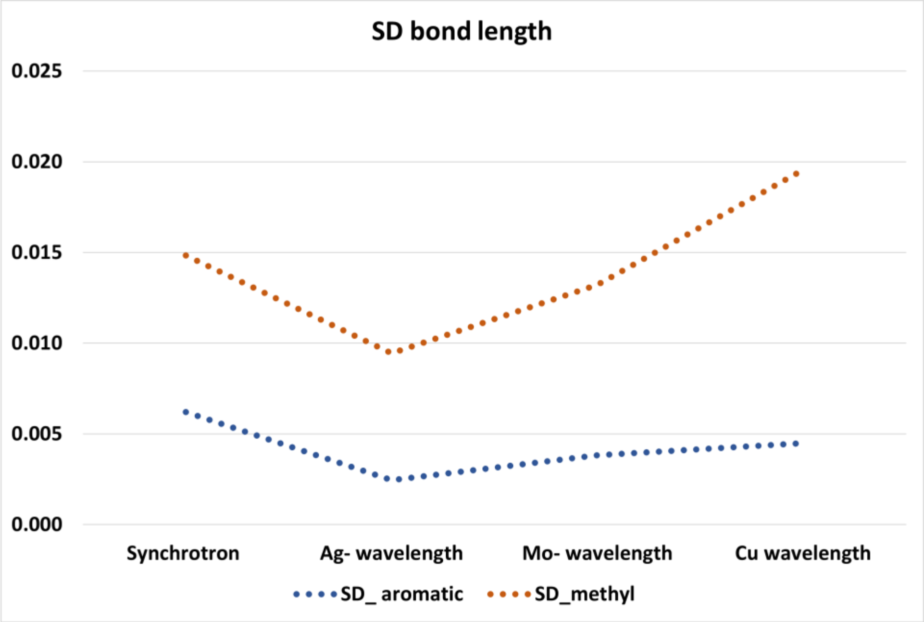
SD bond length
0.025
0.020
0.015
0.010
0.005
0.000
Synchrotron
Ag- wavelength
Mo- wavelength
Cu wavelength
SD_ aromatic
SD_methyl

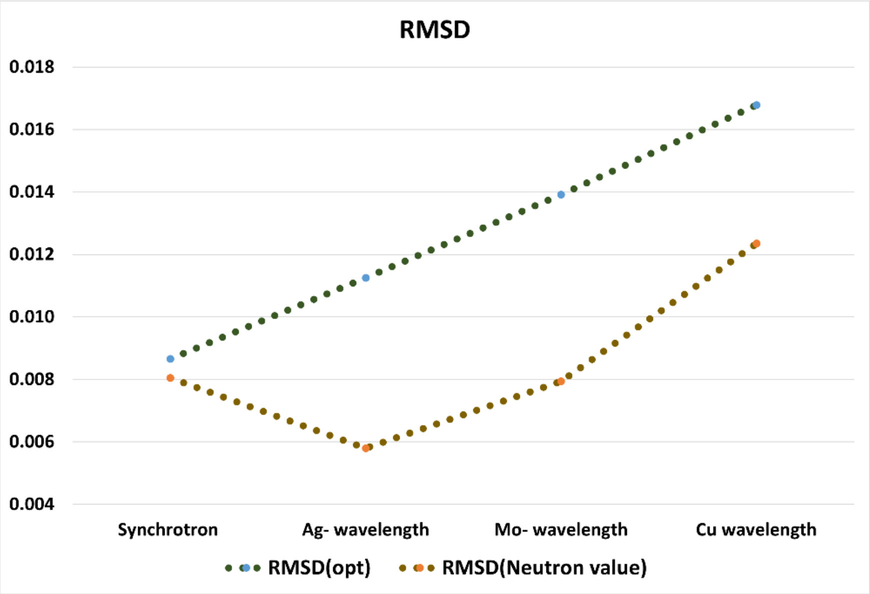

Figure S20. Plots of the statistical evaluation of C-H bond lengths (in Å) for the sub-sets related to the wavelength effect according to Table S19.

Table S20. Statistical evaluation of the electron density at bond-critical points (in eÅ^{-3}) and its Laplacian (in eÅ^{-5}) after XWR for the sub-sets related to the wavelength effect. Selected datasets: Synchrotron (datasets 6,7,8); Ag radiation (datasets 1,9,11,13,17); Mo radiation (datasets 3,4,5,12,14,16); Cu radiation (datasets 2,10,15,18-23). The column SD refers to the sample standard deviation upon averaging the values of density or Laplacian for the same bond across all selected datasets. RMSD(opt) refers to the root mean square deviation of the XWR results to the corresponding results from theoretical geometry optimization. Full list of values in Table S9.

**Synchrotron (Density)**	**Average of selected datasets**	**SD of selected datasets**
O(1)-C(2)	2.76	0.02
C(2)-C(3)	1.82	0.01
C(1)-S(1)	1.54	0.01
C(11)-S(1)	1.35	0.02
C(6)-H(6)	1.97	0.04
C(10)-H(10A)	1.96	0.05
C(7)-C(8)	2.16	0.03
**RMSD (opt)**	0.07	-
**Synchrotron (Laplacian)**	**Average of selected datasets**	**SD of selected datasets**
O(1)-C(2)	-7.7	3.2
C(2)-C(3)	-18.3	0.3
C(1)-S(1)	-12.6	0.9

C(11)-S(1)	-10.0	0.6
C(6)-H(6)	-26.7	0.9
C(10)-H(10A)	-26.0	1.3
C(7)-C(8)	-24.5	1.1
**RMSD (opt)**	1.9	-
**Ag radiation (Density)**	**Average of selected datasets**	**SD of selected datasets**
O(1)-C(2)	2.67	0.01
C(2)-C(3)	1.84	0.01
C(1)-S(1)	1.55	0.01
C(11)-S(1)	1.37	0.02
C(6)-H(6)	1.94	0.02
C(10)-H(10A)	1.96	0.08
C(7)-C(8)	2.17	0.02
**RMSD (opt)**	0.10	-
**Ag radiation (Laplacian)**	**Average of selected datasets**	**SD of selected datasets**
O(1)-C(2)	0.8	4.1
C(2)-C(3)	-19.2	0.4
C(1)-S(1)	-12.5	0.4
C(11)-S(1)	-10.5	0.9
C(6)-H(6)	-26.0	0.4
C(10)-H(10A)	-24.9	0.5
C(7)-C(8)	-24.6	1.1
**RMSD (opt)**	2.6	-
**Mo radiation (Density)**	**Average of selected datasets**	**SD of selected datasets**
O(1)-C(2)	2.72	0.01
C(2)-C(3)	1.83	0.01
C(1)-S(1)	1.55	0.03
C(11)-S(1)	1.35	0.02
C(6)-H(6)	1.93	0.03
C(10)-H(10A)	1.96	0.10
C(7)-C(8)	2.17	0.03
**RMSD (opt)**	0.09	-
**Mo radiation (Laplacian)**	**Average of selected datasets**	**SD of selected datasets**
O(1)-C(2)	-2.8	2.9
C(2)-C(3)	-18.9	0.9
C(1)-S(1)	-12.5	1.0
C(11)-S(1)	-9.7	1.0
C(6)-H(6)	-25.9	1.1
C(10)-H(10A)	-24.4	0.8

C(7)-C(8)	-24.6	1.5
**RMSD (opt)**	1.5	**-**
**Cu radiation (Density)**	**Average of selected datasets**	**SD of selected datasets**
O(1)-C(2)	2.67	0.05
C(2)-C(3)	1.77	0.02
C(1)-S(1)	1.45	0.08
C(11)-S(1)	1.30	0.05
C(6)-H(6)	1.92	0.04
C(10)-H(10A)	1.90	0.05
C(7)-C(8)	2.15	0.02
**RMSD (opt)**	0.10	**-**
**Cu radiation (Laplacian)**	**Average of selected datasets**	**SD of selected datasets**
O(1)-C(2)	-4.3	3.6
C(2)-C(3)	-16.9	0.7
C(1)-S(1)	-10.0	2.7
C(11)-S(1)	-8.8	1.3
C(6)-H(6)	-25.6	1.1
C(10)-H(10A)	-24.2	1.9
C(7)-C(8)	-24.0	1.1
**RMSD (opt)**	0.9	**-**

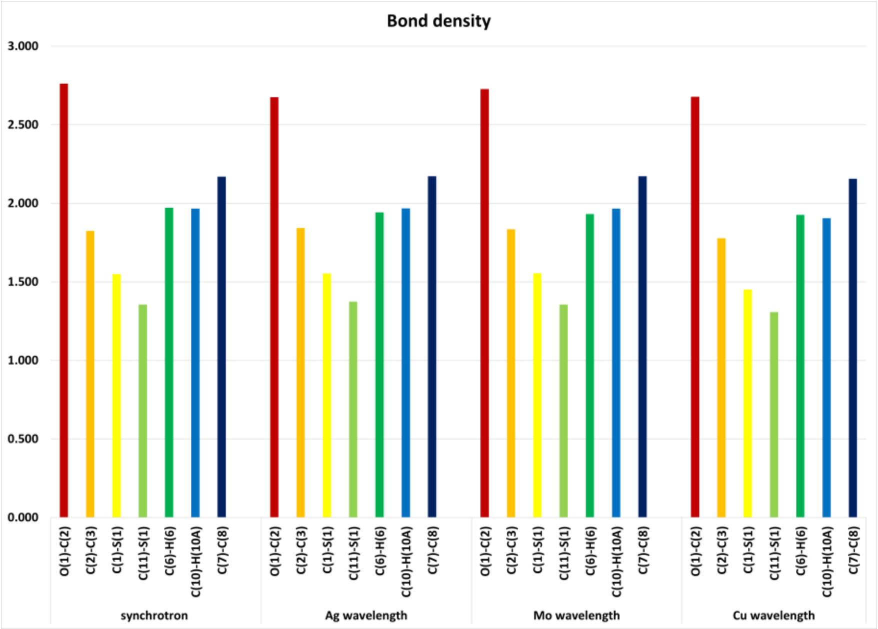

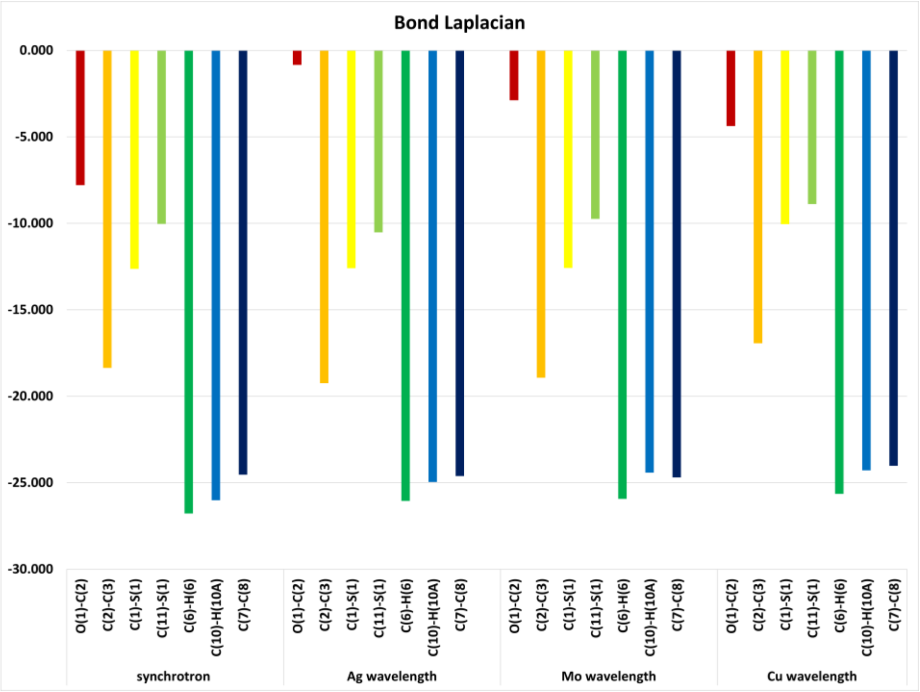
Bond Laplacian
0.000
-5.000
-10.000
-15.000
-20.000
-25.000
-30.000
O(1)-C(2)
C(2)-C(3)
C(1)-S(1)
C(11)-S(1)
C(6)-H(6)
C(10)-H(10A)
C(7)-C(8)
synchrotron
Ag wavelength
Mo wavelength
Cu wavelength

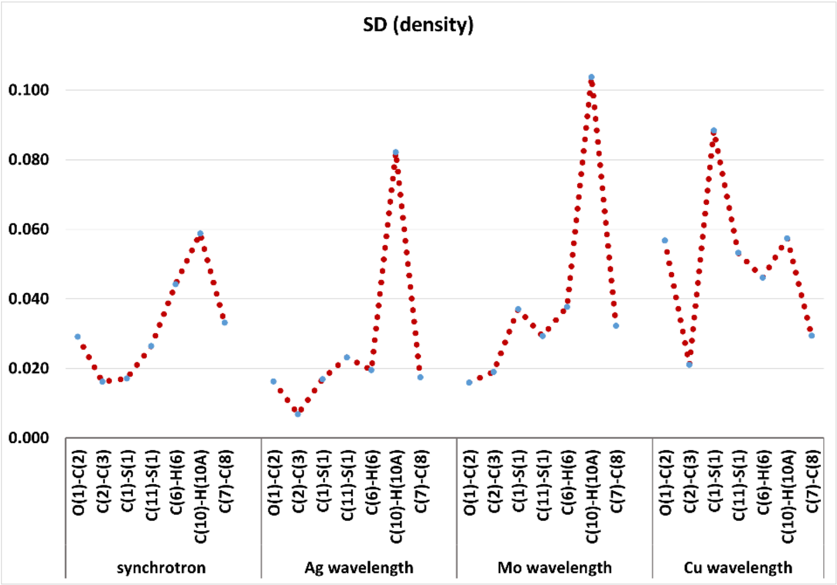
SD (density)
0.100
0.080
0.060
0.040
0.020
0.000
O(1)-C(2)
C(2)-C(3)
C(1)-S(1)
C(11)-S(1)
C(6)-H(6)
C(10)-H(10A)
C(7)-C(8)
synchrotron
Ag wavelength
Mo wavelength
Cu wavelength

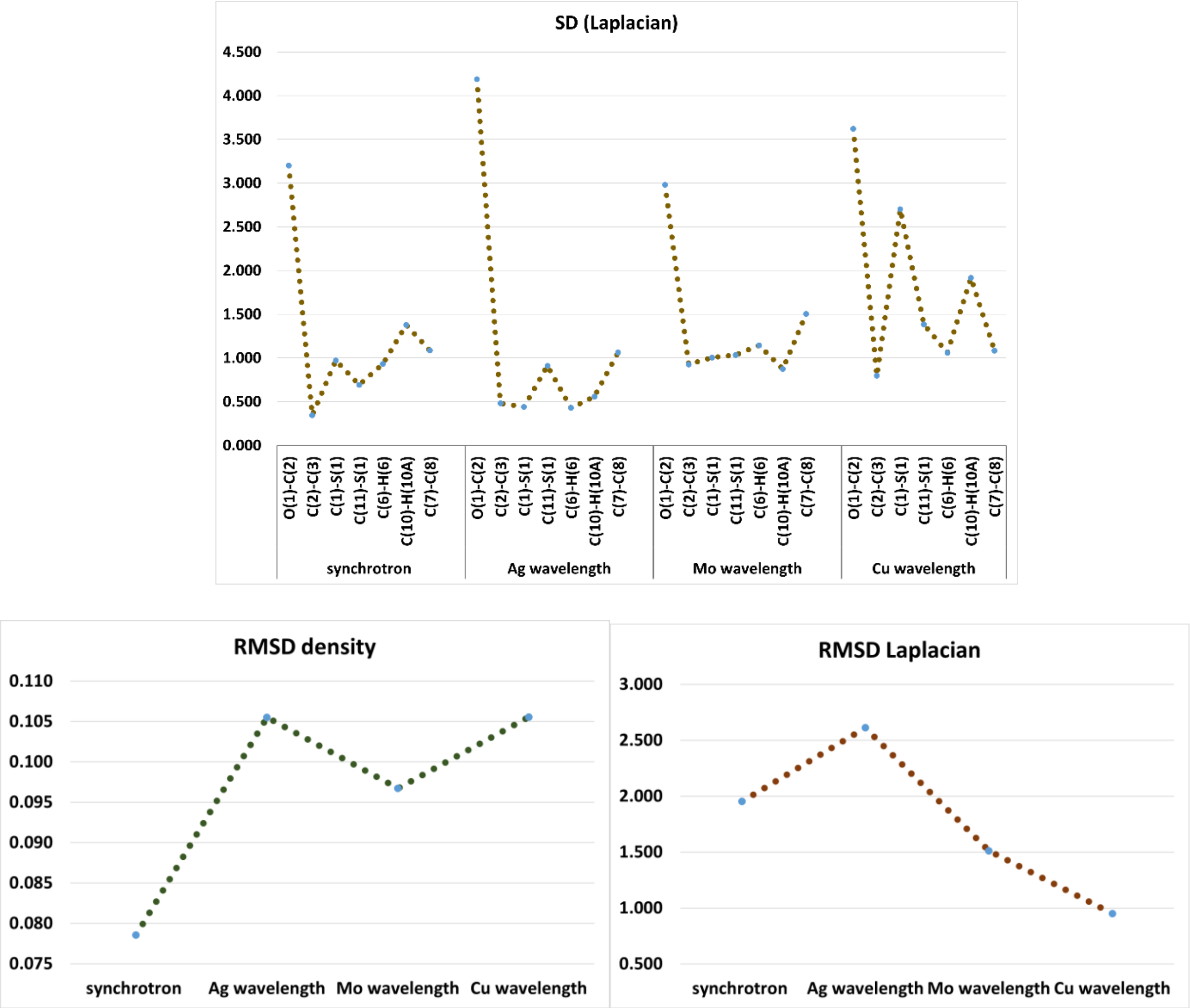

Figure S21. Plots of the statistical evaluation of topological properties of electron density for the sub-sets related to the wavelength effect according to Table S20. Density = electron density at the bond critical point in eÅ^{-3}. Laplacian = Laplacian of the electron density at the bond critical point in eÅ^{-5}.

Table S21. Statistical evaluation of atomic properties Q001 (in e) and V001 (in Å^3) after XWR for the sub-sets related to the wavelength effect. Selected datasets: Synchrotron (datasets 6,7,8); Ag radiation (datasets 1,9,11,13,17); Mo radiation (datasets 3,4,5,12,14,16); Cu radiation (datasets 2,10,15,18-23). The column SD refers to the sample standard deviation upon averaging the values of Q001 and V001 for the same atom across all selected datasets. RMSD(opt) refers to the root mean square deviation of the XWR results to the corresponding results from geometry optimization. Full list of values in Table S11.

Synchrotron (Q001)	Average of selected datasets	SD of selected datasets
S(1)	0.63	0.18
O(1)	-1.28	0.02
C(1)	-0.33	0.10

C(11)	-0.05	0.04
C(7)	-0.08	0.04
H(6)	0.04	0.02
H(10A)	0.10	0.05
RMSD (opt)	0.08	-
**Synchrotron (V001)**	**Average of selected datasets**	**SD of selected datasets**
S(1)	18.87	0.10
O(1)	20.25	0.16
C(1)	12.03	0.25
C(11)	10.97	0.05
C(7)	12.74	0.17
H(6)	6.84	0.12
H(10A)	6.19	0.63
RMSD (opt)	0.67	-
**Ag radiation (Q001)**	**Average of selected datasets**	**SD of selected datasets**
S(1)	0.39	0.06
O(1)	-1.29	0.04
C(1)	-0.24	0.03
C(11)	-0.12	0.02
C(7)	-0.09	0.02
H(6)	0.10	0.01
H(10A)	0.11	0.01
RMSD (opt)	0.06	-
**Ag radiation (V001)**	**Average of selected datasets**	**SD of selected datasets**
S(1)	18.87	0.14
O(1)	20.25	0.20
C(1)	12.03	0.08
C(11)	10.97	0.13
C(7)	12.74	0.09
H(6)	6.84	0.05
H(10A)	6.19	0.23
RMSD (opt)	0.38	-
**Mo radiation (Q001)**	**Average of selected datasets**	**SD of selected datasets**
S(1)	0.40	0.10
O(1)	-1.30	0.04
C(1)	-0.23	0.05
C(11)	-0.08	0.03
C(7)	-0.08	0.07
H(6)	0.09	0.03

H(10A)	0.11	0.02
RMSD (opt)	0.07	-
**Mo radiation (V001)**	**Average of selected datasets**	**SD of selected datasets**
S(1)	18.77	0.56
O(1)	20.18	0.59
C(1)	12.09	0.41
C(11)	10.94	0.15
C(7)	12.64	0.37
H(6)	6.72	0.57
H(10A)	6.08	0.51
RMSD (opt)	0.40	-
**Cu radiation (Q001)**	**Average of selected datasets**	**SD of selected datasets**
S(1)	0.45	0.19
O(1)	-1.25	0.04
C(1)	-0.20	0.04
C(11)	-0.19	0.08
C(7)	-0.09	0.02
H(6)	0.08	0.03
H(10A)	0.13	0.02
RMSD (opt)	0.06	-
**Cu radiation (V001)**	**Average of selected datasets**	**SD of selected datasets**
S(1)	18.51	0.82
O(1)	20.68	0.69
C(1)	12.04	0.29
C(11)	11.37	0.29
C(7)	12.86	0.21
H(6)	6.91	0.25
H(10A)	6.27	0.46
RMSD (opt)	0.59	-

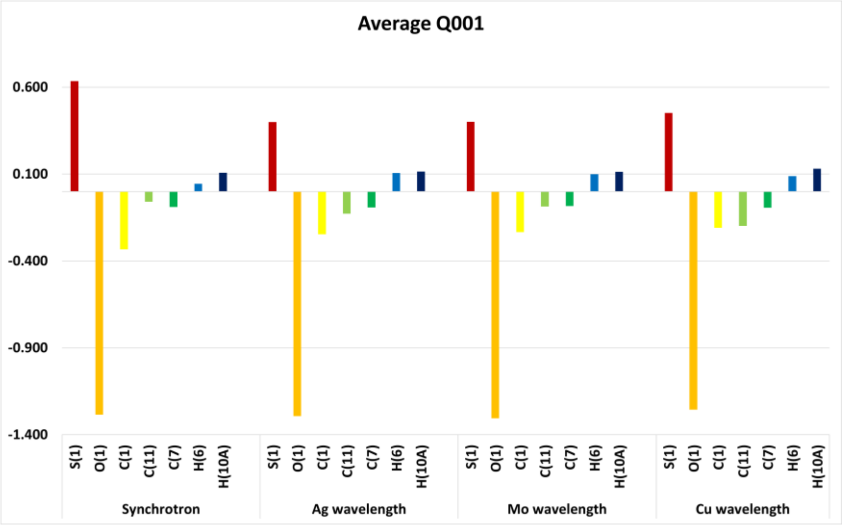
Average Q001
0.600
0.100
-0.400
-0.900
-1.400
S(1)
O(1)
C(1)
C(11)
C(7)
H(6)
H(10A)
Synchrotron
Ag wavelength
Mo wavelength
Cu wavelength

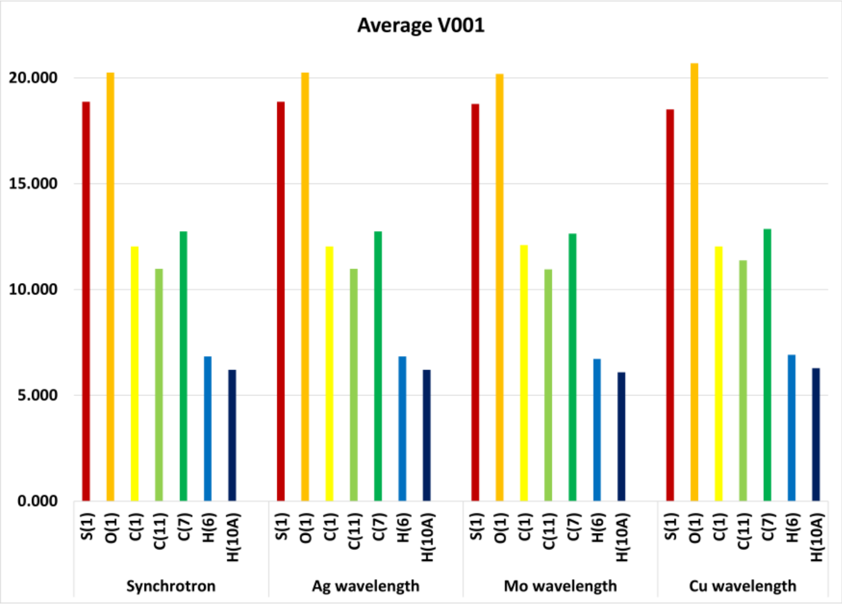
Average V001
20.000
15.000
10.000
5.000
0.000
S(1)
O(1)
C(1)
C(11)
C(7)
H(6)
H(10A)
Synchrotron
Ag wavelength
Mo wavelength
Cu wavelength

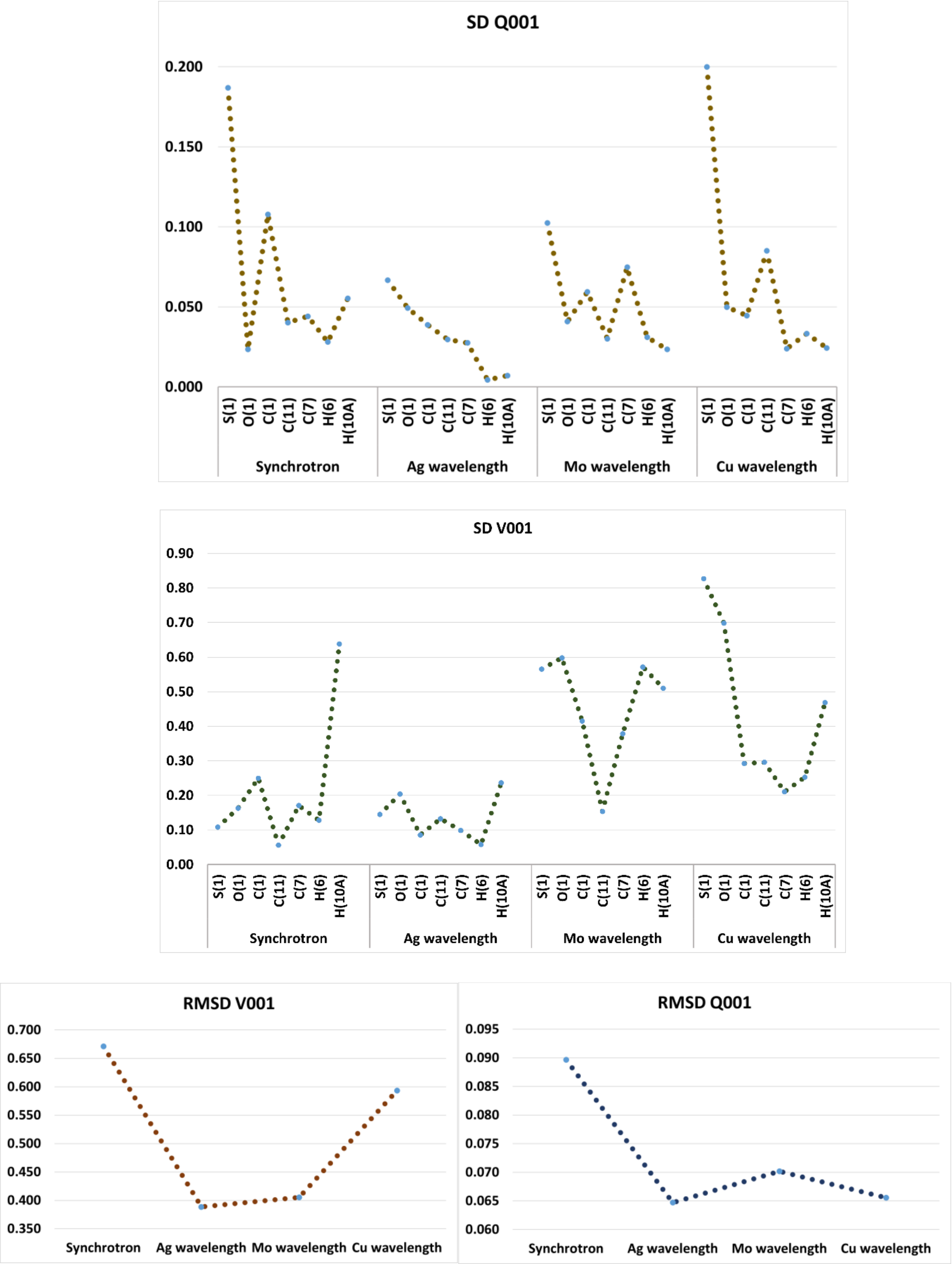

Figure S22. Plots of the statistical evaluation of atomic properties for the sub-sets related to the wavelength effect according to Table S21. Q001 in e, V001 in Å^3.

Temperature effect

Table S22. Statistical evaluation of C-H bond lengths (in Å) after HAR for the sub-sets related to the temperature effect. Selected datasets: 100K (datasets 1-8); 150K (datasets 9,10); 292K (datasets 11-23). The column SD refers to the sample standard deviation upon averaging the bond lengths for the same bond across all selected datasets. RMSD(opt) refers to the root mean square deviation of the HAR results to the corresponding results from theoretical geometry optimization that are given in Table S7. RMSD(neutron) refers to the root mean square deviation of the HAR results to averaged tabulated results from neutron diffraction. The average neutron-diffraction values for C-H (methyl) = 1.083 Å and C-H (aromatic) =1.077 Å.

**100K**	**Average of selected datasets**	**SD of selected datasets**
**C9-H9**	1.084	0.006
**C8-H8**	1.082	0.006
**C7-H7**	1.089	0.010
**C6-H6**	1.088	0.006
**C10-H10A**	1.085	0.007
**C10-H10B**	1.084	0.009
**C10-H10C**	1.082	0.011
**C11-H11A**	1.083	0.011
**C11-H11B**	1.086	0.011
**C11-H11C**	1.077	0.006
**RMSD (opt)**	0.005	**-**
**RMSD (neutron)**	0.006	**-**
**Average aromatic**	1.086	**-**
**Average methyl**	1.083	**-**
**SD aromatic**	0.003	**-**
**SD methyl**	0.003	**-**
**150K**	**Average of selected datasets**	**SD of selected datasets**
**C9-H9**	1.086	0.001
**C8-H8**	1.082	0.004
**C7-H7**	1.096	0.013
**C6-H6**	1.092	0.006
**C10-H10A**	1.073	0.002
**C10-H10B**	1.089	0.003
**C10-H10C**	1.083	0.009
**C11-H11A**	1.093	0.004
**C11-H11B**	1.086	0.001
**C11-H11C**	1.083	0.004
**RMSD (opt)**	0.007	**-**
**RMSD (neutron)**	0.009	**-**

**Average aromatic**	1.089	**-**
**Average methyl**	1.084	**-**
**SD aromatic**	0.006	**-**
**SD methyl**	0.006	**-**
**292K (room temperature)**	**Average of selected datasets**	**SD of selected datasets**
**C9-H9**	1.087	0.011
**C8-H8**	1.089	0.007
**C7-H7**	1.090	0.008
**C6-H6**	1.091	0.011
**C10-H10A**	1.058	0.015
**C10-H10B**	1.078	0.011
**C10-H10C**	1.065	0.012
**C11-H11A**	1.084	0.012
**C11-H11B**	1.069	0.019
**C11-H11C**	1.055	0.027
**RMSD (opt)**	0.017	**-**
**RMSD (neutron)**	0.011	**-**
**Average aromatic**	1.089	**-**
**Average methyl**	1.068	**-**
**SD aromatic**	0.001	**-**
**SD methyl**	0.011	**-**

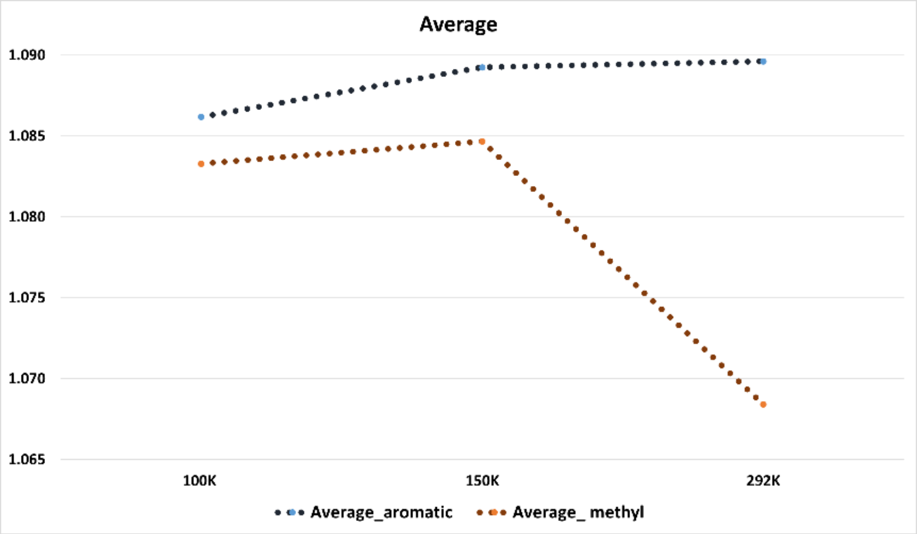

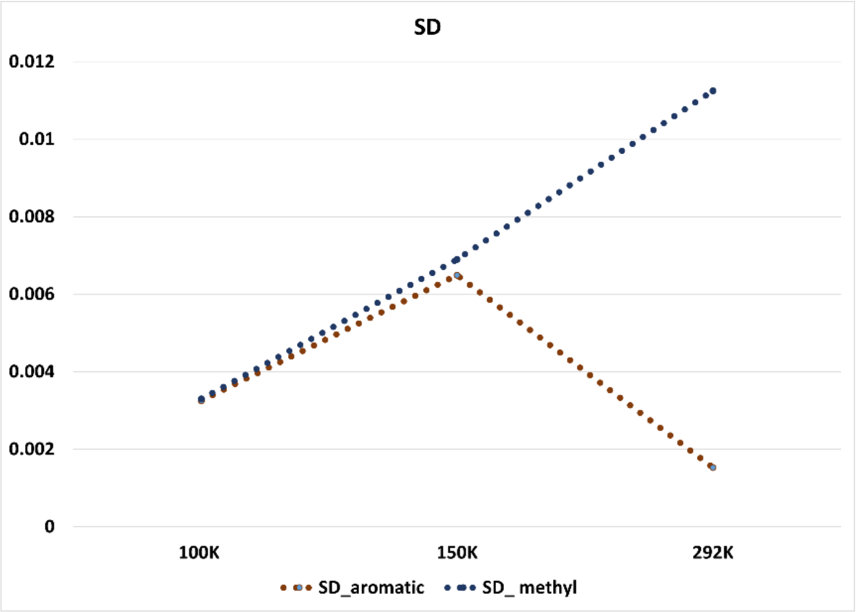

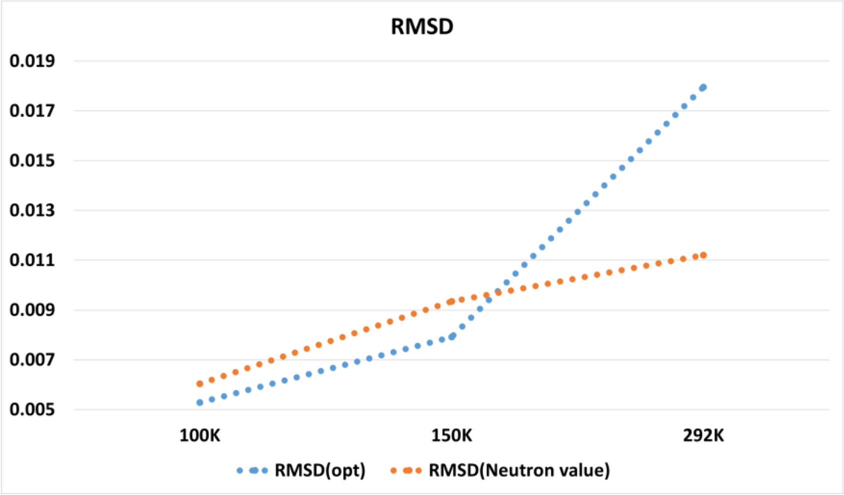

Figure S23. Plots of the statistical evaluation of C-H bond lengths (in Å) for the sub-sets related to the temperature effect according to Table S22.

Table S23. Statistical evaluation of the electron density at bond-critical points (in eÅ^{-3}) and its Laplacian (in eÅ^{-5}) after XWR for the sub-sets related to the temperature effect. Selected datasets: 100K (datasets 1-8); 150K (datasets 9,10); 292K (datasets 11-23). The column SD refers to the sample standard deviation upon averaging the values of density or Laplacian for the same bond across all selected datasets. RMSD(opt) refers to the root mean square deviation of the XWR results to the corresponding results from theoretical geometry optimization. Full list of values in Table S9.

**100K (Density)**	**Average of selected datasets**	**SD of selected datasets**
O(1)-C(2)	2.73	0.031
C(2)-C(3)	1.81	0.025
C(1)-S(1)	1.55	0.029
C(11)-S(1)	1.35	0.02
C(6)-H(6)	1.95	0.03
C(10)-H(10A)	1.93	0.03
C(7)-C(8)	2.16	0.03
RMSD (opt)	0.08	-
**100K (Laplacian)**	**Average of selected datasets**	**SD of selected datasets**
O(1)-C(2)	-6.3	3.0
C(2)-C(3)	-18.1	0.6
C(1)-S(1)	-12.7	0.8
C(11)-S(1)	-9.7	0.5
C(6)-H(6)	-26.1	0.7
C(10)-H(10A)	-25.3	0.9
C(7)-C(8)	-24.2	1.5
RMSD (opt)	1.3	-
**150K (Density)**	**Average of selected datasets**	**SD of selected datasets**
O(1)-C(2)	2.71	0.03
C(2)-C(3)	1.83	0.01
C(1)-S(1)	1.54	0.01
C(11)-S(1)	1.36	0.01
C(6)-H(6)	1.93	0.02
C(10)-H(10A)	1.92	0.01
C(7)-C(8)	2.16	0.00
RMSD (opt)	0.09	-
**150K (Laplacian)**	**Average of selected datasets**	**SD of selected datasets**
O(1)-C(2)	-3.1	7.5
C(2)-C(3)	-18.7	0.5
C(1)-S(1)	-12.6	0.3
C(11)-S(1)	-9.8	0.3

C(6)-H(6)	-25.6	0.6
C(10)-H(10A)	-25.2	0.1
C(7)-C(8)	-24.1	0.1
RMSD (opt)	1.3	-
**292K (Density)**	**Average of selected datasets**	**SD of selected datasets**
O(1)-C(2)	2.67	0.04
C(2)-C(3)	1.80	0.04
C(1)-S(1)	1.48	0.08
C(11)-S(1)	1.33	0.05
C(6)-H(6)	1.93	0.04
C(10)-H(10A)	1.94	0.10
C(7)-C(8)	2.16	0.02
RMSD (opt)	0.10	-
**292K (Laplacian)**	**Average of selected datasets**	**SD of selected datasets**
O(1)-C(2)	-1.4	3.5
C(2)-C(3)	-18.0	1.5
C(1)-S(1)	-10.7	2.4
C(11)-S(1)	-9.5	1.6
C(6)-H(6)	-25.9	1.1
C(10)-H(10A)	-24.2	1.6
C(7)-C(8)	-24.5	1.1
RMSD (opt)	1.6	-

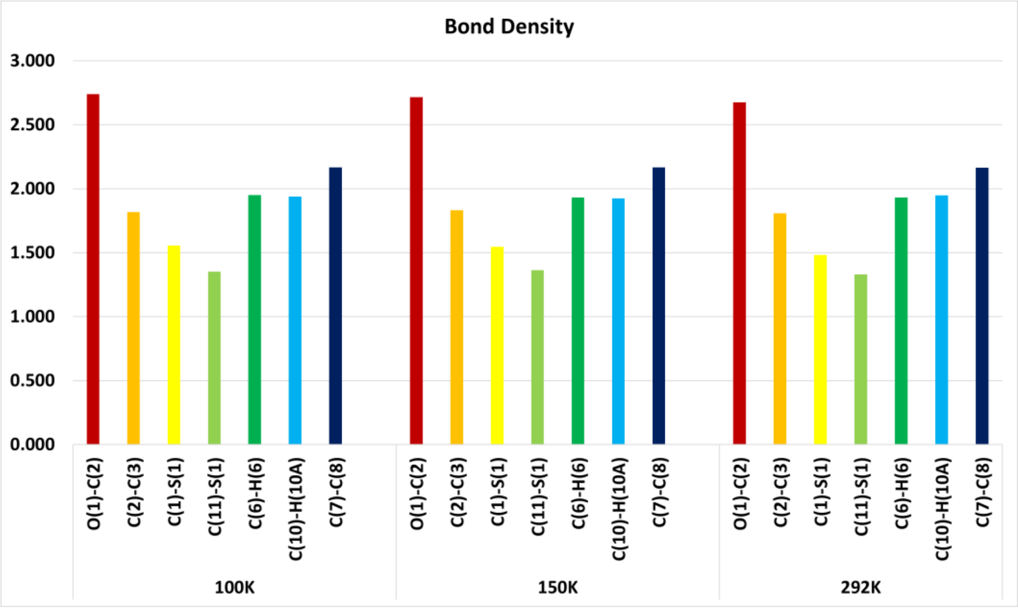

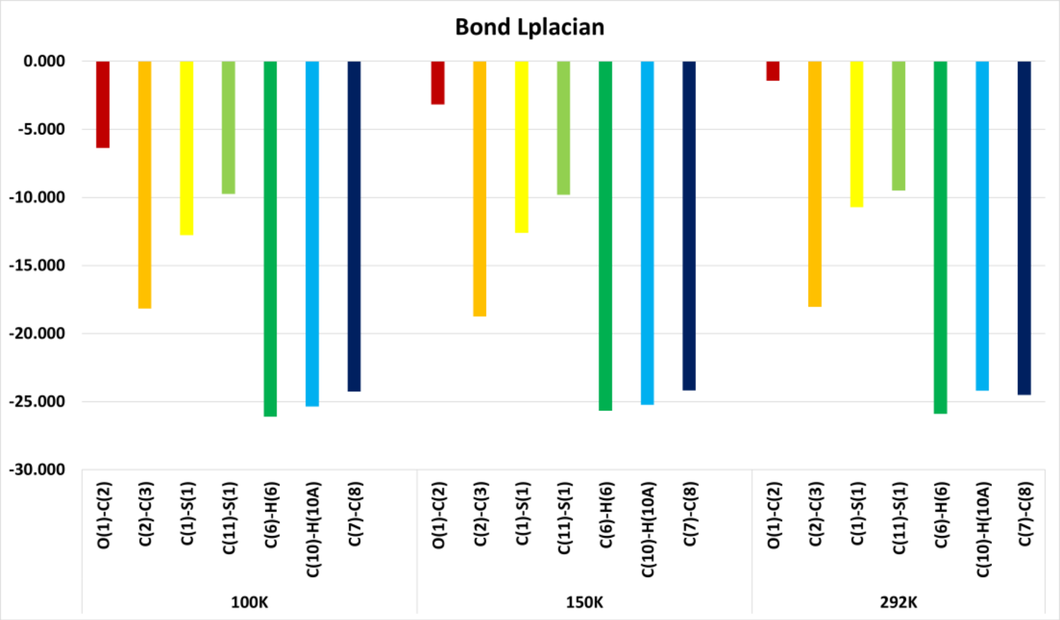
Bond Lplacian
0.000
-5.000
-10.000
-15.000
-20.000
-25.000
-30.000
O(1)-C(2)
C(2)-C(3)
C(1)-S(1)
C(11)-S(1)
C(6)-H(6)
C(10)-H(10A)
C(7)-C(8)
100K
150K
292K

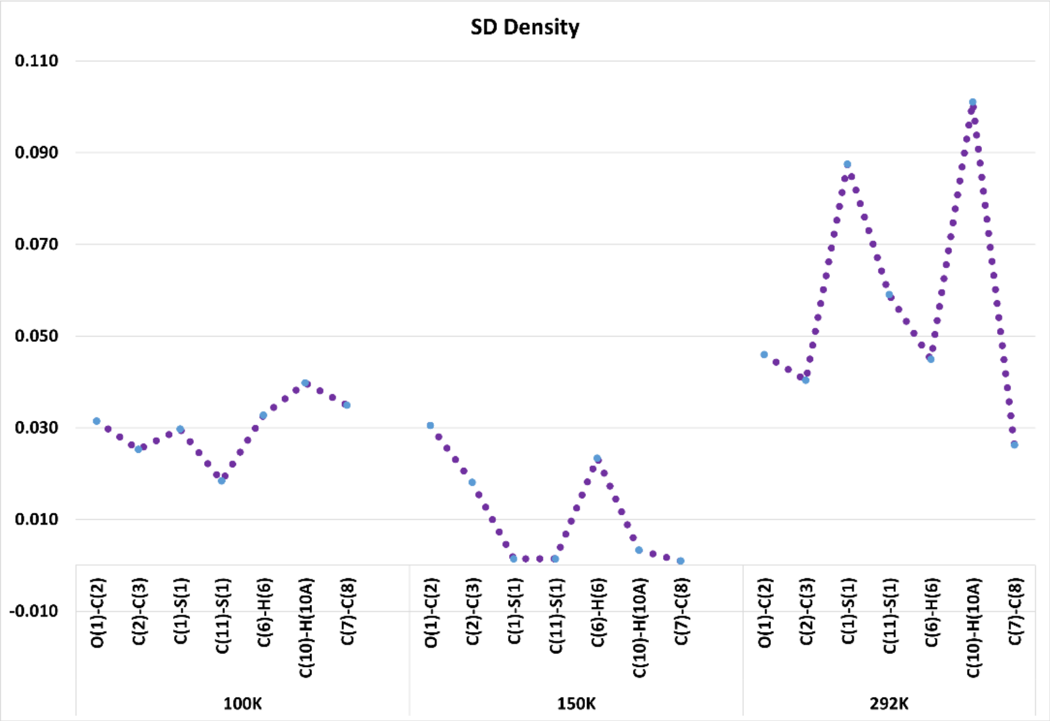
SD Density
0.110
0.090
0.070
0.050
0.030
0.010
-0.010
O(1)-C(2)
C(2)-C(3)
C(1)-S(1)
C(11)-S(1)
C(6)-H(6)
C(10)-H(10A)
C(7)-C(8)
100K
150K
292K

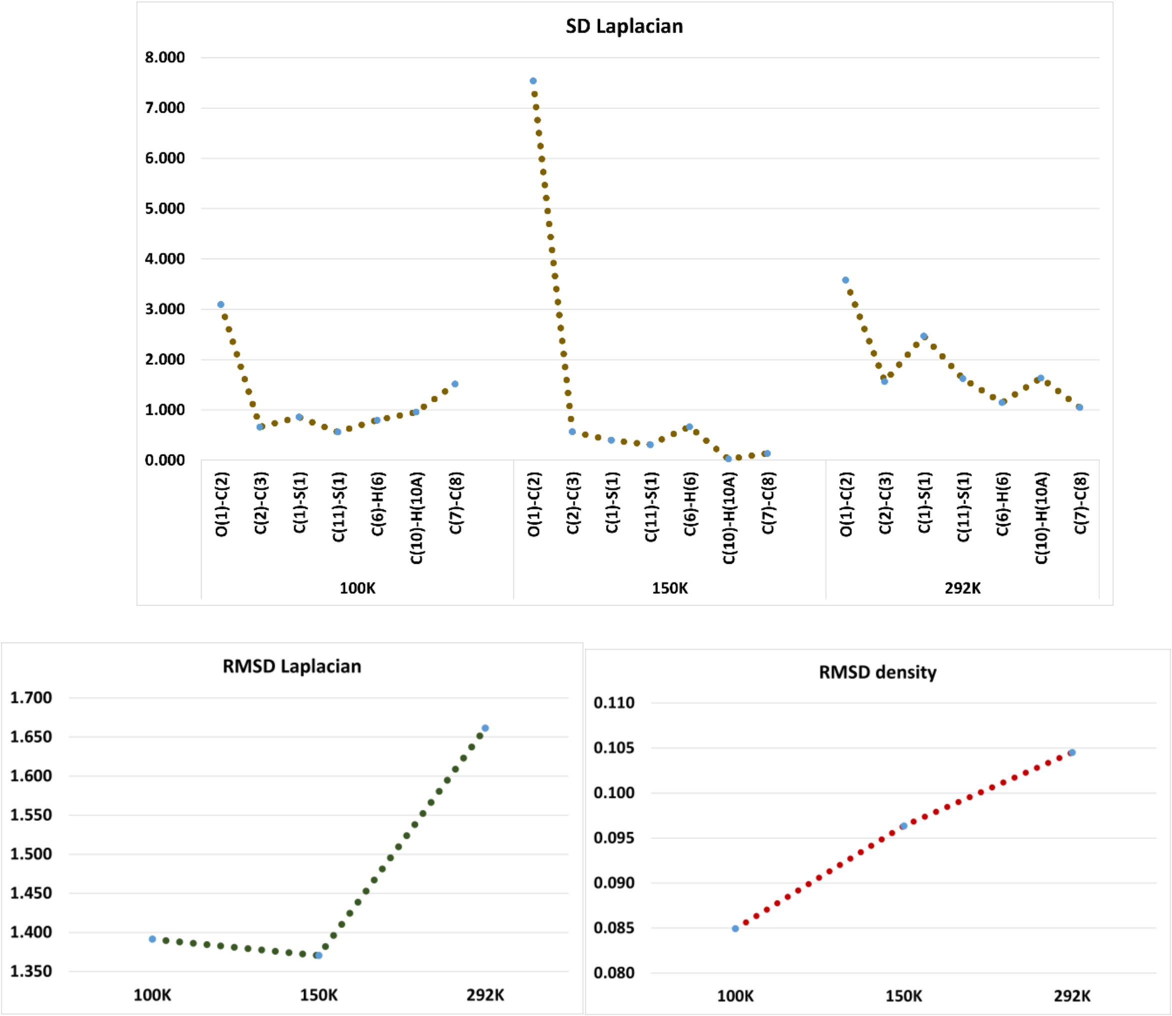

Figure S24. Plots of the statistical evaluation of topological properties of electron density for the sub-sets related to the temperature effect according to Table S23. Density = electron density at the bond critical point in eÅ^{-3}. Laplacian = Laplacian of the electron density at the bond critical point in eÅ^{-5}.

Table S24. Statistical evaluation of atomic properties Q001 (in e) and V001 (in Å^3) after XWR for the sub-sets related to the temperature effect. Selected datasets: 100K (datasets 1-8); 150K (datasets 9,10); 292K (datasets 11-23). The column SD refers to the sample standard deviation upon averaging the values of Q001 and V001 for the same atom across all selected datasets. RMSD(opt) refers to the root mean square deviation of the XWR results to the corresponding results from theoretical geometry optimization. Full list of values in Table S11.

**100K (Q001)**	**Average of selected datasets**	**SD of selected datasets**
S(1)	0.46	0.18
O(1)	-1.27	0.04
C(1)	-0.27	0.08
C(11)	-0.09	0.05
C(7)	-0.07	0.05
H(6)	0.07	0.03
H(10A)	0.11	0.03
RMSD (opt)	0.05	**-**
**100K (V001)**	**Average of selected datasets**	**SD of selected datasets**
S(1)	18.47	0.62
O(1)	20.27	0.44
C(1)	12.02	0.35
C(11)	10.81	0.31
C(7)	12.61	0.21
H(6)	6.92	0.27
H(10A)	6.17	0.53
RMSD (opt)	0.46	**-**
**150K (Q001)**	**Average of selected datasets**	**SD of selected datasets**
S(1)	0.34	0.10
O(1)	-1.25	0.05
C(1)	-0.23	0.02
C(11)	-0.14	0.01
C(7)	-0.09	0.01
H(6)	0.10	0.01
H(10A)	0.11	0.01
RMSD (opt)	0.07	**-**
**150K (V001)**	**Average of selected datasets**	**SD of selected datasets**
S(1)	19.15	0.43
O(1)	20.50	0.12
C(1)	11.98	0.13
C(11)	11.10	0.02

C(7)	12.81	0.074
H(6)	6.86	0.094
H(10A)	5.76	0.013
RMSD (opt)	0.52	-
**292K (Q001)**	**Average of selected datasets**	**SD of selected datasets**
S(1)	0.45	0.15
O(1)	-1.28	0.04
C(1)	-0.21	0.04
C(11)	-0.15	0.08
C(7)	-0.09	0.04
H(6)	0.09	0.03
H(10A)	0.12	0.02
RMSD (opt)	0.06	-
**292K (V001)**	**Average of selected datasets**	**SD of selected datasets**
S(1)	18.58	0.65
O(1)	20.57	0.67
C(1)	12.04	0.27
C(11)	11.20	0.34
C(7)	12.79	0.29
H(6)	6.83	0.39
H(10A)	6.31	0.37
RMSD (opt)	0.52	-

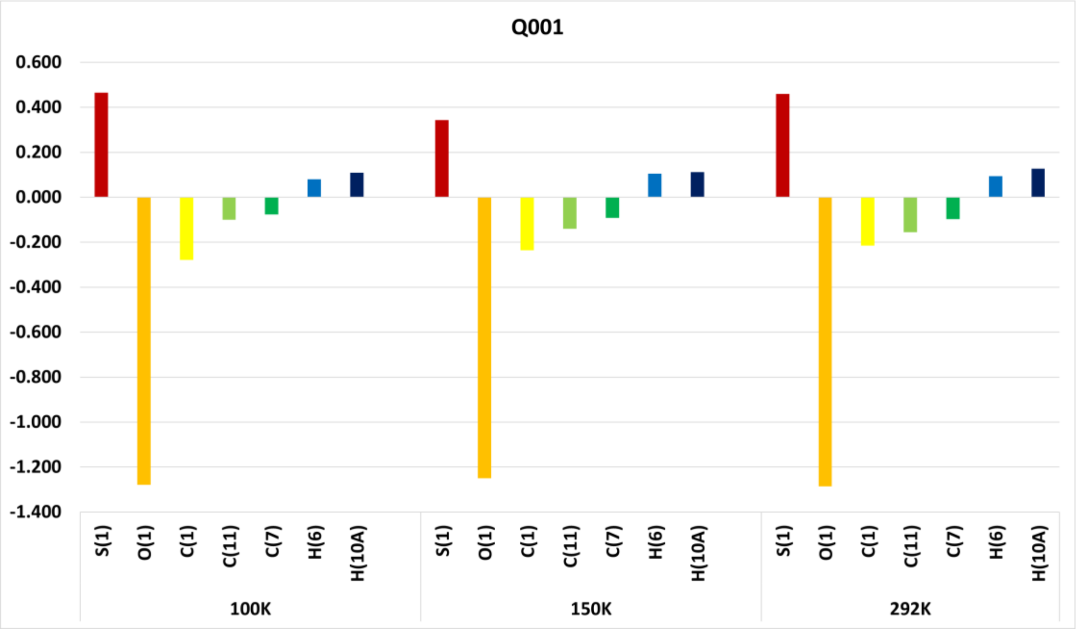

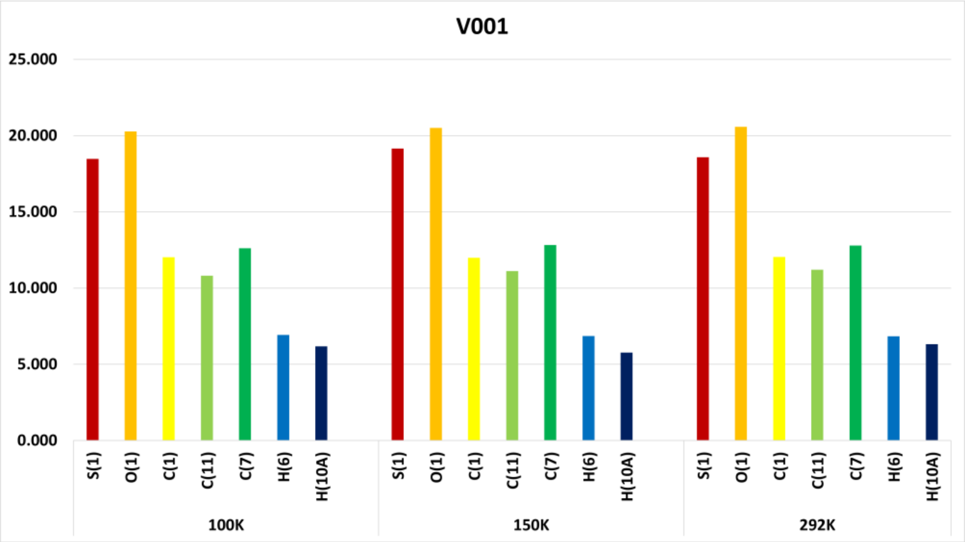
V001
25.000
20.000
15.000
10.000
5.000
0.000
S(1)
O(1)
C(1)
C(11)
C(7)
H(6)
H(10A)
100K
150K
292K

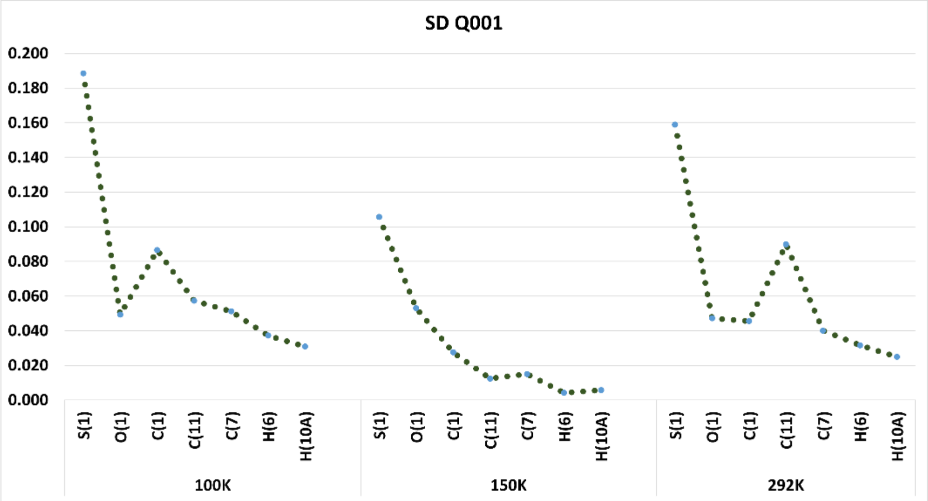
SD Q001
0.200
0.180
0.160
0.140
0.120
0.100
0.080
0.060
0.040
0.020
0.000
S(1)
O(1)
C(1)
C(11)
C(7)
H(6)
H(10A)
100K
150K
292K

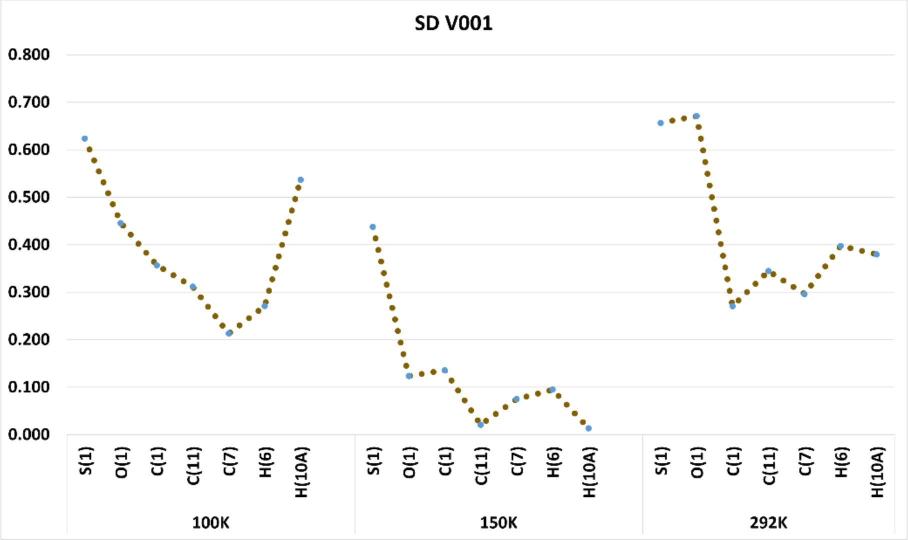
SD V001
0.800
0.700
0.600
0.500
0.400
0.300
0.200
0.100
0.000
S(1)
O(1)
C(1)
C(11)
C(7)
H(6)
H(10A)
100K
150K
292K

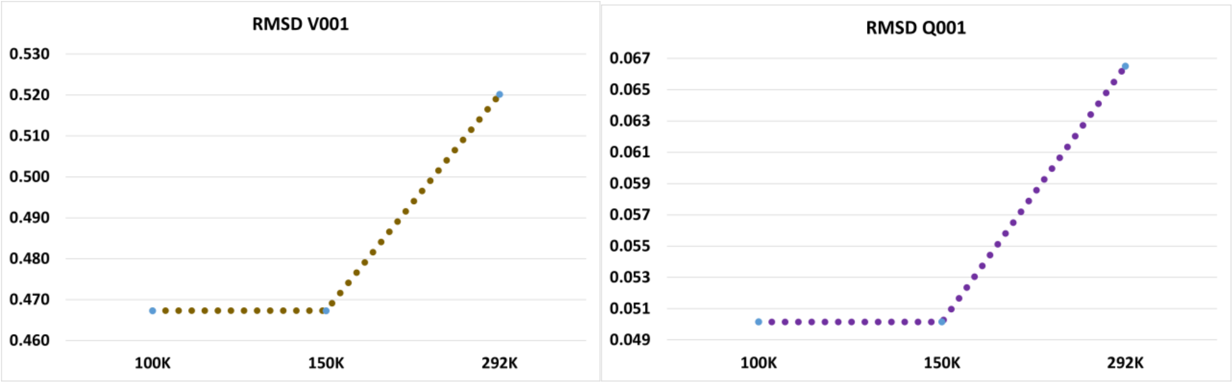

Figure S25. Plots of the statistical evaluation of atomic properties for the sub-sets related to the temperature effect according to Table S24. Q001 in e, V001 in Å^3.

Table S25. Similarity index (S) for ADPs calculated using the Shade3 server. This is a comparison between average ADPs from neutron diffraction data. The closer this value is to 1, the better the similarity.

Datasets	Mean S
Dataset 1	1.19
Dataset 2	2.15
Dataset 3	2.72
Dataset 4	2.12
Dataset 5	1.99
Dataset 6	2.10
Dataset 7	1.81
Dataset 8	1.34
Dataset 9	1.24
Dataset 10	1.06
Dataset 11	2.04
Dataset 12	3.09
Dataset 13	1.96
Dataset 14	2.18
Dataset 15	1.66
Dataset 16	2.58
Dataset 17	1.52

Dataset 18	2.09
Dataset 19	1.88
Dataset 20	2.98
Dataset 21	1.45
Dataset 22	2.13
Dataset 23	1.94

References used in the Supplementary Information

[1] Hansen, N. K. & Coppens, P. Testing aspherical atom refinements on small-molecule data sets. *Acta Cryst. A.* **34(6)**, 909-921 (1978).

[2] XD2016 - A computer program package for multipole refinement, topological analysis of charge densities and evaluation of intermolecular energies from experimental and theoretical structure factors. Volkov, A., Macchi, P., Farrugia, L. J., Gatti, C., Mallinson, P., Richter, T. & Koritsanszky, T. S. (2016)

[3] Jarzembska, K. N., & Dominiak, P. M. New version of the theoretical databank of transferable aspherical pseudoatoms, UBDB2011–towards nucleic acid modelling. *Acta Cryst. A.* **68**, 139-147 (2012).

[4] Allen, F. H. & Bruno, I. J. Bond lengths in organic and metal-organic compounds revisited: X—H bond lengths from neutron diffraction data. *Acta Cryst. B.* **66**, 380-386 (2010).

[5] Volkov, A., Abramov, Y. A. & Coppens, P. Density-optimized radial exponents for X-ray charge-density refinement from ab initio crystal calculations. *Acta Cryst. A.* **57**, 272–282 (2001).

[6] Madsen, A. Ø. & Hoser, A. A. SHADE3 server: a streamlined approach to estimate H-atom anisotropic displacement parameters using periodic ab initio calculations or experimental information. *J. Appl. Cryst.* **47**, 2100-2104 (2014).

[7] Woińska, M., Jayatilaka, D., Spackman, M. A., Edwards, A. J., Dominiak, P. M., Woźniak, K., Nishibori, E., Sugimoto, K. & Grabowsky, S. Hirshfeld atom refinement for modelling strong hydrogen bonds. *Acta Cryst. A.* **70**, 483-498 (2014).

[8] Pal, R., Jelsch, C., Momma, K. & Grabowsky, S. π-Hole bonding in a new co-crystal hydrate of gallic acid and pyrazine: static and dynamic charge density analysis. *Acta Cryst. B.* **78**, 231-246 (2022).

[9] Fugel, M., Jayatilaka, D., Hupf, E., Overgaard, J., Hathwar, V. R., Macchi, P., Turner, M. J., Howard, J. A. K., Dolomanov, O. V., Puschmann, H., Iversen, B. B., Bürgi, H.-B. & Grabowsky, S. Probing the accuracy and precision of Hirshfeld atom refinement with HARt interfaced with Olex2. *IUCrJ*. **5**, 32-44 (2018).

[10] Momma, K. & Izumi, F. VESTA 3 for three-dimensional visualization of crystal, volumetric and morphology data. *J. Appl. Cryst.* **44**, 1272-1276 (2011).

[11] Munshi, P., Madsen, A. Ø., Spackman, M. A., Larsen, S. & Destro, R. Estimated H-atom anisotropic displacement parameters: a comparison between different methods and with neutron diffraction results. *Acta Cryst. A.* **64**, 465-475 (2008).

[12] Kuhs, W. F. Generalized atomic displacements in crystallographic structure analysis, *Acta Cryst. A* **48**, 80-98 (1992).

[13] Woińska, M., Grabowsky, S., Dominiak, P. M., Woźniak, K. & Jayatilaka, D. Hydrogen atoms can be located accurately and precisely by x-ray crystallography. *Sci. Adv.* **2(5)**, e1600192 (2016).
