# Supplementary material for: A quantum crystallographic protocol for general use
Source: Sci Rep. 2025 Apr 19;15:13584. doi: 10.1038/s41598-025-96400-0 (PMC12009286; doi:10.1038/s41598-025-96400-0)

checkCIF/PLATON report

Structure factors have been supplied for datablock(s) 100_Mo_HAR

THIS REPORT IS FOR GUIDANCE ONLY. IF USED AS PART OF A REVIEW PROCEDURE FOR PUBLICATION, IT SHOULD NOT REPLACE THE EXPERTISE OF AN EXPERIENCED CRYSTALLOGRAPHIC REFEREE.

No syntax errors found. CIF dictionary Interpreting this report

Datablock: 100_Mo_HAR

Bond precision:	C-C = 0.0006 A		Wavelength=0.71073
Cell:	a=5.84810(5)	b=8.92817(7)	c=18.33261(16)
	alpha=90	beta=90	gamma=90
Temperature:	100 K		

	Calculated	Reported
Volume	957.198(14)	957.197(13)
Space group	P 21 21 21	P 21 21 21
Hall group	P 2ac 2ab	P 2ac 2ab
Moiety formula	C11 H10 O2 S	C11 H10 O2 S
Sum formula	C11 H10 O2 S	C11 H10 O2 S
Mr	206.25	206.27
Dx,g cm-3	1.431	0.000
Z	4	4
Mu (mm-1)	0.305	0.000
F000	432.0	432.0
F000'	432.69	
h,k,lmax	10,16,33	10,16,33
Nref	6328[3581]	3581
Tmin,Tmax		
Tmin'		

Correction method= Not given

Data completeness= 1.00/0.57 Theta(max)= 41.057

R(reflections)= 0.0157(3581) wR2(reflections)= wR= 0.0148(3581)

S = 1.477 Npar= 242

The following ALERTS were generated. Each ALERT has the format
test-name_ALERT_alert-type_alert-level.
Click on the hyperlinks for more details of the test.

Alert level A

PLAT924_ALERT_1_A The Reported and Calculated Rho(min) Differ by . 4.49 eA-3

Author Response: CheckCIF calculates the residual density based on tabulated spherical atomic form factors, whereas Tonto calculates it based on non-spherical atomic form factors. Therefore, differences must occur.

PLAT972_ALERT_2_A Check Calcd Resid. Dens. 0.05Ang From S1 -4.62 eA-3

Author Response: CheckCIF calculates the residual density based on tabulated spherical atomic form factors, whereas Tonto calculates it based on non-spherical atomic form factors. Therefore, differences must occur.

PLAT992_ALERT_5_A Repd & Actual _reflns_number_gt Values Differ by 124 Check

Author Response: This keyword calculates values with respect to F>4sigma(F). However, the refinement in Tonto does not use any cutoff and consequently produces all statistics based on F>0sigma(F). In the Tonto CIF, _reflns_number_gt and reflns_number_total and _refine_ls_number_reflns are always the same number. We do not alter these numbers just to pass the checkCIF routine.

Alert level B

PLAT230_ALERT_2_B Hirshfeld Test Diff for S1 --C1 . 10.5 s.u.

Author Response: This is a consequence of the non-spherical Hirshfeld Atom Refinement. It could only be avoided by using restraints, which are not available in Tonto. Since we compare models across different measurements statistically in this study, we decided to use the refinement results as they are.

PLAT230_ALERT_2_B Hirshfeld Test Diff for S1 --C10 . 14.8 s.u.

Author Response: This is a consequence of the non-spherical Hirshfeld Atom Refinement. It could only be avoided by using restraints, which are not available in Tonto. Since we compare models across different measurements statistically in this study, we decided to use the refinement results as they are.

```
PLAT230_ALERT_2_B Hirshfeld Test Diff for    S1       --C11      .       13.0 s.u.
```

Author Response: This is a consequence of the non-spherical Hirshfeld Atom Refinement. It could only be avoided by using restraints, which are not available in Tonto. Since we compare models across different measurements statistically in this study, we decided to use the refinement results as they are.

Alert level C

```
PLAT925_ALERT_1_C The Reported and Calculated Rho(max) Differ by .       1.12 eA-3
```

Author Response: CheckCIF calculates the residual density based on tabulated spherical atomic form factors, whereas Tonto calculates it based on non-spherical atomic form factors. Therefore, differences must occur.

Alert level G

```
PLAT005_ALERT_5_G No Embedded Refinement Details Found  in the CIF     Please Do !
PLAT142_ALERT_4_G s.u. on b - Axis Small or Missing .............    0.00007 Ang.
PLAT143_ALERT_4_G s.u. on c - Axis Small or Missing .............    0.00016 Ang.
PLAT808_ALERT_5_G No Parseable SHELXL Style Weighting Scheme Found     Please Check
PLAT883_ALERT_1_G Absent Datum for _atom_sites_solution_primary ..     Please Do !
PLAT929_ALERT_5_G No Weight Pars,Obs and Calc R1,wR2,S not Checked          ! Info
PLAT961_ALERT_5_G Dataset Contains no Negative Intensities .......     Please Check
PLAT962_ALERT_5_G Number  FCF File  Relections with Sigma(I) = 0.0         28 Note
PLAT969_ALERT_5_G The 'Henn et al.' R-Factor-gap value ..........      1.454 Note
              Predicted wR2: Based on SigI**2  2.02 or SHELX Weight  2.02
PLAT978_ALERT_2_G Number C-C Bonds with Positive Residual Density.          3 Info
PLAT980_ALERT_1_G No Anomalous Scattering Factors Found in CIF ...     Please Check
```

```
   3 ALERT level A = Most likely a serious problem - resolve or explain
   3 ALERT level B = A potentially serious problem, consider carefully
   1 ALERT level C = Check. Ensure it is not caused by an omission or oversight
  11 ALERT level G = General information/check it is not something unexpected

   4 ALERT type 1 CIF construction/syntax error, inconsistent or missing data
   5 ALERT type 2 Indicator that the structure model may be wrong or deficient
   0 ALERT type 3 Indicator that the structure quality may be low
   2 ALERT type 4 Improvement, methodology, query or suggestion
   7 ALERT type 5 Informative message, check
```

It is advisable to attempt to resolve as many as possible of the alerts in all categories. Often the minor alerts point to easily fixed oversights, errors and omissions in your CIF or refinement strategy, so attention to these fine details can be worthwhile. In order to resolve some of the more serious problems it may be necessary to carry out additional measurements or structure refinements. However, the purpose of your study may justify the reported deviations and the more serious of these should normally be commented upon in the discussion or experimental section of a paper or in the "special_details" fields of the CIF. checkCIF was carefully designed to identify outliers and unusual parameters, but every test has its limitations and alerts that are not important in a particular case may appear. Conversely, the absence of alerts does not guarantee there are no aspects of the results needing attention. It is up to the individual to critically assess their own results and, if necessary, seek expert advice.

Publication of your CIF in IUCr journals

A basic structural check has been run on your CIF. These basic checks will be run on all CIFs submitted for publication in IUCr journals (*Acta Crystallographica*, *Journal of Applied Crystallography*, *Journal of Synchrotron Radiation*); however, if you intend to submit to *Acta Crystallographica Section C* or *E* or *IUCrData*, you should make sure that full publication checks are run on the final version of your CIF prior to submission.

Publication of your CIF in other journals

Please refer to the *Notes for Authors* of the relevant journal for any special instructions relating to CIF submission.

PLATON version of 11/11/2024; check.def file version of 11/11/2024

Datablock 100_Mo_HAR - ellipsoid plot

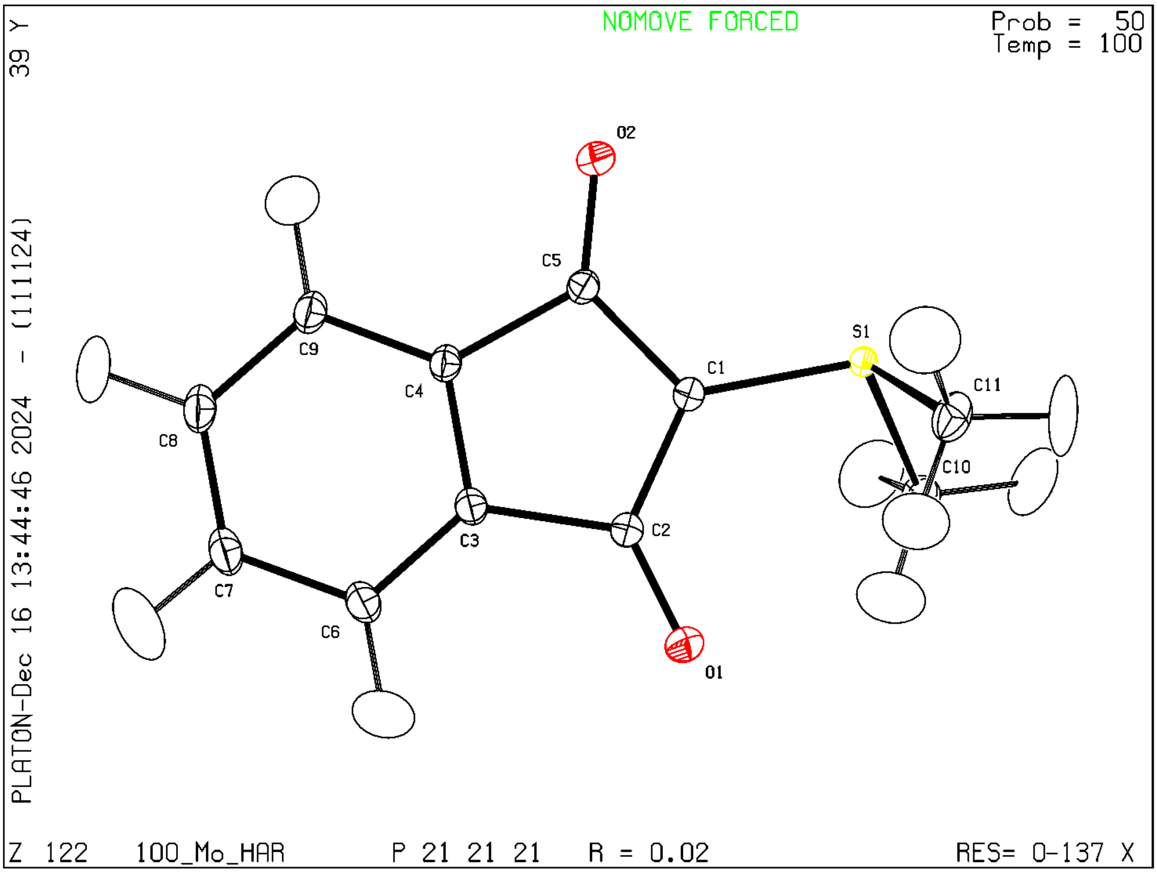

Supplement: Supplementary file 1 — Supplementary Information 1. [file Dataset_3_HAR.pdf › Dataset_3_HAR.pdf]
