# Supplementary material for: A quantum crystallographic protocol for general use
Source: Sci Rep. 2025 Apr 19;15:13584. doi: 10.1038/s41598-025-96400-0 (PMC12009286; doi:10.1038/s41598-025-96400-0)

checkCIF/PLATON report

Structure factors have been supplied for datablock(s) S_ylide_Cr5_quick2_

THIS REPORT IS FOR GUIDANCE ONLY. IF USED AS PART OF A REVIEW PROCEDURE FOR PUBLICATION, IT SHOULD NOT REPLACE THE EXPERTISE OF AN EXPERIENCED CRYSTALLOGRAPHIC REFEREE.

No syntax errors found.   CIF dictionary   Interpreting this report

Datablock: S_ylide_Cr5_quick2_

```
Bond precision:   C-C = 0.0002 A          Wavelength=0.24830

Cell:              a=5.8519(1)      b=8.9299(1)      c=18.3315(2)
                   alpha=90         beta=90          gamma=90
Temperature:       100 K

                Calculated                  Reported
Volume          957.95(2)                   957.95(2)
Space group     P 21 21 21                  P 21 21 21
Hall group      P 2ac 2ab                   P 2ac 2ab
Moiety formula  C11 H10 O2 S                C11 H10 O2 S
Sum formula     C11 H10 O2 S                C11 H10 O2 S
Mr              206.25                      206.27
Dx,g cm-3       1.430                       1.430
Z               4                           4
Mu (mm-1)       0.038                       0.037
F000            432.0                       431.8
F000'           431.92
h,k,lmax        13,20,42                    13,20,42
Nref            12117[ 6709]                6696
Tmin,Tmax       0.993,0.998                 0.540,1.000
Tmin'           0.993

Correction method= # Reported T Limits: Tmin=0.540 Tmax=1.000
AbsCorr = MULTI-SCAN

Data completeness= 1.00/0.55       Theta(max)= 16.540

                                                  wR2(reflections)=
R(reflections)= 0.0103( 6571)                     0.0249( 6696)

S = 1.298                   Npar= 242
```

The following ALERTS were generated. Each ALERT has the format
test-name_ALERT_alert-type_alert-level.
Click on the hyperlinks for more details of the test.

Alert level C

```
PLAT767_ALERT_4_C INS Embedded LIST 6 Instruction Should be LIST 4     Please Check
```

Author Response: For further XCW fittings in Tonto, LIST 6 is necessary to include the correction of anomalous dispersion effects in the fcf file.

```
PLAT910_ALERT_3_C Missing # of FCF Reflection(s) Below Theta(Min).          5 Note
                1  0  1,   0  1  1,   0  0  2,   0  1  2,   0  1  3,
PLAT911_ALERT_3_C Missing FCF Refl Between Thmin & STh/L=    0.600         13 Report
                3  2  0,   4  6  0,   3  9  0,   0  8  3,   0  0  4,   4  0  8,
                4  1  9,   4  0 10,   2  0 12,   4  0 12,   0  5 13,   0  4 15,
                0  2 18,
PLAT914_ALERT_3_C No Bijvoet Pairs in FCF for Non-centro Structure     Please Check
```

Alert level G

```
ABSMU01_ALERT_1_G  Calculation of _exptl_absorpt_correction_mu
                not performed for this radiation type.
PLAT003_ALERT_2_G Number of Uiso or U(i,j)  Restrained non-H-Atoms          3 Report
PLAT019_ALERT_1_G _diffrn_measured_fraction_theta_full/*_max < 1.0      0.985 Report
PLAT187_ALERT_4_G The CIF-Embedded .res File Contains RIGU Records          2 Report
PLAT190_ALERT_3_G A Non-default RIGU Restraint Value for First Par     0.0000 Report
PLAT190_ALERT_3_G A Non-default RIGU Restraint Value for SecondPar     0.0000 Report
PLAT190_ALERT_3_G A Non-default RIGU Restraint Value for First Par     0.0001 Report
PLAT190_ALERT_3_G A Non-default RIGU Restraint Value for SecondPar     0.0001 Report
PLAT802_ALERT_4_G CIF Input Record(s) with more than 80 Characters          1 Info
PLAT860_ALERT_3_G Number of Least-Squares Restraints ............           6 Note
PLAT872_ALERT_4_G ALERTS Related to Anharmonic Refine   Suppressed          ! Info
PLAT881_ALERT_1_G No Datum for _diffrn_reflns_av_R_equivalents ...     Please Do !
PLAT883_ALERT_1_G Absent Datum for _atom_sites_solution_primary ..     Please Do !
PLAT933_ALERT_2_G Number of HKL-OMIT Records in Embedded .res File         13 Note
               -4  1  9,   0  2 18,   0  4 15,   0  5 13,   0  8  3,   2  0 12,
                3  2  0,   3  9  0,   4  0  8,   4  0 10,   4  0 12,   4  1  9,
                4  6  0,
PLAT961_ALERT_5_G Dataset Contains no Negative Intensities .......     Please Check
PLAT963_ALERT_2_G Both SHELXL WEIGHT Parameter Values Zero .......     Please Check
PLAT969_ALERT_5_G The 'Henn et al.' R-Factor-gap value ..........       1.298 Note
              Predicted wR2: Based on SigI**2  1.92 or SHELX Weight  1.92
PLAT978_ALERT_2_G Number C-C Bonds with Positive Residual Density.         10 Info
PLAT979_ALERT_1_G NoSpherA2 Scattering Factors Used .............      Please Note
```

0 **ALERT level A** = Most likely a serious problem - resolve or explain
0 **ALERT level B** = A potentially serious problem, consider carefully
4 **ALERT level C** = Check. Ensure it is not caused by an omission or oversight
19 **ALERT level G** = General information/check it is not something unexpected

5 ALERT type 1 CIF construction/syntax error, inconsistent or missing data

```
4 ALERT type 2 Indicator that the structure model may be wrong or deficient
8 ALERT type 3 Indicator that the structure quality may be low
4 ALERT type 4 Improvement, methodology, query or suggestion
2 ALERT type 5 Informative message, check
```

It is advisable to attempt to resolve as many as possible of the alerts in all categories. Often the minor alerts point to easily fixed oversights, errors and omissions in your CIF or refinement strategy, so attention to these fine details can be worthwhile. In order to resolve some of the more serious problems it may be necessary to carry out additional measurements or structure refinements. However, the purpose of your study may justify the reported deviations and the more serious of these should normally be commented upon in the discussion or experimental section of a paper or in the "special_details" fields of the CIF. checkCIF was carefully designed to identify outliers and unusual parameters, but every test has its limitations and alerts that are not important in a particular case may appear. Conversely, the absence of alerts does not guarantee there are no aspects of the results needing attention. It is up to the individual to critically assess their own results and, if necessary, seek expert advice.

Publication of your CIF in IUCr journals

A basic structural check has been run on your CIF. These basic checks will be run on all CIFs submitted for publication in IUCr journals (*Acta Crystallographica*, *Journal of Applied Crystallography*, *Journal of Synchrotron Radiation*); however, if you intend to submit to *Acta Crystallographica Section C* or *E* or *IUCrData*, you should make sure that full publication checks are run on the final version of your CIF prior to submission.

Publication of your CIF in other journals

Please refer to the *Notes for Authors* of the relevant journal for any special instructions relating to CIF submission.

PLATON version of 11/11/2024; check.def file version of 11/11/2024

Datablock S_ylide_Cr5_quick2_ - ellipsoid plot

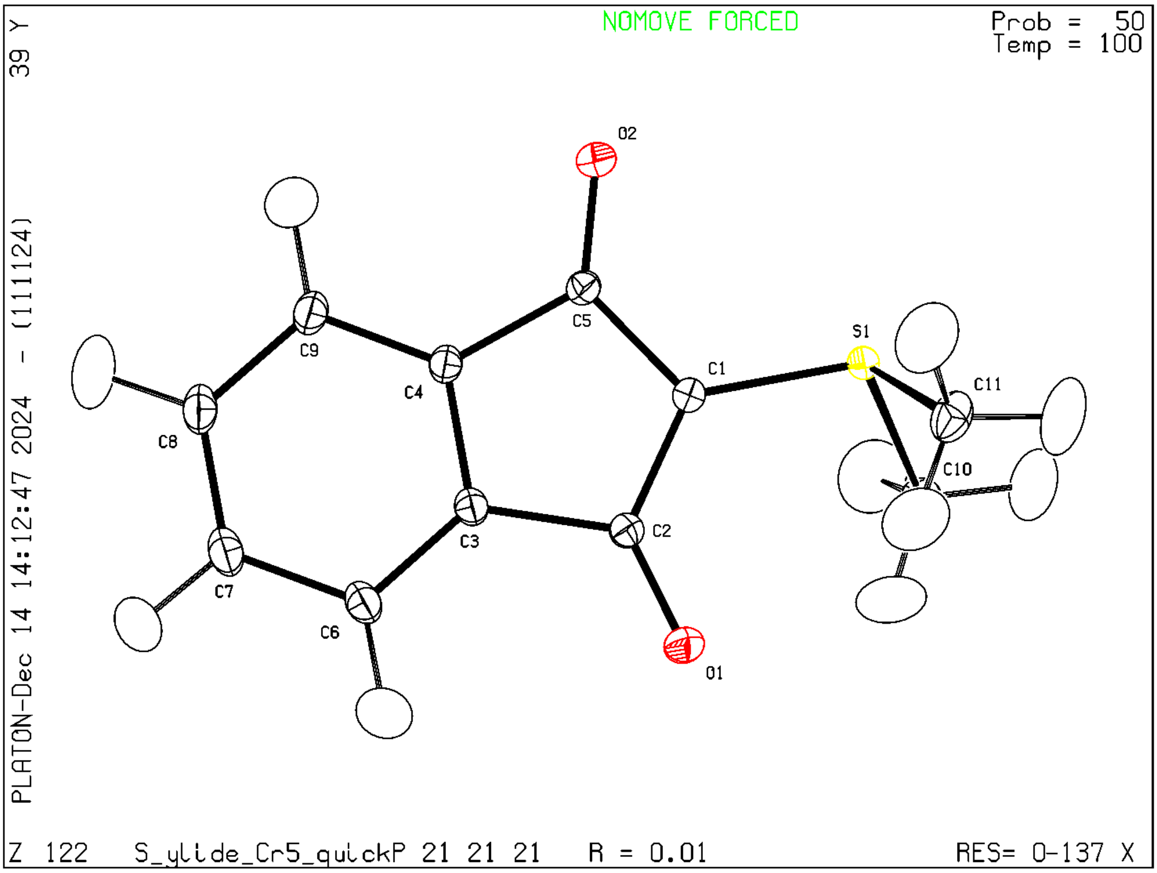

Supplement: Supplementary file 1 — Supplementary Information 1. [file Dataset_7_HAR.pdf › Dataset_7_HAR.pdf]
